# A systematic summary and comparison of animal models for chemotherapy induced (peripheral) neuropathy (CIPN)

**DOI:** 10.1371/journal.pone.0221787

**Published:** 2019-08-28

**Authors:** Suvarna Gadgil, Mehmet Ergün, Sandra A. van den Heuvel, Selina E. van der Wal, Gert Jan Scheffer, Carlijn R. Hooijmans

**Affiliations:** 1 Department of Anesthesiology, Pain and Palliative Medicine, Radboud University Medical Center, Nijmegen, The Netherlands; 2 Department for Health Evidence unit SYRCLE, Radboud University Medical Center, Nijmegen, the Netherlands; University of Texas Medical Branch at Galveston, UNITED STATES

## Abstract

Despite the large amount of human and experimental studies no effective (prophylactic) treatment exists for chemotherapy induced peripheral neuropathy (CIPN), a disabling side effect of many cancer treatments. One of the underlying reasons for this could be that often the preclinical animal models used are not the best representation of the clinical situation. We therefore present a systematic summary and comparison of all animal models currently described in literature for CIPN focusing on stimulus evoked pain-like behaviour and neurophysiological alterations in nerve function (650 included papers, and a comparison of 183 models), that resulted in a clear overview of the most effective and robust CIPN models using an administration route used in clinical practice. Using our three-step approach (step 1: efficacy; step; 2 robustness and step 3: mimicking the clinical situation) we show that all mice CIPN models treated with either paclitaxel or cisplatin using an administration route used in clinical practice seem suitable models. Three specific models using paclitaxel or cisplatin that stand out are 1) C57BL/6 female mice receiving paclitaxel and 2) CD1 male mice receiving paclitaxel and 3) C57BL/6 male mice receiving cisplatin. This overview may help scientists selecting suitable CIPN models for their research. We hypothesize that by using effective and robust animal models that mimic the clinical situation as much as possible, the translational value of preclinical study results with respect to the potential of identifying promising treatments for CIPN in the future, will prove. The methodology described in this paper, aimed at comparing animal models, is novel and can be used by scientist in other research fields as well.

## Introduction

Over the last decades, there has been a remarkable increase in the long-term survival rate in cancer patients due to improvement in early detection, precise subtype characterization and development of new treatment options [1, 2], An emerging issue in cancer treatment is dealing with long-term sequelae that impair quality of life in cancer patients and survivors [3, 4]. One of the most common reported symptoms derived from cancer treatment is chemotherapy induced peripheral neuropathy (CIPN), which is caused by differential damage to peripheral nervous system depending on the administered neurotoxic antineoplastic agent, such as taxanes, platinum compounds, vinca alkaloids, epothilones, protease inhibitors and thalidomide [5, 6]. It mainly presents as a dose-dependent sensory length-dependent neuropathy with symptoms including numbness, paresthesias, loss of proprioception and hyperalgesia; less often patients will present with motor weakness and autonomic changes [7, 8].

The incidence of CIPN varies between type and dose of chemotherapeutic agents, method of assessment [9] and period after cessation of chemotherapy [10–12]. At 6 months roughly 30% up to 71% of patients continue to suffer from CIPN [9, 13]. Some patient groups with neuropathy at baseline, older age, genetic varieties or prior exposure to neurotoxic agents are more at risk for developing CIPN [13, 14]. Neuropathic symptoms may lead to dose reduction or early cessation of chemotherapy, thereby potentially impacting patient survival. Despite the large amount of human and experimental studies, until now no effective prophylactic treatment exists [15–17], treatment of CIPN related pain remains difficult [5, 15, 16, 18] and reduces quality of life of cancer patients and survivors [19].

One of the underlying reasons for this could be the fact that often not all laboratory work done in animals hasn’t been evaluated before deciding to apply it to patients. As a consequence, specific treatment options might be either discarded for testing in humans or are erroneously selected resulting in a mismatch between preclinical and clinical benefit. In addition, the animal models and experimental design used in the preclinical phase may not be the best representation of the clinical situation possibly leading to translational failure. In case of CIPN for example, often doses and mode of deliveries of the chemotherapeutic agents are used that may not representative of the clinical situation, and many preclinical studies do not seem to be adequately randomised and blinded.

To identify possible treatment strategies for CIPN, in either the already published literature or by conducting new animal studies, it is urgent to identify and compare the efficacy and relevance to the clinical situation of all CIPN animal models used in literature so far and select the most promising ones.

Therefore, we conducted a systematic review of all animal studies published so far in the field of CIPN and developed a strategy to compare the resulting animal models.

## Results

### Study selection

**Fig 1:**
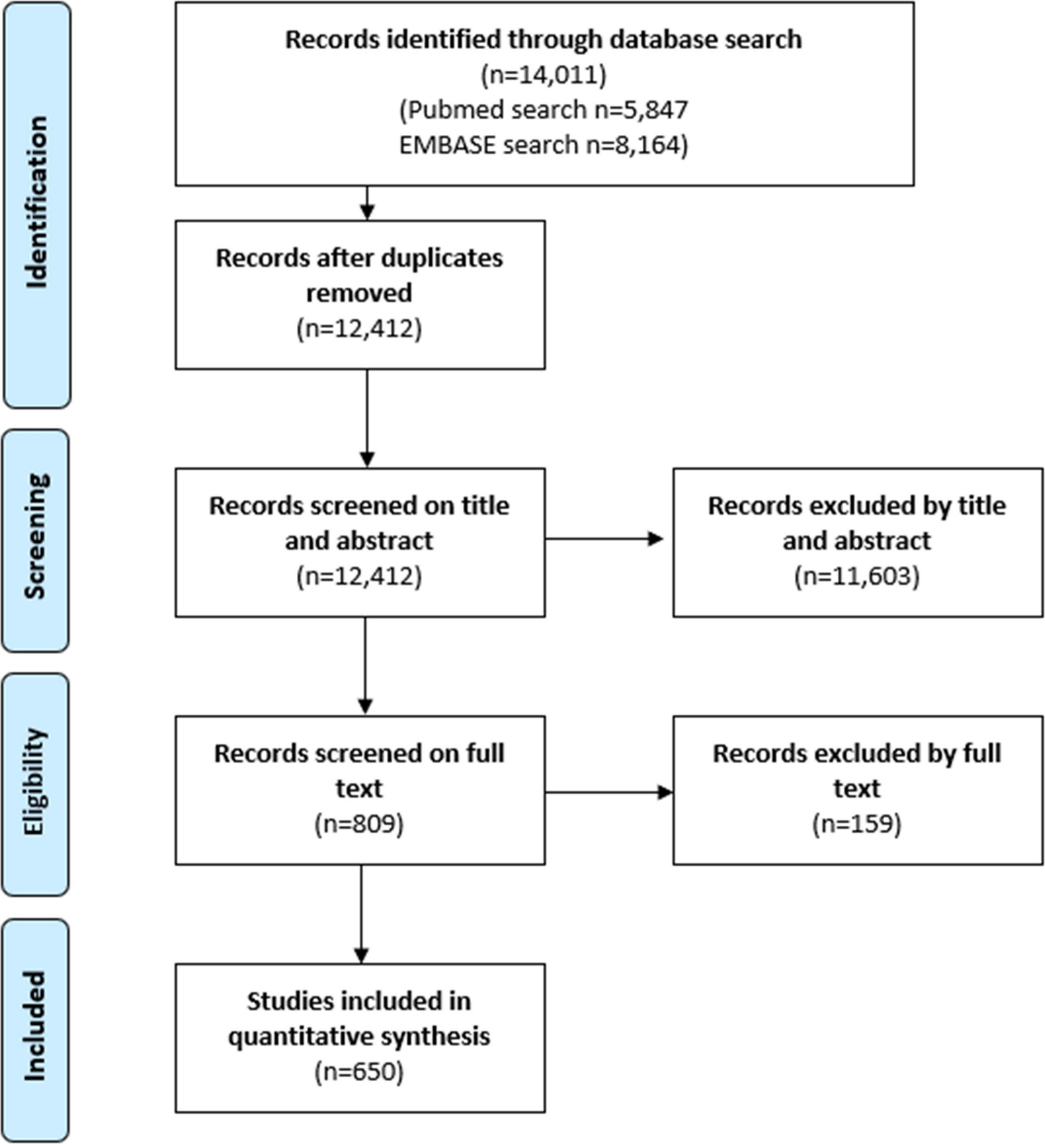
Flowchart diagram.

Fig 1 shows that the electronic search strategy retrieved 5,847 records from PubMed and 8,164 articles from EMBASE. Out of 12,412 unique references, 809 papers were included after screening of the title and abstracts. Out of these 809 papers, 650 met our predefined inclusion criteria. The references of the included studies can be found in S1 File.

### Study characteristics

Based on the species, strain, sex and type of chemotherapy used, the various CIPN models used in literature were identified. A total of 183 unique CIPN models were identified (S1 Table). The number of times that the models were used in these 650 included papers was also counted and resulted in 1,023 comparisons.

#### Animal characteristics

In these 183 different models, twelve different species were used. 85.2% of the models conducted experiments in rodents (92 in mice and 64 in rat) and 14.8% were conducted in other species: guinea pigs (n = 6); cats (n = 4); chickens (n = 3), cynomolgus monkey (n = 3), rhesus monkey (n = 1), rabbits (n = 3), drosophila melanogaster (n = 3), dogs (n = 2), catfish (n = 1) and zebrafish (n = 1). Forty different strains amongst the different species have been used.

Data were predominantly obtained from male animals (697 (68%) out of 1023 comparisons). Regarding the 183 different models, 69 (37.7%) models used male animals. Females were used in 45 models (24.6%), and 34 (18.6%) models used mixed sex groups. Unfortunately in 35 (19.1%) models the sex was not described.

#### Chemotherapy characteristics

Twenty seven different types of chemotherapy had been used among these 183 models: 1) 28.4% of the models used a platinum based chemotherapy (e.g. cisplatin (n = 29), oxaliplatin (n = 20), carboplatin (n = 1), lipoplatin (n = 1) and ormaplatin (n = 1)); 2) 28.4% of the models used taxanes (e.g. paclitaxel (n = 46), docetaxel (n = 5) and nab-paclitaxel (n = 1)); 3) 26.8% of the models used a vinca-alkaloid based chemotherapy (vincristine (n = 38), vinblastine (n = 6), vindesine (n = 4) and vinorelbine (n = 1)) and 4) 16.4% of the models used another type of chemotherapy (e.g. bortezomib (n = 10), methotrexate (n = 1), etoposide (n = 1), thalidomide (n = 2), ethoglucid (n = 1), epothilone-B (n = 1), eribulin (n = 1), gemcitabine (n = 2), ixabepilone (n = 1), quelamycin (n = 1), sorafenib (n = 1), suramin (n = 1), doxorubicin (n = 4), salinomycin (n = 1) and tamoxifen (n = 2)).

#### Route of administration

Also the route of administration varied greatly across and within the models (e.g. a CIPN animal model was initially defined based on species, strain, sex and type of chemotherapy used, thus within a model the same chemotherapeutic agent could be administered in various ways).

In order to analyse the variation in administration route we added route of administration as a factor in the specification of the various CIPN models used in literature. This resulted in 222 different CIPN models.

Seven different routes of administration had been used: intraperitoneal (59.5%), intravenous (27.5%), subcutaneous (6,3%), oral (1,8%), intramuscular (0,5%), intradermal (0,5%), and intraventricular (0,5%). In 3,6% of the models failed to report the route of administration and hence were classified as unknown.

#### Polyneuropathy characteristics

In order to assess later in this review whether or not a model caused CIPN we extracted and analysed whether or not a study measured one of the following outcomes: mechanical allodynia, thermal hyper and hypoalgesia, motor function and/or electrophysiological measurements and histological damage to the peripheral nervous system.

Overall 28.430% (n = 52) models used only one of the above-mentioned outcomes to assess CIPN. The other 71.6% assessed two or more outcomes. 34.4% (n = 63) used two tests, 12.6% (n = 23) used three tests, 13.1% (n = 24) used four tests and 11.5% (n = 21) used all five tests.

### Step 1: Efficacy of the CIPN models

In order to assess how effective the CIPN models used in literature are in causing polyneuropathy the efficacy of each model was calculated (% of CIPN on two of our (peripheral) polyneuropathy outcomes), and subsequently the animal models with promising efficacy based on the following criteria: 1) the animal model should at least have been tested five times; 2) the animal model causes significant (peripheral) polyneuropathy in at least 90% of the experiments in two or more outcomes, were selected, and tabulated.

S1 Table provides an overview of the efficacy of CIPN models ranked by animal species.

S2 Table provides an overview of the efficacy of CIPN models ranked by type of chemotherapy used.

S3 Table provides a summary of the efficacy of the models that might be promising, as they scored significant (peripheral) polyneuropathy in at least 90% of the experiments in two or more outcomes but did not reach our reproducibility limit (n = 5).

#### The Big Five models based on efficacy

This above-mentioned analyses resulted in five promising models (Table 1). Rodents were used in all of these models (rats 60%, mice 40%). Two models used taxane (paclitaxel n = 2), two models used a platinum-based chemotherapy (cisplatin n = 1, oxaliplatin n = 1) and one model used a vinca-alkaloid (vincristine n = 1). Three models showed in 100% of the studies polyneuropathy (in at least two out of our five outcomes) after chemotherapy (Table 1).

**Table 1:** Overview of the “Big Five”–most effective animal models of chemotherapy-induced polyneuropathy, defined by species, strain, chemotherapy and sex.

Species	Strain	Chemotherapy	Sex	n_1	CIPN_1 (%)	n_>1	CIPN_>1 (%)
rats	Wistar	vincristine	both	10	100%	10	100%
mice	CD1	paclitaxel	male	9	100%	6	100%
mice	C57BL/6	paclitaxel	female	13	100%	8	100%
rats	Wistar	cisplatin	female	34	94%	18	94%
rats	Sprague Dawley	oxaliplatin	male	104	100%	62	92%

Most effective animal models of chemotherapy-induced polyneuropathy based on calculating the efficacy of each model in causing polyneuropathy. CIPN model are defined by species, strain, chemotherapy and sex. n: number of experiments using a specific model. _1: chemotherapy-induced polyneuropathy in one of the predefined outcomes. _>1: chemotherapy-induced polyneuropathy in more than one of the predefined outcomes (e.g. mechanical allodynia, thermal hyper and hypoalgesia, sensory-motor coordination, electrophysiological measurements and/or histological damage to the peripheral nervous system

***Rats Wistar, both sexes, receiving vincristine***

A total of ten experiments used this model. All experiments showed significant polyneuropathy (100%) in at least two outcomes for polyneuropathy. All five predefined outcomes for polyneuropathy have been studied in this model and they all showed in all comparisons significant polyneuropathy. In this model all tests have been conducted e.g. thermal hyper and hypoalgesia (n = 10 (100%)), mechanical allodynia (n = 10 (100%)), histological damage to the peripheral nervous system (n = 5 (83%)), sensory-motor coordination impairment (n = 2 (100%)) and electrophysiological impairments (n = 1 (100%)). All experiments administered vincristine intraperitoneally (100%). The cumulative dosage of vincristine ranged from 0,5 mg/kg (n = 7) up to 1 mg/kg (n = 2), with in between 0,75 mg/kg (n = 1).

***Mice CD1, male, receiving paclitaxel***

A total of nine experiments used this CIPN model, whereof a total of six experiments measured polyneuropathy in at least two outcomes. All experiments showed significant polyneuropathy (100%). The age of the mice ranged from 4 to 52 weeks. In a two experiments age was not reported. The cumulative dosage ranged from 8 mg/kg (n = 2) up to 126,6 mg (n = 1), with in between 10 mg/kg (n = 5) and 16 mg/kg (n = 1). Route of administration was intraperitoneal in eight experiments (89%) and subcutaneous in one experiment (11%).

Four of our predefined outcomes have been assessed in this model e.g. assessment of histological damage to the peripheral nervous system (n = 4 (100%)), electrophysiological impairments (n = 1 (100%)), thermal hyper or hypoalgesia (n = 7 (100%)) and mechanical allodynia (n = 7 (100%)). Sensory-motor coordination tests were not conducted in this model.

***Mice C57BL/6, female, receiving paclitaxel***

A total of thirteen experiments have reportedly been performed, whereof a total of eight experiments measured polyneuropathy in at least two outcomes. All thirteen experiments showed significant polyneuropathy (100%)

The age of the mice ranged from 5 weeks up to adulthood. In six experiments age was not reported. Cumulative dosage ranged from 4 mg/kg (n = 1) up to 180 mg/kg (n = 3), with in between 8 mg/kg (n = 1), 16 mg/kg (n = 2), 18 mg/kg (n = 2), 32 mg/kg (n = 3) and 70 mg/kg (n = 1). Route of administration was intraperitoneal in ten experiments (77%) and intravenous in three experiments (23%).

In this model all predefined outcomes have been assessed e.g. histological damage to the peripheral nervous system (n = 4 (100%)), electrophysiological measurements (n = 3 (100%)), thermal hyper and hypoalgesia (n = 6 (100%)), sensory-motor coordination impairment (n = 4 (80%)) and mechanical allodynia (n = 7 (100%)).

***Rats Wistar, female, receiving cisplatin***

A total of thirty-seven experiments have used this model. Nineteen experiments measured polyneuropathy in at least two outcomes. Ninety-five percent of the comparisons showed significant polyneuropathy. All 5 predefined outcomes were assessed. In 95% of the comparisons that studied histological damage to the peripheral nervous system significant damage was observed. Electrophysiological impairment and mechanical allodynia were observed in all comparisons (electrophysiological testing n = 27, and mechanical allodynia n = 2). Seventy five percent of the experiments showed significant thermal hyper and hypoalgesia (n = 3) or sensory motor impairment (n = 3).

The age of the mice ranged from 8 weeks up to 13 weeks. In eighteen experiments age was not reported. Cumulative dosage ranged from 0,0528 mg/kg (n = 1) up to 32 mg/kg (n = 2), with in between 4 mg/kg (n = 1), 7 mg/kg (n = 3), 9 mg/kg (n = 1), 12 mg/kg (n = 1), 15 mg/kg (n = 1), 16 mg/kg (n = 8), 18 mg/kg (n = 8), 20 mg/kg (n = 5), 22 mg/kg (n = 2), 23 mg/kg (n = 1) and 5 mg (n = 1).

In 36 experiments (97%) the route of administration was intraperitoneal. Only one study (3% administered cisplatin intravenously.

***Rats Sprague Dawley, male, receiving oxaliplatin***

A total of 104 experiments used this model. Sixty-two experiments measured polyneuropathy in at least two outcomes. In 92% of the comparisons significant polyneuropathy was observed (92%). All predefined outcomes were assessed. Mechanical allodynia was predominantly studied (n = 89 (100%)). Significant thermal hyper and hypoalgesia was observed in 90% of the comparisons (n = 68). Histological damage to the peripheral nervous system and electrophysiological impairment was observed in all comparisons (histological damage n = 24; electrophysiological damage n = 8). Eight out of 24 experiments showed sensory motor impairment.

The age of the mice ranged from 5 weeks up to adulthood. In sixty-nine experiments age was not reported. Cumulative dosage ranged from 0,002 mg/kg (n = 1) up to 90 mg/kg (n = 1), with in between 0,004 mg/kg (n = 1), 0,01 mg/kg (n = 1), 0,02 mg/kg (n = 1), 0,5mg/kg (n = 1), 1 mg/kg (n = 1), 2 mg/kg (n = 5), 3 mg/kg (n = 1), 4 mg/kg (n = 2), 5 mg/kg (n = 1), 6 mg/kg (n = 17), 8 mg/kg (n = 7), 9 mg/kg (n = 1), 10 mg/kg (n = 8), 12mg/kg (n = 1), 15mg/kg (n = 1), 16 mg/kg (n = 4), 18mg/kg (n = 3), 19,2 mg/kg (n = 1), 20mg/kg (n = 2), 21mg/kg (n = 1), 24 mg/kg (n = 4), 30mg/kg (n = 1), 32 mg/kg (n = 15), 36 mg/kg (n = 18), 50,4 mg/kg (n = 1), 64 mg/kg (n = 1) and 72 mg/kg (n = 1). Route of administration was intraperitoneal in 85 experiments (82%), intravenous in fifteen experiments (14%) and intradermal in four experiments (4%).

### Step 2: Robustness of the CIPN models

In order to assess the robustness of our “big five” models, the data set was reanalysed using different categories to specify the various CIPN models used (e.g. strain and/or sex included in the specification of CIPN models). Our analysis showed that three of our five initially selected models appear either in all four or in at least three analyses as most effective models. These three models are:

1. Mice C57BL/6, female, receiving paclitaxel; appeared as effective model in 4 out of 4 analyses using different categories to define CIPN models.
2. Mice CD1, male, receiving paclitaxel; appeared as effective model in 4 out of 4 analyses with using different categories to define CIPN models.
3. Rats Sprague Dawley, male, receiving oxaliplatin; appeared as effective model in three out of four analyses using different categories to define CIPN models (Table 2), indicating that this model appears robust across sex, but not very robust across strains (e.g. not reaching the 90% significance level for peripheral neuropathy in 2 or more outcomes).

**Table 2:** Overview of the efficacy and number of comparisons per model of CIPN models ranked animal species.

	Species	Strain	Chemotherapy	Sex	n	CIPN	
species, strain, chemotherapy, sex	Mice	C57BL/6	Paclitaxel	Female	8	8 (100%)	4
	Mice	CD1	Paclitaxel	Male	6	6 (100%)	4
	Rats	SD	Oxaliplatin	Male	62	57 (92%)	3
	Rats	Wistar	Cisplatin	Female	19	18 (95%)	1
	Rats	Wistar	Vincristine	Both	10	10 (100%)	2
species, chemotherapy, sex	Mice	-	Paclitaxel	Male	33	30 (91%)	4
	Mice	-	Paclitaxel	Female	21	19 (90%)	4
	Mice	-	Paclitaxel	Both	5	5 (100%)	2
species, strain, chemotherapy	Mice	CD1	Paclitaxel	-	10	10 (100%)	4
	Mice	C57BL/6	Paclitaxel	-	30	28 (93%)	4
	Rats	Wistar	Vincristine	-	15	15 (100%)	2
	Rats	SD	Oxaliplatin	-	63	58 (92%)	3
species, chemotherapy	Mice	-	Bortezomib	-	10	9 (90%)	1
	Mice	-	Cisplatin	-	29	26 (90%)	1
	Mice	-	Paclitaxel	-	64	58 (91%)	4
							4
							2
	Rats	-	Oxaliplatin	-	86	77 (90%)	3

n: number of experiments using a specific model. CIPN: number of experiments using a specific model for chemotherapy induced peripheral neuropathy showing efficacy in more than one of the predefined outcomes (e.g. mechanical allodynia, thermal hyper and hypoalgesia, sensory-motor coordination, electrophysiological measurements and/or histological damage to the peripheral nervous system). The colour code in the last column indicates how often a model appeared as an effective model when using different categories to define CIPN models. Abbreviations: Sprague Dawley (SD)

However, it is important to realize that the number of variations in models used within a category and the amount of available evidence per model largely influences our results. For example, the rat model receiving oxaliplatin (effective model in three out of four analyses using different categories to define CIPN models) appears not so robust across strains (the model did not appear as effective in the analyses using solely, species, chemotherapy and sex to define a CIPN model), and robust across sex of the animals used. However, when taking into account the variations in models within a category, it becomes clear that oxaliplatin was solely tested in males. Therefore, we can’t draw any conclusions on the robustness of this model regarding sex of the animals used. Thus, based on the poor robustness across strains and unclear robustness across sex this model is no longer considered one of the most suitable animal models to study CIPN.

Paclitaxel was tested in nineteen strains of mice. As shown in Table 2 paclitaxel causes significant (peripheral) polyneuropathy in 91% of the experiments using males, and 90% using females.

In 4 strains of mice was paclitaxel tested in both male and female groups separately (e.g. CD1, C57Bl6, AJ and Balb C mice).

Thus, mice CIPN models treated with paclitaxel show efficacy across various strains and sex of the animals used.

### Step 3: Mimicking the clinical situation

To conclude which of the existing CIPN models are most suitable for studying CIPN, not only efficacy and robustness should be taken into account, but the models should also mimic the clinical situation as close as possible. In a subsequent analysis we therefore excluded all models that used administration routes that were not used or contraindicated in clinical practice according to the British Columbia Cancer guidelines.

Nineteen types of administration routes, in total 166 comparisons (16% of the total number of comparisons initially included) were excluded. Bortezomib I.P. (n = 28), Cisplatin S.C. (n = 5), Docetaxel I.P. (n = 1), Gemicitabine I.P. (n = 2) Ormaplatin I.P. (n = 1), Oxaliplatin I.D. (n = 4 and S.C. (n = 5), Paclitaxel S.C. (n = 3), Sorafenib I.V. (n = 1), Suramin I.P. (n = 2), Tamoxifen I.P. (n = 1), Thalidomide I.P. (n = 3) and I.V. (n = 1), Vincristine I.M. (n = 1) and I.P. (n = 104) and S.C. (n = 1) and intraventricular (n = 2), Vindesine S.C. (n = 1).

Re-analyses of step1 (efficacy) of all CIPN models using an administration route of chemotherapeutics that is currently used in clinical practice resulted in 4 of the same models as in the previous analyses (Table 3). Instead of the “Rats Wistar, female, receiving cisplatin” one new model popped up “mice C57BL6, male, receiving cisplatin”.

**Table 3:** Overview of the efficacy and number of comparisons per model of CIPN models ranked animal species after excluding all models that used administration routes that are not used in clinical practice.

	Species	Strain	Chemotherapy	Sex	n	CIPN		
Species, strain, chemotherapy, sex	Mice	C57BL/6	Cisplatin	Male	13	12 (92%)	4	
	Mice	C57BL/6	Paclitaxel	Female	8	8 (100%)	4	
	Mice	CD1	Paclitaxel	Male	6	6 (100%)	4	
	Rats	SD	Oxaliplatin	Male	62	57 (92%)	3	
	Rats	Wistar	Cisplatin	Female	19	18 (95%)	1	
Species, sex, chemotherapy	Mice		Cisplatin	Male	18	17 (94%)	4	
	Mice		Paclitaxel	Male	33	30 (91%)	4	
	Mice		Paclitaxel	Female	21	19 (90%)	4	
	Mice		Paclitaxel	Both	5	5 (100%)	2	
Species, strain, chemotherapy	Mice	C57BL/6	Cisplatin		16	15 (94%)	4	
	Mice	CD1	Paclitaxel		10	10 (100%)	4	
	Mice	C57BL/6	Paclitaxel		30	28 (93%)	4	
	Rats	SD	Oxaliplatin		63	58 (92%)	3	
Species, chemotherapy	Mice		Cisplatin		28	26 (93%)	4	
	Mice		Paclitaxel		64	58 (91%)	4	2
	Rats		Oxaliplatin		86	77 (90%)	3	

n: number of experiments using a specific model. CIPN: number of experiments using a specific model for chemotherapy induced peripheral neuropathy showing efficacy in more than one of the predefined outcomes (e.g. mechanical allodynia, thermal hyper and hypoalgesia, sensory-motor coordination, electrophysiological measurements and/or histological damage to the peripheral nervous system). The colour code in the last column indicates how often a model appeared as an effective model when using different categories to define a CIPN model. Abbreviations: Sprague Dawley (SD)

The robustness analyses (step 2) revealed that three out of the five selected models appear in all four analyses.

1. mice C57BL/6, female, receiving paclitaxel
2. mice CD1, male, receiving paclitaxel
3. mice C57BL/6, male, receiving cisplatin

Two of those models were identified previously as well (1 and 2).

Cisplatin was tested in 8 strains. Cisplatin was tested in C57Bl6 males, females and mixed groups and showed an efficacy of 100%. The remark however should be made that in females, in mixed sex, and unknown sex groups, only one experiment was conducted per group.

Thus, mice CIPN models using administration routes used in clinical practice treated with either cisplatin or paclitaxel show efficacy across various strains and sex of the animals used.

### Sensitivity analyses

In our analyses a model could only be selected as a promising and suitable model in case a model caused significant peripheral neuropathy in at least 90% of the experiments in 2 or more outcomes. In the sensitivity analyses we changed this level to 85% and re-analysed step 1 (efficacy), step 2 (robustness) and step 3 (mimicking the clinical situation) and showed besides the already identified 3 models using the data presented in S1 Table

1. Mice C57BL/6, female, receiving paclitaxel
2. Mice CD1, male, receiving paclitaxel
3. Mice C57BL/6, male, receiving cisplatin one other promising model:
4. Mice C57BL/6, male, receiving paclitaxel

Initially also a second new model appeared; Mice C57BL/6, male, receiving oxaliplatin, however, this model was solely tested in males and it can therefore not be concluded that this model was robust across sex.

### Study quality: Reporting of Blinding, randomization and sample size calculation

In order to obtain a rough overview of the study quality of the 650 included papers we assessed whether the papers reported the presence of any sample size or power calculations or any measures taken to blind the investigators or randomize the allocation of the animals. Out of our 650 included manuscripts 44% reported measures for blinding, 36% reported a measure for randomisation and only 3% of the included papers reported sample size or power calculations. In this score we also included the articles that explicitly mentioned that they did not conduct blinding, randomisation or sample size calculations (S4 Table).

## Discussion

We conducted a comprehensive summary (in the form of a systematic review) and comparison of all animal models currently described in literature for CIPN, that resulted in a clear overview of all effective and robust models for CIPN focusing on stimulus evoked pain-like behaviour and neurophysiological alterations in nerve function with an administration route used in clinical practice.

In this systematic review 650 papers were included, resulting in 183 unique CIPN models (based on species, strain, sex and type of chemotherapy used), and 1,023 independent comparisons. Twelve different species and 27 different types of chemotherapy were used in these models.

Five outcome measures to identify the presence of CIPN were assessed: mechanical allodynia, thermal hyper and hypoalgesia, motor function, histological damage to the peripheral nervous system and changes in electrophysiological measurements such as changes in nerve conduction.

Using our three-step approach (efficacy, robustness and mimicking the clinical situation) we show in this systematic review that all mice CIPN models treated with either paclitaxel or cisplatin using an administration route used in clinical practice seem suitable models. Three specific models using paclitaxel or cisplatin that stand out (based on the finding that thy are reproduced at least five times and significant (peripheral) polyneuropathy in at least 90% of the experiments in two or more outcomes), are

1. C57BL/6 female mice receiving paclitaxel and
2. CD1 male mice receiving paclitaxel and
3. C57BL/6 male mice receiving cisplatin.

Our review also provides a list of models that show high efficacy but have not been reproduced enough times to be included in our analyses but might be promising CIPN models in the future. Last but not least, this review also provides an overview of the efficacy of CIPN models ordered by type of chemotherapy.

A major strength of this paper, in addition to the large evidence base, is that it is the first systematic review comparing the efficacy and relevance of all CIPN models published in PubMed and Embase. Our overview can help scientists to select a suitable CIPN model for their research. Using a suitable model, will probably lead to a higher translational value of preclinical study results with respect to the potential of identifying promising treatments for CIPN. Scientists need to be aware of the various available models, and how they differ in characteristics and efficacy in causing CIPN. Our review fills this gap as the differences between the various CIPN models are analysed and efficacy and internal, external and construct validity issues are taken into account. For example, a suitable model needs to mimic clinical CIPN (e.g. construct validity) as much as possible. By using administration routes, animal characteristics and outcomes that poorly match the clinical situation for example, construct validity can be threatened. We therefore analysed our entire dataset also without all models using administration route that that are not used in the clinical situation and show that in 16% of the comparisons initially included in this review administration routes are used that are not used or are contraindicated in clinical practice. In addition, we included sex of the animals in our robustness analyses because in human patients there is as far as we know, no clear evidence for a difference between males and females in CIPN rate and severity. Further, because all individual CIPN outcomes have their shortcoming [20] we decided to label a study “effective” only in case at least two outcomes related to on stimulus evoked pain-like behaviour and/ or neurophysiological alterations in nerve function are scored significant peripheral neuropathy. In this systematic review we focused on 3 outcomes related to clinical symptoms (motor impairment, thermal hyper and hypoalgesia and mechanical allodynia) and 2 related to clinical pathophysiology (neuronal damage identified by either histological damage in the peripheral nervous system or electrophysiological measurements).

However, it is important to realize that no animal model will represents the full clinical situation perfectly (because of evolution determined species differences and by inevitable dissimilarities between the conditions created in animals and the human disorders being researched [21]) and research findings therefore need to be confirmed in multiple animal models (as comparable results in multiple similar animal models would increase our confidence in the results and applicability for the clinical situation).

Nevertheless, the results presented in this systematic review seem to be largely in line with the clinical situation. We show that mice CIPN models treated with either paclitaxel or cisplatin show high efficacy in causing CIPN (also across sex and various strains used), and this is in concordance with the results from a recent meta-analysis of clinical studies showing high prevalence of CIPN for both oxaliplatin (72%) and cisplatin (42%) as well [13]. Also, the results from a recent multi-country multisite prospective longitudinal observational study shows that especially paclitaxel is a chemotherapeutic causing one of the highest CIPN rates in patients [9].

### Limitations

This review has some important limitations. Firstly, we summarize and compare animal models for CIPN based on outcome measures related to allodynia/ hyperalgesia and neurophysiological alterations in nerve function, whereas many patients also report other symptoms such as numbness, tingling and ongoing pain.

Theoretically it would be better to use animal models that replicate all symptoms observed in humans. This remains however until today very challenging. Measures like numbness, tingling and ongoing pain rely on verbal report from the patient, often occur spontaneously, and therefore are very difficult to replicate in animal models. Fortunately, investigation into novel measures of ongoing pain in rodents is an emerging are, but for now, developing animal models of CIPN which replicate all the symptoms that patients report remains very challenging, and we therefore focus in this review on allodynia/ hyperalgesia and neurophysiological alterations in nerve function.

Second, the internal validity of the included studies could not be reliably estimated as many of the essential methodological details of animal studies included in our review were poorly reported. 44% and respectively 35% of the included studies reported any measure for blinding or randomization, and only 3% reported sample size calculation or power analyses validating the group sizes that they used.

As a consequence, we cannot reliably estimate how valid the results of the included studies are. Nevertheless, we included the poorly reported papers in this review because papers that do not report essential details are not necessarily methodologically impaired. However, it is important to emphasize that consistent reporting of essential details regarding experimental design for future animal experiments, as described for example in the ARRIVE guidelines, is urgently needed.

Third, our relative low reproducibility number (n = 5) in combination with high efficacy levels (at least 90% in 2 patient important outcomes) may have led to potentially excluding relevant CIPN models. We therefore created a list containing all models that were excluded from analyses because they did not reach our reproducibility limit (n = 5). In addition, we conducted a sensitivity analyses in which we challenged our efficacy level and showed that in case the efficacy level was reduced to 85% the same three effective models using an administration route used in clinical practice are identified, and one other promising model enters the scene (e.g. mice C57BL/6, male, receiving paclitaxel). This new model provides us with additional evidence that all mice models using paclitaxel using an administration route used in clinical practice seem effective (as only the sex is different in this new model compared to one of our previous identified effective models using an administration route in clinical practice).

Fourthly, our conclusions are based on vote counting (is there any evidence of CIPN, or in other words; comparing the number of studies with a significant effect to the number of studies with a non-significant outcome), and the significance of the effect as calculated by the author. This approach has some limitations as we needed to trust that the authors used appropriate statistical tests and that in vote counting procedures the weight of the individual study (largely based on the sample size) is not taken into account.

We nevertheless believe that pooling our results (conducting a real meta-analysis) is not sensible in this case, as we are not interested in an overall summary effect nor the actual effect size, but only the significance of the individual effects.

Fifthly, the studies included in this review are of course heterogenous on many more characteristics than the four we included to define CIPN models (e.g. chemotherapy, species, strain, sex). The dosage (actual amount, frequency and duration) of chemotherapy used may, for example, have influenced whether a model was effective or not. We decided, however, not to include dose as a component to assess the study for clinical relevance (step 3) because we needed to make too many assumptions for each individual study regarding the representative human patient group, the actual indication why the chemotherapeutics are prescribed, and estimates to back transform human treatment regime to animal treatment regime, leading to unreliable results.

In more detail; for each individual comparison the used animal population needs to be matched to a human representative group (regarding, age, sex, weight etc). Subsequently for each specific hypothetical human patients group, and each specific chemotherapeutic, a minimal dose/ or treatment regime used in clinical practice needs to be determined. To do this, many assumptions regarding the type of cancer and stage of disease need to be made (as various types of cancer and stages of disease are treated with different treatment regimes, e.g. dose, number of therapeutic cycles duration). Last but not least, the resulting theoretical dose/ treatment regime needs to be back transformed to a relevant dose in the animal population using estimates based on even more assumptions. In this paper we therefore did not include dose to ‘value” each individual animal model.

In addition, in our current analyses to determine reliable animal models for CIPN we focus on efficacy, and all models that are selected did as a consequence score positive on our CIPN related outcomes, and therefore the dosage used must have been sufficient.

Sixthly, although we included administration route as a component to assess the study for clinical relevance and excluded in step 3 of our methodology all studies using routes of administration that are currently not recommended to use in clinical practice, there seems to be a mismatch in the balance of administration routes used in our models compared to the clinical situation. Paclitaxel and cisplatin, for example, are in our effective, robust CIPN models only administered intravenously in the minority of papers (23% in C57BL/6 female mice receiving paclitaxel, 8% in C57BL/6 male mice receiving cisplatin, and not at all in CD1 male mice receiving paclitaxel) although in clinical practice these levels seem to be higher.

Another important issue regarding clinical relevance of the included models is that the majority of animal models was cancer free, whereas in the clinical situation most CIPN patient have or experienced previously cancer which may confound the results related to the used animal models

Last but not least there is a possibility that not all the studies investigating the efficacy of CIPN models have been published. Potential CIPN models (varying in chemotherapy, dose, administration route, species, strain, age etc.) that ultimately did not cause polyneuropathy symptoms are probably not all published. This resulted in relatively high levels of efficacy of the models included in this review. To partly overcome this, in our analyses we only selected models as an effective model when they caused significant (peripheral) polyneuropathy in at least 90% of the experiments.

### Conclusions and future directions

In this systematic review we show evidence that mice CIPN models treated with either paclitaxel or cisplatin using an administration route used in clinical practice seem suitable models to study CIPN. Three specific models using paclitaxel or cisplatin that stand out are 1) C57BL/6 female mice receiving paclitaxel and 2) CD1 male mice receiving paclitaxel and 3) C57BL/6 male mice receiving cisplatin.

The results and comparisons between various CIPN models described in this paper can be used by scientists that aim to select a suitable CIPN model for their research. We hypothesize that by using effective and robust animal models that mimic the clinical situation as much as possible, the translation to the clinical situation, with respect to the potential of identifying promising treatments for CIPN in the future, will improve.

We believe that more research is needed in models that were potentially effective, but were not reproduced enough to be included in this review, and that there is potential in studying the differences in efficacy between CIPN models as this may help the research community to unravel the mechanism behind the cause of CIPN

We further recommend that scientists in other research fields as well who are planning to conduct animal experiments start with a transparent comparison of the available animal models. The methodology described in this paper can serve as a guidance document. In addition we recommend that scientists register their trial (e.g. www.preclinicaltrials.eu) in order to decrease the likelihood of publication bias [22] or at least publish their experiment according to the available reporting and methodological quality guidelines [23, 24].

## Materials and methods

This systematic review identifies animal models that investigate the effects of CIPN. The review methodology was specified in advance and documented using SYRCLE’s systematic review protocol for animal intervention studies [25] and put online on the SYRCLE Web site (S2 File).

### Literature search strategy

We performed a systematic, computerized search in Medline through the PubMed interface and EMBASE to identify all the animal studies examining CIPN. The full search strategy (S3 File) was based on the search components “experimental animal” [25], “chemotherapy”, and “polyneuropathy”. Search results from both databases were combined and duplicates were removed. In addition, we checked the reference lists of all included studies and relevant reviews identified by our search for additional eligible references. The search was performed on May 18th, 2016 and updated on December 19th, 2017.

### Study selection

All search results were imported in reference manager software. Abstract were initially screened based on title and obvious irrelevant papers were excluded. Early Review Organising Software (EROS; Institute of Clinical Effectiveness and Health Policy, Buenos Aires, Argentina) was used to randomly allocate all remaining references to two independent reviewers, who screened it for inclusion on the basis of its title and abstract (reviewers: SG, SW, SH, and ME). Studies were included if they met all of the following criteria: 1) the study was an original full paper which presented unique data; 2) the study was performed in animals in vivo; 3) the study examined the effect of chemotherapy; 4) the study reported on the outcome (peripheral) neuropathy (e.g. mechanical allodynia, thermal hyper and hypoalgesia, sensory-motor coordination, electrophysiological measurements and/or histological damage to the peripheral nervous system); 5) the study included an appropriate control group. No language or publication date restrictions were applied. If necessary, publications in languages other than English were translated by a native speaker for that particular language.

In case of doubt, the whole publication was evaluated. Full-text copies of all publications eligible for inclusion were subsequently assessed by two independent reviewers and included when they met our pre-specified inclusion criteria. Disagreement was solved by discussion or by consulting a third investigator (CH).

### Study characteristics and data extraction

We extracted bibliographic details such as author, journal, and year of publication, as well as data on the following study characteristics: animal species, strain, sex, age, and weight; type of chemotherapeutic used, dose, frequency, duration of treatment, and route of administration; type of outcomes assessed (e.g. mechanical allodynia, thermal hyper and hypoalgesia, sensory-motor coordination, electrophysiological measurements and/or histological damage to the peripheral nervous system), method of outcome assessment and timing of the outcome measurement relative to chemotherapy induced polyneuropathy induction.

With regard to outcome data extraction; we extracted for all relevant comparisons the presence of statistical evidence for a significant effect.

### Methodological quality

The methodological quality of all selected studies was evaluated by scoring the reporting of three key characteristics of scientific reporting: reporting of any measure of randomization, reporting of any measure of blinding, and reporting of sample size and/or power calculation. For these three items, a ‘Yes’ score indicates ‘reported’, and a ‘No’ score indicates ‘unreported’.

### Data analyses

Step 1: All animal models were stratified according to the species, strain, sex, and type of chemotherapy used. Efficacy of the animal models was assessed by analysing the number of times the animal models was used in different papers, and the percentage of studies that showed a significant effect of chemotherapy on (peripheral) polyneuropathy on either one or two of our outcomes (e.g. mechanical allodynia, thermal hyper and hypoalgesia, sensory-motor coordination, electrophysiological measurements and/or histological damage to the peripheral nervous system). Based on the results and conclusions of the authors of the original paper it was decided whether the effect of chemotherapy induced (peripheral) polyneuropathy on our selected outcomes was significant.

To be selected as models with promising efficacy, animal models needed to meet the following criteria: 1) reproduced at least five times; 2) causing significant (peripheral) polyneuropathy in at least 90% of the experiments in two or more outcomes. Subsequently (step 2), the resulting list of promising animal models was assessed for robustness. The same analysis as described above was repeated using different categories to define CIPN models. In our original analyses CIPN models were classified based on species, strain, sex, and type of chemotherapy used. In this robustness assessment CIPN models were classified according to either: 1) species and chemotherapy alone; 2) species, strain, and chemotherapy; 3) species, chemotherapy, and sex, which allows us to assess how a model performs across strains and the sex of animals used. In case a model performs well in all assessments it is to be expected that the model is quite independent of the strain and sex of the animals used.

However, it is important to realize that the number of variations in models used within a category and the amount of available evidence per model may influence the results of this robustness assessment, and therefore the results of the robustness assessment will be analysed with respect to the actual number of variations in models within each category (for example, for a models to be considered as robust across sex, the animal model must have been tested in both sexes, and in at least 2 different strains).

The above-mentioned analysis will lead to a list of models with the highest efficacy. However, in order to increase the utility of the list with the most promising animal models the animal model should mimic the clinical situation as closely as possible (step 3). Therefore, in a subsequent analysis we excluded all models that used administration routes that were not used in clinical practice (e.g. intraperitoneal vincristine and intradermal oxaliplatin) and repeated our analysis as previously described. All analyses were compared.

Last but not least a sensitivity analyses was conducted. In our analyses a model could only be selected as a promising and suitable model in case a model caused significant peripheral neuropathy in at least 90% of the experiments in 2 or more outcomes. In the sensitivity analyses we changed this level to 85% and re-analysed step 1 (efficacy), step 2 (robustness) and step 3 (mimicking the clinical situation).

## Supporting information

S1 File References of included manuscripts. (DOCX)

S2 File SR protocol. (DOCX)

S3 File Full search strategy. (DOCX)

S4 File PRISMA 2009 checklist. (DOC)

S1 Table Overview of the efficacy of CIPN models ranked by animal species. * chemotherapy-induced polyneuropathy in one outcome measurement.
^†^ chemotherapy-induced polyneuropathy in more than one outcome measurement.
(DOCX)

S2 Table Overview of the efficacy of CIPNP models ranked by type of chemotherapy used. * chemotherapy-induced polyneuropathy in one outcome measurement.
^†^ chemotherapy-induced polyneuropathy in more than one outcome measurement.
(DOCX)

S3 Table Overview of the efficacy of promising CIPN models. * chemotherapy-induced polyneuropathy in one outcome measurement.
^†^ chemotherapy-induced polyneuropathy in more than one outcome measurement
(DOCX)

S4 Table Quality of reporting. (DOCX)
