# Supplementary material for: A systematic summary and comparison of animal models for chemotherapy induced (peripheral) neuropathy (CIPN)
Source: PLoS One. 2019 Aug 28;14(8):e0221787. doi: 10.1371/journal.pone.0221787 (PMC6713358; doi:10.1371/journal.pone.0221787)
Supplement: S1 File — (DOCX) [file pone.0221787.s001.docx]

# S1 File: References of included manuscripts

1. Abad ANA, Nouri MHK, Gharjanie A, Tavakoli F. Effect of Matricaria chamomilla Hydroalcoholic Extract on Cisplatin-induced Neuropathy in Mice. Chinese Journal of Natural Medicines. 2011:126-31.

2. Abed A, Khoshnoud MJ, Taghian M, Aliasgharzadeh M, Mesdaghinia A. Quetiapine reverses paclitaxel-induced neuropathic pain in mice: role of alpha2- adrenergic receptors. Iranian Journal of Basic Medical Sciences. 2017(11):1182-8.

3. Abram M, Zagaja M, Mogilski S, Andres-Mach M, Latacz G, Bas S, et al. Multifunctional Hybrid Compounds Derived from 2-(2,5-Dioxopyrrolidin-1-yl)-3-methoxypropanamides with Anticonvulsant and Antinociceptive Properties. J Med Chem. 2017(20):8565-79.

4. Adamek P, Tatsui CE, Rhines LD, Mrozkova P, Li Q, Kosturakis AK, et al. The cancer chemotherapeutic paclitaxel increases human and rodent sensory neuron responses to TRPV1 by activation of TLR4. Journal of Neuroscience. 2015:13487-500.

5. Ahmad N, Subhan F, Islam NU, Shahid M, Rahman FU, Sewell RDE. Gabapentin and its salicylaldehyde derivative alleviate allodynia and hypoalgesia in a cisplatin-induced neuropathic pain model. Eur J Pharmacol. 2017:302-12.

6. Ahn SH, Chang IA, Kim KJ, Kim CJ, Namgung U, Cho CS. Bogijetong decoction and its active herbal components protect the peripheral nerve from damage caused by taxol or nerve crush. BMC Complementary and Alternative Medicine. 2016(402).

7. Akman T, Akman L, Erbas O, Terek MC, Taskiran D, Ozsaran A. The preventive effect of oxytocin to Cisplatin-induced neurotoxicity: an experimental rat model. Biomed Res Int. 2015:167235.

8. Al Moundhri MS, Al-Salam S, Al Mahrouqee A, Beegam S, Ali BH. The effect of curcumin on oxaliplatin and cisplatin neurotoxicity in rats: some behavioral, biochemical, and histopathological studies. J Med Toxicol. 2013:25-33.

9. Alaedini A, Xiang Z, Kim H, Sung YJ, Latov N. Up-regulation of apoptosis and regeneration genes in the dorsal root ganglia during cisplatin treatment. Experimental Neurology. 2008:368-74.

10. Ale A, Bruna J, Calls A, Karamita M, Haralambous S, Probert L, et al. Inhibition of the neuronal NFkappaB pathway attenuates bortezomib-induced neuropathy in a mouse model. Neurotoxicology. 2016:58-64.

11. Ale A, Bruna J, Morell M, van de Velde H, Monbaliu J, Navarro X, et al. Treatment with anti-TNF alpha protects against the neuropathy induced by the proteasome inhibitor bortezomib in a mouse model. Exp Neurol. 2014:165-73.

12. Alessandri-Haber N, Dina OA, Joseph EK, Reichling DB, Levine JD. Interaction of transient receptor potential vanilloid 4, integrin, and Src tyrosine kinase in mechanical hyperalgesia. Journal of Neuroscience. 2008:1046-57.

13. Alessandri-Haber N, Dina OA, Yeh JJ, Parada CA, Reichling DB, Levine JD. Transient receptor potential vanilloid 4 is essential in chemotherapy-induced neuropathic pain in the rat. J Neurosci. 2004:4444-52.

14. Alexa T, Luca A, Bohotin C, Lupchian D, Stanciuc I, Badescu M. The Effect of Cobalt Chloride Preconditioning on Paclitaxel-Induced Peripheral Neuropathy. Revista medico-chirurgicala a Societatii de Medici si Naturalisti din Iasi. 2015:447-53.

15. Aley KO, Levine JD. Different peripheral mechanisms mediate enhanced nociception in metabolic/toxic and traumatic painful peripheral neuropathies in the rat. Neuroscience. 2002:389-97.

16. Aley KO, Reichling DB, Levine JD. Vincristine hyperalgesia in the rat: A model of painful vincristine neuropathy in humans. Neuroscience. 1996:259-65.

17. Alimoradi H, Pourmohammadi N, Mehr SE, Hassanzadeh G, Hadian MR, Sharifzadeh M, et al. Effects of lithium on peripheral neuropathy induced by vincristine in rats. Acta Medica Iranica. 2012:373-9.

18. Aloe L, Manni L, Properzi F, De Santis S, Fiore M. Evidence that nerve growth factor promotes the recovery of peripheral neuropathy induced in mice by cisplatin: behavioral, structural and biochemical analysis. Auton Neurosci. 2000:84-93.

19. Alvarez P, Ferrari LF, Levine JD. Muscle pain in models of chemotherapy-induced and alcohol-induced peripheral neuropathy. Ann Neurol. 2011:101-9.

20. Ameyaw EO, Boampong JN, Kukuia KE, Amoateng P, Obese E, Osei-Sarpong C, et al. Effect of xylopic acid on paclitaxel-induced neuropathic pain in rats. Journal of Medical and Biomedical Sciences. 2013:6-12.

21. Ameyaw EO, Woode E, Boakye-Gyasi E, Abotsi WK, Kyekyeku JO, Adosraku RK. Anti-allodynic and Anti-hyperalgesic effects of an ethanolic extract and xylopic acid from the fruits of Xylopia aethiopica in murine models of neuropathic pain. Pharmacognosy Res. 2014:172-9.

22. Ami N, Okamoto K, Oshima H. Analgesic effect of magnetic stimulation on paclitaxel-induced peripheral neuropathic pain in mice. Brain Res. 2012:24-9.

23. Amoateng P, Adjei S, Osei-Safo D, Ameyaw EO, Ahedor B, Guessan B B, et al. A hydro-ethanolic extract of Synedrella nodiflora (L.) Gaertn ameliorates hyperalgesia and allodynia in vincristine-induced neuropathic pain in rats. J Basic Clin Physiol Pharmacol. 2015:383-94.

24. Amoateng P, Adjei S, Osei-Safo D, Kukuia KKE, Kretchy IA, Sarkodie JA, et al. Analgesic effects of a hydro-ethanolic whole plant extract of Synedrella nodiflora (L.) Gaertn in paclitaxel-induced neuropathic pain in rats. BMC Res Notes. 2017(1):226.

25. Andoh T, Kitamura R, Kuraishi Y. Milnacipran inhibits oxaliplatin-induced mechanical allodynia through spinal action in mice. Biological and Pharmaceutical Bulletin. 2015:151-4.

26. Andoh T, Kobayashi N, Uta D, Kuraishi Y. Prophylactic topical paeoniflorin prevents mechanical allodynia caused by paclitaxel in mice through adenosine A1 receptors. Phytomedicine. 2017:1-7.

27. Andoh T, Sakamoto A, Kuraishi Y. Effects of xaliproden, a 5-HT1A agonist, on mechanical allodynia caused by chemotherapeutic agents in mice. European Journal of Pharmacology. 2013:231-6.

28. Andoh T, Uta D, Kato M, Toume K, Komatsu K, Kuraishi Y. Prophylactic administration of aucubin inhibits paclitaxel-induced mechanical allodynia via the inhibition of endoplasmic reticulum stress in peripheral Schwann cells. Biological and Pharmaceutical Bulletin. 2017(4):473-8.

29. Aoki M, Kurauchi Y, Mori A, Nakahara T, Sakamoto K, Ishii K. Comparison of the effects of single doses of elcatonin and pregabalin on oxaliplatin-induced cold and mechanical allodynia in rats. Biol Pharm Bull. 2014:322-6.

30. Aoki M, Mori A, Nakahara T, Sakamoto K, Ishii K. Effect of synthetic eel calcitonin, elcatonin, on cold and mechanical allodynia induced by oxaliplatin and paclitaxel in rats. Eur J Pharmacol. 2012:62-9.

31. Aoki M, Mori A, Nakahara T, Sakamoto K, Ishii K. Salmon calcitonin reduces oxaliplatin-induced cold and mechanical allodynia in rats. Biol Pharm Bull. 2013:326-9.

32. Aouad M, Charlet A, Rodeau JL, Poisbeau P. Reduction and prevention of vincristine-induced neuropathic pain symptoms by the non-benzodiazepine anxiolytic etifoxine are mediated by 3alpha-reduced neurosteroids. Pain. 2009:54-9.

33. Apfel SC, Arezzo JC, Lewis ME, Kessler JA. The use of insulin-like growth factor I in the prevention of vincristine neuropathy in mice. Annals of the New York Academy of Sciences. 1993:243-5.

34. Apfel SC, Arezzo JC, Lipson L, Kessler JA. Nerve growth factor prevents experimental cisplatin neuropathy. Annals of Neurology. 1992:76-80.

35. Apfel SC, Lipton RB, Arezzo JC, Kessler JA. Nerve growth factor prevents toxic neuropathy in mice. Ann Neurol. 1991:87-90.

36. Areti A, Komirishetty P, Akuthota M, Malik RA, Kumar A. Melatonin prevents mitochondrial dysfunction and promotes neuroprotection by inducing autophagy during oxaliplatin-evoked peripheral neuropathy. Journal of Pineal Research. 2017(e12393).

37. Areti A, Komirishetty P, Kumar A. Carvedilol prevents functional deficits in peripheral nerve mitochondria of rats with oxaliplatin-evoked painful peripheral neuropathy. Toxicol Appl Pharmacol. 2017:97-103.

38. Arrieta O, Hernandez-Pedro N, Fernandez-Gonzalez-Aragon MC, Saavedra-Perez D, Campos-Parra AD, Rios-Trejo MA, et al. Retinoic acid reduces chemotherapy-induced neuropathy in an animal model and patients with lung cancer. Neurology. 2011:987-95.

39. Authier N, Fialip J, Eschalier A, Coudore F. Assessment of allodynia and hyperalgesia after cisplatin administration to rats. Neuroscience Letters. 2000:73-6.

40. Authier N, Gillet JP, Fialip J, Eschalier A, Coudore F. Description of a short-term Taxol-induced nociceptive neuropathy in rats. Brain Res. 2000:239-49.

41. Authier N, Gillet JP, Fialip J, Eschalier A, Coudore F. A new animal model of vincristine-induced nociceptive peripheral neuropathy. Neurotoxicology. 2003:797-805.

42. Authier N, Gillet JP, Fialip J, Eschalier A, Coudore F. An animal model of nociceptive peripheral neuropathy following repeated cisplatin injections. Exp Neurol. 2003:12-20.

43. Azevedo MI, Pereira AF, Nogueira RB, Rolim FE, Brito GAC, Wong DVT, et al. The antioxidant effects of the flavonoids rutin and quercetin inhibit oxaliplatin-induced chronic painful peripheral neuropathy. Molecular Pain. 2013.

44. Babu A, Prasanth KG, Balaji B. Effect of curcumin in mice model of vincristine-induced neuropathy. Pharm Biol. 2015:838-48.

45. Bahar MA, Andoh T, Ogura K, Hayakawa Y, Saiki I, Kuraishi Y. Herbal medicine goshajinkigan prevents paclitaxel-induced mechanical allodynia without impairing antitumor activity of paclitaxel. Evidence-based Complementary and Alternative Medicine. 2013.

46. Balayssac D, Cayre A, Authier N, Bourdu S, Penault-Llorca F, Gillet JP, et al. Patterns of P-glycoprotein activity in the nervous system during vincristine-induced neuropathy in rats. J Peripher Nerv Syst. 2005:301-10.

47. Balayssac D, Cayre A, Authier N, Ling B, Maublant J, Eschalier A, et al. Involvement of the multidrug resistance transporters in cisplatin-induced neuropathy in rats. Comparison with the chronic constriction injury model and monoarthritic rats. Eur J Pharmacol. 2006:49-57.

48. Balayssac D, Cayre A, Ling B, Maublant J, Penault-Llorca F, Eschalier A, et al. Vincristine-induced neuropathy in the rat is not modified by drug-drug interactions with the P-glycoprotein inhibitor verapamil. Chemotherapy. 2008:336-42.

49. Balayssac D, Cayre A, Ling B, Maublant J, Penault-Llorca F, Eschalier A, et al. Increase in morphine antinociceptive activity by a P-glycoprotein inhibitor in cisplatin-induced neuropathy. Neuroscience Letters. 2009:108-12.

50. Balayssac D, Ling B, Ferrier J, Pereira B, Eschalier A, Authier N. Assessment of thermal sensitivity in rats using the thermal place preference test: Description and application in the study of oxaliplatin-induced acute thermal hypersensitivity and inflammatory pain models. Behavioural Pharmacology. 2014:99-111.

51. Bang S, Kim YS, Jeong SR. Anti-allodynic effect of theoesberiven F in a vincristine-induced neuropathy model. Experimental and Therapeutic Medicine. 2016(2):799-803.

52. Baptista-de-Souza D, Di Cesare Mannelli L, Zanardelli M, Micheli L, Nunes-de-Souza RL, Canto-de-Souza A, et al. Serotonergic modulation in neuropathy induced by oxaliplatin: effect on the 5HT2C receptor. Eur J Pharmacol. 2014:141-9.

53. Barajon I, Bersani M, Quartu M, Del Fiacco M, Cavaletti G, Holst JJ, et al. Neuropeptides and morphological changes in cisplatin-induced dorsal root ganglion neuronopathy. Exp Neurol. 1996:93-104.

54. Bardos G, Moricz K, Jaszlits L, Rabloczky G, Tory K, Racz I, et al. BGP-15, a hydroximic acid derivative, protects against cisplatin- or taxol-induced peripheral neuropathy in rats. Toxicology and Applied Pharmacology. 2003:9-16.

55. Barzegar-Fallah A, Alimoradi H, Mehrzadi S, Barzegar-Fallah N, Zendedel A, Abbasi A, et al. The neuroprotective effect of tropisetron on vincristine-induced neurotoxicity. Neurotoxicology. 2014:1-8.

56. Benbow SJ, Cook BM, Reifert J, Wozniak KM, Slusher BS, Littlefield BA, et al. Effects of Paclitaxel and Eribulin in Mouse Sciatic Nerve: A Microtubule-Based Rationale for the Differential Induction of Chemotherapy-Induced Peripheral Neuropathy. Neurotoxicity Research. 2016:299-313.

57. Bennett GJ, Liu GK, Xiao WH, Jin HW, Siau C. Terminal arbor degeneration--a novel lesion produced by the antineoplastic agent paclitaxel. Eur J Neurosci. 2011:1667-76.

58. Bhadri N, Sanji T, Madakasira Guggilla H, Razdan R. Amelioration of behavioural, biochemical, and neurophysiological deficits by combination of monosodium glutamate with resveratrol/alpha-lipoic acid/coenzyme Q10 in rat model of cisplatin-induced peripheral neuropathy. ScientificWorldJournal. 2013:565813.

59. Bhalla S, Singh N, Jaggi AS. Dose-related neuropathic and anti-neuropathic effects of simvastatin in vincristine-induced neuropathic pain in rats. Food Chem Toxicol. 2015:32-40.

60. Bhattacharya MR, Gerdts J, Naylor SA, Royse EX, Ebstein SY, Sasaki Y, et al. A model of toxic neuropathy in Drosophila reveals a role for MORN4 in promoting axonal degeneration. J Neurosci. 2012:5054-61.

61. Bianchi R, Brines M, Lauria G, Savino C, Gilardini A, Nicolini G, et al. Protective effect of erythropoietin and its carbamylated derivative in experimental Cisplatin peripheral neurotoxicity. Clin Cancer Res. 2006:2607-12.

62. Bianchi R, Gilardini A, Rodriguez-Menendez V, Oggioni N, Canta A, Colombo T, et al. Cisplatin-induced peripheral neuropathy: neuroprotection by erythropoietin without affecting tumour growth. Eur J Cancer. 2007:710-7.

63. Bianco MR, Cirillo G, Petrosino V, Marcello L, Soleti A, Merizzi G, et al. Neuropathic pain and reactive gliosis are reversed by dialdehydic compound in neuropathic pain rat models. Neurosci Lett. 2012:85-90.

64. Boegman RJ, Scarth B, Dragovic L, Robertson DM. Neurotoxicity of Adriamycin and misonidazole in the mouse. Exp Neurol. 1985:1-8.

65. Boehmerle W, Huehnchen P, Peruzzaro S, Balkaya M, Endres M. Electrophysiological, behavioral and histological characterization of paclitaxel, cisplatin, vincristine and bortezomib-induced neuropathy in C57Bl/6 mice. Sci Rep. 2014:6370.

66. Boehmerle W, Muenzfeld H, Springer A, Huehnchen P, Endres M. Specific targeting of neurotoxic side effects and pharmacological profile of the novel cancer stem cell drug salinomycin in mice. J Mol Med (Berl). 2014:889-900.

67. Boiko N, Medrano G, Montano E, Jiang N, Williams CR, Madungwe NB, et al. TrpA1 activation in peripheral sensory neurons underlies the ionic basis of pain hypersensitivity in response to vinca alkaloids. PLoS One. 2017;12(10):e0186888.

68. Bond MR, Wolman L. EXPERIMENTAL AND CLINICAL OBSERVATIONS CONCERNING THE NEUROTOXICITY OF ETHOGLUCID. Br Med J. 1965:1161-3.

69. Borzan J, LaGraize SC, Fuchs PN. Effect of chronic vincristine treatment on mechanical withdrawal response and pre-pulse inhibition in the rat. Neuroscience Letters. 2004:110-3.

70. Boyette-Davis J, Dougherty PM. Protection against oxaliplatin-induced mechanical hyperalgesia and intraepidermal nerve fiber loss by minocycline. Exp Neurol. 2011:353-7.

71. Boyette-Davis JA, Fuchs PN. Differential effects of paclitaxel treatment on cognitive functioning and mechanical sensitivity. Neuroscience Letters. 2009:170-4.

72. Boyle FM, Beatson C, Monk R, Grant SL, Kurek JB. The experimental neuroprotectant leukaemia inhibitory factor (LIF) does not compromise antitumour activity of paclitaxel, cisplatin and carboplatin. Cancer Chemotherapy and Pharmacology. 2001:429-34.

73. Boyle FM, Wheeler HR, Shenfield GM. Glutamate ameliorates experimental vincristine neuropathy. J Pharmacol Exp Ther. 1996:410-5.

74. Boyle FM, Wheeler HR, Shenfield GM. Amelioration of experimental cisplatin and paclitaxel neuropathy with glutamate. Journal of Neuro-Oncology. 1999:107-16.

75. Bradley WG. The neuromyopathy of vincristine in the guinea pig. An electrophysiological and pathological study. Journal of the neurological sciences. 1970:133-62.

76. Bradley WG, Williams MH. Axoplasmic flow in axonal neuropathies. I. Axoplasmic flow in cats with toxic neuropathies. Brain. 1973:235-46.

77. Brandolini L, Benedetti E, Ruffini PA, Russo R, Cristiano L, Antonosante A, et al. CXCR1/2 pathways in paclitaxel-induced neuropathic pain. Oncotarget. 2017(14):23188-201.

78. Braz JM, Wang X, Guan Z, Rubenstein JL, Basbaum AI. Transplant-mediated enhancement of spinal cord GABAergic inhibition reverses paclitaxel-induced mechanical and heat hypersensitivity. Pain. 2015:1084-91.

79. Brederson JD, Joshi SK, Browman KE, Mikusa J, Zhong C, Gauvin D, et al. PARP inhibitors attenuate chemotherapy-induced painful neuropathy. J Peripher Nerv Syst. 2012:324-30.

80. Bregman CL, Buroker RA, Hirth RS, Crosswell AR, Durham SK. Etoposide- and BMY-40481-induced sensory neuropathy in mice. Toxicologic Pathology. 1994:528-35.

81. Bremer M, Frob F, Kichko T, Reeh P, Tamm ER, Suter U, et al. Sox10 is required for Schwann-cell homeostasis and myelin maintenance in the adult peripheral nerve. Glia. 2011:1022-32.

82. Brito AMS, Godin AM, Augusto PSA, Menezes RR, Melo ISF, Dutra M, et al. Antiallodynic activity of leflunomide is partially inhibited by naltrexone and glibenclamide and associated with reduced production of TNF-alpha and CXCL-1. Eur J Pharmacol. 2017:17-25.

83. Bruna J, Ale A, Velasco R, Jaramillo J, Navarro X, Udina E. Evaluation of pre-existing neuropathy and bortezomib retreatment as risk factors to develop severe neuropathy in a mouse model. Journal of the Peripheral Nervous System. 2011:199-212.

84. Brusco I, Camponogara C, Carvalho FB, Schetinger MRC, Oliveira MS, Trevisan G, et al. alpha-Spinasterol: a COX inhibitor and a transient receptor potential vanilloid 1 antagonist presents an antinociceptive effect in clinically relevant models of pain in mice. Br J Pharmacol. 2017(23):4247-62.

85. Brusco I, Silva CR, Trevisan G, de Campos Velho Gewehr C, Rigo FK, La Rocca Tamiozzo L, et al. Potentiation of Paclitaxel-Induced Pain Syndrome in Mice by Angiotensin I Converting Enzyme Inhibition and Involvement of Kinins. Mol Neurobiol. 2017(10):7824-37.

86. Bujalska M, Arazna M, Makulska-Nowak H, Gumulka SW. Alpha1- and alpha2-Adrenoreceptor antagonists

in streptozotocin- and vincristine-induced hyperalgesia. Pharmacological Reports. 2008:499-507.

87. Bujalska M, Gumulka SW. Effect of cyclooxygenase and nitric oxide synthase inhibitors on vincristine induced hyperalgesia in rats. Pharmacological Reports. 2008:735-41.

88. Bujalska M, Makulska-Nowak H. Bradykinin receptor antagonists and cyclooxygenase inhibitors in vincristine- and streptozotocin-induced hyperalgesia. Pharmacol Rep. 2009:631-40.

89. Bujalska M, Makulska-Nowak H, Gumulka SW. Magnesium ions and opioid agonists in vincristine-induced neuropathy. Pharmacol Rep. 2009:1096-104.

90. Bullinger KL, Nardelli P, Wang Q, Rich MM, Cope TC. Oxaliplatin neurotoxicity of sensory transduction in rat proprioceptors. J Neurophysiol. 2011:704-9.

91. Burgos E, Gomez-Nicola D, Pascual D, Martin MI, Nieto-Sampedro M, Goicoechea C. Cannabinoid agonist WIN 55,212-2 prevents the development of paclitaxel-induced peripheral neuropathy in rats. Possible involvement of spinal glial cells. Eur J Pharmacol. 2012:62-72.

92. Callizot N, Andriambeloson E, Glass J, Revel M, Ferro P, Cirillo R, et al. Interleukin-6 protects against paclitaxel, cisplatin and vincristine-induced neuropathies without impairing chemotherapeutic activity. Cancer Chemotherapy and Pharmacology. 2008:995-1007.

93. Canta A, Chiorazzi A, Carozzi V, Meregalli C, Oggioni N, Sala B, et al. In vivo comparative study of the cytotoxicity of a liposomal formulation of cisplatin (lipoplatin). Cancer Chemother Pharmacol. 2011:1001-8.

94. Carozzi V, Chiorazzi A, Canta A, Oggioni N, Gilardini A, Rodriguez-Menendez V, et al. Effect of the chronic combined administration of cisplatin and paclitaxel in a rat model of peripheral neurotoxicity. Eur J Cancer. 2009:656-65.

95. Carozzi VA, Canta A, Oggioni N, Sala B, Chiorazzi A, Meregalli C, et al. Neurophysiological and neuropathological characterization of new murine models of chemotherapy-induced chronic peripheral neuropathies. Exp Neurol. 2010:301-9.

96. Carozzi VA, Chiorazzi A, Canta A, Lapidus RG, Slusher BS, Wozniak KM, et al. Glutamate carboxypeptidase inhibition reduces the severity of chemotherapy-induced peripheral neurotoxicity in rat. Neurotox Res. 2010:380-91.

97. Carozzi VA, Chiorazzi A, Canta A, Meregalli C, Oggioni N, Cavaletti G, et al. Chemotherapy-induced peripheral neurotoxicity in immune-deficient mice: new useful ready-to-use animal models. Exp Neurol. 2015:92-102.

98. Carozzi VA, Renn CL, Bardini M, Fazio G, Chiorazzi A, Meregalli C, et al. Bortezomib-induced painful peripheral neuropathy: an electrophysiological, behavioral, morphological and mechanistic study in the mouse. PLoS One. 2013:e72995.

99. Carta F, Di Cesare Mannelli L, Pinard M, Ghelardini C, Scozzafava A, McKenna R, et al. A class of sulfonamide carbonic anhydrase inhibitors with neuropathic pain modulating effects. Bioorg Med Chem. 2015:1828-40.

100. Cata JP, Weng HR, Dougherty PM. Cyclooxygenase inhibitors and thalidomide ameliorate vincristine-induced hyperalgesia in rats. Cancer Chemotherapy and Pharmacology. 2004:391-7.

101. Cata JP, Weng HR, Dougherty PM. Behavioral and electrophysiological studies in rats with cisplatin-induced chemoneuropathy. Brain Research. 2008:91-8.

102. Cata JP, Weng HR, Dougherty PM. The effects of thalidomide and minocycline on taxol-induced hyperalgesia in rats. Brain Research. 2008:100-10.

103. Cavaletti G, Fabbrica D, Minoia C, Frattola L, Tredici G. Carboplatin toxic effects on the peripheral nervous system of the rat. Ann Oncol. 1998:443-7.

104. Cavaletti G, Gilardini A, Canta A, Rigamonti L, Rodriguez-Menendez V, Ceresa C, et al. Bortezomib-induced peripheral neurotoxicity: a neurophysiological and pathological study in the rat. Exp Neurol. 2007:317-25.

105. Cavaletti G, Pezzoni G, Pisano C, Oggioni N, Sala F, Zoia C, et al. Cisplatin-induced peripheral neurotoxicity in rats reduces the circulating levels of nerve growth factor. Neurosci Lett. 2002:103-6.

106. Cavaletti G, Tredici G, Braga M, Tazzari S. Experimental peripheral neuropathy induced in adult rats by repeated intraperitoneal administration of taxol. Exp Neurol. 1995:64-72.

107. Cavaletti G, Tredici G, Marmiroli P, Petruccioli MG, Barajon I, Fabbrica D. Morphometric study of the sensory neuron and peripheral nerve changes induced by chronic cisplatin (DDP) administration in rats. Acta Neuropathologica. 1992:364-71.

108. Cavaletti G, Tredici G, Petruccioli MG, Donde E, Tredici P, Marmiroli P, et al. Effects of different schedules of oxaliplatin treatment on the peripheral nervous system of the rat. Eur J Cancer. 2001:2457-63.

109. Cece R, Petruccioli MG, Cavaletti G, Barajon I, Tredici G. An ultrastructural study of neuronal changes in dorsal root ganglia (DRG) of rats after chronic cisplatin administrations. Histol Histopathol. 1995:837-45.

110. Celebi N, Cil H, Cil O, Canbay O, Onur R, Aypar U. Protective effect of coenzyme Q10 in paclitaxel-induced peripheral neuropathy in rats. Neurosciences (Riyadh). 2013:133-7.

111. Cerles O, Benoit E, Chereau C, Chouzenoux S, Morin F, Guillaumot MA, et al. Niclosamide inhibits oxaliplatin neurotoxicity while improving colorectal cancer therapeutic response. Molecular Cancer Therapeutics. 2017(2):300-11.

112. Cervellini I, Bello E, Frapolli R, Porretta-Serapiglia C, Oggioni N, Canta A, et al. The neuroprotective effect of erythropoietin in docetaxel-induced peripheral neuropathy causes no reduction of antitumor activity in 13762 adenocarcinoma-bearing rats. Neurotox Res. 2010:151-60.

113. Cetinkaya-Fisgin A, Joo MG, Ping X, Thakor NV, Ozturk C, Hoke A, et al. Identification of fluocinolone acetonide to prevent paclitaxel-induced peripheral neuropathy. Journal of the Peripheral Nervous System. 2016(3):128-33.

114. Chaumette T, Chapuy E, Berrocoso E, Llorca-Torralba M, Bravo L, Mico JA, et al. Effects of S 38093, an antagonist/inverse agonist of histamine H3 receptors, in models of neuropathic pain in rats. Eur J Pain. 2018(1):127-41.

115. Chelini A, Brogi S, Paolino M, Di Capua A, Cappelli A, Giorgi G, et al. Synthesis and Biological Evaluation of Novel Neuroprotective Pyridazine Derivatives as Excitatory Amino Acid Transporter 2 (EAAT2) Activators. J Med Chem. 2017(12):5216-21.

116. Chen H, Wang Q, Shi D, Yao D, Zhang L, Xiong J, et al. Celecoxib alleviates oxaliplatin-induced hyperalgesia through inhibition of spinal ERK1/2 signaling. Journal of Toxicologic Pathology. 2016(4):253-9.

117. Chen K, Zhang ZF, Liao MF, Yao WL, Wang J, Wang XR. Blocking PAR2 attenuates oxaliplatin-induced neuropathic pain via TRPV1 and releases of substance P and CGRP in superficial dorsal horn of spinal cord. J Neurol Sci. 2015:62-7.

118. Chen LH, Sun YT, Chen YF, Lee MY, Chang LY, Chang JY, et al. Integrating Image-Based High-Content Screening with Mouse Models Identifies 5-Hydroxydecanoate as a Neuroprotective Drug for Paclitaxel-Induced Neuropathy. Mol Cancer Ther. 2015(10):2206-14.

119. Chen SR, Zhu L, Chen H, Wen L, Laumet G, Pan HL. Increased spinal cord Na(+)-K(+)-2Cl(-) cotransporter-1 (NKCC1) activity contributes to impairment of synaptic inhibition in paclitaxel-induced neuropathic pain. J Biol Chem. 2014:31111-20.

120. Chen X, Green PG, Levine JD. Abnormal muscle afferent function in a model of taxol chemotherapy-induced painful neuropathy. Journal of Neurophysiology. 2011:274-9.

121. Chen Y, Yang C, Wang ZJ. Proteinase-activated receptor 2 sensitizes transient receptor potential vanilloid 1, transient receptor potential vanilloid 4, and transient receptor potential ankyrin 1 in paclitaxel-induced neuropathic pain. Neuroscience. 2011:440-51.

122. Chen Z, Janes K, Chen C, Doyle T, Bryant L, Tosh DK, et al. Controlling murine and rat chronic pain through A3 adenosine receptor activation. Faseb j. 2012:1855-65.

123. Cheng X, Huo J, Wang D, Cai X, Sun X, Lu W, et al. Herbal Medicine AC591 Prevents Oxaliplatin-Induced Peripheral Neuropathy in Animal Model and Cancer Patients. Front Pharmacol. 2017:344.

124. Chentanez V, Sanguanrungsirigul S, Panyasawad N. Effects of ganglioside on paclitaxel (Taxol) induced neuropathy in rats. Journal of the Medical Association of Thailand. 2003:449-56.

125. Chentanez V, Thanomsridejchai N, Duangmardphon N, Agthong S, Kaewsema A, Huanmanop T, et al. Ganglioside GM1 (porcine) ameliorates paclitaxel-induced neuropathy in rats. Journal of the Medical Association of Thailand. 2009:50-7.

126. Chiba T, Oka Y, Kambe T, Koizumi N, Abe K, Kawakami K, et al. Paclitaxel-induced peripheral neuropathy increases substance P release in rat spinal cord. Eur J Pharmacol. 2016:46-51.

127. Chiba T, Oka Y, Sashida H, Kanbe T, Abe K, Utsunomiya I, et al. Vincristine-induced peripheral neuropathic pain and expression of transient receptor potential vanilloid 1 in rat. J Pharmacol Sci. 2017(4):254-60.

128. Cho ES, Lowndes HE, Goldstein BD. Neurotoxicology of vincristine in the cat. Morphological study. Archives of Toxicology. 1983:83-90.

129. Cho ES, Spencer PS, Jortner BS, Schaumburg HH. A single intravenous injection of doxorubicin (Adriamycin) induces sensory neuronopathy in rats. NeuroToxicology. 1980:583-91.

130. Cho ES, Yi JM, Park JS, Lee YJ, Lim CJ, Bang OS, et al. Aqueous extract of Lithospermi radix attenuates oxaliplatin-induced neurotoxicity in both in vitro and in vivo models. BMC Complement Altern Med. 2016(1):419.

131. Chogtu B, Bairy KL, Smitha D, Dhar S, Himabindu P. Comparison of the efficacy of carbamazepine, gabapentin and lamotrigine for neuropathic pain in rats. Indian J Pharmacol. 2011:596-8.

132. Choi J, Jeon C, Lee JH, Jang JU, Quan FS, Lee K, et al. Suppressive Effects of Bee Venom Acupuncture on Paclitaxel-Induced Neuropathic Pain in Rats: Mediation by Spinal alpha(2)-Adrenergic Receptor. Toxins (Basel). 2017(11).

133. Choi JW, Kang SY, Choi JG, Kang DW, Kim SJ, Lee SD, et al. Analgesic effect of electroacupuncture on paclitaxel-induced neuropathic pain via spinal opioidergic and adrenergic mechanisms in mice. Am J Chin Med. 2015:57-70.

134. Choi S, Yamada A, Kim W, Kim SK, Furue H. Noradrenergic inhibition of spinal hyperexcitation elicited by cutaneous cold stimuli in rats with oxaliplatin-induced allodynia: electrophysiological and behavioral assessments. The journal of physiological sciences : JPS. 2017(3):431-8.

135. Choi SS, Koh WU, Nam JS, Shin JW, Leem JG, Suh JH. Effect of ethyl pyruvate on Paclitaxel-induced neuropathic pain in rats. Korean J Pain. 2013:135-41.

136. Christensen SB, Hone AJ, Roux I, Kniazeff J, Pin JP, Upert G, et al. RgIA4 Potently Blocks Mouse α9α10 nAChRs and Provides Long Lasting Protection against Oxaliplatin-Induced Cold Allodynia. Front Cell Neurosci. 2017:219.

137. Chtourou Y, Gargouri B, Kebieche M, Fetoui H. Naringin Abrogates Cisplatin-Induced Cognitive Deficits and Cholinergic Dysfunction Through the Down-Regulation of AChE Expression and iNOS Signaling Pathways in Hippocampus of Aged Rats. Journal of Molecular Neuroscience. 2015:349-62.

138. Ciotu IC, Lupuliasa D, Chirita C, Zbarcea CE, Negres S. The antihyperalgic effect of memantine in a rat model of paclitaxel induced neuropathic pain. Farmacia. 2016(5):809-12.

139. Ciotu IC, Lupuliasa D, Zbarcea CE, Negres S. The effect of nimodipine on a rat model of paclitaxel - Induced peripheral neuropathy. Farmacia. 2016(4):493-7.

140. Cliffer KD, Siuciak JA, Carson SR, Radley HE, Park JS, Lewis DR, et al. Physiological characterization of taxol-induced large-fiber sensory neuropathy in the rat. Annals of Neurology. 1998:46-55.

141. Contreras PC, Vaught JL, Gruner JA, Brosnan C, Steffler C, Arezzo JC, et al. Insulin-like growth factor-I prevents development of a vincristine neuropathy in mice. Brain Research. 1997:20-6.

142. Coriat R, Alexandre J, Nicco C, Quinquis L, Benoit E, Chereau C, et al. Treatment of oxaliplatin-induced peripheral neuropathy by intravenous mangafodipir. J Clin Invest. 2014:262-72.

143. Corsetti G, Rodella L, Rezzani R, Stacchiotti A, Bianchi R. Cytoplasmic changes in satellite cells of spinal ganglia induced by cisplatin treatment in rats. Ultrastructural Pathology. 2000:259-65.

144. Costa R, Bicca MA, Manjavachi MN, Segat GC, Dias FC, Fernandes ES, et al. Kinin Receptors Sensitize TRPV4 Channel and Induce Mechanical Hyperalgesia: Relevance to Paclitaxel-Induced Peripheral Neuropathy in Mice. Mol Neurobiol. 2017.

145. Costa R, Motta EM, dutra RC, Manjavachi MN, Bento AF, Malinsky FR, et al. Anti-nociceptive effect of kinin B1 and B2 receptor antagonists on peripheral neuropathy induced by paclitaxel in mice. British Journal of Pharmacology. 2011:681-93.

146. Dambska M, Muzylak M, Maslinska D. The features of peripheral nerve lesions in young and adult rabbits after vincristine administration. Folia neuropathologica / Association of Polish Neuropathologists and Medical Research Centre, Polish Academy of Sciences. 1995:21-4.

147. De Koning P, Neijt JP, Jennekens FGI, Gispen WH. Org. 2766 protects from cisplatin-induced neurotoxicity in rats. Experimental Neurology. 1987:746-50.

148. De Koning P, Neijt JP, Jennekens FGI, Gispen WH. Evaluation of cis-diamminedichloroplatinum (II) (Cisplatin) neurotoxicity in rats. Toxicology and Applied Pharmacology. 1987:81-7.

149. Deng B, Jia L, Pan L, Song A, Wang Y, Tan H, et al. Wen-Luo-Tong Prevents Glial Activation and Nociceptive Sensitization in a Rat Model of Oxaliplatin-Induced Neuropathic Pain. Evid Based Complement Alternat Med. 2016:3629489.

150. Deng L, Cornett BL, Mackie K, Hohmann AG. CB1 Knockout Mice Unveil Sustained CB2-Mediated Antiallodynic Effects of the Mixed CB1/CB2 Agonist CP55,940 in a Mouse Model of Paclitaxel-Induced Neuropathic Pain. Mol Pharmacol. 2015:64-74.

151. Deng L, Guindon J, Cornett BL, Makriyannis A, Mackie K, Hohmann AG. Chronic cannabinoid receptor 2 activation reverses paclitaxel neuropathy without tolerance or cannabinoid receptor 1-dependent withdrawal. Biol Psychiatry. 2015:475-87.

152. Deng L, Lee WH, Xu Z, Makriyannis A, Hohmann AG. Prophylactic treatment with the tricyclic antidepressant desipramine prevents development of paclitaxel-induced neuropathic pain through activation of endogenous analgesic systems. Pharmacological Research. 2016:75-89.

153. Descoeur J, Pereira V, Pizzoccaro A, Francois A, Ling B, Maffre V, et al. Oxaliplatin-induced cold hypersensitivity is due to remodelling of ion channel expression in nociceptors. EMBO Molecular Medicine. 2011:266-78.

154. Deuis JR, Lim YL, Rodrigues de Sousa S, Lewis RJ, Alewood PF, Cabot PJ, et al. Analgesic effects of clinically used compounds in novel mouse models of polyneuropathy induced by oxaliplatin and cisplatin. Neuro Oncol. 2014:1324-32.

155. Deuis JR, Zimmermann K, Romanovsky AA, Possani LD, Cabot PJ, Lewis RJ, et al. An animal model of oxaliplatin-induced cold allodynia reveals a crucial role for Nav1.6 in peripheral pain pathways. Pain. 2013(9):1749-57.

156. Di Cesare Mannelli L, Lucarini E, Micheli L, Mosca I, Ambrosino P, Soldovieri MV, et al. Effects of natural and synthetic isothiocyanate-based H2S-releasers against chemotherapy-induced neuropathic pain: Role of Kv7 potassium channels. Neuropharmacology. 2017:49-59.

157. Di Cesare Mannelli L, Marcoli M, Micheli L, Zanardelli M, Maura G, Ghelardini C, et al. Oxaliplatin evokes P2X7-dependent glutamate release in the cerebral cortex: A pain mechanism mediated by Pannexin 1. Neuropharmacology. 2015:133-41.

158. Di Cesare Mannelli L, Maresca M, Farina C, Scherz MW, Ghelardini C. A model of neuropathic pain induced by sorafenib in the rat: Effect of dimiracetam. Neurotoxicology. 2015:101-7.

159. Di Cesare Mannelli L, Maresca M, Micheli L, Farina C, Scherz MW, Ghelardini C. A rat model of FOLFOX-induced neuropathy: effects of oral dimiracetam in comparison with duloxetine and pregabalin. Cancer Chemother Pharmacol. 2017(6):1091-103.

160. Di Cesare Mannelli L, Pacini A, Bonaccini L, Zanardelli M, Mello T, Ghelardini C. Morphologic features and glial activation in rat oxaliplatin-dependent neuropathic pain. J Pain. 2013:1585-600.

161. Di Cesare Mannelli L, Pacini A, Corti F, Boccella S, Luongo L, Esposito E, et al. Antineuropathic profile of N-palmitoylethanolamine in a rat model of oxaliplatin-induced neurotoxicity. PLoS One. 2015:e0128080.

162. Di Cesare Mannelli L, Pacini A, Matera C, Zanardelli M, Mello T, De Amici M, et al. Involvement of alpha7 nAChR subtype in rat oxaliplatin-induced neuropathy: effects of selective activation. Neuropharmacology. 2014:37-48.

163. Di Cesare Mannelli L, Pacini A, Micheli L, Femia AP, Maresca M, Zanardelli M, et al. Astragali radix: could it be an adjuvant for oxaliplatin-induced neuropathy? Sci Rep. 2017;7:42021.

164. Di Cesare Mannelli L, Pacini A, Micheli L, Tani A, Zanardelli M, Ghelardini C. Glial role in oxaliplatin-induced neuropathic pain. Exp Neurol. 2014:22-33.

165. Di Cesare Mannelli L, Tenci B, Micheli L, Vona A, Corti F, Zanardelli M, et al. Adipose-derived stem cells decrease pain in a rat model of oxaliplatin-induced neuropathy: Role of VEGF-A modulation. Neuropharmacology. 2017.

166. Di Cesare Mannelli L, Zanardelli M, Failli P, Ghelardini C. Oxaliplatin-induced neuropathy: Oxidative Stress as Pathological Mechanism. Protective Effect of Silibinin. Journal of Pain. 2012:276-84.

167. Di Cesare Mannelli L, Zanardelli M, Ghelardini C. Nicotine is a pain reliever in trauma- and chemotherapy-induced neuropathy models. Eur J Pharmacol. 2013:87-94.

168. Di Cesare Mannelli L, Zanardelli M, Landini I, Pacini A, Ghelardini C, Mini E, et al. Effect of the SOD mimetic MnL4 on in vitro and in vivo oxaliplatin toxicity: Possible aid in chemotherapy induced neuropathy. Free Radic Biol Med. 2016:67-76.

169. Dilley A, Richards N, Pulman KG, Bove GM. Disruption of fast axonal transport in the rat induces behavioral changes consistent with neuropathic pain. Journal of Pain. 2013;14(11):1437-49.

170. Dina OA, Chen X, Reichling D, Levine JD. Role of protein kinase Cepsilon and protein kinase A in a model of paclitaxel-induced painful peripheral neuropathy in the rat. Neuroscience. 2001:507-15.

171. Dina OA, Parada CA, Yeh J, Chen X, McCarter GC, Levine JD. Integrin signaling in inflammatory and neuropathic pain in the rat. Eur J Neurosci. 2004:634-42.

172. Djaldetti R, Hart J, Alexandrova S, Cohen S, Beilin BZ, Djaldetti M, et al. Vincristine-induced alterations in Schwann cells of mouse peripheral nerve. American Journal of Hematology. 1996:254-7.

173. Donvito G, Wilkerson JL, Damaj MI, Lichtman AH. Palmitoylethanolamide reverses paclitaxel-induced allodynia in mice. Journal of Pharmacology and Experimental Therapeutics. 2016(2):310-8.

174. Doyle T, Chen Z, Muscoli C, Bryant L, Esposito E, Cuzzocrea S, et al. Targeting the overproduction of peroxynitrite for the prevention and reversal of paclitaxel-induced neuropathic pain. J Neurosci. 2012:6149-60.

175. Draxler P, Honsek SD, Forsthuber L, Hadschieff V, Sandkuhler J. VGluT3(+) primary afferents play distinct roles in mechanical and cold hypersensitivity depending on pain etiology. J Neurosci. 2014:12015-28.

176. Duggett NA, Flatters SJL. Characterization of a rat model of bortezomib-induced painful neuropathy. British Journal of Pharmacology. 2017.

177. Duggett NA, Griffiths LA, Flatters SJL. Paclitaxel-induced painful neuropathy is associated with changes in mitochondrial bioenergetics, glycolysis, and an energy deficit in dorsal root ganglia neurons. Pain. 2017(8):1499-508.

178. Duggett NA, Griffiths LA, McKenna OE, de Santis V, Yongsanguanchai N, Mokori EB, et al. Oxidative stress in the development, maintenance and resolution of paclitaxel-induced painful neuropathy. Neuroscience. 2016:13-26.

179. Dzagnidze A, Katsarava Z, Makhalova J, Liedert B, Yoon MS, Kaube H, et al. Repair capacity for platinum-DNA adducts determines the severity of cisplatin-induced peripheral neuropathy. J Neurosci. 2007:9451-7.

180. Egashira N, Hirakawa S, Kawashiri T, Yano T, Ikesue H, Oishi R. Mexiletine reverses oxaliplatin-induced neuropathic pain in rats. J Pharmacol Sci. 2010:473-6.

181. El-Masry TA, El Sayaad ME, Gaaboub IA, Fouda WM. Effects of capsaicin on rat sciatic nerve in vincristine-induced neuropathic pain model. International Journal of Pharmaceutical Sciences and Research. 2013:663-7.

182. Erken HA, Koc ER, Yazici H, Yay A, Onder GO, Sarici SF. Selenium partially prevents cisplatin-induced neurotoxicity: a preliminary study. Neurotoxicology. 2014:71-5.

183. Fardell JE, Vardy J, Monds LA, Johnston IN. The long-term impact of oxaliplatin chemotherapy on rodent cognition and peripheral neuropathy. Behavioural Brain Research. 2015:80-8.

184. Fariello RG, Ghelardini C, Di Cesare Mannelli L, Bonanno G, Pittaluga A, Milanese M, et al. Broad spectrum and prolonged efficacy of dimiracetam in models of neuropathic pain. Neuropharmacology. 2014:85-94.

185. Farshid AA, Tamaddonfard E, Najafi S. Effects of histidine and n-acetylcysteine on experimental lesions induced by doxorubicin in sciatic nerve of rats. Drug and Chemical Toxicology. 2015:436-41.

186. Favaro G, Di Gregorio F, Panozzo C, Fiori MG. Ganglioside treatment of vincristine-induced neuropathy. An electrophysiologic study. Toxicology. 1988:325-9.

187. Favre-Guilmard C, Auguet M, Chabrier PE. Different antinociceptive effects of botulinum toxin type A in inflammatory and peripheral polyneuropathic rat models. Eur J Pharmacol. 2009:48-53.

188. Ferrari LF, Chum A, Bogen O, Reichling DB, Levine JD. Role of Drp1, a key mitochondrial fission protein, in neuropathic pain. J Neurosci. 2011:11404-10.

189. Ferrier J, Bayet-Robert M, Dalmann R, El Guerrab A, Aissouni Y, Graveron-Demilly D, et al. Cholinergic Neurotransmission in the Posterior Insular Cortex Is Altered in Preclinical Models of Neuropathic Pain: Key Role of Muscarinic M2 Receptors in Donepezil-Induced Antinociception. J Neurosci. 2015(50):16418-30.

190. Ferrier J, Bayet-Robert M, Pereira B, Daulhac L, Eschalier A, Pezet D, et al. A polyamine-deficient diet prevents oxaliplatin-induced acute cold and mechanical hypersensitivity in rats. PLoS One. 2013:e77828.

191. Fidanboylu M, Griffiths LA, Flatters SJL. Global inhibition of reactive oxygen species (ROS) inhibits paclitaxel-induced painful peripheral neuropathy. PLoS ONE. 2011.

192. Fiori MG, Schiavinato A, Lini E, Nunzi MG. Peripheral neuropathy induced by intravenous administration of vincristine sulfate in the rabbit. An ultrastructural study. Toxicol Pathol. 1995:248-55.

193. Fischer SJ, McDonald ES, Gross L, Windebank AJ. Alterations in cell cycle regulation underlie cisplatin induced apoptosis of dorsal root ganglion neurons in vivo. Neurobiol Dis. 2001:1027-35.

194. Fitzgerald M, Woolf CJ, Gibson SJ, Mallaburn PS. Alterations in the structure, function, and chemistry of C fibers following local application of vinblastine to the sciatic nerve of the rat. J Neurosci. 1984:430-41.

195. Flatters SJ, Bennett GJ. Ethosuximide reverses paclitaxel- and vincristine-induced painful peripheral neuropathy. Pain. 2004:150-61.

196. Flatters SJ, Bennett GJ. Studies of peripheral sensory nerves in paclitaxel-induced painful peripheral neuropathy: evidence for mitochondrial dysfunction. Pain. 2006:245-57.

197. Flatters SJL, Xiao WH, Bennett GJ. Acetyl-L-carnitine prevents and reduces paclitaxel-induced painful peripheral neuropathy. Neuroscience Letters. 2006:219-23.

198. Friesland A, Weng Z, Duenas M, Massa SM, Longo FM, Lu Q. Amelioration of cisplatin-induced experimental peripheral neuropathy by a small molecule targeting p75NTR. NeuroToxicology. 2014:81-90.

199. Fujita S, Ushio S, Ozawa N, Masuguchi K, Kawashiri T, Oishi R, et al. Exenatide facilitates recovery from oxaliplatin-induced peripheral neuropathy in rats. PLoS ONE. 2015.

200. Fukuizumi T, Ohkubo T, Kitamura K. Spinal sensitization mechanism in vincristine-induced hyperalgesia in mice. Neuroscience Letters. 2003:89-92.

201. Galeotti N, Vivoli E, Bilia AR, Vincieri FF, Ghelardini C. St. John’s Wort reduces neuropathic pain through a hypericin-mediated inhibition of the protein kinase C g and e activity. Biochem Pharmacol. 2010:1327-36.

202. Galley HF, McCormick B, Wilson KL, Lowes DA, Colvin L, Torsney C. Melatonin limits paclitaxel-induced mitochondrial dysfunction in vitro and protects against paclitaxel-induced neuropathic pain in the rat. J Pineal Res. 2017(4).

203. Gao L, Zheng Y, Zhao C, Teng H. Investigation on effect of basalin coated silver nanoparticles as antioxidant for alleviating peripheral neuropathy in mice treated with oxaliplatin. J Photochem Photobiol B. 2017:56-61.

204. Gao M, Yan X, Weng HR. Inhibition of glycogen synthase kinase 3beta activity with lithium prevents and attenuates paclitaxel-induced neuropathic pain. Neuroscience. 2013:301-11.

205. Gao W, Zan Y, Wang ZJ, Hu XY, Huang F. Quercetin ameliorates paclitaxel-induced neuropathic pain by stabilizing mast cells, and subsequently blocking PKCepsilon-dependent activation of TRPV1. Acta Pharmacol Sin. 2016(9):1166-77.

206. Gao WQ, Dybdal N, Shinsky N, Murnane A, Schmelzer C, Siegel M, et al. Neurotrophin-3 reverses experimental cisplatin-induced peripheral sensory neuropathy. Ann Neurol. 1995:30-7.

207. Garcia JM, Cata JP, Dougherty PM, Smith RG. Ghrelin prevents cisplatin-induced mechanical hyperalgesia and cachexia. Endocrinology. 2008:455-60.

208. Gauchan P, Andoh T, Ikeda K, Fujita M, Sasaki A, Kato A, et al. Mechanical allodynia induced by paclitaxel, oxaliplatin and vincristine: different effectiveness of gabapentin and different expression of voltage-dependent calcium channel alpha(2)delta-1 subunit. Biological and Pharmaceutical Bulletin. 2009:732-4.

209. Gauchan P, Andoh T, Kato A, Kuraishi Y. Involvement of increased expression of transient receptor potential melastatin 8 in oxaliplatin-induced cold allodynia in mice. Neuroscience Letters. 2009:93-5.

210. Gauchan P, Andoh T, Kato A, Sasaki A, Kuraishi Y. Effects of the prostaglandin E1 analog limaprost on mechanical allodynia caused by chemotherapeutic agents in mice. Journal of Pharmacological Sciences. 2009:469-72.

211. Geis C, Beyreuther BK, Stohr T, Sommer C. Lacosamide has protective disease modifying properties in experimental vincristine neuropathy. Neuropharmacology. 2011:600-7.

212. Geisler S, Doan RA, Strickland A, Huang X, Milbrandt J, DiAntonio A. Prevention of vincristine-induced peripheral neuropathy by genetic deletion of SARM1 in mice. Brain. 2016(Pt 12):3092-108.

213. Gerritsen Van Der Hoop R, De Koning P, Boven E, Neijt JP, Jennekens FGI, Gispen WH. Efficacy of the neuropeptide ORG.2766 in the prevention and treatment of cisplatin-induced neurotoxicity in rats. European Journal of Cancer and Clinical Oncology. 1988:637-42.

214. Gerritsen van der Hoop R, Hamers FP, Neijt JP, Veldman H, Gispen WH, Jennekens FG. Protection against cisplatin induced neurotoxicity by ORG 2766: histological and electrophysiological evidence. J Neurol Sci. 1994:109-15.

215. Ghelardini C, Desaphy JF, Muraglia M, Corbo F, Matucci R, Dipalma A, et al. Effects of a new potent analog of tocainide on hNav1.7 sodium channels and in vivo neuropathic pain models. Neuroscience. 2010:863-73.

216. Ghelardini C, Menicacci C, Cerretani D, Bianchi E. Spinal administration of mGluR5 antagonist prevents the onset of bortezomib induced neuropathic pain in rat. Neuropharmacology. 2014:294-300.

217. Ghirardi O, Lo Giudice P, Pisano C, Vertechy M, Bellucci A, Vesci L, et al. Acetyl-L-Carnitine prevents and reverts experimental chronic neurotoxicity induced by oxaliplatin, without altering its antitumor properties. Anticancer Res. 2005:2681-7.

218. Gilardini A, Avila RL, Oggioni N, Rodriguez-Menendez V, Bossi M, Canta A, et al. Myelin structure is unaltered in chemotherapy-induced peripheral neuropathy. NeuroToxicology. 2012:1-7.

219. Gispen WH, Hamers FP, Vecht CJ, Jennekens FG, Neyt JP. ACTH/MSH like peptides in the treatment of cisplatin neuropathy. J Steroid Biochem Mol Biol. 1992:179-83.

220. Golchin L, Shabani M, Harandi S, Razavinasab M. Pistachio supplementation attenuates motor and cognition impairments induced by cisplatin or vincristine in rats. Adv Biomed Res. 2015:92.

221. Goldstein BD, Lowndes HE, Cho ES. Neurotoxicology of vincristine in the cat. Electrophysiological studies. Archives of Toxicology. 1981:253-64.

222. Gong SS, Li YX, Zhang MT, Du J, Ma PS, Yao WX, et al. Neuroprotective Effect of Matrine in Mouse Model of Vincristine-Induced Neuropathic Pain. Neurochem Res. 2016(11):3147-59.

223. Goudet C, Chapuy E, Alloui A, Acher F, Pin JP, Eschalier A. Group III metabotropic glutamate receptors inhibit hyperalgesia in animal models of inflammation and neuropathic pain. Pain. 2008:112-24.

224. Goyal S, Menaria G, Kumar D, Paliwal H, Singla S, Khilnani G, et al. Evaluation of the protective effects of Sapindus trifoliatus aqueous extract on vincristine induced neuropathic pain in rats. Journal of Pharmacy Research. 2016(11):683-91.

225. Green LS, Donoso JA, Heller-Bettinger IE, Samson FE. Axonal transport disturbances in the vincristine-induced peripheral neuropathy. Transactions of the American Neurological Association. 1977:195-6.

226. Greeshma N, Prasanth KG, Balaji B. Tetrahydrocurcumin exerts protective effect on vincristine induced neuropathy: Behavioral, biochemical, neurophysiological and histological evidence. Chem Biol Interact. 2015:118-28.

227. Griffiths LA, Flatters SJL. Pharmacological Modulation of the Mitochondrial Electron Transport Chain in Paclitaxel-Induced Painful Peripheral Neuropathy. Journal of Pain. 2015:981-94.

228. Gris G, Portillo-Salido E, Aubel B, Darbaky Y, Deseure K, Vela JM, et al. The selective sigma-1 receptor antagonist E-52862 attenuates neuropathic pain of different aetiology in rats. Sci Rep. 2016:24591.

229. Gui Q, Xu C, Li D, Zhuang L, Xia S, Yu S. Urinary N telopeptide levels in predicting the anti-nociceptive responses of zoledronic acid and paclitaxel in a rat model of bone metastases. Molecular Medicine Reports. 2015:4243-9.

230. Guindon J, Deng L, Fan B, Wager-Miller J, Hohmann AG. Optimization of a cisplatin model of chemotherapy-induced peripheral neuropathy in mice: use of vitamin C and sodium bicarbonate pretreatments to reduce nephrotoxicity and improve animal health status. Mol Pain. 2014:56.

231. Guindon J, Hohmann AG. Use of sodium bicarbonate to promote weight gain, maintain body temperature, normalize renal functions and minimize mortality in rodents receiving the chemotherapeutic agent cisplatin. Neuroscience Letters. 2013:41-6.

232. Guindon J, Lai Y, Takacs SM, Bradshaw HB, Hohmann AG. Alterations in endocannabinoid tone following chemotherapy-induced peripheral neuropathy: effects of endocannabinoid deactivation inhibitors targeting fatty-acid amide hydrolase and monoacylglycerol lipase in comparison to reference analgesics following cisplatin treatment. Pharmacol Res. 2013:94-109.

233. Guo Z, Man Y, Wang X, Jin H, Sun X, Su X, et al. Levo-tetrahydropalmatine attenuates oxaliplatin-induced mechanical hyperalgesia in mice. Sci Rep. 2014:3905.

234. Hache G, Guiard BP, Nguyen TH, Quesseveur G, Gardier AM, Peters D, et al. Antinociceptive activity of the new triple reuptake inhibitor NS18283 in a mouse model of chemotherapy-induced neuropathic pain. Eur J Pain. 2015:322-33.

235. Hamers FP, van der Hoop RG, Steerenburg PA, Neijt JP, Gispen WH. Putative neurotrophic factors in the protection of cisplatin-induced peripheral neuropathy in rats. Toxicol Appl Pharmacol. 1991:514-22.

236. Hamers FPT, Brakkee JH, Cavalletti E, Tedeschi M, Marmonti L, Pezzoni G, et al. Reduced glutathione protects against cisplatin-induced neurotoxicity in rats. Cancer Research. 1993:544-9.

237. Hamers FPT, Pette C, Bravenboer B, Vecht CJ, Neijt JP, Gispen WH. Cisplatin-induced neuropathy in mature rats: Effects of the melanocortin-derived peptide ORG 2766. Cancer Chemotherapy and Pharmacology. 1993:162-6.

238. Hamity MV, White SR, Walder RY, Schmidt MS, Brenner C, Hammond DL. Nicotinamide riboside, a form of vitamin B3 and NAD + precursor, relieves the nociceptive and aversive dimensions of paclitaxel-induced peripheral neuropathy in female rats. Pain. 2017(5):962-72.

239. Han FY, Wyse BD, Smith MT. Optimization and pharmacological characterization of a refined cisplatin-induced rat model of peripheral neuropathic pain. Behav Pharmacol. 2014:732-40.

240. Han SM, Kim YH, Jo HU, Kwak JA, Park HJ. Tianeptine Reduces Mechanical Allodynia in Spinal Nerve-ligated and Chemotherapy-induced Neuropathic Mice. Pain Physician. 2017(4):E593-e600.

241. Hansen N, Uceyler N, Palm F, Zelenka M, Biko L, Lesch KP, et al. Serotonin transporter deficiency protects mice from mechanical allodynia and heat hyperalgesia in vincristine neuropathy. Neuroscience Letters. 2011:93-7.

242. Hara T, Chiba T, Abe K, Makabe A, Ikeno S, Kawakami K, et al. Effect of paclitaxel on transient receptor potential vanilloid 1 in rat dorsal root ganglion. Pain. 2013:882-9.

243. Harris HM, Sufka KJ, Gul W, ElSohly MA. Effects of Delta-9-Tetrahydrocannabinol and Cannabidiol on Cisplatin-Induced Neuropathy in Mice. Planta Med. 2016(13):1169-72.

244. He ZW, Wei W, Li SP, Ling Q, Liao KJ, Wang X. Anti-allodynic effects of obtusifolin and gluco-obtusifolin against inflammatory and neuropathic pain possible mechanism for neuroinflammation. Biol Pharm Bull. 2014:1606-16.

245. Hidaka T, Shima T, Nagira K, Ieki M, Nakamura T, Aono Y, et al. Herbal medicine Shakuyaku-kanzo-to reduces paclitaxel-induced painful peripheral neuropathy in mice. European Journal of Pain. 2009:22-7.

246. Higuchi H, Yamamoto S, Ushio S, Kawashiri T, Egashira N. Goshajinkigan reduces bortezomib-induced mechanical allodynia in rats: Possible involvement of kappa opioid receptor. Journal of Pharmacological Sciences. 2015:196-9.

247. Hohmann SW, Angioni C, Tunaru S, Lee S, Woolf CJ, Offermanns S, et al. The G2A receptor (GPR132) contributes to oxaliplatin-induced mechanical pain hypersensitivity. Sci Rep. 2017(1):446.

248. Holmes J, Stanko J, Varchenko M, Ding H, Madden VJ, Bagnell CR, et al. Comparative neurotoxicity of oxaliplatin, cisplatin, and ormaplatin in a Wistar rat model. Toxicol Sci. 1998:342-51.

249. Honore P, Donnelly-Roberts D, Namovic MT, Hsieh G, Zhu CZ, Mikusa JP, et al. A-740003 [N-(1-{[(cyanoimino)(5-quinolinylamino) methyl]amino}-2,2-dimethylpropyl)-2-(3,4-dimethoxyphenyl)acetamide], a novel and selective P2X7 receptor antagonist, dose-dependently reduces neuropathic pain in the rat. J Pharmacol Exp Ther. 2006:1376-85.

250. Hori K, Ozaki N, Suzuki S, Sugiura Y. Upregulations of P2X(3) and ASIC3 involve in hyperalgesia induced by cisplatin administration in rats. Pain. 2010:393-405.

251. Horvath P, Szilvassy J, Nemeth J, Peitl B, Szilasi M, Szilvassy Z. Decreased sensory neuropeptide release in isolated bronchi of rats with cisplatin-induced neuropathy. European Journal of Pharmacology. 2005:247-52.

252. Horvath P, Szilvassy Z, Peitl B, Szilvassy J, Helyes Z, Szolcsanyi J, et al. Changes in tracheo-bronchial sensory neuropeptide receptor gene expression pattern in rats with cisplatin-induced sensory neuropathy. Neuropeptides. 2006:77-83.

253. Hsieh YL, Chen HY, Yang CH, Yang CC. Analgesic Effects of Transcutaneous Ultrasound Nerve Stimulation in a Rat Model of Oxaliplatin-Induced Mechanical Hyperalgesia and Cold Allodynia. Ultrasound in Medicine and Biology. 2017(7):1466-75.

254. Hsieh YL, Fan YC, Yang CC. Low-level laser therapy alleviates mechanical and cold allodynia induced by oxaliplatin administration in rats. Supportive Care in Cancer. 2016:233-42.

255. Hu LY, Zhou Y, Cui WQ, Hu XM, Du LX, Mi WL, et al. Triggering receptor expressed on myeloid cells 2 (TREM2) dependent microglial activation promotes cisplatin-induced peripheral neuropathy in mice. Brain, Behavior, and Immunity. 2017.

256. Huang K, Bian D, Jiang B, Zhai Q, Gao N, Wang R. TRPA1 contributed to the neuropathic pain induced by docetaxel treatment. Cell Biochem Funct. 2017(3):141-3.

257. Huang ZZ, Li D, Liu CC, Cui Y, Zhu HQ, Zhang WW, et al. CX3CL1-mediated macrophage activation contributed to paclitaxel-induced DRG neuronal apoptosis and painful peripheral neuropathy. Brain Behav Immun. 2014:155-65.

258. Huang ZZ, Li D, Ou-Yang HD, Liu CC, Liu XG, Ma C, et al. Cerebrospinal Fluid Oxaliplatin Contributes to the Acute Pain Induced by Systemic Administration of Oxaliplatin. Anesthesiology. 2016:1109-21.

259. Huang ZZ, Wei JY, Ou-Yang HD, Li D, Xu T, Wu SL, et al. mir-500-Mediated GAD67 Downregulation Contributes to Neuropathic Pain. J Neurosci. 2016(23):6321-31.

260. Huehnchen P, Boehmerle W, Endres M. Assessment of paclitaxel induced sensory polyneuropathy with "Catwalk" automated gait analysis in mice. PLoS One. 2013:e76772.

261. Hwang BY, Kim ES, Kim CH, Kwon JY, Kim HK. Gender differences in paclitaxel-induced neuropathic pain behavior and analgesic response in rats. Korean J Anesthesiol. 2012:66-72.

262. Ishii N, Tsubouchi H, Miura A, Yanagi S, Ueno H, Shiomi K, et al. Ghrelin alleviates paclitaxel-induced peripheral neuropathy by reducing oxidative stress and enhancing mitochondrial anti-oxidant functions in mice. Eur J Pharmacol. 2017:35-42.

263. Ito N, Sakai A, Miyake N, Maruyama M, Iwasaki H, Miyake K, et al. miR-15b mediates oxaliplatin-induced chronic neuropathic pain through BACE1 down-regulation. Br J Pharmacol. 2017(5):386-95.

264. Ito S, Tajima K, Nogawa M, Inoue N, Kyoi T, Takahashi Y, et al. Etodolac, a cyclooxygenase-2 inhibitor, attenuates paclitaxel-induced peripheral neuropathy in a mouse model of mechanical allodynia. Journal of Pharmacology and Experimental Therapeutics. 2012:53-60.

265. Ito Y, Kobuchi S, Shimizu R, Katsuyama Y. Pharmacokinetic and toxicodynamic evaluation of oxaliplatin-induced neuropathy and hematological toxicity in rats. Cancer Chemotherapy and Pharmacology. 2017:1-7.

266. Ja’afer FM, Hamdan FB, Mohammed FH. Vincristine-induced neuropathy in rat: electrophysiological and histological study. Exp Brain Res. 2006:334-45.

267. Jaggi AS, Kaur G, Bali A, Singh N. Pharmacological investigations on mast cell stabilizer and histamine receptor antagonists in vincristine-induced neuropathic pain. Naunyn-Schmiedeberg's Archives of Pharmacology. 2017(11):1087-96.

268. Jaggi AS, Singh N. Differential effect of spironolactone in chronic constriction injury and vincristine-induced neuropathic pain in rats. Eur J Pharmacol. 2010:102-9.

269. Jaggi AS, Singh N. Analgesic potential of intrathecal farnesyl thiosalicylic acid and GW 5074 in vincristine-induced neuropathic pain in rats. Food Chem Toxicol. 2012:1295-301.

270. Jain V, Jaggi A, Singh N. Non-beneficial effects of rosiglitazone in oxaliplatin-induced cold hyperalgesia in rats. Journal of Pharmaceutical Negative Results. 2011:28-34.

271. James SE, Dunham M, Carrion-Jones M, Murashov A, Lu Q. Rho kinase inhibitor Y-27632 facilitates recovery from experimental peripheral neuropathy induced by anti-cancer drug cisplatin. Neurotoxicology. 2010:188-94.

272. Jamieson SM, Liu J, Hsu T, Baguley BC, McKeage MJ. Paclitaxel induces nucleolar enlargement in dorsal root ganglion neurons in vivo reducing oxaliplatin toxicity. Br J Cancer. 2003:1942-7.

273. Jamieson SMF, Liu J, Connor B, McKeage MJ. Oxaliplatin causes selective atrophy of a subpopulation of dorsal root ganglion neurons without inducing cell loss. Cancer Chemotherapy and Pharmacology. 2005:391-9.

274. Janes K, Doyle T, Bryant L, Esposito E, Cuzzocrea S, Ryerse J, et al. Bioenergetic deficits in peripheral nerve sensory axons during chemotherapy-induced neuropathic pain resulting from peroxynitrite-mediated post-translational nitration of mitochondrial superoxide dismutase. Pain. 2013:2432-40.

275. Janes K, Esposito E, Doyle T, Cuzzocrea S, Tosh DK, Jacobson KA, et al. A3 adenosine receptor agonist prevents the development of paclitaxel-induced neuropathic pain by modulating spinal glial-restricted redox-dependent signaling pathways. Pain. 2014:2560-7.

276. Janes K, Little JW, Li C, Bryant L, Chen C, Chen Z, et al. The development and maintenance of paclitaxel-induced neuropathic pain require activation of the sphingosine 1-phosphate receptor subtype 1. J Biol Chem. 2014:21082-97.

277. Janes K, Wahlman C, Little JW, Doyle T, Tosh DK, Jacobson KA, et al. Spinal neuroimmune activation is independent of T-cell infiltration and attenuated by A3 adenosine receptor agonists in a model of oxaliplatin-induced peripheral neuropathy. Brain Behav Immun. 2015:91-9.

278. Ji XT, Qian NS, Zhang T, Li JM, Li XK, Wang P, et al. Spinal astrocytic activation contributes to mechanical allodynia in a rat chemotherapy-induced neuropathic pain model. PLoS One. 2013:e60733.

279. Jia M, Wu C, Gao F, Xiang H, Sun N, Peng P, et al. Activation of NLRP3 inflammasome in peripheral nerve contributes to paclitaxel-induced neuropathic pain. Mol Pain. 2017:1744806917719804.

280. Jiang SP, Zhang ZD, Kang LM, Wang QH, Zhang L, Chen HP. Celecoxib reverts oxaliplatin-induced neuropathic pain through inhibiting PI3K/Akt2 pathway in the mouse dorsal root ganglion. Exp Neurol. 2016:11-6.

281. Jin HW, Flatters SJ, Xiao WH, Mulhern HL, Bennett GJ. Prevention of paclitaxel-evoked painful peripheral neuropathy by acetyl-L-carnitine: effects on axonal mitochondria, sensory nerve fiber terminal arbors, and cutaneous Langerhans cells. Exp Neurol. 2008:229-37.

282. Jin HY, Lee NY, Ko HA, Lee KA, Park TS. Comparison of sensory tests and neuronal quantity of peripheral nerves between streptozotocin (STZ)-induced diabetic rats and paclitaxel (PAC)-treated rats. Somatosens Mot Res. 2016(3-4):186-95.

283. Johnston IN, Tan M, Cao J, Matsos A, Forrest DRL, Si E, et al. Ibudilast reduces oxaliplatin-induced tactile allodynia and cognitive impairments in rats. Behav Brain Res. 2017:109-18.

284. Jortner BS, Cho ES. Neurotoxicity of quelamycin in the rat. NeuroToxicology. 1981:789-92.

285. Joseph EK, Chen X, Bogen O, Levine JD. Oxaliplatin acts on IB4-positive nociceptors to induce an oxidative stress-dependent acute painful peripheral neuropathy. J Pain. 2008:463-72.

286. Joseph EK, Levine JD. Sexual dimorphism for protein kinase c epsilon signaling in a rat model of vincristine-induced painful peripheral neuropathy. Neuroscience. 2003:831-8.

287. Joseph EK, Levine JD. Caspase signalling in neuropathic and inflammatory pain in the rat. Eur J Neurosci. 2004:2896-902.

288. Joseph EK, Levine JD. Comparison of oxaliplatin- and cisplatin-induced painful peripheral neuropathy in the rat. J Pain. 2009:534-41.

289. Jung Y, Lee JH, Kim W, Yoon SH, Kim SK. Anti-allodynic effect of Buja in a rat model of oxaliplatin-induced peripheral neuropathy via spinal astrocytes and pro-inflammatory cytokines suppression. BMC Complement Altern Med. 2017(1):48.

290. Jungwirth U, Xanthos DN, Gojo J, Bytzek AK, Korner W, Heffeter P, et al. Anticancer activity of methyl-substituted oxaliplatin analogs. Molecular Pharmacology. 2012:719-28.

291. Kahng J, Kim TK, Chung EY, Kim YS, Moon JY. The effect of thioctic acid on allodynia in a rat vincristine-induced neuropathy model. J Int Med Res. 2015:350-5.

292. Kamei J, Hayashi S, Sakai A, Nakanishi Y, Kai M, Ikegami M, et al. Rikkunshito prevents paclitaxel-induced peripheral neuropathy through the suppression of the nuclear factor kappa B (NFkappaB) phosphorylation in spinal cord of mice. PLoS One. 2017(2):e0171819.

293. Kamei J, Nozaki C, Saitoh A. Effect of mexiletine on vincristine-induced painful neuropathy in mice. Eur J Pharmacol. 2006:123-7.

294. Kamei J, Tamura N, Saitoh A. Possible involvement of the spinal nitric oxide/cGMP pathway in vincristine-induced painful neuropathy in mice. Pain. 2005:112-20.

295. Kamisli S, Ciftci O, Cetin A, Kaya K, Kamisli O, Celik H. Fish oil protects the peripheral and central nervous systems against cisplatin-induced neurotoxicity. Nutritional Neuroscience. 2014:116-26.

296. Kamisli S, Ciftci O, Kaya K, Cetin A, Kamisli O, Ozcan C. Hesperidin protects brain and sciatic nerve tissues against cisplatin-induced oxidative, histological and electromyographical side effects in rats. Toxicology and Industrial Health. 2015:841-51.

297. Kanat O, Bagdas D, Ozboluk HY, Gurun MS. Preclinical evidence for the antihyperalgesic activity of CDP-choline in oxaliplatin-induced neuropathic pain. J buon. 2013:1012-8.

298. Kanbara T, Nakamura A, Shibasaki M, Mori T, Suzuki T, Sakaguchi G, et al. Morphine and oxycodone, but not fentanyl, exhibit antinociceptive effects mediated by G-protein inwardly rectifying potassium (GIRK) channels in an oxaliplatin-induced neuropathy rat model. Neurosci Lett. 2014:119-24.

299. Kanbara T, Nakamura A, Takasu K, Ogawa K, Shibasaki M, Mori T, et al. The contribution of Gi/o protein to opioid antinociception in an oxaliplatin-induced neuropathy rat model. Journal of Pharmacological Sciences. 2014:264-73.

300. Kassem LA, Gamal El-Din MM, Yassin NA. Mechanisms of vincristine-induced neurotoxicity: Possible reversal by erythropoietin. Drug Discov Ther. 2011:136-43.

301. Kato Y, Tateai Y, Ohkubo M, Saito Y, Amagai SY, Kimura YS, et al. Gosha-jinki-gan reduced oxaliplatin-induced hypersensitivity to cold sensation and its effect would be related to suppression of the expression of TRPM8 and TRPA1 in rats. Anti-Cancer Drugs. 2014:39-43.

302. Katsuyama S, Aso H, Otowa A, Yagi T, Kishikawa Y, Komatsu T, et al. Antinociceptive Effects of the Serotonin and Noradrenaline Reuptake Inhibitors Milnacipran and Duloxetine on Vincristine-Induced Neuropathic Pain Model in Mice. ISRN Pain. 2014:915464.

303. Katsuyama S, Kuwahata H, Yagi T, Kishikawa Y, Komatsu T, Sakurada T, et al. Intraplantar injection of linalool reduces paclitaxel-induced acute pain in mice. Biomedical Research (Japan). 2012:175-81.

304. Katsuyama S, Sato K, Yagi T, Kishikawa Y, Nakamura H. Effects of repeated milnacipran and fluvoxamine treatment on mechanical allodynia in a mouse paclitaxel-induced neuropathic pain model. Biomed Res. 2013:105-11.

305. Kaur G, Jaggi AS, Singh N. Exploring the potential effect of Ocimum sanctum in vincristine-induced neuropathic pain in rats. J Brachial Plex Peripher Nerve Inj. 2010:3.

306. Kawakami K, Chiba T, Katagiri N, Saduka M, Abe K, Utsunomiya I, et al. Paclitaxel increases high voltage-dependent calcium channel current in dorsal root ganglion neurons of the rat. J Pharmacol Sci. 2012:187-95.

307. Kawashiri T, Egashira N, Itoh Y, Shimazoe T, Ikegami Y, Yano T, et al. Neurotropin reverses paclitaxel-induced neuropathy without affecting anti-tumour efficacy. European Journal of Cancer. 2009:154-63.

308. Kawashiri T, Egashira N, Kurobe K, Tsutsumi K, Yamashita Y, Ushio S, et al. L type Ca2+ channel blockers prevent

oxaliplatin-induced cold hyperalgesia

and TRPM8 overexpression in rats. Molecular Pain. 2012.

309. Kawashiri T, Egashira N, Watanabe H, Ikegami Y, Hirakawa S, Mihara Y, et al. Prevention of oxaliplatin-induced mechanical allodynia and neurodegeneration by neurotropin in the rat model. European Journal of Pain. 2011:344-50.

310. Kawata D, Wu Z. Regulatable Transgene Expression for Prevention of Chemotherapy-Induced Peripheral Neuropathy. Molecular Therapy - Methods and Clinical Development. 2017:91-101.

311. Khasabova IA, Khasabov S, Paz J, Harding-Rose C, Simone DA, Seybold VS. Cannabinoid type-1 receptor reduces pain and neurotoxicity produced by chemotherapy. J Neurosci. 2012:7091-101.

312. Khasabova IA, Yao X, Paz J, Lewandowski CT, Lindberg AE, Coicou L, et al. JZL184 is anti-hyperalgesic in a murine model of cisplatin-induced peripheral neuropathy. Pharmacol Res. 2014:67-75.

313. Kiguchi N, Maeda T, Kobayashi Y, Kishioka S. Up-regulation of tumor necrosis factor-alpha in spinal cord contributes to vincristine-induced mechanical allodynia in mice. Neuroscience Letters. 2008:140-3.

314. Kiguchi N, Maeda T, Kobayashi Y, Kondo T, Ozaki M, Kishioka S. The critical role of invading peripheral macrophage-derived interleukin-6 in vincristine-induced mechanical allodynia in mice. Eur J Pharmacol. 2008:87-92.

315. Kilpatrick TJ, Phan S, Reardon K, Lopes EC, Cheema SS. Leukaemia inhibitory factor abrogates Paclitaxel-induced axonal atrophy in the Wistar rat. Brain Research. 2001:163-7.

316. Kim HK, Hwang SH, Abdi S. Tempol ameliorates and prevents mechanical hyperalgesia in a rat model of chemotherapy-induced neuropathic pain. Frontiers in Pharmacology. 2017(532).

317. Kim HK, Hwang SH, Lee SO, Kim SH, Abdi S. Pentoxifylline Ameliorates Mechanical Hyperalgesia in a Rat Model of Chemotherapy-Induced Neuropathic Pain. Pain Physician. 2016(4):E589-600.

318. Kim HK, Hwang SH, Oh E, Abdi S. Rolipram, a selective phosphodiesterase 4 inhibitor, ameliorates mechanical hyperalgesia in a rat model of chemotherapy-induced neuropathic pain through inhibition of inflammatory cytokines in the dorsal root ganglion. Frontiers in Pharmacology. 2017(885).

319. Kim HK, Kwon JY, Yoo C, Abdi S. The Analgesic Effect of Rolipram, a Phosphodiesterase 4 Inhibitor, on Chemotherapy-Induced Neuropathic Pain in Rats. Anesth Analg. 2015:822-8.

320. Kim HK, Zhang YP, Gwak YS, Abdi S. Phenyl N-tert-butylnitrone, a free radical scavenger, reduces mechanical allodynia in chemotherapy-induced neuropathic pain in rats. Anesthesiology. 2010:432-9.

321. Kim ST, Chung YH, Lee HS, Chung SJ, Lee JH, Sohn UD, et al. Protective effects of phosphatidylcholine on oxaliplatin-induced neuropathy in rats. Life sciences. 2015:81-7.

322. Kim ST, Kyung EJ, Suh JS, Lee HS, Lee JH, Chae SI, et al. Phosphatidylcholine attenuated docetaxel-induced peripheral neurotoxicity in rats. Drug Chem Toxicol. 2017:1-10.

323. Kim W, Chung Y, Choi S, Min BI, Kim SK. Duloxetine Protects against Oxaliplatin-Induced Neuropathic Pain and Spinal Neuron Hyperexcitability in Rodents. Int J Mol Sci. 2017(12).

324. Kim W, Kim MJ, Go D, Min BI, Na HS, Kim SK. Combined Effects of Bee Venom Acupuncture and Morphine on Oxaliplatin-Induced Neuropathic Pain in Mice. Toxins (Basel). 2016.

325. King KM, Myers AM, Soroka-Monzo AJ, Tuma RF, Tallarida RJ, Walker EA, et al. Single and combined effects of Delta(9) -tetrahydrocannabinol and cannabidiol in a mouse model of chemotherapy-induced neuropathic pain. Br J Pharmacol. 2017(17):2832-41.

326. Kirchmair R, Tietz AB, Panagiotou E, Walter DH, Silver M, Yoon YS, et al. Therapeutic angiogenesis inhibits or rescues chemotherapy-induced peripheral neuropathy: Taxol- and thalidomide-induced injury of vasa nervorum is ameliorated by VEGF. Molecular Therapy. 2007:69-75.

327. Kirchmair R, Walter DH, Ii M, Rittig K, Tietz AB, Murayama T, et al. Antiangiogenesis mediates cisplatin-induced peripheral neuropathy attenuation or reversal by local vascular endothelial growth factor gene therapy without augmenting tumor growth. Circulation. 2005:2662-70.

328. Kitamura R, Andoh T, Fushimi H, Komatsu K, Shibahara N, Kuraishi Y. Involvement of descending monoaminergic systems in antiallodynic effect of goshajinkigan in oxaliplatintreated mice. Journal of Traditional Medicines. 2013:183-9.

329. Kitamura R, Andoh T, Mizoguchi S, Saito Y, Takahata H, Kuraishi Y. Gabapentin inhibits bortezomib-induced mechanical allodynia through supraspinal action in mice. Journal of Pharmacological Sciences. 2014:502-10.

330. Kiya T, Kawamata T, Namiki A, Yamakage M. Role of satellite cell-derived L-serine in the dorsal root ganglion in paclitaxel-induced painful peripheral neuropathy. Neuroscience. 2011:190-9.

331. Ko MH, Hu ME, Hsieh YL, Lan CT, Tseng TJ. Peptidergic intraepidermal nerve fibers in the skin contribute to the neuropathic pain in paclitaxel-induced peripheral neuropathy. Neuropeptides. 2014:109-17.

332. Kono T, Suzuki Y, Mizuno K, Miyagi C, Omiya Y, Sekine H, et al. Preventive effect of oral goshajinkigan on chronic oxaliplatin-induced hypoesthesia in rats. Sci Rep. 2015:16078.

333. Kozachik SL, Opp MR, Page GG. Recovery sleep does not mitigate the effects of prior sleep loss on paclitaxel-induced mechanical hypersensitivity in Sprague-Dawley rats. Biological research for nursing. 2015:207-13.

334. Kozachik SL, Page GG. A Hyperresponsive HPA Axis May Confer Resilience Against Persistent Paclitaxel-Induced Mechanical Hypersensitivity. Biol Res Nurs. 2016:290-8.

335. Krishnaveni A, Gokila P, Murugeswari K, Periyanayagam K. Effect of hydroalcoholic extract of samanea saman barks in vincristine-induced peripheral neuropathy in rats. Journal of Global Trends in Pharmaceutical Sciences. 2017(1):3544-53.

336. Krukowski K, Eijkelkamp N, Laumet G, Hack CE, Li Y, Dougherty PM, et al. CD8+ T Cells and Endogenous IL-10 Are Required for Resolution of Chemotherapy-Induced Neuropathic Pain. Journal of Neuroscience. 2016(43):11074-83.

337. Krukowski K, Ma J, Golonzhka O, Laumet GO, Gutti T, Van Duzer JH, et al. HDAC6 inhibition effectively reverses chemotherapy-induced peripheral neuropathy. Pain. 2017(6):1126-37.

338. Krukowski K, Nijboer CH, Huo X, Kavelaars A, Heijnen CJ. Prevention of chemotherapy-induced peripheral neuropathy by the small-molecule inhibitor pifithrin-mu. Pain. 2015:2184-92.

339. Kuyrukluyildiz U, Kupeli I, Bedir Z, Ozmen O, Onk D, Suleyman B, et al. The Effect of Anakinra on Paclitaxel-Induced Peripheral Neuropathic Pain in Rats. Turk J Anaesthesiol Reanim. 2016(6):287-94.

340. Kyte SL, Toma W, Bagdas D, Meade JA, Schurman LD, Lichtman AH, et al. Nicotine prevents and reverses paclitaxel-induced mechanical allodynia in a mouse model of CIPN. J Pharmacol Exp Ther. 2017.

341. Leandri M, Ghignotti M, Emionite L, Leandri S, Cilli M. Electrophysiological features of the mouse tail nerves and their changes in chemotherapy induced peripheral neuropathy (CIPN). Journal of Neuroscience Methods. 2012:403-9.

342. Ledeboer A, Jekich BM, Sloane EM, Mahoney JH, Langer SJ, Milligan ED, et al. Intrathecal interleukin-10 gene therapy attenuates paclitaxel-induced mechanical allodynia and proinflammatory cytokine expression in dorsal root ganglia in rats. Brain Behav Immun. 2007:686-98.

343. Lee JH, Go D, Kim W, Lee G, Bae H, Quan FS, et al. Involvement of spinal muscarinic and serotonergic receptors in the anti-allodynic effect of electroacupuncture in rats with oxaliplatin-induced neuropathic pain. Korean J Physiol Pharmacol. 2016(4):407-14.

344. Lee JH, Li DX, Yoon H, Go D, Quan FS, Min BI, et al. Serotonergic mechanism of the relieving effect of bee venom acupuncture on oxaliplatin-induced neuropathic cold allodynia in rats. BMC Complement Altern Med. 2014:471.

345. Lee JS, Kim YT, Jeon EK, Won HS, Cho YS, Ko YH. Effect of green tea extracts on oxaliplatin-induced peripheral neuropathy in rats. BMC Complement Altern Med. 2012:124.

346. Lee M, Cho S, Roh K, Chae J, Park JH, Park J, et al. Glutathione alleviated peripheral neuropathy in oxaliplatin-treated mice by removing aluminum from dorsal root ganglia. Am J Transl Res. 2017(3):926-39.

347. Lee-Kubli CAG, Calcutt NA. Altered rate-dependent depression of the spinal H-reflex as an indicator of spinal disinhibition in models of neuropathic pain. Pain. 2014(2):250-60.

348. Leo M, Schmitt LI, Erkel M, Melnikova M, Thomale J, Hagenacker T. Cisplatin-induced neuropathic pain is mediated by upregulation of N-type voltage-gated calcium channels in dorsal root ganglion neurons. Exp Neurol. 2017:62-74.

349. Leo M, Schmitt LI, Jastrow H, Thomale J, Kleinschnitz C, Hagenacker T. Cisplatin alters the function and expression of N-type voltage-gated calcium channels in the absence of morphological damage of sensory neurons. Mol Pain. 2017:1744806917746565.

350. Leonetti C, Biroccio A, Gabellini C, Scarsella M, Maresca V, Flori E, et al. Alpha-tocopherol protects against cisplatin-induced toxicity without interfering with antitumor efficacy. Int J Cancer. 2003:243-50.

351. Li D, Chen H, Luo XH, Sun Y, Xia W, Xiong YC. CX3CR1-Mediated Akt1 Activation Contributes to the Paclitaxel-Induced Painful Peripheral Neuropathy in Rats. Neurochemical Research. 2016(6):1305-14.

352. Li D, Huang ZZ, Ling YZ, Wei JY, Cui Y, Zhang XZ, et al. Up-regulation of CX3CL1 via Nuclear Factor-kappaB-dependent Histone Acetylation Is Involved in Paclitaxel-induced Peripheral Neuropathy. Anesthesiology. 2015:1142-51.

353. Li D, Kim W, Shin D, Jung Y, Bae H, Kim SK. Preventive Effects of Bee Venom Derived Phospholipase A(2) on Oxaliplatin-Induced Neuropathic Pain in Mice. Toxins (Basel). 2016.

354. Li D, Lee Y, Kim W, Lee K, Bae H, Kim SK. Analgesic Effects of Bee Venom Derived Phospholipase A2 in

a Mouse Model of Oxaliplatin-Induced Neuropathic Pain. Toxins. 2015:2422-34.

355. Li Y, Kosturakis AK, Cassidy RM, Zhang H, Kennamer-Chapman RM, Jawad AB, et al. MAPK signaling downstream to TLR4 contributes to paclitaxel-induced peripheral neuropathy. Brain, Behavior, and Immunity. 2015:255-66.

356. Li Y, Tatsui CE, Rhines LD, North RY, Harrison DS, Cassidy RM, et al. Dorsal root ganglion neurons become hyperexcitable and increase expression of voltage-gated T-type calcium channels (Cav3.2) in paclitaxel-induced peripheral neuropathy. Pain. 2017(3):417-29.

357. Li ZY, Zhang YP, Zhang J, Zhang SB, Li D, Huang ZZ, et al. The possible involvement of JNK activation in the spinal dorsal horn in bortezomib-induced allodynia: the role of TNF-alpha and IL-1beta. Journal of Anesthesia. 2016:55-63.

358. Lim BS, Moon HJ, Li DX, Gil M, Min JK, Lee G, et al. Effect of bee venom acupuncture on oxaliplatin-induced cold allodynia in rats. Evidence-based Complementary and Alternative Medicine. 2013.

359. Lin H, Heo BH, Yoon MH. A New Rat Model of Cisplatin-induced Neuropathic Pain. Korean J Pain. 2015:236-43.

360. Lin HM, Lin LF, Xia ZZ, Mao Y, Liu J, Xu LY, et al. Neuroprotective effects and UPLC-Q-TOF/MS-based active components identification of external applied a novel Wen-Luo-Tong microemulsion. Artif Cells Nanomed Biotechnol. 2017:1-11.

361. Lin X, Dhopeshwarkar AS, Huibregtse M, Mackie K, Hohmann AG. Slowly Signaling G Protein-Biased CB2 Cannabinoid Receptor Agonist LY2828360 Suppresses Neuropathic Pain with Sustained Efficacy and Attenuates Morphine Tolerance and Dependence. Mol Pharmacol. 2018;93(2):49-62.

362. Ling B, Authier N, Balayssac D, Eschalier A, Coudore F. Behavioral and pharmacological description of oxaliplatin-induced painful neuropathy in rat. Pain. 2007:225-34.

363. Ling B, Coudore F, Decalonne L, Eschalier A, Authier N. Comparative antiallodynic activity of morphine, pregabalin and lidocaine in a rat model of neuropathic pain produced by one oxaliplatin injection. Neuropharmacology. 2008:724-8.

364. Ling B, Coudore-Civiale MA, Balayssac D, Eschalier A, Coudore F, Authier N. Behavioral and immunohistological assessment of painful neuropathy induced by a single oxaliplatin injection in the rat. Toxicology. 2007:176-84.

365. Ling YZ, Li ZY, Ou-Yang HD, Ma C, Wu SL, Wei JY, et al. The inhibition of spinal synaptic plasticity mediated by activation of AMP-activated protein kinase signaling alleviates the acute pain induced by oxaliplatin. Experimental Neurology. 2017:85-93.

366. Linglu D, Yuxiang L, Yaqiong X, Ru Z, Lin M, Shaoju J, et al. Antinociceptive effect of matrine on vincristine-induced neuropathic pain model in mice. Neurol Sci. 2014:815-21.

367. Lisse TS, Middleton LJ, Pellegrini AD, Martin PB, Spaulding EL, Lopes O, et al. Paclitaxel-induced epithelial damage and ectopic MMP-13 expression promotes neurotoxicity in zebrafish. Proc Natl Acad Sci U S A. 2016.

368. Liu C, Luan S, OuYang H, Huang Z, Wu S, Ma C, et al. Upregulation of CCL2 via ATF3/c-Jun interaction mediated the Bortezomib-induced peripheral neuropathy. Brain, Behavior, and Immunity. 2016:96-104.

369. Liu CC, Lu N, Cui Y, Yang T, Zhao ZQ, Xin WJ, et al. Prevention of paclitaxel-induced allodynia by minocycline: Effect on loss of peripheral nerve fibers and infiltration of macrophages in rats. Mol Pain. 2010:76.

370. Liu H, Wang CH, Yang H, Wang F. Effects of fisetin on oxaliplatin-induced neuropathic pain in mice. Bangladesh Journal of Pharmacology. 2015:138-42.

371. Liu HP, Ren TW, Yan WJ, Liu J, Liu RB. Ellagic acid alleviates inflammatory pain and paclitaxel-induced neuropathic pain in murine models. International Journal of Clinical and Experimental Medicine. 2016(7):12514-20.

372. Liu X, Zhang G, Dong L, Wang X, Sun H, Shen J, et al. Repeated administration of mirtazapine attenuates oxaliplatin-induced mechanical allodynia and spinal NR2B up-regulation in rats. Neurochem Res. 2013:1973-9.

373. LoCoco PM, Risinger AL, Smith HR, Chavera TS, Berg KA, Clarke WP. Pharmacological augmentation of nicotinamide phosphoribosyltransferase (NAMPT) protects against paclitaxel-induced peripheral neuropathy. Elife. 2017.

374. Lolignier S, Bonnet C, Gaudioso C, Noel J, Ruel J, Amsalem M, et al. The Nav1.9 Channel Is a Key Determinant of Cold Pain Sensation and Cold Allodynia. Cell Reports. 2015:1067-78.

375. Lucas D, Scheiermann C, Chow A, Kunisaki Y, Bruns I, Barrick C, et al. Chemotherapy-induced bone marrow nerve injury impairs hematopoietic regeneration. Nature Medicine. 2013:695-703.

376. Luo J, Bavencoffe A, Yang P, Feng J, Yin S, Qian A, et al. Zinc inhibits TRPV1 to alleviate chemotherapy-induced neuropathic pain. J Neurosci. 2017.

377. Luo X, Fitzsimmons B, Mohan A, Zhang L, Terrando N, Kordasiewicz H, et al. Intrathecal administration of antisense oligonucleotide against p38alpha but not p38beta MAP kinase isoform reduces neuropathic and postoperative pain and TLR4-induced pain in male mice. Brain Behav Immun. 2017.

378. Lynch JJ, 3rd, Wade CL, Mikusa JP, Decker MW, Honore P. ABT-594 (a nicotinic acetylcholine agonist): anti-allodynia in a rat chemotherapy-induced pain model. Eur J Pharmacol. 2005:43-8.

379. Lynch JJ, 3rd, Wade CL, Zhong CM, Mikusa JP, Honore P. Attenuation of mechanical allodynia by clinically utilized drugs in a rat chemotherapy-induced neuropathic pain model. Pain. 2004:56-63.

380. Maj MA, Ma J, Krukowski KN, Kavelaars A, Heijnen CJ. Inhibition of mitochondrial p53 accumulation by PFT-micro prevents cisplatin-induced peripheral neuropathy. Frontiers in Molecular Neuroscience. 2017(108).

381. Makker PG, Duffy SS, Lees JG, Perera CJ, Tonkin RS, Butovsky O, et al. Characterisation of Immune and Neuroinflammatory Changes Associated with Chemotherapy-Induced Peripheral Neuropathy. PLoS One. 2017(1):e0170814.

382. Mangaiarkkarasi A, Rameshkannan S, Ali RM. Effect of Gabapentin and Pregabalin in Rat Model of Taxol Induced Neuropathic Pain. J Clin Diagn Res. 2015:Ff11-4.

383. Marchand F, Alloui A, Pelissier T, Hernandez A, Authier N, Alvarez P, et al. Evidence for an antihyperalgesic effect of venlafaxine in vincristine-induced neuropathy in rat. Brain Res. 2003:117-20.

384. Masocha W. Paclitaxel-induced hyposensitivity to nociceptive chemical stimulation in mice can be prevented by treatment with minocycline. Scientific reports. 2014:6719.

385. Masocha W. Comprehensive analysis of the GABAergic system gene expression profile in the anterior cingulate cortex of mice with Paclitaxel-induced neuropathic pain. Gene Expr. 2015:145-53.

386. Masocha W. Gene expression profile of sodium channel subunits in the anterior cingulate cortex during experimental paclitaxel-induced neuropathic pain in mice. PeerJ. 2016:e2702.

387. Masocha W, Parvathy SS. Preventative and therapeutic effects of a GABA transporter 1 inhibitor administered systemically in a mouse model of paclitaxel-induced neuropathic pain. PeerJ. 2016:e2798.

388. Massicot F, Hache G, David L, Chen D, Leuxe C, Garnier-Legrand L, et al. P2X7 Cell Death Receptor Activation and Mitochondrial Impairment in Oxaliplatin-Induced Apoptosis and Neuronal Injury: Cellular Mechanisms and In Vivo Approach. PLoS ONE. 2013.

389. Masuguchi K, Watanabe H, Kawashiri T, Ushio S, Ozawa N, Morita H, et al. Neurotropin(R) relieves oxaliplatin-induced neuropathy via Gi protein-coupled receptors in the monoaminergic descending pain inhibitory system. Life Sci. 2014:49-54.

390. Matsumoto M, Inoue M, Hald A, Xie W, Ueda H. Inhibition of paclitaxel-induced A-fiber hypersensitization by gabapentin. J Pharmacol Exp Ther. 2006:735-40.

391. Matsumura Y, Yokoyama Y, Hirakawa H, Shigeto T, Futagami M, Mizunuma H. The prophylactic effects of a traditional Japanese medicine, goshajinkigan, on paclitaxel-induced peripheral neuropathy and its mechanism of action. Molecular pain. 2014:61.

392. McCormick B, Lowes DA, Colvin L, Torsney C, Galley HF. MitoVitE, a mitochondria-targeted antioxidant, limits paclitaxel-induced oxidative stress and mitochondrial damage in vitro, and paclitaxel-induced mechanical hypersensitivity in a rat pain model. Br J Anaesth. 2016(5):659-66.

393. McKeage MJ, Boxall FE, Jones M, Harrap KR. Lack of neurotoxicity of oral bisacetatoamminedichlorocyclohexylamine-platinum(IV) in comparison to cisplatin and tetraplatin in the rat. Cancer Res. 1994:629-31.

394. Melli G, Jack C, Lambrinos GL, Ringkamp M, Hoke A. Erythropoietin protects sensory axons against paclitaxel-induced distal degeneration. Neurobiol Dis. 2006:525-30.

395. Meng X, Zhang Y, Li A, Xin J, Lao L, Ren K, et al. The effects of opioid receptor antagonists on electroacupuncture-produced anti-allodynia/hyperalgesia in rats with paclitaxel-evoked peripheral neuropathy. Brain Res. 2011:58-65.

396. Meregalli C, Canta A, Carozzi VA, Chiorazzi A, Oggioni N, Gilardini A, et al. Bortezomib-induced painful neuropathy in rats: a behavioral, neurophysiological and pathological study in rats. Eur J Pain. 2010:343-50.

397. Meregalli C, Ceresa C, Canta A, Carozzi VA, Chiorazzi A, Sala B, et al. CR4056, a new analgesic I2 ligand, is highly effective against bortezomib-induced painful neuropathy in rats. J Pain Res. 2012:151-67.

398. Meregalli C, Chiorazzi A, Carozzi VA, Canta A, Sala B, Colombo M, et al. Evaluation of tubulin polymerization and chronic inhibition of proteasome as citotoxicity mechanisms in bortezomib-induced peripheral neuropathy. Cell Cycle. 2014:612-21.

399. Meyer L, Patte-Mensah C, Taleb O, Mensah-Nyagan AG. Cellular and functional evidence for a protective action of neurosteroids against vincristine chemotherapy-induced painful neuropathy. Cell Mol Life Sci. 2010:3017-34.

400. Meyer L, Patte-Mensah C, Taleb O, Mensah-Nyagan AG. Allopregnanolone prevents and suppresses oxaliplatin-evoked painful neuropathy: multi-parametric assessment and direct evidence. Pain. 2011:170-81.

401. Meyer L, Patte-Mensah C, Taleb O, Mensah-Nyagan AG. Neurosteroid 3alpha-androstanediol efficiently counteracts paclitaxel-induced peripheral neuropathy and painful symptoms. PLoS One. 2013:e80915.

402. Micheli L, Di Cesare Mannelli L, Rizzi A, Guerrini R, Trapella C, Calo G, et al. Intrathecal administration of nociceptin/orphanin FQ receptor agonists in rats: A strategy to relieve chemotherapy-induced neuropathic hypersensitivity. Eur J Pharmacol. 2015:155-62.

403. Michot B, Kayser V, Bastian G, Bourgoin S, Hamon M. Differential pharmacological alleviation of oxaliplatin-induced hyperalgesia/allodynia at cephalic versus extra-cephalic level in rodents. Neuropharmacology. 2014:432-43.

404. Mihara Y, Egashira N, Sada H, Kawashiri T, Ushio S, Yano T, et al. Involvement of spinal NR2B-containing NMDA receptors in oxaliplatin-induced mechanical allodynia in rats. Molecular Pain. 2011.

405. Mizoguchi S, Andoh T, Yakura T, Kuraishi Y. Involvement of c-Myc-mediated transient receptor potential melastatin 8 expression in oxaliplatin-induced cold allodynia in mice. Pharmacological Reports. 2016:645-8.

406. Mizuno K, Kono T, Suzuki Y, Miyagi C, Omiya Y, Miyano K, et al. Goshajinkigan, a traditional Japanese medicine, prevents oxaliplatin-induced acute peripheral neuropathy by suppressing functional alteration of TRP channels in rat. Journal of Pharmacological Sciences. 2014:91-8.

407. Mizuno K, Shibata K, Komatsu R, Omiya Y, Kase Y, Koizumi S. An effective therapeutic approach for oxaliplatin-induced peripheral neuropathy using a combination therapy with goshajinkigan and bushi. Cancer Biology and Therapy. 2016(11):1206-12.

408. Mo M, Erdelyi I, Szigeti-Buck K, Benbow JH, Ehrlich BE. Prevention of paclitaxel-induced peripheral neuropathy by lithium pretreatment. FASEB Journal. 2012:4696-709.

409. Moon HJ, Lim BS, Lee DI, Ye MS, Lee G, Min BI, et al. Effects of electroacupuncture on oxaliplatin-induced neuropathic cold hypersensitivity in rats. J Physiol Sci. 2014:151-6.

410. Moreira DRM, Santos DS, Espirito Santo RFD, Santos FED, de Oliveira Filho GB, Leite ACL, et al. Structural improvement of new thiazolidinones compounds with antinociceptive activity in experimental chemotherapy-induced painful neuropathy. Chem Biol Drug Des. 2017(2):297-307.

411. Mori T, Kanbara T, Harumiya M, Iwase Y, Masumoto A, Komiya S, et al. Establishment of opioid-induced rewarding effects under oxaliplatin- and Paclitaxel-induced neuropathy in rats. J Pharmacol Sci. 2014:47-55.

412. Muller LJ, Gerritsen Van Der Hoop R, Moorer-Van Delft CM, Gispen WH, Roubos EW. Morphological and electrophysiological study of the effects of cisplatin and ORG.2766 on rat spinal ganglion neurons. Cancer Research. 1990:2437-42.

413. Mustafa G, Anderson EM, Bokrand-Donatelli Y, Neubert JK, Caudle RM. Anti-nociceptive effect of a conjugate of substance P and light chain of botulinum neurotoxin type A. Pain. 2013:2547-53.

414. Muthuraman A, Singh N. Attenuating effect of hydroalcoholic extract of Acorus calamus in vincristine-induced painful neuropathy in rats. J Nat Med. 2011:480-7.

415. Muthuraman A, Singh N, Jaggi AS. Protective effect of Acorus calamus L. in rat model of vincristine induced painful neuropathy: an evidence of anti-inflammatory and anti-oxidative activity. Food Chem Toxicol. 2011:2557-63.

416. Naguib M, Xu JJ, Diaz P, Brown DL, Cogdell D, Bie B, et al. Prevention of paclitaxel-induced neuropathy through activation of the central cannabinoid type 2 receptor system. Anesthesia and Analgesia. 2012:1104-20.

417. Nakahashi Y, Kamiya Y, Funakoshi K, Miyazaki T, Uchimoto K, Tojo K, et al. Role of nerve growth factor-tyrosine kinase receptor A signaling in paclitaxel-induced peripheral neuropathy in rats. Biochem Biophys Res Commun. 2014:415-9.

418. Namvaran-Abbas-Abad A, Tavakkoli F. Antinociceptive effect of Salvia extract on cisplatin-induced hyperalgesia in mice. Neurophysiology. 2012:452-8.

419. Nashawi H, Masocha W, Edafiogho IO, Kombian SB. Paclitaxel Causes Electrophysiological Changes in the Anterior Cingulate Cortex via Modulation of the gamma-Aminobutyric Acid-ergic System. Med Princ Pract. 2016(5):423-8.

420. Nassini R, Gees M, Harrison S, De Siena G, Materazzi S, Moretto N, et al. Oxaliplatin elicits mechanical and cold allodynia in rodents via TRPA1 receptor stimulation. Pain. 2011:1621-31.

421. Nativi C, Gualdani R, Dragoni E, Di Cesare Mannelli L, Sostegni S, Norcini M, et al. A TRPA1 antagonist reverts oxaliplatin-induced neuropathic pain. Sci Rep. 2013:2005.

422. Nayebi AM, Sharifi H, Ramadzani M, Rezazadeh H. Effect of acute and chronic administration of carbamazepine on Cisplatin-induced hyperalgesia in rats. Jundishapur J Nat Pharm Prod. 2012:27-30.

423. Neelakantan H, Ward SJ, Walker EA. Effects of paclitaxel on mechanical sensitivity and morphine reward in male and female C57Bl6 mice. Experimental and Clinical Psychopharmacology. 2016(6):485-95.

424. Neuman ISA, Heijmen PS, Peters RC, Ruigt GSF. Fish electroreception as a model for vincristine-induced neuropathies and a possible preventive role for ORG 2766 treatment. Comparative Biochemistry and Physiology - C Pharmacology Toxicology and Endocrinology. 1993:165-73.

425. Nie B, Liu C, Bai X, Chen X, Wu S, Zhang S, et al. AKAP150 involved in paclitaxel-induced neuropathic pain via inhibiting CN/NFAT2 pathway and downregulating IL-4. Brain Behav Immun. 2017.

426. Nie B, Zhang S, Huang Z, Huang J, Chen X, Zheng Y, et al. Synergistic Interaction Between Dexmedetomidine and Ulinastatin Against Vincristine-Induced Neuropathic Pain in Rats. J Pain. 2017(11):1354-64.

427. Nie J, Liu X. Leonurine attenuates hyperalgesia in mice with induced adenomyosis. Medical Science Monitor. 2017:1701-6.

428. Nie J, Liu X. Quercetin alleviates generalized hyperalgesia in mice with induced adenomyosis. Molecular Medicine Reports. 2017(4):5370-6.

429. Nieto FR, Cendan CM, Canizares FJ, Cubero MA, Vela JM, Fernandez-Segura E, et al. Genetic inactivation and pharmacological blockade of sigma-1 receptors prevent paclitaxel-induced sensory-nerve mitochondrial abnormalities and neuropathic pain in mice. Mol Pain. 2014:11.

430. Nieto FR, Entrena JM, Cendan CM, Pozo ED, Vela JM, Baeyens JM. Tetrodotoxin inhibits the development and expression of neuropathic pain induced by paclitaxel in mice. Pain. 2008:520-31.

431. Nishida K, Kuchiiwa S, Oiso S, Futagawa T, Masuda S, Takeda Y, et al. Up-regulation of matrix metalloproteinase-3 in the dorsal root ganglion of rats with paclitaxel-induced neuropathy. Cancer Sci. 2008:1618-25.

432. Nishida T, Tsubota M, Kawaishi Y, Yamanishi H, Kamitani N, Sekiguchi F, et al. Involvement of high mobility group box 1 in the development and maintenance of chemotherapy-induced peripheral neuropathy in rats. Toxicology. 2016:48-58.

433. Noda K, Akita H, Ogata M, Saji M. Paclitaxel-induced hyperalgesia modulates negative affective component of pain and NR1 receptor expression in the frontal cortex in rats. Neuroscience Research. 2014:32-7.

434. Nodera H, Spieker A, Sung M, Rutkove S. Neuroprotective effects of Kv7 channel agonist, retigabine, for cisplatin-induced peripheral neuropathy. Neuroscience Letters. 2011:223-7.

435. Norcini M, Vivoli E, Galeotti N, Bianchi E, Bartolini A, Ghelardini C. Supraspinal role of protein kinase C in oxaliplatin-induced neuropathy in rat. Pain. 2009:141-7.

436. Nozaki-Taguchi N, Chaplan SR, Higuera ES, Ajakwe RC, Yaksh TL. Vincristine-induced allodynia in the rat. Pain. 2001:69-76.

437. Ochi-ishi R, Nagata K, Inoue T, Tozaki-Saitoh H, Tsuda M, Inoue K. Involvement of the chemokine CCL3 and the purinoceptor P2X7 in the spinal cord in paclitaxel-induced mechanical allodynia. Mol Pain. 2014:53.

438. Ogawa T, Mimura Y, Isowa K, Kato H, Mitsuishi M, Toyoshi T, et al. An antimicrotubule agent, TZT-1027, does not induce neuropathologic alterations which are detected after administration of vincristine or paclitaxel in animal models. Toxicol Lett. 2001:97-106.

439. Ohsawa M, Otake S, Murakami T, Yamamoto S, Makino T, Ono H. Gabapentin prevents oxaliplatin-induced mechanical hyperalgesia in mice. Journal of Pharmacological Sciences. 2014:292-9.

440. Okubo K, Takahashi T, Sekiguchi F, Kanaoka D, Matsunami M, Ohkubo T, et al. Inhibition of T-type calcium channels and hydrogen sulfide-forming enzyme reverses paclitaxel-evoked neuropathic hyperalgesia in rats. Neuroscience. 2011:148-56.

441. Old EA, Nadkarni S, Grist J, Gentry C, Bevan S, Kim KW, et al. Monocytes expressing CX3CR1 orchestrate the development of vincristine-induced pain. J Clin Invest. 2014:2023-36.

442. Orhan B, Yalcin S, Nurlu G, Zeybek D, Muftuoglu S. Erythropoietin against cisplatin-induced peripheral neurotoxicity in rats. Med Oncol. 2004:197-203.

443. Ozturk G, Anlar O, Erdogan E, Kosem M, Ozbek H, Turker A. The effect of Ginkgo extract EGb761 in cisplatin-induced peripheral neuropathy in mice. Toxicology and Applied Pharmacology. 2004:169-75.

444. Ozturk G, Erdogan E, Anlar O, Kosem M, Taspinar M. Effect of leukemia inhibitory factor in experimental cisplatin neuropathy in mice. Cytokine. 2005:31-41.

445. Pacini A, Micheli L, Maresca M, Branca JJ, McIntosh JM, Ghelardini C, et al. The alpha9alpha10 nicotinic receptor antagonist alpha-conotoxin RgIA prevents neuropathic pain induced by oxaliplatin treatment. Exp Neurol. 2016.

446. Park BY, Park SH, Kim WM, Yoon MH, Lee HG. Antinociceptive Effect of Memantine and Morphine on Vincristine-induced Peripheral Neuropathy in Rats. Korean J Pain. 2010:179-85.

447. Park HJ, Kim YH, Koh HJ, Park CS, Kang SH, Choi JH, et al. Analgesic effects of dexmedetomidine in vincristine-evoked painful neuropathic rats. Journal of Korean Medical Science. 2012:1411-7.

448. Park HJ, Lee HG, Kim YS, Lee JY, Jeon JP, Park C, et al. Ginkgo biloba extract attenuates hyperalgesia in a rat model of vincristine-induced peripheral neuropathy. Anesth Analg. 2012:1228-33.

449. Park HJ, Marino MJ, Rondon ES, Xu Q, Yaksh TL. The effects of intraplantar and intrathecal botulinum toxin type B on tactile allodynia in mono and polyneuropathy in the mouse. Anesth Analg. 2015:229-38.

450. Park HJ, Stokes JA, Corr M, Yaksh TL. Toll-like receptor signaling regulates cisplatin-induced mechanical allodynia in mice. Cancer Chemother Pharmacol. 2014:25-34.

451. Park HJ, Stokes JA, Pirie E, Skahen J, Shtaerman Y, Yaksh TL. Persistent hyperalgesia in the cisplatin-treated mouse as defined by threshold measures, the conditioned place preference paradigm, and changes in dorsal root ganglia activated transcription factor 3: the effects of gabapentin, ketorolac, and etanercept. Anesth Analg. 2013:224-31.

452. Park JH, Chae J, Roh K, Kil EJ, Lee M, Auh CK, et al. Oxaliplatin-Induced Peripheral Neuropathy via TRPA1 Stimulation in Mice Dorsal Root Ganglion Is Correlated with Aluminum Accumulation. PLoS One. 2015:e0124875.

453. Park JS, Kim S, Hoke A. An exercise regimen prevents development paclitaxel induced peripheral neuropathy in a mouse model. Journal of the Peripheral Nervous System. 2015:7-14.

454. Parvathy SS, Masocha W. Matrix metalloproteinase inhibitor COL-3 prevents the development of paclitaxel-induced hyperalgesia in mice. Medical Principles and Practice. 2013:35-41.

455. Parvathy SS, Masocha W. Coadministration of indomethacin and minocycline attenuates established paclitaxel-induced neuropathic thermal hyperalgesia: Involvement of cannabinoid CB1 receptors. Scientific reports. 2015:10541.

456. Pascual D, Goicoechea C, Burgos E, Martin MI. Antinociceptive effect of three common analgesic drugs on peripheral neuropathy induced by paclitaxel in rats. Pharmacol Biochem Behav. 2010:331-7.

457. Pascual D, Goicoechea C, Suardiaz M, Martin MI. A cannabinoid agonist, WIN 55,212-2, reduces neuropathic nociception induced by paclitaxel in rats. Pain. 2005:23-34.

458. Paton KF, Kumar N, Crowley RS, Harper JL, Prisinzano TE, Kivell BM. The analgesic and anti-inflammatory effects of Salvinorin A analogue beta-tetrahydropyran Salvinorin B in mice. Eur J Pain. 2017(6):1039-50.

459. Peng P, Xi Q, Xia S, Zhuang L, Gui Q, Chen Y, et al. Pregabalin attenuates docetaxel-induced neuropathy in rats. Journal of Huazhong University of Science and Technology - Medical Science. 2012:586-90.

460. Persohn E, Canta A, Schoepfer S, Traebert M, Mueller L, Gilardini A, et al. Morphological and morphometric analysis of paclitaxel and docetaxel-induced peripheral neuropathy in rats. Eur J Cancer. 2005:1460-6.

461. Peters CM, Jimenez-Andrade JM, Jonas BM, Sevcik MA, Koewler NJ, Ghilardi JR, et al. Intravenous paclitaxel administration in the rat induces a peripheral sensory neuropathy characterized by macrophage infiltration and injury to sensory neurons and their supporting cells. Experimental Neurology. 2007:42-54.

462. Petrini M, Vaglini F, Cervetti G, Cavalletti M, Sartucci F, Murri L, et al. Is lithium able to reverse neurological damage induced by vinca alkaloids? Journal of Neural Transmission. 1999:569-75.

463. Pevida M, Lastra A, Hidalgo A, Baamonde A, Menendez L. Spinal CCL2 and microglial activation are involved in paclitaxel-evoked cold hyperalgesia. Brain Res Bull. 2013:21-7.

464. Pisano C, Pratesi G, Laccabue D, Zunino F, Lo Giudice P, Bellucci A, et al. Paclitaxel and Cisplatin-induced neurotoxicity: a protective role of acetyl-L-carnitine. Clin Cancer Res. 2003:5756-67.

465. Podratz JL, Lee H, Knorr P, Koehler S, Forsythe S, Lambrecht K, et al. Cisplatin induces mitochondrial deficits in Drosophila larval segmental nerve. Neurobiol Dis. 2017;97(Pt A):60-9.

466. Podratz JL, Staff NP, Boesche JB, Giorno NJ, Hainy ME, Herring SA, et al. An automated climbing apparatus to measure chemotherapy-induced neurotoxicity in Drosophila melanogaster. Fly (Austin). 2013:187-92.

467. Podratz JL, Staff NP, Froemel D, Wallner A, Wabnig F, Bieber AJ, et al. Drosophila melanogaster: a new model to study cisplatin-induced neurotoxicity. Neurobiol Dis. 2011:330-7.

468. Polomano RC, Mannes AJ, Clark US, Bennett GJ. A painful peripheral neuropathy in the rat produced by the chemotherapeutic drug, paclitaxel. Pain. 2001:293-304.

469. Ponsati B, Carreno C, Curto-Reyes V, Valenzuela B, Duart MJ, Van den Nest W, et al. An inhibitor of neuronal exocytosis (DD04107) displays long-lasting in vivo activity against chronic inflammatory and neuropathic pain. J Pharmacol Exp Ther. 2012:634-45.

470. Pourmohammadi N, Alimoradi H, Mehr SE, Hassanzadeh G, Hadian MR, Sharifzadeh M, et al. Lithium Attenuates Peripheral Neuropathy Induced by Paclitaxel in Rats. Basic and Clinical Pharmacology and Toxicology. 2012:231-7.

471. Pradat PF, Finiels F, Kennel P, Naimi S, Orsini C, Delaere P, et al. Partial prevention of cisplatin-induced neuropathy by electroporation-mediated nonviral gene transfer. Human Gene Therapy. 2001:367-75.

472. Rahn EJ, Deng L, Thakur GA, Vemuri K, Zvonok AM, Lai YY, et al. Prophylactic cannabinoid administration blocks the development of paclitaxel-induced neuropathic nociception during analgesic treatment and following cessation of drug delivery. Mol Pain. 2014:27.

473. Rahn EJ, Makriyannis A, Hohmann AG. Activation of cannabinoid CB1 and CB2 receptors suppresses neuropathic nociception evoked by the chemotherapeutic agent vincristine in rats. Br J Pharmacol. 2007:765-77.

474. Rahn EJ, Zvonok AM, Thakur GA, Khanolkar AD, Makriyannis A, Hohmann AG. Selective activation of cannabinoid CB2 receptors suppresses neuropathic nociception induced by treatment with the chemotherapeutic agent paclitaxel in rats. J Pharmacol Exp Ther. 2008:584-91.

475. Renn CL, Carozzi VA, Rhee P, Gallop D, Dorsey SG, Cavaletti G. Multimodal assessment of painful peripheral neuropathy induced by chronic oxaliplatin-based chemotherapy in mice. Mol Pain. 2011:29.

476. Rigo FK, Dalmolin GD, Trevisan G, Tonello R, Silva MA, Rossato MF, et al. Effect of omega-conotoxin MVIIA and Phalpha1beta on paclitaxel-induced acute and chronic pain. Pharmacol Biochem Behav. 2013:16-22.

477. Robinson CR, Dougherty PM. Spinal astrocyte gap junction and glutamate transporter expression contributes to a rat model of bortezomib-induced peripheral neuropathy. Neuroscience. 2015:1-10.

478. Robinson CR, Zhang H, Dougherty PM. Altered discharges of spinal neurons parallel the behavioral phenotype shown by rats with bortezomib related chemotherapy induced peripheral neuropathy. Brain Research. 2014:6-13.

479. Robinson CR, Zhang H, Dougherty PM. Astrocytes, but not microglia, are activated in oxaliplatin and bortezomib-induced peripheral neuropathy in the rat. Neuroscience. 2014:308-17.

480. Rodriguez-Menendez V, Gilardini A, Bossi M, Canta A, Oggioni N, Carozzi V, et al. Valproate protective effects on cisplatin-induced peripheral neuropathy: an in vitro and in vivo study. Anticancer Res. 2008:335-42.

481. Roglio I, Bianchi R, Camozzi F, Carozzi V, Cervellini I, Crippa D, et al. Docetaxel-induced peripheral neuropathy: protective effects of dihydroprogesterone and progesterone in an experimental model. J Peripher Nerv Syst. 2009:36-44.

482. Romero HK, Christensen SB, Di Cesare Mannelli L, Gajewiak J, Ramachandra R, Elmslie KS, et al. Inhibition of alpha9alpha10 nicotinic acetylcholine receptors prevents chemotherapy-induced neuropathic pain. Proc Natl Acad Sci U S A. 2017(10):E1825-e32.

483. Ruiz-Medina J, Baulies A, Bura SA, Valverde O. Paclitaxel-induced neuropathic pain is age dependent and devolves on glial response. Eur J Pain. 2013:75-85.

484. Russell JW, Gill JS, Sorenson EJ, Schultz DA, Windebank AJ. Suramin-induced neuropathy in an animal model. J Neurol Sci. 2001:71-80.

485. Ruyang T, Yang Z, Wei F. Gabapentin prevents oxaliplatin-induced central sensitization in the dorsal horn neurons in rats. Iran J Basic Med Sci. 2015:493-8.

486. Sada H, Egashira N, Ushio S, Kawashiri T, Shirahama M, Oishi R. Repeated administration of amitriptyline reduces oxaliplatin-induced mechanical allodynia in rats. Journal of Pharmacological Sciences. 2012:547-51.

487. Saha L, Hota D, Chakrabarti A. Evaluation of lercanidipine in Paclitaxel-induced neuropathic pain model in rat: a preliminary study. Pain Res Treat. 2012:143579.

488. Saika F, Kiguchi N, Kobayashi Y, Fukazawa Y, Maeda T, Ozaki M, et al. Suppressive effect of imipramine on vincristine-induced mechanical allodynia in mice. Biol Pharm Bull. 2009:1231-4.

489. Sakamoto A, Andoh T, Kuraishi Y. Involvement of mast cells and proteinase-activated receptor 2 in oxaliplatin-induced mechanical allodynia in mice. Pharmacol Res. 2016:84-92.

490. Sakurai M, Egashira N, Kawashiri T, Yano T, Ikesue H, Oishi R. Oxaliplatin-induced neuropathy in the rat: involvement of oxalate in cold hyperalgesia but not mechanical allodynia. Pain. 2009:165-74.

491. Salat K, Cios A, Wyska E, Salat R, Mogilski S, Filipek B, et al. Antiallodynic and antihyperalgesic activity of 3-[4-(3-trifluoromethyl-phenyl)-piperazin-1-yl]-dihydrofuran-2-one compared to pregabalin in chemotherapy-induced neuropathic pain in mice. Pharmacol Biochem Behav. 2014:173-81.

492. Salat K, Furgala A, Salat R. Evaluation of cebranopadol, a dually acting nociceptin/orphanin FQ and opioid receptor agonist in mouse models of acute, tonic, and chemotherapy-induced neuropathic pain. Inflammopharmacology. 2017.

493. Salat K, Kolaczkowski M, Furgala A, Rojek A, Sniecikowska J, Varney MA, et al. Antinociceptive, antiallodynic and antihyperalgesic effects of the 5-HT1A receptor selective agonist, NLX-112 in mouse models of pain. Neuropharmacology. 2017:181-8.

494. Samineni VK, Premkumar LS, Faingold CL. Neuropathic pain-induced enhancement of spontaneous and pain-evoked neuronal activity in the periaqueductal gray that is attenuated by gabapentin. Pain. 2017(7):1241-53.

495. Sanna MD, Ghelardini C, Galeotti N. St. John's Wort Potentiates anti-Nociceptive Effects of Morphine in Mice Models of Neuropathic Pain. Pain Med. 2017;18(7):1334-43.

496. Schappacher KA, Styczynski L, Baccei ML. Early life vincristine exposure evokes mechanical pain hypersensitivity in the developing rat. Pain. 2017(9):1647-55.

497. Schellingerhout D, LeRoux LG, Hobbs BP, Bredow S. Impairment of retrograde neuronal transport in oxaliplatin-induced neuropathy demonstrated by molecular imaging. PLoS One. 2012:e45776.

498. Schwingel TE, Klein CP, Nicoletti NF, Dora CL, Hadrich G, Bica CG, et al. Effects of the compounds resveratrol, rutin, quercetin, and quercetin nanoemulsion on oxaliplatin-induced hepatotoxicity and neurotoxicity in mice. Naunyn Schmiedebergs Arch Pharmacol. 2014:837-48.

499. Screnci D, McKeage MJ, Galettis P, Hambley TW, Palmer BD, Baguley BC. Relationships between hydrophobicity, reactivity, accumulation and peripheral nerve toxicity of a series of platinum drugs. Br J Cancer. 2000:966-72.

500. Segat GC, Manjavachi MN, Matias DO, Passos GF, Freitas CS, Costa R, et al. Antiallodynic effect of beta-caryophyllene on paclitaxel-induced peripheral neuropathy in mice. Neuropharmacology. 2017:207-19.

501. Seto Y, Okazaki F, Horikawa K, Zhang J, Sasaki H, To H. Influence of dosing times on cisplatin-induced peripheral neuropathy in rats. BMC Cancer. 2016(756).

502. Seto Y, Takase M, Tsuji Y, To H. Pregabalin reduces cisplatin-induced mechanical allodynia in rats. J Pharmacol Sci. 2017(3):175-80.

503. Shabani M, Nazeri M, Parsania S, Razavinasab M, Zangiabadi N, Esmaeilpour K, et al. Walnut consumption protects rats against cisplatin-induced neurotoxicity. Neurotoxicology. 2012:1314-21.

504. Shahid M, Subhan F, Ahmad N, Sewell RDE. The flavonoid 6-methoxyflavone allays cisplatin-induced neuropathic allodynia and hypoalgesia. Biomed Pharmacother. 2017:1725-33.

505. Sharawy N, Rashed L, Youakim MF. Evaluation of multi-neuroprotective effects of erythropoietin using cisplatin induced peripheral neurotoxicity model. Exp Toxicol Pathol. 2015:315-22.

506. Shen Y, Zhang ZJ, Zhu MD, Jiang BC, Yang T, Gao YJ. Exogenous induction of HO-1 alleviates vincristine-induced neuropathic pain by reducing spinal glial activation in mice. Neurobiol Dis. 2015:100-10.

507. Shidahara Y, Ogawa S, Nakamura M, Nemoto S, Awaga Y, Takashima M, et al. Pharmacological comparison of a nonhuman primate and a rat model of oxaliplatin-induced neuropathic cold hypersensitivity. Pharmacology Research and Perspectives. 2016:1-11.

508. Shimizu H, Ohgoh M, Momose Y, Nishizawa Y, Ogura H. Massive cell death of cerebellar granule neurons accompanied with caspase-3-like protease activation and subsequent motor discoordination after intracerebroventricular injection of vincristine in mice. Neuroscience. 2002:55-65.

509. Shin YK, Jang SY, Lee HK, Jung J, Suh DJ, Seo SY, et al. Pathological adaptive responses of schwann cells to endoplasmic reticulum stress in bortezomib-induced peripheral neuropathy. Glia. 2010:1961-76.

510. Shirahama M, Ushio S, Egashira N, Yamamoto S, Sada H, Masuguchi K, et al. Inhibition of Ca2+/Calmodulin-dependent protein kinase II reverses oxaliplatin-induced mechanical allodynia in Rats. Molecular Pain. 2012.

511. Siau C, Bennett GJ. Dysregulation of cellular calcium homeostasis in chemotherapy-evoked painful peripheral neuropathy. Anesth Analg. 2006:1485-90.

512. Siau C, Xiao W, Bennett GJ. Paclitaxel- and vincristine-evoked painful peripheral neuropathies: loss of epidermal innervation and activation of Langerhans cells. Exp Neurol. 2006:507-14.

513. Sisignano M, Angioni C, Park CK, Meyer Dos Santos S, Jordan H, Kuzikov M, et al. Targeting CYP2J to reduce paclitaxel-induced peripheral neuropathic pain. Proc Natl Acad Sci U S A. 2016(44):12544-9.

514. Slivicki RA, Ali YO, Lu HC, Hohmann AG. Impact of Genetic Reduction of NMNAT2 on Chemotherapy-Induced Losses in Cell Viability In Vitro and Peripheral Neuropathy In Vivo. PloS one. 2016(1):e0147620.

515. Smith SB, Crager SE, Mogil JS. Paclitaxel-induced neuropathic hypersensitivity in mice: responses in 10 inbred mouse strains. Life Sci. 2004:2593-604.

516. Sprowl JA, Ciarimboli G, Lancaster CS, Giovinazzo H, Gibson AA, Du G, et al. Oxaliplatin-induced neurotoxicity is dependent on the organic cation transporter OCT2. Proc Natl Acad Sci U S A. 2013:11199-204.

517. Sui M, Lessans S, Yan T, Cao D, Lao L, Dorsey SG. Mechanism of electroacupuncture on Zusanli (ST 36) for chemotherapy-induced peripheral neuropathy. Zhongguo zhen jiu = Chinese acupuncture & moxibustion. 2016(5):512-6.

518. Suzuki T, Miyamoto K, Yokoyama N, Sugi M, Kagioka A, Kitao Y, et al. Processed aconite root and its active ingredient neoline may alleviate oxaliplatin-induced peripheral neuropathic pain. J Ethnopharmacol. 2016.

519. Suzuki T, Yamamoto A, Ohsawa M, Motoo Y, Mizukami H, Makino T. Effect of ninjin'yoeito and ginseng extracts on oxaliplatin-induced neuropathies in mice. J Nat Med. 2017(4):757-64.

520. Sweitzer SM, Pahl JL, DeLeo JA. Propentofylline attenuates vincristine-induced peripheral neuropathy in the rat. Neuroscience Letters. 2006:258-61.

521. Szilvassy J, Sziklai I, Racz T, Horvath P, Rabloczky G, Szilvassy Z. Impaired bronchomotor responses to field stimulation in guinea-pigs with cisplatin-induced neuropathy. European Journal of Pharmacology. 2000:259-65.

522. Ta LE, Bieber AJ, Carlton SM, Loprinzi CL, Low PA, Windebank AJ. Transient Receptor Potential Vanilloid 1 is essential for cisplatin-induced heat hyperalgesia in mice. Molecular Pain. 2010.

523. Ta LE, Low PA, Windebank AJ. Mice with cisplatin and oxaliplatin-induced painful neuropathy develop distinct early responses to thermal stimuli. Molecular Pain. 2009.

524. Ta LE, Schmelzer JD, Bieber AJ, Loprinzi CL, Sieck GC, Brederson JD, et al. A novel and selective poly (ADP-ribose) polymerase inhibitor ameliorates chemotherapy-induced painful neuropathy. PLoS One. 2013:e54161.

525. Taleb O, Bouzobra F, Tekin-Pala H, Meyer L, Mensah-Nyagan AG, Patte-Mensah C. Behavioral and electromyographic assessment of oxaliplatin-induced motor dysfunctions: Evidence for a therapeutic effect of allopregnanolone. Behavioural Brain Research. 2016.

526. Tanner KD, Levine JD, Topp KS. Microtubule disorientation and axonal swelling in unmyelinated sensory axons during vincristine-induced painful neuropathy in rat. J Comp Neurol. 1998:481-92.

527. Tanner KD, Reichling DB, Gear RW, Paul SM, Levine JD. Altered temporal pattern of evoked afferent activity in a rat model of vincristine-induced painful peripheral neuropathy. Neuroscience. 2003:809-17.

528. Tanner KD, Reichling DB, Levine JD. Nociceptor hyper-responsiveness during vincristine-induced painful peripheral neuropathy in the rat. J Neurosci. 1998:6480-91.

529. Tasnim A, Rammelkamp Z, Slusher AB, Wozniak K, Slusher BS, Farah MH. Paclitaxel causes degeneration of both central and peripheral axon branches of dorsal root ganglia in mice. BMC Neurosci. 2016;17(1):47.

530. Tassler P, Dellon AL, Lesser GJ, Grossman S. Utility of decompressive surgery in the prophylaxis and treatment of cisplatin neuropathy in adult rats. Journal of Reconstructive Microsurgery. 2000:457-63.

531. Tatsushima Y, Egashira N, Kawashiri T, Mihara Y, Yano T, Mishima K, et al. Involvement of substance P in peripheral neuropathy induced by paclitaxel but not oxaliplatin. Journal of Pharmacology and Experimental Therapeutics. 2011:226-35.

532. Tenci B, Di Cesare Mannelli L, Maresca M, Micheli L, Pieraccini G, Mulinacci N, et al. Effects of a water extract of Lepidium meyenii root in different models of persistent pain in rats. Zeitschrift fur Naturforschung C, Journal of biosciences. 2017;72(11-12):449-57.

533. Teo S. Lack of peripheral neuropathy in Beagle dogs after 53 weeks oral administration of thalidomide capsules. Human and Experimental Toxicology. 2000:615-22.

534. Ter Laak MP, Hamers FPT, Kirk CJ, Gispen WH. rhGGF2 protects against cisplatin-induced neuropathy in the rat. Journal of Neuroscience Research. 2000:237-44.

535. Thacheril Mohanan A, Venkatesan S, Sermugapandian N, Al-Safhi M, Khan G. Attenuating effect of Cilostazol against vincristine - Induced neuropathic pain in mice. Journal of Pharmacy Research. 2013:579-82.

536. Thangamani D, Edafiogho IO, Masocha W. The anticonvulsant enaminone E139 attenuates paclitaxel-induced neuropathic pain in rodents. ScientificWorldJournal. 2013:240508.

537. Thiagarajan VR, Shanmugam P, Krishnan UM, Muthuraman A. Ameliorative effect of Vernonia cinerea in vincristine-induced painful neuropathy in rats. Toxicol Ind Health. 2014:794-805.

538. Thiagarajan VR, Shanmugam P, Krishnan UM, Muthuraman A, Singh N. Antinociceptive effect of Butea monosperma on vincristine-induced neuropathic pain model in rats. Toxicol Ind Health. 2013:3-13.

539. Thibault K, Calvino B, Rivals I, Marchand F, Dubacq S, McMahon SB, et al. Molecular mechanisms underlying the enhanced analgesic effect of oxycodone compared to morphine in chemotherapy-induced neuropathic pain. PLoS One. 2014:e91297.

540. Thibault K, Elisabeth B, Sophie D, Claude FZ, Bernard R, Bernard C. Antinociceptive and anti-allodynic effects of oral PL37, a complete inhibitor of enkephalin-catabolizing enzymes, in a rat model of peripheral neuropathic pain induced by vincristine. Eur J Pharmacol. 2008:71-7.

541. Thibault K, Rivals I, Dahoma S, Dubacq S, Pezet S, Calvino B. Structural and molecular alterations of primary afferent fibres in the spinal dorsal horn in vincristine-induced neuropathy in rat. J Mol Neurosci. 2013:880-92.

542. Tian L, Fan T, Zhou N, Guo H, Zhang W. Role of PAR2 in regulating oxaliplatin-induced neuropathic pain via TRPA1. Translational Neuroscience. 2015:111-6.

543. Todd GC, Griffing WJ, Gibson WR, Morton DM. Animal models for the comparative assessment of neurotoxicity following repeated administration of vinca alkaloids. Cancer Treat Rep. 1979:35-41.

544. Toma W, Kyte SL, Bagdas D, Alkhlaif Y, Alsharari SD, Lichtman AH, et al. Effects of paclitaxel on the development of neuropathy and affective behaviors in the mouse. Neuropharmacology. 2017:305-15.

545. Tomiwa K, Nolan C, Cavanagh JB. The effects of cisplatin on rat spinal ganglia: a study by light and electron microscopy and by morphometry. Acta Neuropathologica. 1986:295-308.

546. Tomohisa M, Junpei O, Aki M, Masato H, Mika F, Kazumi Y, et al. Possible involvement of the Sigma-1 receptor chaperone in chemotherapeutic-induced neuropathic pain. Synapse. 2015:526-32.

547. Tonello R, Fusi C, Materazzi S, Marone IM, De Logu F, Benemei S, et al. The peptide Phalpha1beta, from spider venom, acts as a TRPA1 channel antagonist with antinociceptive effects in mice. Br J Pharmacol. 2017(1):57-69.

548. Topp KS, Tanner KD, Levine JD. Damage to the cytoskeleton of large diameter sensory neurons and myelinated axons in vincristine-induced painful peripheral neuropathy in the rat. Journal of Comparative Neurology. 2000:563-76.

549. Toyama S, Shimoyama N, Ishida Y, Koyasu T, Szeto HH, Shimoyama M. Characterization of acute and chronic neuropathies induced by oxaliplatin in mice and differential effects of a novel mitochondria-targeted antioxidant on the neuropathies. Anesthesiology. 2014:459-73.

550. Toyama S, Shimoyama N, Shimoyama M. The analgesic effect of orexin-A in a murine model of chemotherapy-induced neuropathic pain. Neuropeptides. 2017:95-100.

551. Tredici G, Braga M, Nicolini G, Miloso M, Marmiroli P, Schenone A, et al. Effect of recombinant human nerve growth factor on cisplatin neurotoxicity in rats. Experimental Neurology. 1999:551-8.

552. Tredici G, Cavaletti G, Petruccioli MG, Fabbrica D, Tedeschi M, Venturino P. Low-dose glutathione administration in the prevention of cisplatin- induced peripheral neuropathy in rats. NeuroToxicology. 1994:701-4.

553. Tsubaki M, Takeda T, Tani T, Shimaoka H, Suzuyama N, Sakamoto K, et al. PKC/MEK inhibitors suppress oxaliplatin-induced neuropathy and potentiate the antitumor effects. International Journal of Cancer. 2015:243-50.

554. Tuncer S, Dalkilic N, Akif Dunbar M, Keles B. Comparative effects of alpha lipoic acid and melatonin on cisplatin-induced neurotoxicity. Int J Neurosci. 2010:655-63.

555. Turkiew E, Falconer D, Reed N, Hoke A. Deletion of Sarm1 gene is neuroprotective in two models of peripheral neuropathy. J Peripher Nerv Syst. 2017;22(3):162-71.

556. Uceyler N, Kobsar I, Biko L, Ulzheimer J, Levinson SR, Martini R, et al. Heterozygous P0 deficiency protects mice from vincristine-induced polyneuropathy. J Neurosci Res. 2006:37-46.

557. Uchida H, Nagai J, Ueda H. Lysophosphatidic acid and its receptors LPA1 and LPA3 mediate paclitaxel-induced neuropathic pain in mice. Mol Pain. 2014:71.

558. Uchino H, Matsumura Y, Negishi T, Koizumi F, Hayashi T, Honda T, et al. Cisplatin-incorporating polymeric micelles (NC-6004) can reduce nephrotoxicity and neurotoxicity of cisplatin in rats. Br J Cancer. 2005:678-87.

559. Uhelski ML, Khasabova IA, Simone DA. Inhibition of anandamide hydrolysis attenuates nociceptor sensitization in a murine model of chemotherapy-induced peripheral neuropathy. J Neurophysiol. 2015:1501-10.

560. Uy QL, Moen TH, Johns RJ, Owens AH, Jr. Vincristine neurotoxicity in rodents. Johns Hopkins Med J. 1967:349-60.

561. Van Helleputte L, Kater M, Cook DP, Eykens C, Rossaert E, Haeck W, et al. Inhibition of histone deacetylase 6 (HDAC6) protects against vincristine-induced peripheral neuropathies and inhibits tumor growth. Neurobiol Dis. 2017.

562. Vashistha B, Sharma A, Jain V. Ameliorative potential of ferulic acid in vincristine-induced painful neuropathy in rats: An evidence of behavioral and biochemical examination. Nutr Neurosci. 2014.

563. Vencappa S, Donaldson LF, Hulse RP. Cisplatin induced sensory neuropathy is prevented by vascular endothelial growth factor-A. Am J Transl Res. 2015:1032-44.

564. Venkata G, Ramalingayya, Cheruku SP, Nayak PG, Kishore A, Shenoy R, et al. Rutin protects against neuronal damage in vitro and ameliorates doxorubicin-induced memory deficits in vivo in Wistar rats. Drug Design, Development and Therapy. 2017:1011-26.

565. Vera G, Cabezos PA, Martin MI, Abalo R. Characterization of cannabinoid-induced relief of neuropathic pain in a rat model of cisplatin-induced neuropathy. Pharmacol Biochem Behav. 2013:205-12.

566. Vera G, Chiarlone A, Cabezos PA, Pascual D, Martin MI, Abalo R. WIN 55,212-2 prevents mechanical allodynia but not alterations in feeding behaviour induced by chronic cisplatin in the rat. Life Sci. 2007:468-79.

567. Verdu E, Vilches JJ, Rodriguez FJ, Ceballos D, Valero A, Navarro X. Physiological and immunohistochemical characterization of cisplatin- induced neuropathy in mice. Muscle and Nerve. 1999:329-40.

568. Verheyen A, Peeraer E, Lambrechts D, Poesen K, Carmeliet P, Shibuya M, et al. Therapeutic potential of VEGF and VEGF-derived peptide in peripheral neuropathies. Neuroscience. 2013:77-89.

569. Verheyen A, Peeraer E, Nuydens R, Dhondt J, Poesen K, Pintelon I, et al. Systemic anti-vascular endothelial growth factor therapies induce a painful sensory neuropathy. Brain. 2012:2629-41.

570. Viana-Cardoso KV, da Silva MT, Junior RC, Peixoto Junior AA, Pinho LG, Santos AA, et al. Repeated cisplatin treatments inhibit gastrointestinal motility and induces baroreflex changes and mechanical hyperalgesia in rats. Cancer Invest. 2011:494-500.

571. Vincent JA, Wieczerzak KB, Gabriel HM, Nardelli P, Rich MM, Cope TC. A novel path to chronic proprioceptive disability with oxaliplatin: Distortion of sensory encoding. Neurobiol Dis. 2016:54-65.

572. Wan CF, Zheng LL, Liu Y, Yu X. Houttuynia cordata Thunb reverses oxaliplatin-induced neuropathic pain in rat by regulating Th17/Treg balance. Am J Transl Res. 2016:1609-14.

573. Wang ML, Yu G, Yi SP, Zhang FY, Wang ZT, Huang B, et al. Antinociceptive effects of incarvillateine, a monoterpene alkaloid from Incarvillea sinensis, and possible involvement of the adenosine system. Sci Rep. 2015:16107.

574. Wang MS, Davis AA, Culver DG, Glass JD. Wld<sup>s</sup> mice are resistant to paclitaxel (Taxol) neuropathy. Annals of Neurology. 2002:442-7.

575. Wang MS, Davis AA, Culver DG, Wang Q, Powers JC, Glass JD. Calpain inhibition protects against Taxol-induced sensory neuropathy. Brain. 2004:671-9.

576. Wang Y, Cao SE, Tian J, Liu G, Zhang X, Li P. Auraptenol attenuates vincristine-induced mechanical hyperalgesia through serotonin 5-HT1A receptors. Sci Rep. 2013:3377.

577. Wang YS, Li YY, Cui W, Li LB, Zhang ZC, Tian BP, et al. Melatonin Attenuates Pain Hypersensitivity and Decreases Astrocyte-Mediated Spinal Neuroinflammation in a Rat Model of Oxaliplatin-Induced Pain. Inflammation. 2017(6):2052-61.

578. Ward SJ, McAllister SD, Kawamura R, Murase R, Neelakantan H, Walker EA. Cannabidiol inhibits paclitaxel-induced neuropathic pain through 5-HT(1A) receptors without diminishing nervous system function or chemotherapy efficacy. Br J Pharmacol. 2014:636-45.

579. Ward SJ, Ramirez MD, Neelakantan H, Walker EA. Cannabidiol prevents the development of cold and mechanical allodynia in paclitaxel-treated female C57Bl6 mice. Anesth Analg. 2011:947-50.

580. Waseem M, Tabassum H, Parvez S. Neuroprotective effects of melatonin as evidenced by abrogation of oxaliplatin induced behavioral alterations, mitochondrial dysfunction and neurotoxicity in rat brain. Mitochondrion. 2016:168-76.

581. Watanabe H, Kawashiri T, Ushio S, Ozawa N, Morita H, Oishi R, et al. Neurotropin relieves oxaliplatin-induced neuropathy via G<inf>i</inf> protein-coupled receptors in the monoaminergic descending pain inhibitory system. Life Sciences. 2014:49-54.

582. Wei JY, Liu CC, Ouyang HD, Ma C, Xie MX, Liu M, et al. Activation of RAGE/STAT3 pathway by methylglyoxal contributes to spinal central sensitization and persistent pain induced by bortezomib. Experimental Neurology. 2017:74-82.

583. Weissman-Fogel I, Dashkovsky A, Rogowski Z, Yarnitsky D. Vagal damage enhances polyneuropathy pain: additive effect of two algogenic mechanisms. Pain. 2008:153-62.

584. Weng HR, Aravindan N, Cata JP, Chen JH, Shaw ADS, Dougherty PM. Spinal glial glutamate transporters downregulate in rats with taxol-induced hyperalgesia. Neuroscience Letters. 2005:18-22.

585. Weng HR, Cordella JV, Dougherty PM. Changes in sensory processing in the spinal dorsal horn accompany vincristine-induced hyperalgesia and allodynia. Pain. 2003:131-8.

586. Whitaker-Azmitia PM, Raio M, Raio D, Borella A. A 5-HT 3 receptor antagonist fails to prevent cisplatin-induced toxicity in

immature rat spinal cord. European Journal of Pharmacology. 1995:139-43.

587. Wilkerson JL, Ghosh S, Bagdas D, Mason BL, Crowe MS, Hsu KL, et al. Diacylglycerol lipase beta inhibition reverses nociceptive behaviour in mouse models of inflammatory and neuropathic pain. Br J Pharmacol. 2016(10):1678-92.

588. Woller SA, Corr M, Yaksh TL. Differences in cisplatin-induced mechanical allodynia in male and female mice. Eur J Pain. 2015:1476-85.

589. Wongtawatchai T, Agthong S, Kaewsema A, Chentanez V. Sex-related differences in cisplatin-induced neuropathy in rats. J Med Assoc Thai. 2009:1485-91.

590. Wongtawatchai T, Agthong S, Kaewsema A, Chentanez V. Altered phosphorylation of mitogen-activated protein kinases in dorsal root ganglia and sciatic nerve of rats with cisplatin-induced neuropathy. Asian Biomedicine. 2012:397-411.

591. Working PK, Newman MS, Sullivan T, Brunner M, Podell M, Sahenk Z, et al. Comparative intravenous toxicity of cisplatin solution and cisplatin encapsulated in long-circulating, pegylated liposomes in cynomolgus monkeys. Toxicol Sci. 1998:155-65.

592. Wozniak KM, Nomoto K, Lapidus RG, Wu Y, Carozzi V, Cavaletti G, et al. Comparison of neuropathy-inducing effects of eribulin mesylate, paclitaxel, and ixabepilone in mice. Cancer Res. 2011:3952-62.

593. Wozniak KM, Vornov JJ, Wu Y, Liu Y, Carozzi VA, Rodriquez-Menendez V, et al. Peripheral neuropathy induced by microtubule-targeted chemotherapies: insights into acute injury and long-term recovery. Cancer Res. 2017.

594. Wozniak KM, Vornov JJ, Wu Y, Nomoto K, Littlefield BA, DesJardins C, et al. Sustained Accumulation of Microtubule-Binding Chemotherapy Drugs in the Peripheral Nervous System: Correlations with Time Course and Neurotoxic Severity. Cancer Res. 2016(11):3332-9.

595. Wozniak KM, Wu Y, Farah MH, Littlefield BA, Nomoto K, Slusher BS. Neuropathy-inducing effects of eribulin mesylate versus paclitaxel in mice with preexisting neuropathy. Neurotoxicity Research. 2013:338-44.

596. Wozniak KM, Wu Y, Vornov JJ, Lapidus R, Rais R, Rojas C, et al. The orally active glutamate carboxypeptidase II inhibitor E2072 exhibits sustained nerve exposure and attenuates peripheral neuropathy. J Pharmacol Exp Ther. 2012:746-54.

597. Wu Y, Li J, Zhou J, Feng Y. Dynamic long-term microstructural and ultrastructural alterations in sensory nerves of rats of paclitaxel-induced neuropathic pain. Chin Med J (Engl). 2014:2945-52.

598. Wu YQ, Dang RL, Tang MM, Cai HL, Li HD, Liao DH, et al. Long Chain Omega-3 Polyunsaturated Fatty Acid Supplementation Alleviates Doxorubicin-Induced Depressive-Like Behaviors and Neurotoxicity in Rats: Involvement of Oxidative Stress and Neuroinflammation. Nutrients. 2016(4):243.

599. Wu Z, Wang S, Wu I, Mata M, Fink DJ. Activation of TLR-4 to produce tumour necrosis factor-alpha in neuropathic pain caused by paclitaxel. Eur J Pain. 2015:889-98.

600. Xiao W, Boroujerdi A, Bennett GJ, Luo ZD. Chemotherapy-evoked painful peripheral neuropathy: analgesic effects of gabapentin and effects on expression of the alpha-2-delta type-1 calcium channel subunit. Neuroscience. 2007:714-20.

601. Xiao WH, Bennett GJ. Chemotherapy-evoked neuropathic pain: Abnormal spontaneous discharge in A-fiber and C-fiber primary afferent neurons and its suppression by acetyl-L-carnitine. Pain. 2008:262-70.

602. Xiao WH, Zheng FY, Bennett GJ, Bordet T, Pruss RM. Olesoxime (cholest-4-en-3-one, oxime): analgesic and neuroprotective effects in a rat model of painful peripheral neuropathy produced by the chemotherapeutic agent, paclitaxel. Pain. 2009:202-9.

603. Xiao WH, Zheng H, Bennett GJ. Characterization of oxaliplatin-induced chronic painful peripheral neuropathy in the rat and comparison with the neuropathy induced by paclitaxel. Neuroscience. 2012:194-206.

604. Xiao WH, Zheng H, Zheng FY, Nuydens R, Meert TF, Bennett GJ. Mitochondrial abnormality in sensory, but not motor, axons in paclitaxel-evoked painful peripheral neuropathy in the rat. Neuroscience. 2011:461-9.

605. Xie JD, Chen SR, Chen H, Pan HL. Bortezomib induces neuropathic pain through protein kinase C-mediated activation of presynaptic NMDA receptors in the spinal cord. Neuropharmacology. 2017:477-87.

606. Xu F, Xu S, Wang L, Chen C, Zhou X, Lu Y, et al. Antinociceptive efficacy of verticinone in murine models of inflammatory pain and paclitaxel induced neuropathic pain. Biol Pharm Bull. 2011:1377-82.

607. Xu J, Wang W, Zhong XX, Feng Y, Wei X, Liu XG. Methylcobalamin ameliorates neuropathic pain induced by vincristine in rats: Effect on loss of peripheral nerve fibers and imbalance of cytokines in the spinal dorsal horn. Mol Pain. 2016.

608. Xu JJ, Diaz P, Bie B, Astruc-Diaz F, Wu J, Yang H, et al. Spinal gene expression profiling and pathways analysis of a CB<inf>2</inf> agonist (MDA7)-targeted prevention of paclitaxel-induced neuropathy. Neuroscience. 2014:185-94.

609. Xu T, Li D, Zhou X, Ouyang HD, Zhou LJ, Zhou H, et al. Oral Application of Magnesium-L-Threonate Attenuates Vincristine-induced Allodynia and Hyperalgesia by Normalization of Tumor Necrosis Factor-alpha/Nuclear Factor-kappaB Signaling. Anesthesiology. 2017(6):1151-68.

610. Xu T, Zhang XL, Ou-Yang HD, Li ZY, Liu CC, Huang ZZ, et al. Epigenetic upregulation of CXCL12 expression mediates antitubulin chemotherapeutics-induced neuropathic pain. Pain. 2017(4):637-48.

611. Xu Y, Cheng G, Zhu Y, Zhang X, Pu S, Wu J, et al. Anti-nociceptive roles of the glia-specific metabolic inhibitor fluorocitrate in paclitaxel-evoked neuropathic pain. Acta Biochim Biophys Sin (Shanghai). 2016(10):902-8.

612. Yalcin S, Kilickap S, Temucin CM, Erman M. Recombinant human erythropoietin in comparison to amifostine against cisplatin-induced peripheral sensorial neurotoxicity in rats. Journal of Experimental and Clinical Cancer Research. 2006:523-7.

613. Yalcin S, Nurlu G, Orhan B, Zeybek D, Muftuoglu S, Sarer B, et al. Protective effect of amifostine against cisplatin-induced motor neuropathy in rat. Med Oncol. 2003:175-80.

614. Yamamoto K, Chiba N, Chiba T, Kambe T, Abe K, Kawakami K, et al. Transient receptor potential ankyrin 1 that is induced in dorsal root ganglion neurons contributes to acute cold hypersensitivity after oxaliplatin administration. Mol Pain. 2015:69.

615. Yamamoto K, Tsuboi M, Kambe T, Abe K, Nakatani Y, Kawakami K, et al. Oxaliplatin administration increases expression of the voltage-dependent calcium channel alpha2delta-1 subunit in the rat spinal cord. J Pharmacol Sci. 2016:117-22.

616. Yamamoto S, Kawashiri T, Higuchi H, Tsutsumi K, Ushio S, Kaname T, et al. Behavioral and pharmacological characteristics of bortezomib-induced peripheral neuropathy in rats. Journal of Pharmacological Sciences. 2015:43-50.

617. Yamamoto S, Ono H, Kume K, Ohsawa M. Oxaliplatin treatment changes the function of sensory nerves in rats. J Pharmacol Sci. 2016:189-93.

618. Yamashita Y, Egashira N, Masuguchi K, Ushio S, Kawashiri T, Oishi R. Comparison of peripheral neuropathy induced by standard and nanoparticle albumin-bound paclitaxel in rats. Journal of Pharmacological Sciences. 2011:116-20.

619. Yamashita Y, Irie K, Kochi A, Kimura N, Hayashi T, Matsuo K, et al. Involvement of Charcot-Marie-Tooth disease gene mitofusin 2 expression in paclitaxel-induced mechanical allodynia in rats. Neuroscience Letters. 2017:337-40.

620. Yan F, Liu JJ, Ip V, Jamieson SM, McKeage MJ. Role of platinum DNA damage-induced transcriptional inhibition in chemotherapy-induced neuronal atrophy and peripheral neurotoxicity. J Neurochem. 2015;135(6):1099-112.

621. Yan X, Maixner DW, Yadav R, Gao M, Li P, Bartlett MG, et al. Paclitaxel Induces Acute Pain via Directly Activating Toll like Receptor 4. Molecular Pain. 2015(no pagination).

622. Yang Y, Zhang YG, Lin GA, Xie HQ, Pan HT, Huang BQ, et al. Spinal changes of a newly isolated neuropeptide endomorphin-2 concomitant with vincristine-induced allodynia. PLoS One. 2014:e89583.

623. Yeo JH, Yoon SY, Kim SJ, Oh SB, Lee JH, Beitz AJ, et al. Clonidine, an alpha-2 adrenoceptor agonist relieves mechanical allodynia in oxaliplatin-induced neuropathic mice; potentiation by spinal p38 MAPK inhibition without motor dysfunction and hypotension. Int J Cancer. 2016:2466-76.

624. Yeo JH, Yoon SY, Kwon SK, Kim SJ, Lee JH, Beitz AJ, et al. Repetitive acupuncture point treatment with diluted bee venom relieves mechanical allodynia and restores intraepidermal nerve fiber loss in oxaliplatin-induced neuropathic mice. Journal of Pain. 2016:298-309.

625. Yilmaz E, Gold MS. Sensory neuron subpopulation-specific dysregulation of intracellular calcium in a rat model of chemotherapy-induced peripheral neuropathy. Neuroscience. 2015:210-8.

626. Yilmaz E, Gold MS. Paclitaxel-induced increase in NCX activity in subpopulations of nociceptive afferents: A protective mechanism against chemotherapy-induced peripheral neuropathy? Cell Calcium. 2016.

627. Yoon H, Kim MJ, Yoon I, Li DX, Bae H, Kim SK. Nicotinic Acetylcholine Receptors Mediate the Suppressive Effect of an Injection of Diluted Bee Venom into the GV3 Acupoint on Oxaliplatin-Induced Neuropathic Cold Allodynia in Rats. Biol Pharm Bull. 2015:710-4.

628. Yoon MS, Katsarava Z, Obermann M, Schafers M, Liedert B, Dzagnidze A, et al. Erythropoietin overrides the triggering effect of DNA platination products in a mouse model of cisplatin-induced neuropathy. BMC Neurosci. 2009:77.

629. Yoon SY, Robinson CR, Zhang H, Dougherty PM. Spinal astrocyte gap junctions contribute to oxaliplatin-induced mechanical hypersensitivity. J Pain. 2013:205-14.

630. Yoon SY, Yeo JH, Han SD, Bong DJ, Oh B, Roh DH. Diluted bee venom injection reduces ipsilateral mechanical allodynia in oxaliplatin-induced neuropathic mice. Biol Pharm Bull. 2013:1787-93.

631. Zanardelli M, Micheli L, Cinci L, Failli P, Ghelardini C, Di Cesare Mannelli L. Oxaliplatin neurotoxicity involves peroxisome alterations. PPARgamma agonism as preventive pharmacological approach. PLoS One. 2014:e102758.

632. Zbarcea CE, Ciotu IC, Bild V, ChiriTa C, Tanase AM, Seremet OC, et al. Therapeutic potential of certain drug combinations on paclitaxel-induced peripheral neuropathy in rats. Rom J Morphol Embryol. 2017(2):507-16.

633. Zbarcea CE, Negres S, Chirita C. Gabapentin, alone and associated with tramadol reduces peripheral paclitaxel-induced neuropathy in rats. Farmacia. 2011:414-23.

634. Zbarcea CE, Negres S, Nicoleta Cristea A, Chirita C. The effect of dextromethorphan, gabapentin, amitriptyline and tramadol on a mouse model of vincristine - induced peripheral neuropathy. Farmacia. 2011:809-19.

635. Zhang H, Boyette-Davis JA, Kosturakis AK, Li Y, Yoon SY, Walters ET, et al. Induction of monocyte chemoattractant protein-1 (mcp-1) and its receptor ccr2 in primary sensory neurons contributes to paclitaxel-induced peripheral neuropathy. Journal of Pain. 2013:1031-44.

636. Zhang H, Dougherty PM. Enhanced excitability of primary sensory neurons and altered gene expression of neuronal ion channels in dorsal root ganglion in paclitaxel-induced peripheral neuropathy. Anesthesiology. 2014:1463-75.

637. Zhang H, Li Y, de Carvalho-Barbosa M, Kavelaars A, Heijnen CJ, Albrecht PJ, et al. Dorsal Root Ganglion Infiltration by Macrophages Contributes to Paclitaxel Chemotherapy-Induced Peripheral Neuropathy. J Pain. 2016.

638. Zhang J, Su YM, Li D, Cui Y, Huang ZZ, Wei JY, et al. TNF-alpha-mediated JNK activation in the dorsal root ganglion neurons contributes to Bortezomib-induced peripheral neuropathy. Brain, Behavior, and Immunity. 2014:185-91.

639. Zhang J, Tuckett RP. Comparison of paclitaxel and cisplatin effects on the slowly adapting type I mechanoreceptor. Brain Research. 2008:50-7.

640. Zhang Y, Li A, Xin J, Ren K, Berman BM, Lao L, et al. Electroacupuncture alleviates chemotherapy-induced pain through inhibiting phosphorylation of spinal CaMKII in rats. Eur J Pain. 2017.

641. Zhao M, Isami K, Nakamura S, Shirakawa H, Nakagawa T, Kaneko S. Acute cold hypersensitivity characteristically induced by oxaliplatin is caused by the enhanced responsiveness of TRPA1 in mice. Molecular Pain. 2012.

642. Zhao M, Nakamura S, Miyake T, So K, Shirakawa H, Tokuyama S, et al. Pharmacological characterization of standard analgesics on oxaliplatin-induced acute cold hypersensitivity in mice. Journal of Pharmacological Sciences. 2014:514-7.

643. Zheng FY, Xiao WH, Bennett GJ. The response of spinal microglia to chemotherapy-evoked painful peripheral neuropathies is distinct from that evoked by traumatic nerve injuries. Neuroscience. 2011:447-54.

644. Zheng H, Xiao WH, Bennett GJ. Functional deficits in peripheral nerve mitochondria in rats with paclitaxel- and oxaliplatin-evoked painful peripheral neuropathy. Exp Neurol. 2011:154-61.

645. Zhou FH, Yu Y, Zhou XF, Xian CJ. Methotrexate chemotherapy triggers touch-evoked pain and increased CGRP-positive sensory fibres in the tibial periosteum of young rats. Bone. 2015:24-31.

646. Zhou HH, Zhang L, Zhou QG, Fang Y, Ge WH. (+)-Borneol attenuates oxaliplatin-induced neuropathic hyperalgesia in mice. Neuroreport. 2016:160-5.

647. Zhou W, Kavelaars A, Heijnen CJ. Metformin prevents cisplatin-induced cognitive impairment and brain damage in mice. PLoS ONE. 2016.

648. Zhu HQ, Xu J, Shen KF, Pang RP, Wei XH, Liu XG. Bulleyaconitine A depresses neuropathic pain and potentiation at C-fiber synapses in spinal dorsal horn induced by paclitaxel in rats. Exp Neurol. 2015:263-72.

649. Zhu J, Carozzi VA, Reed N, Mi R, Marmiroli P, Cavaletti G, et al. Ethoxyquin provides neuroprotection against cisplatin-induced neurotoxicity. Sci Rep. 2016;6:28861.

650. Zhu J, Chen W, Mi R, Zhou C, Reed N, Hoke A. Ethoxyquin prevents chemotherapy-induced neurotoxicity via Hsp90 modulation. Ann Neurol. 2013:893-904.
