# Supplementary material for: A systematic summary and comparison of animal models for chemotherapy induced (peripheral) neuropathy (CIPN)
Source: PLoS One. 2019 Aug 28;14(8):e0221787. doi: 10.1371/journal.pone.0221787 (PMC6713358; doi:10.1371/journal.pone.0221787)
Supplement: S3 File — (DOCX) [file pone.0221787.s003.docx]

# S3 File: Full search strategy

| **Pubmed** | |
| --- | --- |
| Chemotherapy | 3-Deazauridine [MESH] OR 3-Iodobenzylguanidine [MESH] OR 6-Mercaptopurine [MESH] OR Abiraterone Acetate [MESH] OR Aclarubicin [MESH] OR Acronine [MESH] OR “Adenosine-5'-(N-ethylcarboxamide)” [MESH] OR Albumin-Bound Paclitaxel [MESH] OR Altretamine [MESH] OR Aminoglutethimide [MESH] OR Amsacrine [MESH] OR Ancitabine [MESH] OR Aniline Mustard [MESH] OR Anthramycin [MESH] OR Asparaginase [MESH] OR Azacitidine [MESH] OR Azaguanine [MESH] OR Azaserine [MESH] OR Azathioprine [MESH] OR Azauridine [MESH] OR Bendamustine Hydrochloride [MESH] OR Bevacizumab [MESH] OR Bleomycin [MESH] OR Bortezomib [MESH] OR Bromodeoxyuridine [MESH] OR Busulfan [MESH] OR Buthionine Sulfoximine [MESH] OR Camptothecin [MESH] OR Capecitabine [MESH] OR Carbazilquinone [MESH] OR Carboplatin [MESH] OR Carmustine [MESH] OR Carubicin [MESH] OR Cetuximab [MESH] OR Chlorambucil [MESH] OR Chlorotrianisene [MESH] OR Cisplatin [MESH] OR Cladribine [MESH] OR Coformycin [MESH] OR Cyclophosphamide [MESH] OR Cyproterone Acetate [MESH] OR Cytarabine [MESH] OR Dacarbazine [MESH] OR Dactinomycin [MESH] OR Dasatinib [MESH] OR Daunorubicin [MESH] OR Demecolcine [MESH] OR Deuterium Oxide OR Dianhydrogalactitol [MESH] OR Diazooxonorleucine [MESH] OR Dihematoporphyrin Ether [MESH] OR Doxorubicin [MESH] OR Ellipticines [MESH] OR Epirubicin [MESH] OR Erlotinib Hydrochloride [MESH] OR Estramustine [MESH] OR Etanidazole [MESH] OR Ethoglucid [MESH] OR Ethyl Methanesulfonate [MESH] OR Etoposide [MESH] OR Everolimus [MESH] OR Fadrozole [MESH] OR Fenretinide [MESH] OR Floxuridine [MESH] OR Fluorouracil [MESH] OR Flutamide [MESH] OR Formycins [MESH] OR Genistein [MESH] OR Glaucarubin [MESH] OR Gold Colloid, Radioactive [MESH] OR Goserelin [MESH] OR Guanazole [MESH] OR Harringtonines [MESH] OR Hematoporphyrin Derivative [MESH] OR Hydroxyurea [MESH] OR Idarubicin [MESH] OR Ifosfamide [MESH] OR Imatinib Mesylate [MESH] OR Interferon beta-1a [MESH] OR Interferon Type I [MESH] OR Interferon-beta [MESH] OR Interferons [MESH] OR Interleukin-12 [MESH] OR Interleukin-2 [MESH] OR Interleukin-4 [MESH] OR Lentinan [MESH] OR Leuprolide [MESH] OR Mannomustine [MESH] OR Maytansine [MESH] OR Megestrol [MESH] OR Megestrol Acetate [MESH] OR Melphalan [MESH] OR Menogaril [MESH] OR Methotrexate [MESH] OR Methyl Methanesulfonate [MESH] OR Mitobronitol [MESH] OR Mitoguazone [MESH] OR Mitolactol [MESH] OR Mitomycin [MESH] OR Mitomycins [MESH] OR Mitotane [MESH] OR Mitoxantrone [MESH] OR Mopidamol [MESH] OR Mycophenolic Acid [MESH] OR Nafoxidine [MESH] OR Nimustine [MESH] OR Nitracrine [MESH] OR Nitrogen Mustard Compounds [MESH] OR Nocodazole [MESH] OR Nogalamycin OR Oncostatin M [MESH] OR Paclitaxel [MESH] OR Pactamycin OR Pemetrexed [MESH] OR Pentostatin [MESH] OR Peplomycin [MESH] OR Peptichemio [MESH] OR Phleomycins [MESH] OR Phosphoramide Mustards [MESH] OR Picibanil [MESH] OR Pipobroman [MESH] OR Plicamycin [MESH] OR Podophyllin [MESH] OR Podophyllotoxin [MESH] OR Porfiromycin [MESH] OR Prednimustine [MESH] OR Procarbazine [MESH] OR Prospidium [MESH] OR Puromycin [MESH] OR Puromycin Aminonucleoside [MESH] OR Pyran Copolymer [MESH] OR Ranibizumab [MESH] OR Razoxane [MESH] OR Rituximab [MESH] OR Semustine [MESH] OR Showdomycin [MESH] OR Sirolimus [MESH] OR Sparsomycin [MESH] OR Streptonigrin OR Streptozocin [MESH] OR Sulindac [MESH] OR Swainsonine [MESH] OR Tamoxifen [MESH] OR Tegafur [MESH] OR Teniposide [MESH] OR Tenuazonic Acid [MESH] OR Testolactone [MESH] OR Thalidomide [MESH] OR Thioguanine [MESH] OR Thioinosine [MESH] OR Thiotepa [MESH] OR Topotecan [MESH] OR Toremifene [MESH] OR Toyocamycin [MESH] OR Trastuzumab [MESH] OR Tretinoin [MESH] OR Triaziquone [MESH] OR Trichosanthin [MESH] OR Triethylenemelamine [MESH] OR Triethylenephosphoramide [MESH] OR Trimetrexate [MESH] OR Triptorelin Pamoate [MESH] OR Tubercidin [MESH] OR Tumor Necrosis Factor Ligand Superfamily Member 15 [MESH] OR Tyrphostins [MESH] OR Uracil Mustard [MESH] OR Vinblastine [MESH] OR Vinca Alkaloids [MESH] OR Vincristine [MESH] OR Vindesine [MESH] OR Altretamine [tiab] OR Aminoglutethimide [tiab] OR Amsacrine [tiab] OR Ancitabine [tiab] OR Aniline Mustard [tiab] OR Anthramycin [tiab] OR Azaguanine [tiab] OR Azaserine [tiab] OR Azathioprine [tiab] OR Azauridine [tiab] OR Bromodeoxyuridine [tiab] OR Camptothecin [tiab] OR Carubicin [tiab] OR Carbazilquinone [tiab] OR Chlorotrianisene [tiab] OR Cladribine [tiab] OR Coformycin [tiab] OR Cyproterone Acetate [tiab] OR Etanidazole [tiab] OR Demecolcine [tiab] OR Deuterium Oxide [tiab] OR Sirolimus [tiab] OR Nocodazole [tiab] OR Swainsonine [tiab] OR Plicamycin [tiab] OR Idarubicin [tiab] OR idarubicine [tiab] OR Harringtonine [tiab] OR Semustine [tiab] OR leuprolide [tiab] OR Estramustine [tiab] OR Estramustin [tiab] OR Guanazole [tiab] OR Trichosanthin [tiab] OR Gold Colloid Radioactive [tiab] OR Mopidamol [tiab] OR Picibanil [tiab] OR Glaucarubine [tiab] OR Glaucarubin [tiab] OR Fenretidine [tiab] OR Nimustine [tiab] OR Nimustin [tiab] OR Razoxane [tiab] OR Estramustine [tiab] OR Estramustin [tiab] OR Toremifene [tiab] OR Lentinan [tiab] OR Hydroxyurea [tiab] OR hydroxycarbamide [tiab] OR Genistein [tiab] OR Nitracrine [tiab] OR Tretinoin [tiab] OR Thioinosine [tiab] OR Pactamycin [tiab] OR Prospidium [tiab] OR Pentostatin OR Pemetrexed [tiab] OR prednimustine [tiab] OR Mitolactol [tiab] OR Mitotane [tiab] OR Sulindac [tiab] OR Tegafur [tiab] OR Ellipticines [tiab] OR Ellipticine [tiab] OR Menogaril [tiab] OR Dianhydrogalactitol [tiab] OR Mannomustine [tiab] OR Mannomustin [tiab] OR Teniposide [tiab] OR Maytansine [tiab] OR Maytansin [tiab] OR Pipobroman [tiab] OR Fluorouracil [tiab] OR Toremifene [tiab] OR Ethoglucid [tiab] OR Nafoxidine [tiab] OR Nafoxidin [tiab] OR Triaziquone [tiab] OR Sparsomycin [tiab] OR Puromycin [tiab] OR Nogalamycin [tiab] OR Trimetrexate [tiab] OR Buthionine Sulfoximine [tiab] OR Diazooxonorleucine [tiab] OR Peplomycin [tiab] OR Formycins [tiab] OR Fadrozole [tiab] OR Toyocamycin [tiab] OR Testolactone [tiab] OR Porfiromycin [tiab] OR Mitoxantrone [tiab] OR Dihematoporphyrin [tiab] OR Mitobronitol [tiab] OR Methanesulfonate [tiab] OR Mitoguazone [tiab] OR Podophyllin [tiab] OR Triptorelin Pamoate [tiab] OR Hematoporphyrin Derivative [tiab] OR Floxuridine [tiab] OR Pyran Copolymer [tiab] OR Peptichemio [tiab] OR Tenuazonic Acid [tiab] OR Phleomycins [tiab] OR Triethylenephosphoramide [tiab] OR Podophyllotoxin OR Floxuridine OR Streptonigrin OR Triethylenemelamine OR Phosphoramide Mustards [tiab] OR Showdomycin [tiab] OR Tumor Necrosis Factor Ligand Superfamily Member 15 [tiab] OR Interferon beta-1a [tiab] OR Interferon Type I [tiab] OR Interferon-beta [tiab] OR Interferons [tiab] OR Interleukin-12 [tiab] OR Interleukin-2 [tiab] OR Interleukin-4 [tiab] OR Methyl Methanesulfonate [tiab] OR Streptozocin [tiab] OR chemo-therapy [tiab] OR radio(chemo)therapy [tiab] OR radio(chemo)therapies [tiab] OR paresthesia [mesh] OR parasthesia [tiab] OR paresthesias [tiab] OR dysesthesias [tiab] OR dysesthesia [tiab] OR anticancer drug [tiab] OR anti-cancer drugs [tiab] OR anti-tumor drugs [tiab] OR anti-tumor drug [tiab] OR anti-cancer agents [tiab] OR anti-cancer agent [tiab] OR anti-tumor agent [tiab] OR anti-tumor agents [tiab] OR anti-tumor drug [tiab] OR anti-tumor drugs [tiab] OR anti-carcinogenic agents [tiab] OR anti-carcinogenic agent [tiab] OR anti- carcinogenic agents [tiab] OR anti- carcinogenic agent [tiab] OR anti-carcinogenic drug [tiab] OR anti-carcinogenic drugs [tiab] OR anti- carcinogenic drug [tiab] OR anti- carcinogenic drugs [tiab] OR anti-mitotic agents [tiab] OR anti-mitotic agent [tiab] OR anti-mitotic drugs [tiab] OR anti-mitotic drug [tiab] OR anti-mitotic [tiab] OR anti-mitotics [tiab] OR chemotherapy [tiab] OR chemotherapies [tiab] OR Chemotherapy, Adjuvant [MESH] OR Consolidation chemotherapy [MESH] OR Induction chemotherapy [MESH] OR Maintenance chemotherapy [MESH] OR Chemotherapy, Cancer, Regional Perfusion [MESH] OR Antineoplastic agents [MESH] OR antineoplastics [tiab] OR antineoplastic [tiab] OR chemotherapeutic [tiab] OR anticancer drug [tiab] OR anticancer drugs [tiab] OR antitumor drugs [tiab] OR antitumor drug [tiab] OR anticancer agents [tiab] OR anticancer agent [tiab] OR antitumor agent [tiab] OR antitumor agents [tiab] OR antitumor drug [tiab] OR antitumor drugs [tiab] OR Anticarcinogenic agents [MESH] OR anticarcinogenic agents [tiab] OR anticarcinogenic agent [tiab] OR anti carcinogenic agents [tiab] OR anti carcinogenic agent [tiab] OR anticarcinogenic drug [tiab] OR anticarcinogenic drugs [tiab] OR anti carcinogenic drug [tiab] OR anti carcinogenic drugs [tiab]OR Antimitotic agents [MESH] OR antimitotic agents [tiab] OR antimitotic agent [tiab] OR antimitotic drugs [tiab] OR antimitotic drug [tiab] OR antimitotic [tiab] OR antimitotics [tiab] OR afatinib [tiab] OR everolimus [tiab] OR afinitor [tiab] OR carmustine [tiab] OR carmustin [tiab] OR belinostat [tiab] OR bendamustine [tiab] OR bicalutamide [tiab] OR blinatumomab [tiab] OR brentuximab [tiab] OR busulfan [tiab] OR bortezomib [tiab] OR bevacizumab [tiab] OR bosutinib [tiab] OR bleomycine [tiab] OR bleomycin [tiab] OR blinatumomab [tiab] OR carbazitaxel [tiab] OR carboplatine [tiab] OR carboplatin [tiab] OR capecitabine [tiab] OR capecitabin [tiab] OR lomustine [tiab] OR lomustin [tiab] OR ceritinib [tiab] OR carfilzomib [tiab] OR crizotinib [tiab] OR cyclofosfamide [tiab] OR cyclophosphamide [tiab] OR Ifosfamide [tiab] OR ramucirumab [tiab] OR cytarabine [tiab] OR cytarabin [tiab] OR dabrafenib [tiab] OR dacarbazine [tiab] OR dacarbazin [tiab] OR daratumumab [tiab] OR dasatinib [tiab] OR daunorubicine [tiab] OR daunorubicin [tiab] OR daunomycin [tiab] OR daunomycine [tiab] OR decitabine [tiab] OR decitabin [tiab] OR denileukin [tiab] OR docetaxel [tiab] OR doxorubicine [tiab] OR doxorubicin [tiab] OR rasburicase [tiab] OR epirubicin [tiab] OR epirubicine [tiab] OR elotuzumab [tiab] OR eltrombopag [tiab] OR enzalutamide [tiab] OR eribulin [tiab] OR eribuline [tiab] OR vismodegib [tiab] OR erlotinib [tiab] OR asparaginase [tiab] OR etoposide [tiab] OR panobinostat [tiab] OR fulvestrant [tiab] OR letrozol [tiab] OR letrozole [tiab] OR fludarabine [tiab] OR fludarabin [tiab] OR flutamide [tiab] OR pralatrexate [tiab] OR obinutuzumab [tiab] OR gefitinib [tiab] OR imatinib [tiab] OR trastuzumab [tiab] OR emtansine [tiab] OR topotecan [tiab] OR pembrolizumab [tiab] OR lapatinib [tiab] OR lanreotide [tiab] OR lenalidomide [tiab] OR olaparib [tiab] OR vincristine [tiab] OR vincristin [tiab] OR procarbazine [tiab] OR mustine [tiab] OR mustargen [tiab] OR megestrol [tiab] OR mercaptopurine [tiab] OR temozolomide [tiab] OR trametinib [tiab] OR mitomycine [tiab] OR mitomycin [tiab] OR mitoxantrone [tiab] OR paclitaxel [tiab] OR vinorelbine [tiab] OR sorafenib [tiab] OR nilotinib [tiab] OR nivolumab [tiab] OR tamoxifen [tiab] OR pegaspargase [tiab] OR oxaliplatin [tiab] OR oxaliplatine [tiab] OR panitumumab [tiab] OR pazopanib [tiab] OR pertuzumab [tiab] OR pomalidomide [tiab] OR ponatinib [tiab] OR sipuleucel-T [tiab] OR interferon alfa-2b [tiab] OR regorafenib [tiab] OR rituximab [tiab] OR romidepsin [tiab] OR sunitinib [tiab] OR tioguanine [tiab] OR thioguanine [tiab] OR thiotepa [tiab] OR temsirolimus [tiab] OR thalidomide [tiab] OR trabectedine [tiab] OR trabectedin [tiab] OR vandetanib [tiab] OR vinblastine [tiab] OR ipilimumab [tiab] OR aflibercept [tiab] OR ibritumomab [tiab] OR idelalisib [tiab] OR cetuximab [tiab] OR anastrozol [tiab] OR anastrozole [tiab] OR degarelix [tiab] OR osimertinib [tiab] OR ruxolitinib [tiab] OR palbociclib [tiab] OR ixazomib [tiab] OR ofatumumab [tiab] OR alectinib [tiab] OR filgrastim [tiab] OR vorinostat [tiab] OR talimogene [tiab] OR romiplostin [tiab] OR siltuximab [tiab] OR plerixafor [tiab] OR azacitidine [tiab] OR gosereline [tiab] OR goserelin [tiab] OR abirateron [tiab] OR abiraterone [Tiab] OR lenvatinib [tiab] OR leuproreline [tiab] OR leuprorelin [tiab] OR trifluridine [tiab] OR tipiracil [tiab] OR necitumumab [tiab] OR chlorambucil [Mesh] OR chlorambucil [tiab] OR bexarotene [tiab] OR mechlorethamine [MESH] OR mechlorethamine [tiab] OR omacetaxine [tiab] OR emtansine [tiab] |
| Polyneuropathy | Polyneuropathies[Mesh] OR Polyneuropathies [tiab] OR Polyneuropathy [tiab] OR Polyradiculoneuropathies [tiab] OR Polyradiculoneuropathy [tiab]OR Polyradiculoneuritis [tiab] OR Polyradiculoneuritides [tiab] OR Peripheral Autoimmune Demyelinating Disease [tiab] OR neurotoxicity [tiab] OR Neuralgia[Mesh] OR Neuralgia [tiab] OR neuralgias [tiab] OR nerve pain [tiab] OR nerve pains [tiab] OR neurodynia [tiab] OR Neuropathic pain [tiab] OR neuropathic pains [tiab] OR Peripheral Nerve Injuries [Mesh] OR Peripheral Nerve Injury [tiab] OR Peripheral Nerve Injuries [tiab] OR Peripheral Nerve damage [tiab] OR Peripheral Nerve atrophy [tiab] OR axonopathy [tiab] OR axonopathies [tiab] OR myelinopathy [tiab] OR myelinopathies [tiab] OR "Peripheral Nerves/Injuries"[Mesh] OR neuralgic pain [tiab] OR neuralgic pains [tiab] |
| Experimental animal | ("animal experimentation"[MeSH Terms] OR "models, animal"[MeSH Terms] OR "invertebrates"[MeSH Terms] OR "Animals"[Mesh:noexp] OR "animal population groups"[MeSH Terms] OR "chordata"[MeSH Terms:noexp] OR "vertebrates"[MeSH Terms:noexp] OR "chordata, nonvertebrate"[MeSH Terms] OR "amphibians"[MeSH Terms] OR "birds"[MeSH Terms] OR "fishes"[MeSH Terms] OR "reptiles"[MeSH Terms] OR "mammals"[MeSH Terms:noexp] OR "primates"[MeSH Terms:noexp] OR "artiodactyla"[MeSH Terms] OR "carnivora"[MeSH Terms] OR "cetacea"[MeSH Terms] OR "chiroptera"[MeSH Terms] OR "elephants"[MeSH Terms] OR "hyraxes"[MeSH Terms] OR "insectivora"[MeSH Terms] OR "lagomorpha"[MeSH Terms] OR "marsupialia"[MeSH Terms] OR "monotremata"[MeSH Terms] OR "perissodactyla"[MeSH Terms] OR "rodentia"[MeSH Terms] OR "scandentia"[MeSH Terms] OR "sirenia"[MeSH Terms] OR "xenarthra"[MeSH Terms] OR "haplorhini"[MeSH Terms:noexp] OR "strepsirhini"[MeSH Terms] OR "platyrrhini"[MeSH Terms] OR "tarsii"[MeSH Terms] OR "catarrhini"[MeSH Terms:noexp] OR "cercopithecidae"[MeSH Terms] OR "hylobatidae"[MeSH Terms] OR "hominidae"[MeSH Terms:noexp] OR "gorilla gorilla"[MeSH Terms] OR "pan paniscus"[MeSH Terms] OR "pan troglodytes"[MeSH Terms] OR "pongo pygmaeus"[MeSH Terms]) OR ((animals[tiab] OR animal[tiab] OR mice[Tiab] OR mus[Tiab] OR mouse[Tiab] OR murine[Tiab] OR woodmouse[tiab] OR rats[Tiab] OR rat[Tiab] OR murinae[Tiab] OR muridae[Tiab] OR cottonrat[tiab] OR cottonrats[tiab] OR hamster[tiab] OR hamsters[tiab] OR cricetinae[tiab] OR rodentia[Tiab] OR rodent[Tiab] OR rodents[Tiab] OR pigs[Tiab] OR pig[Tiab] OR swine[tiab] OR swines[tiab] OR piglets[tiab] OR piglet[tiab] OR boar[tiab] OR boars[tiab] OR "sus scrofa"[tiab] OR ferrets[tiab] OR ferret[tiab] OR polecat[tiab] OR polecats[tiab] OR "mustela putorius"[tiab] OR "guinea pigs"[Tiab] OR "guinea pig"[Tiab] OR cavia[Tiab] OR callithrix[Tiab] OR marmoset[Tiab] OR marmosets[Tiab] OR cebuella[Tiab] OR hapale[Tiab] OR octodon[Tiab] OR chinchilla[Tiab] OR chinchillas[Tiab] OR gerbillinae[Tiab] OR gerbil[Tiab] OR gerbils[Tiab] OR jird[Tiab] OR jirds[Tiab] OR merione[Tiab] OR meriones[Tiab] OR rabbits[Tiab] OR rabbit[Tiab] OR hares[Tiab] OR hare[Tiab] OR diptera[Tiab] OR flies[Tiab] OR fly[Tiab] OR dipteral[Tiab] OR drosophila[Tiab] OR drosophilidae[Tiab] OR cats[Tiab] OR cat[Tiab] OR carus[Tiab] OR felis[Tiab] OR nematoda[Tiab] OR nematode[Tiab] OR nematodes[Tiab] OR sipunculida[Tiab] OR dogs[Tiab] OR dog[Tiab] OR canine[Tiab] OR canines[Tiab] OR canis[Tiab] OR sheep[Tiab] OR sheeps[Tiab] OR mouflon[Tiab] OR mouflons[Tiab] OR ovis[Tiab] OR goats[Tiab] OR goat[Tiab] OR capra[Tiab] OR capras[Tiab] OR rupicapra[Tiab] OR chamois[Tiab] OR haplorhini[Tiab] OR monkey[Tiab] OR monkeys[Tiab] OR anthropoidea[Tiab] OR anthropoids[Tiab] OR saguinus[Tiab] OR tamarin[Tiab] OR tamarins[Tiab] OR leontopithecus[Tiab] OR hominidae[Tiab] OR ape[Tiab] OR apes[Tiab] OR "pan paniscus"[Tiab] OR bonobo[Tiab] OR bonobos[Tiab] OR "pan troglodytes"[Tiab] OR gibbon[Tiab] OR gibbons[Tiab] OR siamang[Tiab] OR siamangs[Tiab] OR nomascus[Tiab] OR symphalangus[Tiab] OR chimpanzee[Tiab] OR chimpanzees[Tiab] OR prosimian[Tiab] OR prosimians[Tiab] OR "bush baby"[Tiab] OR bush babies[Tiab] OR galagos[Tiab] OR galago[Tiab] OR pongidae[Tiab] OR gorilla[Tiab] OR gorillas[Tiab] OR "pongo pygmaeus"[Tiab] OR orangutan[Tiab] OR orangutans[Tiab] OR lemur[Tiab] OR lemurs[Tiab] OR lemuridae[Tiab] OR horse[Tiab] OR horses[Tiab] OR equus[Tiab] OR cow[Tiab] OR calf[Tiab] OR bull[Tiab] OR chicken[Tiab] OR chickens[Tiab] OR gallus[Tiab] OR quail[Tiab] OR bird[Tiab] OR birds[Tiab] OR quails[Tiab] OR poultry[Tiab] OR poultries[Tiab] OR fowl[Tiab] OR fowls[Tiab] OR reptile[Tiab] OR reptilia[Tiab] OR reptiles[Tiab] OR snakes[Tiab] OR snake[Tiab] OR lizard[Tiab] OR lizards[Tiab] OR alligator[Tiab] OR alligators[Tiab] OR crocodile[Tiab] OR crocodiles[Tiab] OR turtle[Tiab] OR turtles[Tiab] OR amphibian[Tiab] OR amphibians[Tiab] OR amphibia[Tiab] OR frog[Tiab] OR frogs[Tiab] OR bombina[Tiab] OR salientia[Tiab] OR toad[Tiab] OR toads[Tiab] OR "epidalea calamita"[Tiab] OR salamander[Tiab] OR salamanders[Tiab] OR eel[Tiab] OR eels[Tiab] OR fish[Tiab] OR fishes[Tiab] OR pisces[Tiab] OR catfish[Tiab] OR catfishes[Tiab] OR siluriformes[Tiab] OR arius[Tiab] OR heteropneustes[Tiab] OR sheatfish[Tiab] OR perch[Tiab] OR perches[Tiab] OR percidae[Tiab] OR perca[Tiab] OR trout[Tiab] OR trouts[Tiab] OR char[Tiab] OR chars[Tiab] OR salvelinus[Tiab] OR minnow[Tiab] OR cyprinidae[Tiab] OR carps[Tiab] OR carp[Tiab] OR zebrafish[Tiab] OR zebrafishes[Tiab] OR goldfish[Tiab] OR goldfishes[Tiab] OR guppy[Tiab] OR guppies[Tiab] OR chub[Tiab] OR chubs[Tiab] OR tinca[Tiab] OR barbels[Tiab] OR barbus[Tiab] OR pimephales[Tiab] OR promelas[Tiab] OR "poecilia reticulata"[Tiab] OR mullet[Tiab] OR mullets[Tiab] OR eel[Tiab] OR eels[Tiab] OR seahorse[Tiab] OR seahorses[Tiab] OR mugil curema[Tiab] OR atlantic cod[Tiab] OR shark[Tiab] OR sharks[Tiab] OR catshark[Tiab] OR anguilla[Tiab] OR salmonid[Tiab] OR salmonids[Tiab] OR whitefish[Tiab] OR whitefishes[Tiab] OR salmon[Tiab] OR salmons[Tiab] OR sole[Tiab] OR solea[Tiab] OR lamprey[Tiab] OR lampreys[Tiab] OR pumpkinseed[Tiab] OR sunfish[Tiab] OR sunfishes[Tiab] OR tilapia[Tiab] OR tilapias[Tiab] OR turbot[Tiab] OR turbots[Tiab] OR flatfish[Tiab] OR flatfishes[Tiab] OR sciuridae[Tiab] OR squirrel[Tiab] OR squirrels[Tiab] OR chipmunk[Tiab] OR chipmunks[Tiab] OR suslik[Tiab] OR susliks[Tiab] OR vole[Tiab] OR voles[Tiab] OR lemming[Tiab] OR lemmings[Tiab] OR muskrat[Tiab] OR muskrats[Tiab] OR lemmus[Tiab] OR otter[Tiab] OR otters[Tiab] OR marten[Tiab] OR martens[Tiab] OR martes[Tiab] OR weasel[Tiab] OR badger[Tiab] OR badgers[Tiab] OR ermine[Tiab] OR mink[Tiab] OR minks[Tiab] OR sable[Tiab] OR sables[Tiab] OR gulo[Tiab] OR gulos[Tiab] OR wolverine[Tiab] OR wolverines[Tiab] OR mustela[Tiab] OR llama[Tiab] OR llamas[Tiab] OR alpaca[Tiab] OR alpacas[Tiab] OR camelid[Tiab] OR camelids[Tiab] OR guanaco[Tiab] OR guanacos[Tiab] OR chiroptera[Tiab] OR chiropteras[Tiab] OR bat[Tiab] OR bats[Tiab] OR fox[Tiab] OR foxes[Tiab] OR iguana[Tiab] OR iguanas[Tiab] OR xenopus laevis[Tiab] OR parakeet[Tiab] OR parakeets[Tiab] OR parrot[Tiab] OR parrots[Tiab] OR donkey[Tiab] OR donkeys[Tiab] OR mule[Tiab] OR mules[Tiab] OR zebra[Tiab] OR zebras[Tiab] OR shrew[Tiab] OR shrews[Tiab] OR bison[Tiab] OR bisons[Tiab] OR buffalo[Tiab] OR buffaloes[Tiab] OR deer[Tiab] OR deers[Tiab] OR bear[Tiab] OR bears[Tiab] OR panda[Tiab] OR pandas[Tiab] OR "wild hog"[Tiab] OR "wild boar"[Tiab] OR fitchew[Tiab] OR fitch[Tiab] OR beaver[Tiab] OR beavers[Tiab] OR jerboa[Tiab] OR jerboas[Tiab] OR capybara[Tiab] OR capybaras[Tiab]) NOT medline[sb]) |

| **EMBASE** | |
| --- | --- |
| Chemotherapy | exp 3 deazauridine/ or exp 3 Iodobenzylguanidine/ or exp mercaptopurine/ or exp Abiraterone Acetate/ or exp Abiraterone/ or exp Aclarubicin/ or exp Acronine/ or exp Paclitaxel/ or exp Altretamine/ or exp Aminoglutethimide/ or exp Amsacrine/ or exp Ancitabine/ or exp Aniline Mustard/ or exp Anthramycin/ or exp Asparaginase/ or exp Azacitidine/ or exp 8 Azaguanine/ or exp Azaserine/ or exp Azathioprine/ or exp Azauridine/ or exp Bendamustine/ or exp Bevacizumab/ or exp Bleomycin/ or exp Bortezomib/ or exp Bromodeoxyuridine/ or exp Busulfan/ or exp Buthionine Sulfoximine/ or exp Camptothecin/ or exp Capecitabine/ or exp capecitabine/ or exp carboquone/ or exp Carboplatin/ or exp Carmustine/ or exp Carubicin/ or exp Cetuximab/ or exp Chlorambucil/ or exp Chlorotrianisene/ or exp Cisplatin/ or exp Cladribine/ or exp Coformycin/ or exp Cyclophosphamide/ or exp Cyproterone Acetate/ or exp Cytarabine/ or exp Dactinomycin/ or exp Dasatinib/ or exp Daunorubicin/ or exp Demecolcine/ or exp Deuterium Oxide/ or exp Dianhydrogalactitol/ or exp 6 diazo 5 oxonorleucine/ or exp photofrin II/ or exp Doxorubicin/ or exp Ellipticine derivative/ or exp Epirubicin/ or exp Erlotinib/ or exp Estramustine/ or exp Etanidazole/ or exp Ethoglucid/ or exp mesylic acid ethyl ester/ or exp Etoposide/ or exp Everolimus/ or exp Fadrozole/ or exp Fenretinide/ or exp Floxuridine/ or exp Fluorouracil/ or exp Flutamide/ or exp Formycin derivative/ or exp Genistein/ or exp Glaucarubin/ or exp gold 198/ or exp Goserelin/ or exp Guanazole/ or exp Harringtonine derivative/ or exp Hematoporphyrin Derivative/ or exp Hydroxyurea/ or exp Idarubicin/ or exp Ifosfamide/ or exp Imatinib/ or exp beta1a interferon/ or exp beta interferon/ or exp Interleukin 12/ or exp Interleukin 2/ or exp Interleukin 4/ or exp Lentinan/ or exp Leuprorelin/ or exp Mannomustine/ or exp Maytansine/ or exp Megestrol/ or exp Megestrol Acetate/ or exp Melphalan/ or exp Menogaril/ or exp Methotrexate/ or exp mesylic acid methyl ester/ or exp Mitobronitol/ or exp Mitoguazone/ or exp Mitolactol/ or exp Mitomycin/ or exp Mitomycin derivative/ or exp Mitotane/ or exp Mitoxantrone/ or exp Mopidamol/ or exp Mycophenolic Acid/ or exp Nafoxidine/ or exp Nimustine/ or exp Nitracrine/ or exp chlormethine derivative/ or exp Nocodazole/ or exp Nogalamycin/ or exp Oncostatin M/ or exp Paclitaxel/ or exp Pactamycin/ or exp Pemetrexed/ or exp Pentostatin/ or exp Pepleomycin/ or exp Peptichemio/ or exp Phleomycin/ or exp Phosphoramide Mustard/ or exp Picibanil/ or exp Pipobroman/ or exp mithramycin/ or exp Podophyllin/ or exp Podophyllotoxin/ or exp Porfiromycin/ or exp Prednimustine/ or exp Procarbazine/ or exp Prospidium/ or exp Puromycin/ or exp Puromycin Aminonucleoside/ or exp Pyran Copolymer/ or exp Ranibizumab/ or exp Razoxane/ or exp Rituximab/ or exp Semustine/ or exp Showdomycin/ or exp rapamycin/ or exp Sparsomycin/ or exp rufocromomycin/ or exp Streptozocin/ or exp Sulindac/ or exp Swainsonine/ or exp Tamoxifen/ or exp Tegafur/ or exp Teniposide/ or exp Tenuazonic Acid/ or exp Testolactone/ or exp Thalidomide/ or exp Thioguanine/ or exp mercaptopurine riboside/ or exp Thiotepa/ or exp Topotecan/ or exp Toremifene/ or exp Toyocamycin/ or exp Trastuzumab/ or exp retinoic acid/ or exp Triaziquone/ or exp Trichosanthin/ or exp tretamine/ or exp TEPA/ or exp Trimetrexate/ or exp Triptorelin/ or exp Tubercidin/ or exp vascular endothelial growth inhibitor/ or exp Vinblastine/ or exp Vinca Alkaloid/ or exp Vincristine/ or exp Vindesine/ or anti-neoplastic.ti,ab. or anti-neoplastics.ti,ab,kw. or chemo-therapy.ti,ab. or chemo-therapies.ti,ab. or chemotherapy.ti,ab. or chemotherapies.ti,ab. or chemotherapeutics.ti,ab. or chemotherapeutic.ti,ab. or chemotherapeutica.ti,ab. or chemoradiotherapy.ti,ab. or chemoradiotherapies.ti,ab. or antineoplastic.ti,ab. or antineoplastics.ti,ab. or cancer inhibition.ti,ab. or cancer inhibitions.ti,ab. or chemotherapy-induced.ti,ab. or chemotherapies-induced.ti,ab. or anticancer agents.ti,ab. or anticancinerogen.ti,ab. or anticancinerogens.ti,ab. or anticancinerogenic.ti,ab. or anticancinerogenics.ti,ab. or antitumour.ti,ab. or antitumor.ti,ab. or carcinostatic.ti,ab. or carcinostatics.ti,ab. or cancer inhibitor.ti,ab. or cancer inhibitors.ti,ab. or tumor inhibitor.ti,ab. or tumour inhibitor.ti,ab. or tumor inhibitors.ti,ab. or tumour inhibitors.ti,ab. or carcinochemotherapy.ti,ab. or carcinochemotherapies ti,ab.mp. or chemoembolization.ti,ab. or chemoembolizations.ti,ab. or elektrochemotherapy ti,ab.mp. or elektrochemotherapies.ti,ab. or afatinib.ti,ab. or everolimus.ti,ab. or afinitor.ti,ab. or carmustine.ti,ab. or carmustin.ti,ab. or belinostat.ti,ab. or bendamustine.ti,ab. or bicalutamide.ti,ab. or blinatumomab.ti,ab. or brentuximab.ti,ab. or busulfan.ti,ab. or bortezomib.ti,ab. or bevacizumab.ti,ab. or bicatulamide.ti,ab. or bosutinib.ti,ab. or bleomycine.ti,ab. or bleomycin.ti,ab. or blinatumomab.ti,ab. or carbazitaxel.ti,ab. or irinocetan.ti,ab. or carboplatine.ti,ab. or carboplatin.ti,ab. or capecitabine.ti,ab. or capecitabin.ti,ab. or lomustine.ti,ab. or lomustin.ti,ab. or ceritinib.ti,ab. or carfilzomib.ti,ab. or crizotinib.ti,ab. or cyclofosfamide.ti,ab. or cyclophosphamide.ti,ab. or Ifosfamide.ti,ab. or ramucirumab.ti,ab. or cytarabine.ti,ab. or cytarabin.ti,ab. or clorafibine.ti,ab. or clorafibin.ti,ab. or dabrafenib.ti,ab. or dacarbazine.ti,ab. or dacarbazin.ti,ab. or daratumumab.ti,ab. or dasatinib.ti,ab. or daunorubicine.ti,ab. or daunorubicin.ti,ab. or daunomycin.ti,ab. or daunomycine.ti,ab. or decitabine.ti,ab. or decitabin.ti,ab. or denileukin.ti,ab. or docetaxel.ti,ab. or doxorubicine.ti,ab. or doxorubicin.ti,ab. or rasburicase.ti,ab. or epirubicin.ti,ab. or epirubicine.ti,ab. or elotuzumab.ti,ab. or eltrombopag.ti,ab. or enzalutamide.ti,ab. or eribulin.ti,ab. or eribuline.ti,ab. or vismodegib.ti,ab. or erlotinib.ti,ab. or asparaginase.ti,ab. or etoposide.ti,ab. or panobinostat.ti,ab. or fulvestrant.ti,ab. or letrozol.ti,ab. or letrozole.ti,ab. or fludarabine.ti,ab. or fludarabin.ti,ab. or flutamide.ti,ab. or pralatrexate.ti,ab. or obinutuzumab.ti,ab. or gefitinib.ti,ab. or imatinib.ti,ab. or trastuzumab.ti,ab. or emtansine.ti,ab. or topotecan.ti,ab. or pembrolizumab.ti,ab. or lapatinib.ti,ab. or lanreotide.ti,ab. or lenalidomide.ti,ab. or olaparib.ti,ab. or vincristine.ti,ab. or vincristin.ti,ab. or procarbazine.ti,ab. or mustine.ti,ab. or mustargen.ti,ab. or megestrol.ti,ab. or mercaptopurine.ti,ab. or temozolomide.ti,ab. or trametinib.ti,ab. or mitomycine.ti,ab. or mitomycin.ti,ab. or mitoxantrone.ti,ab. or paclitaxel.ti,ab. or vinorelbine.ti,ab. or sorafenib.ti,ab. or nilotinib.ti,ab. or nivolumab.ti,ab. or tamoxifen.ti,ab. or pegaspargase.ti,ab. or oxaliplatin.ti,ab. or oxaliplatine.ti,ab. or panitumumab.ti,ab. or pazopanib.ti,ab. or pertuzumab.ti,ab. or pomalidomide.ti,ab. or ponatinib.ti,ab. or sipuleucel-T.ti,ab. or interferon alfa-2b.ti,ab. or regorafenib.ti,ab. or rituximab.ti,ab. or romidepsin.ti,ab. or sunitinib.ti,ab. or tioguanine.ti,ab. or thioguanine.ti,ab. or thiotepa.ti,ab. or temsirolimus.ti,ab. or thalidomide.ti,ab. or trabectedine.ti,ab. or trabectedin.ti,ab. or vandetanib.ti,ab. or vinblastine.ti,ab. or ipilimumab.ti,ab. or aflibercept.ti,ab. or ibritumomab.ti,ab. or idelalisib.ti,ab. or cetuximab.ti,ab. or anastrozol.ti,ab. or anastrozole.ti,ab. or degarelix.ti,ab. or osimertinib.ti,ab. or ruxolitinib.ti,ab. or palbociclib.ti,ab. or ixazomib.ti,ab. or ofatumumab.ti,ab. or alectinib.ti,ab. or filgrastim.ti,ab. or vorinostat.ti,ab. or talimogene.ti,ab. or romiplostin.ti,ab. or siltuximab.ti,ab. or plerixafor.ti,ab. or azacitidine.ti,ab. or gosereline.ti,ab. or goserelin.ti,ab. or abirateron.ti,ab. or abiraterone.ti,ab. or lenvatinib.ti,ab. or leuproreline.ti,ab. or leuprorelin.ti,ab. or trifluridine.ti,ab. or tipiracil.ti,ab. or necitumumab.ti,ab. or omacetaxine.ti,ab. or chlorambucil.ti,ab. or bexarotene.ti,ab. or mechlorethamine.ti,ab. or mechlorethamine.ti,ab. or omacetaxine.ti,ab. or emtansine.ti,ab. or clorafibine.ti,ab. or dysesthesia.ti,ab. or anticancer drug.ti,ab. or anticancer drugs.ti,ab. or anti-cancer drug.ti,ab. or anti-cancer drugs.ti,ab. or anti-tumor drugs.ti,ab. or anti-tumor drug.ti,ab. or anti-cancer agents.ti,ab. or anti-cancer agent.ti,ab. or anti-tumor agent.ti,ab. or anti-tumor agents.ti,ab. or anti-tumor drug.ti,ab. or anti-tumor drugs.ti,ab. or anti-carcinogenic agents.ti,ab. or anti-carcinogenic agent.ti,ab. or anti-neoplastic.ti,ab. or chemotherapy.ti,ab. or chemo-therapy.ti,ab. or chemo-therapies.ti,ab. or anti- carcinogenic agents.ti,ab. or anti- carcinogenic agent.ti,ab. or anti-carcinogenic drug.ti,ab. or anti-carcinogenic drugs.ti,ab. or anti- carcinogenic drug.ti,ab. or anti- carcinogenic drugs.ti,ab. or anti-mitotic agents.ti,ab. or anti-mitotic agent.ti,ab. or anti-mitotic drugs.ti,ab. or anti-mitotic drug.ti,ab. or anti-mitotics.ti,ab. |
| Polyneuropathy | exp allodynia/ or exp neuropathy/ or exp hyperalgesia/ or exp peripheral neuropathy/ or exp paresthesia/ or exp paraesthesia/ or exp peripheral neuropathy/ or allodynias.ti,ab. or hyperalgesia.ti,ab. or hyperpathia.ti,ab. or allodynia.ti,ab. or neuropathy.ti,ab. or hyperalgesia.ti,ab. or Polyneuropathia.ti,ab. or polyneuropathy.ti,ab. or polyneuropathies.ti,ab. or neuropathy.ti,ab. or neuropathies.ti,ab. or neurotoxicity.ti,ab. or neurotoxicities.ti,ab. or axonopathy.ti,ab. or axonopathies.ti,ab. or nerve lesion.ti,ab. or mononeuropathy.ti,ab. or mononeuropathies.ti,ab. or neurodystrophy.ti,ab. or neuralgia.ti,ab. or neuralgias.ti,ab. or demyelination.ti,ab. or demyelinations.ti,ab. or demyelinisation.ti,ab. or demyelinisations.ti,ab. or demyelogenic.ti,ab. or demyelogenics.ti,ab. or demyelinizing.ti,ab. or demyelinating.ti,ab. or neuritis.ti,ab. or neuropathic pains.ti,ab. or neuropathic pain.ti,ab. or neuropathogenesis.ti,ab. or neuropathogenese.ti,ab. or neuropathology.ti,ab. or neuropathologies.ti,ab. or polyradioneuritis.ti,ab. or polyradiculoneuropathy.ti,ab. or radiculoneuropathy.ti,ab. or polyradiculoneuropathies.ti,ab. or radiculoneuropathies.ti,ab. or polyradiculoneuropathia.ti,ab. or radiculoneuropathia.ti,ab. or polyradiculoneuropathias.ti,ab. or radiculoneuropathias.ti,ab. or nerve lesion.ti,ab. or nerve lesions.ti,ab. or nerve degeneration.ti,ab. or nerve degenerations.ti,ab. or neuralgic pain.ti,ab. Or neuropathic cancer pain.ti,ab. |
| Experimental animal | exp animal experiment/ or exp animal model/ or exp experimental animal/ or exp transgenic animal/ or exp male animal/ or exp female animal/ or exp juvenile animal/ OR animal/ OR chordata/ OR vertebrate/ OR tetrapod/ OR exp fish/ OR amniote/ OR exp amphibia/ OR mammal/ OR exp reptile/ OR exp sauropsid/ OR therian/OR exp monotremate/ OR placental mammals/ OR exp marsupial/ OR Euarchontoglires/ OR exp Afrotheria/ OR exp Boreoeutheria/ OR exp Laurasiatheria/ OR exp Xenarthra/ OR primate/ OR exp Dermoptera/ OR exp Glires/ OR exp Scandentia/ OR Haplorhini/ OR exp prosimian/ OR simian/ OR exp tarsiiform/ OR Catarrhini/ OR exp Platyrrhini/ OR ape/ OR exp Cercopithecidae/ OR hominid/ OR exp hylobatidae/ OR exp chimpanzee/ OR exp gorilla/ OR exp orang utan/ OR (animal OR animals OR pisces OR fish OR fishes OR catfish OR catfishes OR sheatfish OR silurus OR arius OR heteropneustes OR clarias OR gariepinus OR fathead minnow OR fathead minnows OR pimephales OR promelas OR cichlidae OR trout OR trouts OR char OR chars OR salvelinus OR salmo OR oncorhynchus OR guppy OR guppies OR millionfish OR poecilia OR goldfish OR goldfishes OR carassius OR auratus OR mullet OR mullets OR mugil OR curema OR shark OR sharks OR cod OR cods OR gadus OR morhua OR carp OR carps OR cyprinus OR carpio OR killifish OR eel OR eels OR anguilla OR zander OR sander OR lucioperca OR stizostedion OR turbot OR turbots OR psetta OR flatfish OR flatfishes OR plaice OR pleuronectes OR platessa OR tilapia OR tilapias OR oreochromis OR sarotherodon OR common sole OR dover sole OR solea OR zebrafish OR zebrafishes OR danio OR rerio OR seabass OR dicentrarchus OR labrax OR morone OR lamprey OR lampreys OR petromyzon OR pumpkinseed OR pumpkinseeds OR lepomis OR gibbosus OR herring OR clupea OR harengus OR amphibia OR amphibian OR amphibians OR anura OR salientia OR frog OR frogs OR rana OR toad OR toads OR bufo OR xenopus OR laevis OR bombina OR epidalea OR calamita OR salamander OR salamanders OR newt OR newts OR triturus OR reptilia OR reptile OR reptiles OR bearded dragon OR pogona OR vitticeps OR iguana OR iguanas OR lizard OR lizards OR anguis fragilis OR turtle OR turtles OR snakes OR snake OR aves OR bird OR birds OR quail OR quails OR coturnix OR bobwhite OR colinus OR virginianus OR poultry OR poultries OR fowl OR fowls OR chicken OR chickens OR gallus OR zebra finch OR taeniopygia OR guttata OR canary OR canaries OR serinus OR canaria OR parakeet OR parakeets OR grasskeet OR parrot OR parrots OR psittacine OR psittacines OR shelduck OR tadorna OR goose OR geese OR branta OR leucopsis OR woodlark OR lullula OR flycatcher OR ficedula OR hypoleuca OR dove OR doves OR geopelia OR cuneata OR duck OR ducks OR greylag OR graylag OR anser OR harrier OR circus pygargus OR red knot OR great knot OR calidris OR canutus OR godwit OR limosa OR lapponica OR meleagris OR gallopavo OR jackdaw OR corvus OR monedula OR ruff OR philomachus OR pugnax OR lapwing OR peewit OR plover OR vanellus OR swan OR cygnus OR columbianus OR bewickii OR gull OR chroicocephalus OR ridibundus OR albifrons OR great tit OR parus OR aythya OR fuligula OR streptopelia OR risoria OR spoonbill OR platalea OR leucorodia OR blackbird OR turdus OR merula OR blue tit OR cyanistes OR pigeon OR pigeons OR columba OR pintail OR anas OR starling OR sturnus OR owl OR athene noctua OR pochard OR ferina OR cockatiel OR nymphicus OR hollandicus OR skylark OR alauda OR tern OR sterna OR teal OR crecca OR oystercatcher OR haematopus OR ostralegus OR shrew OR shrews OR sorex OR araneus OR crocidura OR russula OR european mole OR talpa OR chiroptera OR bat OR bats OR eptesicus OR serotinus OR myotis OR dasycneme OR daubentonii OR pipistrelle OR pipistrellus OR cat OR cats OR felis OR catus OR feline OR dog OR dogs OR canis OR canine OR canines OR otter OR otters OR lutra OR badger OR badgers OR meles OR fitchew OR fitch OR foumart or foulmart OR ferrets OR ferret OR polecat OR polecats OR mustela OR putorius OR weasel OR weasels OR fox OR foxes OR vulpes OR common seal OR phoca OR vitulina OR grey seal OR halichoerus OR horse OR horses OR equus OR equine OR equidae OR donkey OR donkeys OR mule OR mules OR pig OR pigs OR swine OR swines OR hog OR hogs OR boar OR boars OR porcine OR piglet OR piglets OR sus OR scrofa OR llama OR llamas OR lama OR glama OR deer OR deers OR cervus OR elaphus OR cow OR cows OR bos taurus OR bos indicus OR bovine OR bull OR bulls OR cattle OR bison OR bisons OR sheep OR sheeps OR ovis aries OR ovine OR lamb OR lambs OR mouflon OR mouflons OR goat OR goats OR capra OR caprine OR chamois OR rupicapra OR leporidae OR lagomorpha OR lagomorph OR rabbit OR rabbits OR oryctolagus OR cuniculus OR laprine OR hares OR lepus OR rodentia OR rodent OR rodents OR murinae OR mouse OR mice OR mus OR musculus OR murine OR woodmouse OR apodemus OR rat OR rats OR rattus OR norvegicus OR guinea pig OR guinea pigs OR cavia OR porcellus OR hamster OR hamsters OR mesocricetus OR cricetulus OR cricetus OR gerbil OR gerbils OR jird OR jirds OR meriones OR unguiculatus OR jerboa OR jerboas OR jaculus OR chinchilla OR chinchillas OR beaver OR beavers OR castor fiber OR castor canadensis OR sciuridae OR squirrel OR squirrels OR sciurus OR chipmunk OR chipmunks OR marmot OR marmots OR marmota OR suslik OR susliks OR spermophilus OR cynomys OR cottonrat OR cottonrats OR sigmodon OR vole OR voles OR microtus OR myodes OR glareolus OR primate OR primates OR prosimian OR prosimians OR lemur OR lemurs OR lemuridae OR loris OR bush baby OR bush babies OR bushbaby OR bushbabies OR galago OR galagos OR anthropoidea OR anthropoids OR simian OR simians OR monkey OR monkeys OR marmoset OR marmosets OR callithrix OR cebuella OR tamarin OR tamarins OR saguinus OR leontopithecus OR squirrel monkey OR squirrel monkeys OR saimiri OR night monkey OR night monkeys OR owl monkey OR owl monkeys OR douroucoulis OR aotus OR spider monkey OR spider monkeys OR ateles OR baboon OR baboons OR papio OR rhesus monkey OR macaque OR macaca OR mulatta OR cynomolgus OR fascicularis OR green monkey OR green monkeys OR chlorocebus OR vervet OR vervets OR pygerythrus OR hominoidea OR ape OR apes OR hylobatidae OR gibbon OR gibbons OR siamang OR siamangs OR nomascus OR symphalangus OR hominidae OR orangutan OR orangutans OR pongo OR chimpanzee OR chimpanzees OR pan troglodytes OR bonobo OR bonobos OR pan paniscus OR gorilla OR gorillas OR troglodytes).ti,ab. |
