# Supplementary material for: A systematic summary and comparison of animal models for chemotherapy induced (peripheral) neuropathy (CIPN)
Source: PLoS One. 2019 Aug 28;14(8):e0221787. doi: 10.1371/journal.pone.0221787 (PMC6713358; doi:10.1371/journal.pone.0221787)
Supplement: S1 Table — * chemotherapy-induced polyneuropathy in one outcome measurement. † chemotherapy-induced polyneuropathy in more than one outcome measurement. (DOCX) [file pone.0221787.s005.docx]

**S1 Table: Overview of the efficacy of CIPN models ranked by animal species**

| **Species** | **Strain** | **Chemotherapy** | **Sex** | **n^*^** | **CIPN^*^** | **n^†^** | **CIPN^†^** |
| --- | --- | --- | --- | --- | --- | --- | --- |
| Catfish | Ictalurus nebulosus | Vincristine |  | 1 | 1 (100%) | 1 | 1 (100%) |
| Cats | Mongrel | Vincristine | Both | 2 | 1 (50%) | 0 |  |
| Cats |  | Vinblastine |  | 1 | 1 (100%) | 0 |  |
| Cats |  | Vincristine |  | 3 | 3 (100%) | 0 |  |
| Cats |  | Vindesine |  | 1 | 1 (100%) | 0 |  |
| Chicken | White Rock | Vinblastine | Both | 1 | 0 (0%) | 0 |  |
| Chicken | White Rock | Vincristine | Both | 1 | 0 (0%) | 0 |  |
| Chicken | White Rock | Vindesine | Both | 1 | 0 (0%) | 0 |  |
| Dogs | Beagle | Thalidomide | Male | 3 | 0 (0%) | 3 | 0 (0%) |
| Dogs | Mongrel | Ethoglucid |  | 1 | 1 (100%) | 0 |  |
| Drosophila melanogaster | Canton Special | Paclitaxel | Both | 1 | 1 (100%) | 0 |  |
| Drosophila melanogaster | Oregon Red | Cisplatin |  | 10 | 6 (60%) | 0 |  |
| Drosophila melanogaster |  | Cisplatin |  | 2 | 2 (100%) | 2 | 2 (100%) |
| Guinea pigs |  | Cisplatin | Male | 1 | 1 (100%) | 0 |  |
| Guinea pigs |  | Vinblastine | Both | 1 | 0 (0%) | 0 |  |
| Guinea pigs |  | Vincristine | Both | 1 | 1 (100%) | 0 |  |
| Guinea pigs |  | Vincristine | Male | 1 | 1 (100%) | 1 | 1 (100%) |
| Guinea pigs |  | Vindesine | Both | 1 | 0 (0%) | 0 |  |
| Guinea pigs |  | Vindesine | Male | 1 | 1 (100%) | 0 |  |
| Mice | 129P3 | Paclitaxel | Both | 1 | 1 (100%) | 0 |  |
| Mice | AJ | Paclitaxel | Both | 1 | 1 (100%) | 0 |  |
| Mice | AJ | Paclitaxel | Female | 2 | 2 (100%) | 1 | 1 (100%) |
| Mice | AJ | Paclitaxel | Male | 2 | 2 (100%) | 2 | 2 (100%) |
| Mice | AKR | Paclitaxel | Both | 1 | 1 (100%) | 0 |  |
| Mice | Athymic nude | Bortezomib | Female | 2 | 2 (100%) | 2 | 1 (50%) |
| Mice | Athymic nude | Cisplatin | Female | 1 | 1 (100%) | 1 | 1 (100%) |
| Mice | Athymic nude | Paclitaxel | Female | 3 | 3 (100%) | 2 | 2 (100%) |
| Mice | BALB/c | Bortezomib | Female | 3 | 2 (67%) | 2 | 2 (100%) |
| Mice | BALB/c | Cisplatin | Female | 2 | 2 (100%) | 2 | 2 (100%) |
| Mice | BALB/c | Epothilone-B | Female | 2 | 2 (100%) | 1 | 0 (0%) |
| Mice | BALB/c | Eribulin | Female | 3 | 3 (100%) | 1 | 1 (100%) |
| Mice | BALB/c | Gemcitabine | Male | 1 | 0 (0%) | 0 |  |
| Mice | BALB/c | Ixabepilone | Female | 2 | 2 (100%) | 1 | 1 (100%) |
| Mice | BALB/c | Oxaliplatin |  | 1 | 1 (100%) | 0 |  |
| Mice | BALB/c | Oxaliplatin | Female | 3 | 3 (100%) | 2 | 2 (100%) |
| Mice | BALB/c | Oxaliplatin | Male | 10 | 8 (80%) | 3 | 2 (67%) |
| Mice | BALB/c | Paclitaxel | Female | 19 | 17 (89%) | 8 | 6 (75%) |
| Mice | BALB/c | Paclitaxel | Male | 3 | 3 (100%) | 1 | 1 (100%) |
| Mice | BALB/c | Vinorelbine | Female | 1 | 1 (100%) | 1 | 1 (100%) |
| Mice | BDF1 | Paclitaxel | Male | 1 | 1 (100%) | 0 |  |
| Mice | BDF1 | Vincristine | Female | 5 | 3 (60%) | 0 |  |
| Mice | BDF1 | Vincristine | Male | 1 | 1 (100%) | 0 |  |
| Mice | C3H | Cisplatin | Male | 5 | 4 (80%) | 3 | 3 (100%) |
| Mice | C3H | Paclitaxel | Both | 1 | 1 (100%) | 0 |  |
| Mice | C57BL/10 | Paclitaxel | Both | 1 | 1 (100%) | 0 |  |
| Mice | C57BL/6 | Bortezomib |  | 1 | 1 (100%) | 1 | 1 (100%) |
| Mice | C57BL/6 | Bortezomib | Female | 1 | 1 (100%) | 0 |  |
| Mice | C57BL/6 | Bortezomib | Male | 5 | 5 (100%) | 3 | 3 (100%) |
| Mice | C57BL/6 | Cisplatin |  | 1 | 1 (100%) | 1 | 1 (100%) |
| Mice | C57BL/6 | Cisplatin | Both | 1 | 1 (100%) | 1 | 1 (100%) |
| Mice | C57BL/6 | Cisplatin | Female | 5 | 5 (100%) | 1 | 1 (100%) |
| Mice | C57BL/6 | Cisplatin | Male | 22 | 20 (91%) | 14 | 12 (86%) |
| Mice | C57BL/6 | Oxaliplatin |  | 1 | 1 (100%) | 0 |  |
| Mice | C57BL/6 | Oxaliplatin | Male | 50 | 48 (96%) | 32 | 28 (88%) |
| Mice | C57BL/6 | Paclitaxel |  | 1 | 1 (100%) | 1 | 1 (100%) |
| Mice | C57BL/6 | Paclitaxel | Both | 7 | 7 (100%) | 2 | 2 (100%) |
| Mice | C57BL/6 | Paclitaxel | Female | 13 | 13 (100%) | 8 | 8 (100%) |
| Mice | C57BL/6 | Paclitaxel | Male | 32 | 29 (91%) | 19 | 17 (89%) |
| Mice | C57BL/6 | Salinomycin | Male | 1 | 1 (100%) | 1 | 1 (100%) |
| Mice | C57BL/6 | Tamoxifen | Male | 1 | 1 (100%) | 0 |  |
| Mice | C57BL/6 | Vinblastine | Male | 1 | 1 (100%) | 1 | 1 (100%) |
| Mice | C57BL/6 | Vincristine |  | 4 | 3 (75%) | 0 |  |
| Mice | C57BL/6 | Vincristine | Both | 2 | 2 (100%) | 2 | 2 (100%) |
| Mice | C57BL/6 | Vincristine | Female | 1 | 1 (100%) | 1 | 1 (100%) |
| Mice | C57BL/6 | Vincristine | Male | 11 | 11 (100%) | 7 | 5 (71%) |
| Mice | CBA | Oxaliplatin |  | 1 | 1 (100%) | 0 |  |
| Mice | CBA | Paclitaxel | Both | 1 | 1 (100%) | 0 |  |
| Mice | CD1 | Cisplatin | Male | 3 | 3 (100%) | 1 | 1 (100%) |
| Mice | CD1 | Etoposide | Female | 2 | 2 (100%) | 0 |  |
| Mice | CD1 | Oxaliplatin |  | 1 | 1 (100%) | 0 |  |
| Mice | CD1 | Oxaliplatin | Male | 5 | 5 (100%) | 3 | 3 (100%) |
| Mice | CD1 | Paclitaxel | Both | 2 | 2 (100%) | 2 | 2 (100%) |
| Mice | CD1 | Paclitaxel | Female | 2 | 2 (100%) | 2 | 2 (100%) |
| Mice | CD1 | Paclitaxel | Male | 9 | 9 (100%) | 6 | 6 (100%) |
| Mice | CD1 | Vincristine |  | 1 | 1 (100%) | 0 |  |
| Mice | CD1 | Vincristine | Male | 2 | 2 (100%) | 2 | 2 (100%) |
| Mice | DBA/2 | Paclitaxel |  | 2 | 2 (100%) | 2 | 1 (50%) |
| Mice | DBA/2 | Paclitaxel | Both | 1 | 1 (100%) | 0 |  |
| Mice | ddY | Oxaliplatin | Male | 4 | 4 (100%) | 4 | 3 (75%) |
| Mice | ddY | Paclitaxel | Male | 17 | 13 (76%) | 1 | 1 (100%) |
| Mice | ddY | Vincristine | Male | 5 | 4 (80%) | 2 | 0 (0%) |
| Mice | ICR | Gemcitabine | Male | 1 | 0 (0%) | 0 |  |
| Mice | ICR | Oxaliplatin | Male | 5 | 5 (100%) | 2 | 2 (100%) |
| Mice | ICR | Paclitaxel |  | 1 | 1 (100%) | 1 | 1 (100%) |
| Mice | ICR | Paclitaxel | Male | 6 | 6 (100%) | 0 |  |
| Mice | ICR | Tamoxifen | Female | 2 | 2 (100%) | 1 | 1 (100%) |
| Mice | ICR | Vincristine |  | 2 | 2 (100%) | 2 | 2 (100%) |
| Mice | ICR | Vincristine | Male | 11 | 8 (73%) | 3 | 2 (67%) |
| Mice | NMRI | Cisplatin | Male | 2 | 2 (100%) | 0 |  |
| Mice | NMRI | Paclitaxel |  | 1 | 1 (100%) | 1 | 1 (100%) |
| Mice | NMRI | Vincristine | Male | 1 | 1 (100%) | 0 |  |
| Mice | OF1 | Bortezomib | Female | 1 | 1 (100%) | 1 | 1 (100%) |
| Mice | OF1 | Cisplatin |  | 1 | 1 (100%) | 0 |  |
| Mice | RIIIS | Paclitaxel | Both | 1 | 1 (100%) | 0 |  |
| Mice | SKH1-Hairless | Paclitaxel | Male | 1 | 1 (100%) | 0 |  |
| Mice | SM | Paclitaxel | Both | 1 | 1 (100%) | 0 |  |
| Mice | Swiss | Bortezomib | Female | 1 | 1 (100%) | 1 | 1 (100%) |
| Mice | Swiss | Cisplatin | Female | 4 | 4 (100%) | 4 | 3 (75%) |
| Mice | Swiss | Cisplatin | Male | 1 | 1 (100%) | 0 |  |
| Mice | Swiss | Doxorubicin | Male | 1 | 1 (100%) | 0 |  |
| Mice | Swiss | Oxaliplatin | Male | 3 | 3 (100%) | 3 | 3 (100%) |
| Mice | Swiss | Paclitaxel | Male | 10 | 10 (100%) | 4 | 3 (75%) |
| Mice | Swiss | Vincristine | Both | 1 | 1 (100%) | 0 |  |
| Mice | Swiss | Vincristine | Female | 2 | 2 (100%) | 2 | 2 (100%) |
| Mice | Swiss | Vincristine | Male | 1 | 1 (100%) | 1 | 1 (100%) |
| Mice |  | Cisplatin | Male | 1 | 1 (100%) | 1 | 1 (100%) |
| Mice |  | Oxaliplatin |  | 1 | 1 (100%) | 1 | 1 (100%) |
| Mice |  | Oxaliplatin | Male | 1 | 1 (100%) | 1 | 1 (100%) |
| Mice |  | Paclitaxel |  | 1 | 1 (100%) | 0 |  |
| Mice |  | Paclitaxel | Both | 1 | 1 (100%) | 1 | 1 (100%) |
| Mice |  | Vincristine | Both | 1 | 1 (100%) | 1 | 1 (100%) |
| Cynomolgus monkey |  | Cisplatin | Female | 1 | 1 (100%) | 1 | 0 (0%) |
| Cynomolgus monkey |  | Cisplatin | Male | 1 | 0 (0%) | 1 | 0 (0%) |
| Cynomolgus monkey |  | Oxaliplatin | Female | 4 | 3 (75%) | 0 |  |
| Rhesus monkey |  | Vincristine | Male | 1 | 1 (100%) | 0 |  |
| Rabbits | New Zealand | Vincristine | Both | 1 | 1 (100%) | 0 |  |
| Rabbits | New Zealand | Vincristine | Male | 5 | 5 (100%) | 0 |  |
| Rabbits |  | Vincristine |  | 2 | 2 (100%) | 0 |  |
| Rats | Dark Agouti | Cisplatin |  | 1 | 1 (100%) | 0 |  |
| Rats | Dark Agouti | Cisplatin | Female | 2 | 2 (100%) | 2 | 1 (50%) |
| Rats | Dark Agouti | Paclitaxel |  | 1 | 1 (100%) | 0 |  |
| Rats | Dark Agouti | Paclitaxel | Female | 1 | 1 (100%) | 1 | 0 (0%) |
| Rats | Dark Agouti | Vincristine | Female | 2 | 2 (100%) | 2 | 2 (100%) |
| Rats | Egyptian | Vincristine | Female | 1 | 1 (100%) | 1 | 1 (100%) |
| Rats | Fisher344 | Cisplatin | Female | 1 | 1 (100%) | 1 | 1 (100%) |
| Rats | Fisher344 | Docetaxel | Female | 1 | 1 (100%) | 1 | 1 (100%) |
| Rats | Fisher344 | Docetaxel | Male | 1 | 1 (100%) | 1 | 1 (100%) |
| Rats | Fisher344 | Paclitaxel | Both | 1 | 1 (100%) | 0 |  |
| Rats | Fisher344 | Paclitaxel | Female | 1 | 1 (100%) | 1 | 1 (100%) |
| Rats | Holtzman | Vincristine | Male | 1 | 1 (100%) | 0 |  |
| Rats | Lewis | Paclitaxel | Both | 1 | 1 (100%) | 0 |  |
| Rats | Lewis | Vincristine | Female | 4 | 3 (75%) | 3 | 0 (0%) |
| Rats | Long Evans | Paclitaxel | Male | 1 | 1 (100%) | 0 |  |
| Rats | Sprague-Dawley | Bortezomib | Male | 21 | 18 (86%) | 13 | 7 (54%) |
| Rats | Sprague-Dawley | Cisplatin | Both | 1 | 1 (100%) | 1 | 1 (100%) |
| Rats | Sprague-Dawley | Cisplatin | Female | 4 | 4 (100%) | 2 | 1 (50%) |
| Rats | Sprague-Dawley | Cisplatin | Male | 37 | 34 (92%) | 29 | 21 (72%) |
| Rats | Sprague-Dawley | Docetaxel | Male | 2 | 2 (100%) | 2 | 2 (100%) |
| Rats | Sprague-Dawley | Doxorubicin | Male | 2 | 2 (100%) | 0 |  |
| Rats | Sprague-Dawley | Methotrexate | Male | 1 | 1 (100%) | 0 |  |
| Rats | Sprague-Dawley | Nab-paclitaxel | Male | 2 | 2 (100%) | 2 | 2 (100%) |
| Rats | Sprague-Dawley | Oxaliplatin | Both | 1 | 1 (100%) | 1 | 1 (100%) |
| Rats | Sprague-Dawley | Oxaliplatin | Male | 104 | 104 (100%) | 62 | 57 (92%) |
| Rats | Sprague-Dawley | Paclitaxel | Both | 2 | 2 (100%) | 1 | 1 (100%) |
| Rats | Sprague-Dawley | Paclitaxel | Female | 15 | 14 (93%) | 6 | 1 (17%) |
| Rats | Sprague-Dawley | Paclitaxel | Male | 120 | 119 (99%) | 64 | 57 (89%) |
| Rats | Sprague-Dawley | Quelamycin | Male | 2 | 2 (100%) | 0 |  |
| Rats | Sprague-Dawley | Sorafenib | Male | 5 | 5 (100%) | 5 | 0 (0%) |
| Rats | Sprague-Dawley | Suramin | Male | 2 | 2 (100%) | 2 | 2 (100%) |
| Rats | Sprague-Dawley | Thalidomide | Male | 1 | 1 (100%) | 1 | 0 (0%) |
| Rats | Sprague-Dawley | Vinblastine | Male | 1 | 1 (100%) | 1 | 0 (0%) |
| Rats | Sprague-Dawley | Vincristine |  | 1 | 1 (100%) | 1 | 1 (100%) |
| Rats | Sprague-Dawley | Vincristine | Both | 3 | 1 (33%) | 3 | 1 (33%) |
| Rats | Sprague-Dawley | Vincristine | Female | 4 | 4 (100%) | 2 | 2 (100%) |
| Rats | Sprague-Dawley | Vincristine | Male | 65 | 65 (100%) | 40 | 33 (83%) |
| Rats | Wistar | Bortezomib | Female | 12 | 9 (75%) | 12 | 9 (75%) |
| Rats | Wistar | Carboplatin | Female | 3 | 3 (100%) | 2 | 2 (100%) |
| Rats | Wistar | Cisplatin |  | 3 | 3 (100%) | 0 |  |
| Rats | Wistar | Cisplatin | Female | 37 | 35 (95%) | 19 | 18 (95%) |
| Rats | Wistar | Cisplatin | Male | 23 | 21 (91%) | 9 | 6 (67%) |
| Rats | Wistar | Docetaxel |  | 1 | 1 (100%) | 1 | 1 (100%) |
| Rats | Wistar | Docetaxel | Female | 3 | 3 (100%) | 1 | 0 (0%) |
| Rats | Wistar | Doxorubicin | Female | 1 | 0 (0%) | 0 |  |
| Rats | Wistar | Doxorubicin | Male | 1 | 1 (100%) | 1 | 1 (100%) |
| Rats | Wistar | Lipoplatin | Female | 2 | 2 (100%) | 2 | 2 (100%) |
| Rats | Wistar | Ormaplatin | Male | 1 | 1 (100%) | 0 |  |
| Rats | Wistar | Oxaliplatin | Both | 2 | 2 (100%) | 2 | 2 (100%) |
| Rats | Wistar | Oxaliplatin | Female | 16 | 13 (81%) | 9 | 8 (89%) |
| Rats | Wistar | Oxaliplatin | Male | 22 | 18 (82%) | 11 | 8 (73%) |
| Rats | Wistar | Paclitaxel |  | 2 | 2 (100%) | 1 | 0 (0%) |
| Rats | Wistar | Paclitaxel | Female | 15 | 14 (93%) | 9 | 6 (67%) |
| Rats | Wistar | Paclitaxel | Male | 22 | 22 (100%) | 16 | 13 (81%) |
| Rats | Wistar | Vinblastine | Both | 3 | 2 (67%) | 3 | 2 (67%) |
| Rats | Wistar | Vincristine |  | 1 | 1 (100%) | 1 | 1 (100%) |
| Rats | Wistar | Vincristine | Both | 10 | 10 (100%) | 10 | 10 (100%) |
| Rats | Wistar | Vincristine | Male | 8 | 8 (100%) | 4 | 4 (100%) |
| Rats |  | Bortezomib | Male | 1 | 1 (100%) | 1 | 1 (100%) |
| Rats |  | Cisplatin |  | 1 | 1 (100%) | 0 |  |
| Rats |  | Cisplatin | Male | 1 | 1 (100%) | 1 | 1 (100%) |
| Rats |  | Oxaliplatin |  | 1 | 1 (100%) | 1 | 1 (100%) |
| Rats |  | Paclitaxel |  | 1 | 1 (100%) | 0 |  |
| Rats |  | Vincristine | Male | 1 | 1 (100%) | 1 | 1 (100%) |
| Zebrafish |  | Paclitaxel |  | 1 | 1 (100%) | 0 |  |

^*^ chemotherapy-induced polyneuropathy in one outcome measurement
^†^ chemotherapy-induced polyneuropathy in more than one outcome measurement
