# Supplementary material for: A systematic summary and comparison of animal models for chemotherapy induced (peripheral) neuropathy (CIPN)
Source: PLoS One. 2019 Aug 28;14(8):e0221787. doi: 10.1371/journal.pone.0221787 (PMC6713358; doi:10.1371/journal.pone.0221787)
Supplement: S4 Table — (DOCX) [file pone.0221787.s008.docx]

# S4 Table; Quality of reporting

| **Author(s)** | **Year** | **Title** | **Blinding** | **Randomisation** | **Sample Size** |
| --- | --- | --- | --- | --- | --- |
| A. N. A. Abad, M. H. K. Nouri, A. Gharjanie, F. Tavakoli | 2011 | Effect of Matricaria chamomilla Hydroalcoholic Extract on Cisplatin-induced Neuropathy in Mice | No | Yes | No |
| A. Abed, M. J. Khoshnoud, M. Taghian, M. Aliasgharzadeh, A. Mesdaghinia | 2017 | Quetiapine reverses paclitaxel-induced neuropathic pain in mice: role of alpha2- adrenergic receptors | No | Yes | No |
| M. Abram, M. Zagaja, S. Mogilski, et al | 2017 | Multifunctional Hybrid Compounds Derived from 2-(2,5-Dioxopyrrolidin-1-yl)-3-methoxypropanamides with Anticonvulsant and Antinociceptive Properties | No | Yes | No |
| P. Adamek, C. E. Tatsui, L. D. Rhines, et al | 2015 | The cancer chemotherapeutic paclitaxel increases human and rodent sensory neuron responses to TRPV1 by activation of TLR4 | Yes | No | No |
| N. Ahmad, F. Subhan, N. U. Islam, M. Shahid, F. U. Rahman, R. D. E. Sewell | 2017 | Gabapentin and its salicylaldehyde derivative alleviate allodynia and hypoalgesia in a cisplatin-induced neuropathic pain model | Yes | Yes | No |
| S. H. Ahn, I. A. Chang, K. J. Kim, C. J. Kim, U. Namgung, C. S. Cho | 2016 | Bogijetong decoction and its active herbal components protect the peripheral nerve from damage caused by taxol or nerve crush | No | Yes | No |
| T. Akman, L. Akman, O. Erbas, M. C. Terek, D. Taskiran, A. Ozsaran | 2015 | The preventive effect of oxytocin to Cisplatin-induced neurotoxicity: an experimental rat model | No | No | No |
| M. S. Al Moundhri, S. Al-Salam, A. Al Mahrouqee, S. Beegam, B. H. Ali | 2013 | The effect of curcumin on oxaliplatin and cisplatin neurotoxicity in rats: some behavioral, biochemical, and histopathological studies | Yes | Yes | No |
| A. Alaedini, Z. Xiang, H. Kim, Y. J. Sung, N. Latov | 2008 | Up-regulation of apoptosis and regeneration genes in the dorsal root ganglia during cisplatin treatment | No | Yes | No |
| A. Ale, J. Bruna, A. Calls, et al | 2016 | Inhibition of the neuronal NFkappaB pathway attenuates bortezomib-induced neuropathy in a mouse model | No | No | No |
| A. Ale, J. Bruna, M. Morell, et al | 2014 | Treatment with anti-TNF alpha protects against the neuropathy induced by the proteasome inhibitor bortezomib in a mouse model | No | No | No |
| N. Alessandri-Haber, O. A. Dina, E. K. Joseph, D. B. Reichling, J. D. Levine | 2008 | Interaction of transient receptor potential vanilloid 4, integrin, and Src tyrosine kinase in mechanical hyperalgesia | No | No | No |
| N. Alessandri-Haber, O. A. Dina, J. J. Yeh, C. A. Parada, D. B. Reichling, J. D. Levine | 2004 | Transient receptor potential vanilloid 4 is essential in chemotherapy-induced neuropathic pain in the rat | No | No | No |
| T. Alexa, A. Luca, C. Bohotin, D. Lupchian, I. Stanciuc, M. Badescu | 2015 | The Effect of Cobalt Chloride Preconditioning on Paclitaxel-Induced Peripheral Neuropathy | No | No | No |
| K. O. Aley, J. D. Levine | 2002 | Different peripheral mechanisms mediate enhanced nociception in metabolic/toxic and traumatic painful peripheral neuropathies in the rat | No | No | No |
| K. O. Aley, D. B. Reichling, J. D. Levine | 1996 | Vincristine hyperalgesia in the rat: A model of painful vincristine neuropathy in humans | No | No | No |
| H. Alimoradi, N. Pourmohammadi, S. E. Mehr, et al | 2012 | Effects of lithium on peripheral neuropathy induced by vincristine in rats | No | Yes | No |
| L. Aloe, L. Manni, F. Properzi, S. De Santis, M. Fiore | 2000 | Evidence that nerve growth factor promotes the recovery of peripheral neuropathy induced in mice by cisplatin: behavioral, structural and biochemical analysis | Yes | No | No |
| P. Alvarez, L. F. Ferrari, J. D. Levine | 2011 | Muscle pain in models of chemotherapy-induced and alcohol-induced peripheral neuropathy | No | Yes | No |
| E. O. Ameyaw, J. N. Boampong, K. E. Kukuia, et al | 2013 | Effect of xylopic acid on paclitaxel-induced neuropathic pain in rats | No | No | No |
| E. O. Ameyaw, E. Woode, E. Boakye-Gyasi, W. K. Abotsi, J. O. Kyekyeku, R. K. Adosraku | 2014 | Anti-allodynic and Anti-hyperalgesic effects of an ethanolic extract and xylopic acid from the fruits of Xylopia aethiopica in murine models of neuropathic pain | No | No | No |
| N. Ami, K. Okamoto, H. Oshima | 2012 | Analgesic effect of magnetic stimulation on paclitaxel-induced peripheral neuropathic pain in mice | No | No | No |
| P. Amoateng, S. Adjei, D. Osei-Safo, et al | 2015 | A hydro-ethanolic extract of Synedrella nodiflora (L.) Gaertn ameliorates hyperalgesia and allodynia in vincristine-induced neuropathic pain in rats | No | No | No |
| P. Amoateng, S. Adjei, D. Osei-Safo, et al | 2017 | Analgesic effects of a hydro-ethanolic whole plant extract of Synedrella nodiflora (L.) Gaertn in paclitaxel-induced neuropathic pain in rats | No | No | No |
| T. Andoh, R. Kitamura, Y. Kuraishi | 2015 | Milnacipran inhibits oxaliplatin-induced mechanical allodynia through spinal action in mice | No | No | No |
| T. Andoh, N. Kobayashi, D. Uta, Y. Kuraishi | 2017 | Prophylactic topical paeoniflorin prevents mechanical allodynia caused by paclitaxel in mice through adenosine A1 receptors | Yes | No | No |
| T. Andoh, A. Sakamoto, Y. Kuraishi | 2013 | Effects of xaliproden, a 5-HT1A agonist, on mechanical allodynia caused by chemotherapeutic agents in mice | No | No | No |
| T. Andoh, D. Uta, M. Kato, K. Toume, K. Komatsu, Y. Kuraishi | 2017 | Prophylactic administration of aucubin inhibits paclitaxel-induced mechanical allodynia via the inhibition of endoplasmic reticulum stress in peripheral Schwann cells | No | No | No |
| M. Aoki, Y. Kurauchi, A. Mori, T. Nakahara, K. Sakamoto, K. Ishii | 2014 | Comparison of the effects of single doses of elcatonin and pregabalin on oxaliplatin-induced cold and mechanical allodynia in rats | Yes | No | No |
| M. Aoki, A. Mori, T. Nakahara, K. Sakamoto, K. Ishii | 2012 | Effect of synthetic eel calcitonin, elcatonin, on cold and mechanical allodynia induced by oxaliplatin and paclitaxel in rats | Yes | No | No |
| M. Aoki, A. Mori, T. Nakahara, K. Sakamoto, K. Ishii | 2013 | Salmon calcitonin reduces oxaliplatin-induced cold and mechanical allodynia in rats | No | No | No |
| M. Aouad, A. Charlet, J. L. Rodeau, P. Poisbeau | 2009 | Reduction and prevention of vincristine-induced neuropathic pain symptoms by the non-benzodiazepine anxiolytic etifoxine are mediated by 3alpha-reduced neurosteroids | No | No | No |
| S. C. Apfel, J. C. Arezzo, M. E. Lewis, J. A. Kessler | 1993 | The use of insulin-like growth factor I in the prevention of vincristine neuropathy in mice | No | No | No |
| S. C. Apfel, J. C. Arezzo, L. Lipson, J. A. Kessler | 1992 | Nerve growth factor prevents experimental cisplatin neuropathy | Yes | No | No |
| S. C. Apfel, R. B. Lipton, J. C. Arezzo, J. A. Kessler | 1991 | Nerve growth factor prevents toxic neuropathy in mice | Yes | No | No |
| A. Areti, P. Komirishetty, M. Akuthota, R. A. Malik, A. Kumar | 2017 | Melatonin prevents mitochondrial dysfunction and promotes neuroprotection by inducing autophagy during oxaliplatin-evoked peripheral neuropathy | Yes | Yes | No |
| A. Areti, P. Komirishetty, A. Kumar | 2017 | Carvedilol prevents functional deficits in peripheral nerve mitochondria of rats with oxaliplatin-evoked painful peripheral neuropathy | No | Yes | No |
| O. Arrieta, N. Hernandez-Pedro, M. C. Fernandez-Gonzalez-Aragon, et al | 2011 | Retinoic acid reduces chemotherapy-induced neuropathy in an animal model and patients with lung cancer | Yes | Yes | No |
| N. Authier, J. Fialip, A. Eschalier, F. Coudore | 2000 | Assessment of allodynia and hyperalgesia after cisplatin administration to rats | No | Yes | No |
| N. Authier, J. P. Gillet, J. Fialip, A. Eschalier, F. Coudore | 2000 | Description of a short-term Taxol-induced nociceptive neuropathy in rats | No | Yes | No |
| N. Authier, J. P. Gillet, J. Fialip, A. Eschalier, F. Coudore | 2003 | A new animal model of vincristine-induced nociceptive peripheral neuropathy | No | Yes | No |
| N. Authier, J. P. Gillet, J. Fialip, A. Eschalier, F. Coudore | 2003 | An animal model of nociceptive peripheral neuropathy following repeated cisplatin injections | Yes | Yes | No |
| M. I. Azevedo, A. F. Pereira, R. B. Nogueira, et al | 2013 | The antioxidant effects of the flavonoids rutin and quercetin inhibit oxaliplatin-induced chronic painful peripheral neuropathy | Yes | No | No |
| A. Babu, K. G. Prasanth, B. Balaji | 2015 | Effect of curcumin in mice model of vincristine-induced neuropathy | No | No | No |
| M. A. Bahar, T. Andoh, K. Ogura, Y. Hayakawa, I. Saiki, Y. Kuraishi | 2013 | Herbal medicine goshajinkigan prevents paclitaxel-induced mechanical allodynia without impairing antitumor activity of paclitaxel | No | No | No |
| D. Balayssac, A. Cayre, N. Authier, et al | 2005 | Patterns of P-glycoprotein activity in the nervous system during vincristine-induced neuropathy in rats | No | Yes | No |
| D. Balayssac, A. Cayre, N. Authier, et al | 2006 | Involvement of the multidrug resistance transporters in cisplatin-induced neuropathy in rats. Comparison with the chronic constriction injury model and monoarthritic rats | No | Yes | No |
| D. Balayssac, A. Cayre, B. Ling, et al | 2008 | Vincristine-induced neuropathy in the rat is not modified by drug-drug interactions with the P-glycoprotein inhibitor verapamil | No | Yes | No |
| D. Balayssac, A. Cayre, B. Ling, et al | 2009 | Increase in morphine antinociceptive activity by a P-glycoprotein inhibitor in cisplatin-induced neuropathy | Yes | Yes | No |
| D. Balayssac, B. Ling, J. Ferrier, B. Pereira, A. Eschalier, N. Authier | 2014 | Assessment of thermal sensitivity in rats using the thermal place preference test: Description and application in the study of oxaliplatin-induced acute thermal hypersensitivity and inflammatory pain models | Yes | Yes | Yes |
| S. Bang, Y. S. Kim, S. R. Jeong | 2016 | Anti-allodynic effect of theoesberiven F in a vincristine-induced neuropathy model | No | Yes | No |
| D. Baptista-de-Souza, L. Di Cesare Mannelli, M. Zanardelli, et al | 2014 | Serotonergic modulation in neuropathy induced by oxaliplatin: effect on the 5HT2C receptor | Yes | Yes | No |
| I. Barajon, M. Bersani, M. Quartu, et al | 1996 | Neuropeptides and morphological changes in cisplatin-induced dorsal root ganglion neuronopathy | No | No | No |
| G. Bardos, K. Moricz, L. Jaszlits, et al | 2003 | BGP-15, a hydroximic acid derivative, protects against cisplatin- or taxol-induced peripheral neuropathy in rats | No | No | No |
| A. Barzegar-Fallah, H. Alimoradi, S. Mehrzadi, et al | 2014 | The neuroprotective effect of tropisetron on vincristine-induced neurotoxicity | No | Yes | No |
| S. J. Benbow, B. M. Cook, J. Reifert, et al | 2016 | Effects of Paclitaxel and Eribulin in Mouse Sciatic Nerve: A Microtubule-Based Rationale for the Differential Induction of Chemotherapy-Induced Peripheral Neuropathy | No | Yes | No |
| G. J. Bennett, G. K. Liu, W. H. Xiao, H. W. Jin, C. Siau | 2011 | Terminal arbor degeneration--a novel lesion produced by the antineoplastic agent paclitaxel | Yes | No | No |
| N. Bhadri, T. Sanji, H. Madakasira Guggilla, R. Razdan | 2013 | Amelioration of behavioural, biochemical, and neurophysiological deficits by combination of monosodium glutamate with resveratrol/alpha-lipoic acid/coenzyme Q10 in rat model of cisplatin-induced peripheral neuropathy | No | No | No |
| S. Bhalla, N. Singh, A. S. Jaggi | 2015 | Dose-related neuropathic and anti-neuropathic effects of simvastatin in vincristine-induced neuropathic pain in rats | No | No | No |
| M. R. Bhattacharya, J. Gerdts, S. A. Naylor, et al | 2012 | A model of toxic neuropathy in Drosophila reveals a role for MORN4 in promoting axonal degeneration | No | No | No |
| R. Bianchi, M. Brines, G. Lauria, et al | 2006 | Protective effect of erythropoietin and its carbamylated derivative in experimental Cisplatin peripheral neurotoxicity | Yes | Yes | No |
| R. Bianchi, A. Gilardini, V. Rodriguez-Menendez, et al | 2007 | Cisplatin-induced peripheral neuropathy: neuroprotection by erythropoietin without affecting tumour growth | No | Yes | No |
| M. R. Bianco, G. Cirillo, V. Petrosino, et al | 2012 | Neuropathic pain and reactive gliosis are reversed by dialdehydic compound in neuropathic pain rat models | Yes | No | No |
| R. J. Boegman, B. Scarth, L. Dragovic, D. M. Robertson | 1985 | Neurotoxicity of Adriamycin and misonidazole in the mouse | No | No | No |
| W. Boehmerle, P. Huehnchen, S. Peruzzaro, M. Balkaya, M. Endres | 2014 | Electrophysiological, behavioral and histological characterization of paclitaxel, cisplatin, vincristine and bortezomib-induced neuropathy in C57Bl/6 mice | Yes | Yes | Yes |
| W. Boehmerle, H. Muenzfeld, A. Springer, P. Huehnchen, M. Endres | 2014 | Specific targeting of neurotoxic side effects and pharmacological profile of the novel cancer stem cell drug salinomycin in mice | Yes | Yes | Yes |
| N. Boiko, G. Medrano, E. Montano, et al | 2017 | TrpA1 activation in peripheral sensory neurons underlies the ionic basis of pain hypersensitivity in response to vinca alkaloids | Yes | No | No |
| M. R. Bond, L. Wolman | 1965 | EXPERIMENTAL AND CLINICAL OBSERVATIONS CONCERNING THE NEUROTOXICITY OF ETHOGLUCID | No | No | No |
| J. Borzan, S. C. LaGraize, P. N. Fuchs | 2004 | Effect of chronic vincristine treatment on mechanical withdrawal response and pre-pulse inhibition in the rat | No | Yes | No |
| J. Boyette-Davis, P. M. Dougherty | 2011 | Protection against oxaliplatin-induced mechanical hyperalgesia and intraepidermal nerve fiber loss by minocycline | Yes | Yes | No |
| J. A. Boyette-Davis, P. N. Fuchs | 2009 | Differential effects of paclitaxel treatment on cognitive functioning and mechanical sensitivity | No | No | No |
| F. M. Boyle, C. Beatson, R. Monk, S. L. Grant, J. B. Kurek | 2001 | The experimental neuroprotectant leukaemia inhibitory factor (LIF) does not compromise antitumour activity of paclitaxel, cisplatin and carboplatin | Yes | No | No |
| F. M. Boyle, H. R. Wheeler, G. M. Shenfield | 1996 | Glutamate ameliorates experimental vincristine neuropathy | Yes | No | No |
| F. M. Boyle, H. R. Wheeler, G. M. Shenfield | 1999 | Amelioration of experimental cisplatin and paclitaxel neuropathy with glutamate | Yes | No | No |
| W. G. Bradley | 1970 | The neuromyopathy of vincristine in the guinea pig. An electrophysiological and pathological study | No | No | No |
| W. G. Bradley, M. H. Williams | 1973 | Axoplasmic flow in axonal neuropathies. I. Axoplasmic flow in cats with toxic neuropathies | No | No | No |
| L. Brandolini, E. Benedetti, P. A. Ruffini, et al | 2017 | CXCR1/2 pathways in paclitaxel-induced neuropathic pain | No | No | No |
| J. M. Braz, X. Wang, Z. Guan, J. L. Rubenstein, A. I. Basbaum | 2015 | Transplant-mediated enhancement of spinal cord GABAergic inhibition reverses paclitaxel-induced mechanical and heat hypersensitivity | Yes | No | No |
| J. D. Brederson, S. K. Joshi, K. E. Browman, et al | 2012 | PARP inhibitors attenuate chemotherapy-induced painful neuropathy | Yes | Yes | No |
| C. L. Bregman, R. A. Buroker, R. S. Hirth, A. R. Crosswell, S. K. Durham | 1994 | Etoposide- and BMY-40481-induced sensory neuropathy in mice | No | Yes | No |
| M. Bremer, F. Frob, T. Kichko, et al | 2011 | Sox10 is required for Schwann-cell homeostasis and myelin maintenance in the adult peripheral nerve | No | No | No |
| A. M. S. Brito, A. M. Godin, P. S. A. Augusto, et al | 2017 | Antiallodynic activity of leflunomide is partially inhibited by naltrexone and glibenclamide and associated with reduced production of TNF-alpha and CXCL-1 | No | No | No |
| J. Bruna, A. Ale, R. Velasco, J. Jaramillo, X. Navarro, E. Udina | 2011 | Evaluation of pre-existing neuropathy and bortezomib retreatment as risk factors to develop severe neuropathy in a mouse model | No | No | No |
| I. Brusco, C. Camponogara, F. B. Carvalho, et al | 2017 | alpha-Spinasterol: a COX inhibitor and a transient receptor potential vanilloid 1 antagonist presents an antinociceptive effect in clinically relevant models of pain in mice | Yes | Yes | No |
| I. Brusco, C. R. Silva, G. Trevisan, et al | 2017 | Potentiation of Paclitaxel-Induced Pain Syndrome in Mice by Angiotensin I Converting Enzyme Inhibition and Involvement of Kinins | Yes | No | No |
| M. Bujalska, M. Arazna, H. Makulska-Nowak, S. W. Gumulka | 2008 | Alpha1- and alpha2-Adrenoreceptor antagonists in streptozotocin- and vincristine-induced hyperalgesia | No | No | No |
| M. Bujalska, S. W. Gumulka | 2008 | Effect of cyclooxygenase and nitric oxide synthase inhibitors on vincristine induced hyperalgesia in rats | No | No | No |
| M. Bujalska, H. Makulska-Nowak | 2009 | Bradykinin receptor antagonists and cyclooxygenase inhibitors in vincristine- and streptozotocin-induced hyperalgesia | No | No | No |
| M. Bujalska, H. Makulska-Nowak, S. W. Gumulka | 2009 | Magnesium ions and opioid agonists in vincristine-induced neuropathy | No | No | No |
| K. L. Bullinger, P. Nardelli, Q. Wang, M. M. Rich, T. C. Cope | 2011 | Oxaliplatin neurotoxicity of sensory transduction in rat proprioceptors | No | No | No |
| E. Burgos, D. Gomez-Nicola, D. Pascual, M. I. Martin, M. Nieto-Sampedro, C. Goicoechea | 2012 | Cannabinoid agonist WIN 55,212-2 prevents the development of paclitaxel-induced peripheral neuropathy in rats. Possible involvement of spinal glial cells | Yes | No | No |
| N. Callizot, E. Andriambeloson, J. Glass, et al | 2008 | Interleukin-6 protects against paclitaxel, cisplatin and vincristine-induced neuropathies without impairing chemotherapeutic activity | No | Yes | No |
| A. Canta, A. Chiorazzi, V. Carozzi, et al | 2011 | In vivo comparative study of the cytotoxicity of a liposomal formulation of cisplatin (lipoplatin) | No | Yes | No |
| V. Carozzi, A. Chiorazzi, A. Canta, et al | 2009 | Effect of the chronic combined administration of cisplatin and paclitaxel in a rat model of peripheral neurotoxicity | No | No | No |
| V. A. Carozzi, A. Canta, N. Oggioni, et al | 2010 | Neurophysiological and neuropathological characterization of new murine models of chemotherapy-induced chronic peripheral neuropathies | No | Yes | No |
| V. A. Carozzi, A. Chiorazzi, A. Canta, et al | 2010 | Glutamate carboxypeptidase inhibition reduces the severity of chemotherapy-induced peripheral neurotoxicity in rat | No | Yes | No |
| V. A. Carozzi, A. Chiorazzi, A. Canta, et al | 2015 | Chemotherapy-induced peripheral neurotoxicity in immune-deficient mice: new useful ready-to-use animal models | No | Yes | No |
| V. A. Carozzi, C. L. Renn, M. Bardini, et al | 2013 | Bortezomib-induced painful peripheral neuropathy: an electrophysiological, behavioral, morphological and mechanistic study in the mouse | Yes | Yes | No |
| F. Carta, L. Di Cesare Mannelli, M. Pinard, et al | 2015 | A class of sulfonamide carbonic anhydrase inhibitors with neuropathic pain modulating effects | No | No | No |
| J. P. Cata, H. R. Weng, P. M. Dougherty | 2004 | Cyclooxygenase inhibitors and thalidomide ameliorate vincristine-induced hyperalgesia in rats | Yes | No | No |
| J. P. Cata, H. R. Weng, P. M. Dougherty | 2008 | Behavioral and electrophysiological studies in rats with cisplatin-induced chemoneuropathy | Yes | No | No |
| J. P. Cata, H. R. Weng, P. M. Dougherty | 2008 | The effects of thalidomide and minocycline on taxol-induced hyperalgesia in rats | Yes | No | No |
| G. Cavaletti, D. Fabbrica, C. Minoia, L. Frattola, G. Tredici | 1998 | Carboplatin toxic effects on the peripheral nervous system of the rat | No | Yes | No |
| G. Cavaletti, A. Gilardini, A. Canta, et al | 2007 | Bortezomib-induced peripheral neurotoxicity: a neurophysiological and pathological study in the rat | No | No | No |
| G. Cavaletti, G. Pezzoni, C. Pisano, et al | 2002 | Cisplatin-induced peripheral neurotoxicity in rats reduces the circulating levels of nerve growth factor | No | Yes | No |
| G. Cavaletti, G. Tredici, M. Braga, S. Tazzari | 1995 | Experimental peripheral neuropathy induced in adult rats by repeated intraperitoneal administration of taxol | No | No | No |
| G. Cavaletti, G. Tredici, P. Marmiroli, M. G. Petruccioli, I. Barajon, D. Fabbrica | 1992 | Morphometric study of the sensory neuron and peripheral nerve changes induced by chronic cisplatin (DDP) administration in rats | No | No | No |
| G. Cavaletti, G. Tredici, M. G. Petruccioli, et al | 2001 | Effects of different schedules of oxaliplatin treatment on the peripheral nervous system of the rat | No | Yes | No |
| R. Cece, M. G. Petruccioli, G. Cavaletti, I. Barajon, G. Tredici | 1995 | An ultrastructural study of neuronal changes in dorsal root ganglia (DRG) of rats after chronic cisplatin administrations | No | No | No |
| N. Celebi, H. Cil, O. Cil, O. Canbay, R. Onur, U. Aypar | 2013 | Protective effect of coenzyme Q10 in paclitaxel-induced peripheral neuropathy in rats | Yes | Yes | No |
| O. Cerles, E. Benoit, C. Chereau, et al | 2017 | Niclosamide inhibits oxaliplatin neurotoxicity while improving colorectal cancer therapeutic response | No | No | No |
| I. Cervellini, E. Bello, R. Frapolli, et al | 2010 | The neuroprotective effect of erythropoietin in docetaxel-induced peripheral neuropathy causes no reduction of antitumor activity in 13762 adenocarcinoma-bearing rats | No | Yes | No |
| A. Cetinkaya-Fisgin, M. G. Joo, X. Ping, et al | 2016 | Identification of fluocinolone acetonide to prevent paclitaxel-induced peripheral neuropathy | No | No | No |
| T. Chaumette, E. Chapuy, E. Berrocoso, et al | 2018 | Effects of S 38093, an antagonist/inverse agonist of histamine H3 receptors, in models of neuropathic pain in rats | Yes | Yes | No |
| A. Chelini, S. Brogi, M. Paolino, et al | 2017 | Synthesis and Biological Evaluation of Novel Neuroprotective Pyridazine Derivatives as Excitatory Amino Acid Transporter 2 (EAAT2) Activators | No | No | No |
| H. Chen, Q. Wang, D. Shi, et al | 2016 | Celecoxib alleviates oxaliplatin-induced hyperalgesia through inhibition of spinal ERK1/2 signaling | Yes | Yes | No |
| K. Chen, Z. F. Zhang, M. F. Liao, W. L. Yao, J. Wang, X. R. Wang | 2015 | Blocking PAR2 attenuates oxaliplatin-induced neuropathic pain via TRPV1 and releases of substance P and CGRP in superficial dorsal horn of spinal cord | No | No | No |
| L. H. Chen, Y. T. Sun, Y. F. Chen, et al | 2015 | Integrating Image-Based High-Content Screening with Mouse Models Identifies 5-Hydroxydecanoate as a Neuroprotective Drug for Paclitaxel-Induced Neuropathy | No | Yes | No |
| S. R. Chen, L. Zhu, H. Chen, L. Wen, G. Laumet, H. L. Pan | 2014 | Increased spinal cord Na(+)-K(+)-2Cl(-) cotransporter-1 (NKCC1) activity contributes to impairment of synaptic inhibition in paclitaxel-induced neuropathic pain | No | No | No |
| X. Chen, P. G. Green, J. D. Levine | 2011 | Abnormal muscle afferent function in a model of taxol chemotherapy-induced painful neuropathy | No | No | No |
| Y. Chen, C. Yang, Z. J. Wang | 2011 | Proteinase-activated receptor 2 sensitizes transient receptor potential vanilloid 1, transient receptor potential vanilloid 4, and transient receptor potential ankyrin 1 in paclitaxel-induced neuropathic pain | No | No | No |
| Z. Chen, K. Janes, C. Chen, et al | 2012 | Controlling murine and rat chronic pain through A3 adenosine receptor activation | Yes | No | No |
| X. Cheng, J. Huo, D. Wang, et al | 2017 | Herbal Medicine AC591 Prevents Oxaliplatin-Induced Peripheral Neuropathy in Animal Model and Cancer Patients | Yes | Yes | No |
| V. Chentanez, S. Sanguanrungsirigul, N. Panyasawad | 2003 | Effects of ganglioside on paclitaxel (Taxol) induced neuropathy in rats | No | No | No |
| V. Chentanez, N. Thanomsridejchai, N. Duangmardphon, et al | 2009 | Ganglioside GM1 (porcine) ameliorates paclitaxel-induced neuropathy in rats | No | Yes | No |
| T. Chiba, Y. Oka, T. Kambe, et al | 2016 | Paclitaxel-induced peripheral neuropathy increases substance P release in rat spinal cord | Yes | No | No |
| T. Chiba, Y. Oka, H. Sashida, et al | 2017 | Vincristine-induced peripheral neuropathic pain and expression of transient receptor potential vanilloid 1 in rat | Yes | Yes | No |
| E. S. Cho, H. E. Lowndes, B. D. Goldstein | 1983 | Neurotoxicology of vincristine in the cat. Morphological study | No | No | No |
| E. S. Cho, P. S. Spencer, B. S. Jortner, H. H. Schaumburg | 1980 | A single intravenous injection of doxorubicin (Adriamycin) induces sensory neuronopathy in rats | No | No | No |
| E. S. Cho, J. M. Yi, J. S. Park, et al | 2016 | Aqueous extract of Lithospermi radix attenuates oxaliplatin-induced neurotoxicity in both in vitro and in vivo models | Yes | No | No |
| B. Chogtu, K. L. Bairy, D. Smitha, S. Dhar, P. Himabindu | 2011 | Comparison of the efficacy of carbamazepine, gabapentin and lamotrigine for neuropathic pain in rats | Yes | No | No |
| J. Choi, C. Jeon, J. H. Lee, et al | 2017 | Suppressive Effects of Bee Venom Acupuncture on Paclitaxel-Induced Neuropathic Pain in Rats: Mediation by Spinal alpha(2)-Adrenergic Receptor | Yes | Yes | No |
| J. W. Choi, S. Y. Kang, J. G. Choi, et al | 2015 | Analgesic effect of electroacupuncture on paclitaxel-induced neuropathic pain via spinal opioidergic and adrenergic mechanisms in mice | Yes | Yes | No |
| S. Choi, A. Yamada, W. Kim, S. K. Kim, H. Furue | 2017 | Noradrenergic inhibition of spinal hyperexcitation elicited by cutaneous cold stimuli in rats with oxaliplatin-induced allodynia: electrophysiological and behavioral assessments | Yes | No | No |
| S. S. Choi, W. U. Koh, J. S. Nam, J. W. Shin, J. G. Leem, J. H. Suh | 2013 | Effect of ethyl pyruvate on Paclitaxel-induced neuropathic pain in rats | Yes | Yes | No |
| S. B. Christensen, A. J. Hone, I. Roux, et al | 2017 | RgIA4 Potently Blocks Mouse α9α10 nAChRs and Provides Long Lasting Protection against Oxaliplatin-Induced Cold Allodynia. | Yes | No | No |
| Y. Chtourou, B. Gargouri, M. Kebieche, H. Fetoui | 2015 | Naringin Abrogates Cisplatin-Induced Cognitive Deficits and Cholinergic Dysfunction Through the Down-Regulation of AChE Expression and iNOS Signaling Pathways in Hippocampus of Aged Rats | No | Yes | No |
| I. C. Ciotu, D. Lupuliasa, C. Chirita, C. E. Zbarcea, S. Negres | 2016 | The antihyperalgic effect of memantine in a rat model of paclitaxel induced neuropathic pain | No | No | No |
| I. C. Ciotu, D. Lupuliasa, C. E. Zbarcea, S. Negres | 2016 | The effect of nimodipine on a rat model of paclitaxel - Induced peripheral neuropathy | No | No | No |
| K. D. Cliffer, J. A. Siuciak, S. R. Carson, et al | 1998 | Physiological characterization of taxol-induced large-fiber sensory neuropathy in the rat | No | No | No |
| P. C. Contreras, J. L. Vaught, J. A. Gruner, et al | 1997 | Insulin-like growth factor-I prevents development of a vincristine neuropathy in mice | Yes | No | No |
| R. Coriat, J. Alexandre, C. Nicco, et al | 2014 | Treatment of oxaliplatin-induced peripheral neuropathy by intravenous mangafodipir | No | No | No |
| G. Corsetti, L. Rodella, R. Rezzani, A. Stacchiotti, R. Bianchi | 2000 | Cytoplasmic changes in satellite cells of spinal ganglia induced by cisplatin treatment in rats | No | No | No |
| R. Costa, M. A. Bicca, M. N. Manjavachi, et al | 2017 | Kinin Receptors Sensitize TRPV4 Channel and Induce Mechanical Hyperalgesia: Relevance to Paclitaxel-Induced Peripheral Neuropathy in Mice | Yes | Yes | Yes |
| R. Costa, E. M. Motta, R. C. dutra, et al | 2011 | Anti-nociceptive effect of kinin B1 and B2 receptor antagonists on peripheral neuropathy induced by paclitaxel in mice | No | Yes | No |
| M. Dambska, M. Muzylak, D. Maslinska | 1995 | The features of peripheral nerve lesions in young and adult rabbits after vincristine administration | No | No | No |
| P. De Koning, J. P. Neijt, F. G. I. Jennekens, W. H. Gispen | 1987 | Org. 2766 protects from cisplatin-induced neurotoxicity in rats | No | No | No |
| P. De Koning, J. P. Neijt, F. G. I. Jennekens, W. H. Gispen | 1987 | Evaluation of cis-diamminedichloroplatinum (II) (Cisplatin) neurotoxicity in rats | No | No | No |
| B. Deng, L. Jia, L. Pan, et al | 2016 | Wen-Luo-Tong Prevents Glial Activation and Nociceptive Sensitization in a Rat Model of Oxaliplatin-Induced Neuropathic Pain | No | No | No |
| L. Deng, B. L. Cornett, K. Mackie, A. G. Hohmann | 2015 | CB1 Knockout Mice Unveil Sustained CB2-Mediated Antiallodynic Effects of the Mixed CB1/CB2 Agonist CP55,940 in a Mouse Model of Paclitaxel-Induced Neuropathic Pain | Yes | Yes | No |
| L. Deng, J. Guindon, B. L. Cornett, A. Makriyannis, K. Mackie, A. G. Hohmann | 2015 | Chronic cannabinoid receptor 2 activation reverses paclitaxel neuropathy without tolerance or cannabinoid receptor 1-dependent withdrawal | Yes | Yes | No |
| L. Deng, W. H. Lee, Z. Xu, A. Makriyannis, A. G. Hohmann | 2016 | Prophylactic treatment with the tricyclic antidepressant desipramine prevents development of paclitaxel-induced neuropathic pain through activation of endogenous analgesic systems | Yes | Yes | No |
| J. Descoeur, V. Pereira, A. Pizzoccaro, et al | 2011 | Oxaliplatin-induced cold hypersensitivity is due to remodelling of ion channel expression in nociceptors | No | Yes | No |
| J. R. Deuis, Y. L. Lim, S. Rodrigues de Sousa, et al | 2014 | Analgesic effects of clinically used compounds in novel mouse models of polyneuropathy induced by oxaliplatin and cisplatin | Yes | No | No |
| J. R. Deuis, K. Zimmermann, A. A. Romanovsky, et al | 2013 | An animal model of oxaliplatin-induced cold allodynia reveals a crucial role for Nav1.6 in peripheral pain pathways | Yes | No | No |
| L. Di Cesare Mannelli, E. Lucarini, L. Micheli, et al | 2017 | Effects of natural and synthetic isothiocyanate-based H2S-releasers against chemotherapy-induced neuropathic pain: Role of Kv7 potassium channels | Yes | No | No |
| L. Di Cesare Mannelli, M. Marcoli, L. Micheli, et al | 2015 | Oxaliplatin evokes P2X7-dependent glutamate release in the cerebral cortex: A pain mechanism mediated by Pannexin 1 | No | No | No |
| L. Di Cesare Mannelli, M. Maresca, C. Farina, M. W. Scherz, C. Ghelardini | 2015 | A model of neuropathic pain induced by sorafenib in the rat: Effect of dimiracetam | No | No | No |
| L. Di Cesare Mannelli, M. Maresca, L. Micheli, C. Farina, M. W. Scherz, C. Ghelardini | 2017 | A rat model of FOLFOX-induced neuropathy: effects of oral dimiracetam in comparison with duloxetine and pregabalin | No | No | No |
| L. Di Cesare Mannelli, A. Pacini, L. Bonaccini, M. Zanardelli, T. Mello, C. Ghelardini | 2013 | Morphologic features and glial activation in rat oxaliplatin-dependent neuropathic pain | Yes | No | No |
| L. Di Cesare Mannelli, A. Pacini, F. Corti, et al | 2015 | Antineuropathic profile of N-palmitoylethanolamine in a rat model of oxaliplatin-induced neurotoxicity | Yes | No | No |
| L. Di Cesare Mannelli, A. Pacini, C. Matera, et al | 2014 | Involvement of alpha7 nAChR subtype in rat oxaliplatin-induced neuropathy: effects of selective activation | Yes | No | No |
| L. Di Cesare Mannelli, A. Pacini, L. Micheli, et al | 2017 | Astragali radix: could it be an adjuvant for oxaliplatin-induced neuropathy? | Yes | Yes | No |
| L. Di Cesare Mannelli, A. Pacini, L. Micheli, A. Tani, M. Zanardelli, C. Ghelardini | 2014 | Glial role in oxaliplatin-induced neuropathic pain | Yes | Yes | No |
| L. Di Cesare Mannelli, B. Tenci, L. Micheli, et al | 2017 | Adipose-derived stem cells decrease pain in a rat model of oxaliplatin-induced neuropathy: Role of VEGF-A modulation | No | Yes | No |
| L. Di Cesare Mannelli, M. Zanardelli, P. Failli, C. Ghelardini | 2012 | Oxaliplatin-induced neuropathy: Oxidative Stress as Pathological Mechanism. Protective Effect of Silibinin | Yes | No | No |
| L. Di Cesare Mannelli, M. Zanardelli, C. Ghelardini | 2013 | Nicotine is a pain reliever in trauma- and chemotherapy-induced neuropathy models | Yes | Yes | No |
| L. Di Cesare Mannelli, M. Zanardelli, I. Landini, et al | 2016 | Effect of the SOD mimetic MnL4 on in vitro and in vivo oxaliplatin toxicity: Possible aid in chemotherapy induced neuropathy | Yes | No | No |
| A. Dilley, N. Richards, K. G. Pulman, G. M. Bove | 2013 | Disruption of fast axonal transport in the rat induces behavioral changes consistent with neuropathic pain | Yes | No | No |
| O. A. Dina, X. Chen, D. Reichling, J. D. Levine | 2001 | Role of protein kinase Cepsilon and protein kinase A in a model of paclitaxel-induced painful peripheral neuropathy in the rat | No | No | No |
| O. A. Dina, C. A. Parada, J. Yeh, X. Chen, G. C. McCarter, J. D. Levine | 2004 | Integrin signaling in inflammatory and neuropathic pain in the rat | No | No | No |
| R. Djaldetti, J. Hart, S. Alexandrova, et al | 1996 | Vincristine-induced alterations in Schwann cells of mouse peripheral nerve | No | No | No |
| G. Donvito, J. L. Wilkerson, M. I. Damaj, A. H. Lichtman | 2016 | Palmitoylethanolamide reverses paclitaxel-induced allodynia in mice | No | Yes | No |
| T. Doyle, Z. Chen, C. Muscoli, et al | 2012 | Targeting the overproduction of peroxynitrite for the prevention and reversal of paclitaxel-induced neuropathic pain | Yes | No | No |
| P. Draxler, S. D. Honsek, L. Forsthuber, V. Hadschieff, J. Sandkuhler | 2014 | VGluT3(+) primary afferents play distinct roles in mechanical and cold hypersensitivity depending on pain etiology | Yes | No | No |
| N. A. Duggett, S. J. L. Flatters | 2017 | Characterization of a rat model of bortezomib-induced painful neuropathy | Yes | Yes | No |
| N. A. Duggett, L. A. Griffiths, S. J. L. Flatters | 2017 | Paclitaxel-induced painful neuropathy is associated with changes in mitochondrial bioenergetics, glycolysis, and an energy deficit in dorsal root ganglia neurons | No | No | No |
| N. A. Duggett, L. A. Griffiths, O. E. McKenna, et al | 2016 | Oxidative stress in the development, maintenance and resolution of paclitaxel-induced painful neuropathy | Yes | No | No |
| A. Dzagnidze, Z. Katsarava, J. Makhalova, et al | 2007 | Repair capacity for platinum-DNA adducts determines the severity of cisplatin-induced peripheral neuropathy | No | Yes | No |
| N. Egashira, S. Hirakawa, T. Kawashiri, T. Yano, H. Ikesue, R. Oishi | 2010 | Mexiletine reverses oxaliplatin-induced neuropathic pain in rats | Yes | No | No |
| T. A. El-Masry, M. E. El Sayaad, I. A. Gaaboub, W. M. Fouda | 2013 | Effects of capsaicin on rat sciatic nerve in vincristine-induced neuropathic pain model | No | No | No |
| H. A. Erken, E. R. Koc, H. Yazici, A. Yay, G. O. Onder, S. F. Sarici | 2014 | Selenium partially prevents cisplatin-induced neurotoxicity: a preliminary study | No | Yes | No |
| J. E. Fardell, J. Vardy, L. A. Monds, I. N. Johnston | 2015 | The long-term impact of oxaliplatin chemotherapy on rodent cognition and peripheral neuropathy | No | No | No |
| R. G. Fariello, C. Ghelardini, L. Di Cesare Mannelli, et al | 2014 | Broad spectrum and prolonged efficacy of dimiracetam in models of neuropathic pain | Yes | No | No |
| A. A. Farshid, E. Tamaddonfard, S. Najafi | 2015 | Effects of histidine and n-acetylcysteine on experimental lesions induced by doxorubicin in sciatic nerve of rats | No | No | No |
| G. Favaro, F. Di Gregorio, C. Panozzo, M. G. Fiori | 1988 | Ganglioside treatment of vincristine-induced neuropathy. An electrophysiologic study | No | No | No |
| C. Favre-Guilmard, M. Auguet, P. E. Chabrier | 2009 | Different antinociceptive effects of botulinum toxin type A in inflammatory and peripheral polyneuropathic rat models | No | No | No |
| L. F. Ferrari, A. Chum, O. Bogen, D. B. Reichling, J. D. Levine | 2011 | Role of Drp1, a key mitochondrial fission protein, in neuropathic pain | No | No | No |
| J. Ferrier, M. Bayet-Robert, R. Dalmann, et al | 2015 | Cholinergic Neurotransmission in the Posterior Insular Cortex Is Altered in Preclinical Models of Neuropathic Pain: Key Role of Muscarinic M2 Receptors in Donepezil-Induced Antinociception | Yes | Yes | No |
| J. Ferrier, M. Bayet-Robert, B. Pereira, et al | 2013 | A polyamine-deficient diet prevents oxaliplatin-induced acute cold and mechanical hypersensitivity in rats | Yes | Yes | No |
| M. Fidanboylu, L. A. Griffiths, S. J. L. Flatters | 2011 | Global inhibition of reactive oxygen species (ROS) inhibits paclitaxel-induced painful peripheral neuropathy | Yes | Yes | No |
| M. G. Fiori, A. Schiavinato, E. Lini, M. G. Nunzi | 1995 | Peripheral neuropathy induced by intravenous administration of vincristine sulfate in the rabbit. An ultrastructural study | No | No | No |
| S. J. Fischer, E. S. McDonald, L. Gross, A. J. Windebank | 2001 | Alterations in cell cycle regulation underlie cisplatin induced apoptosis of dorsal root ganglion neurons in vivo | Yes | No | No |
| M. Fitzgerald, C. J. Woolf, S. J. Gibson, P. S. Mallaburn | 1984 | Alterations in the structure, function, and chemistry of C fibers following local application of vinblastine to the sciatic nerve of the rat | No | No | No |
| S. J. Flatters, G. J. Bennett | 2004 | Ethosuximide reverses paclitaxel- and vincristine-induced painful peripheral neuropathy | Yes | No | No |
| S. J. Flatters, G. J. Bennett | 2006 | Studies of peripheral sensory nerves in paclitaxel-induced painful peripheral neuropathy: evidence for mitochondrial dysfunction | Yes | No | No |
| S. J. L. Flatters, W. H. Xiao, G. J. Bennett | 2006 | Acetyl-L-carnitine prevents and reduces paclitaxel-induced painful peripheral neuropathy | Yes | No | No |
| A. Friesland, Z. Weng, M. Duenas, S. M. Massa, F. M. Longo, Q. Lu | 2014 | Amelioration of cisplatin-induced experimental peripheral neuropathy by a small molecule targeting p75NTR | No | No | No |
| S. Fujita, S. Ushio, N. Ozawa, et al | 2015 | Exenatide facilitates recovery from oxaliplatin-induced peripheral neuropathy in rats | No | No | No |
| T. Fukuizumi, T. Ohkubo, K. Kitamura | 2003 | Spinal sensitization mechanism in vincristine-induced hyperalgesia in mice | No | No | No |
| N. Galeotti, E. Vivoli, A. R. Bilia, F. F. Vincieri, C. Ghelardini | 2010 | St. John’s Wort reduces neuropathic pain through a hypericin-mediated inhibition of the protein kinase C g and e activity | No | No | No |
| H. F. Galley, B. McCormick, K. L. Wilson, D. A. Lowes, L. Colvin, C. Torsney | 2017 | Melatonin limits paclitaxel-induced mitochondrial dysfunction in vitro and protects against paclitaxel-induced neuropathic pain in the rat | Yes | No | No |
| L. Gao, Y. Zheng, C. Zhao, H. Teng | 2017 | Investigation on effect of basalin coated silver nanoparticles as antioxidant for alleviating peripheral neuropathy in mice treated with oxaliplatin | No | No | No |
| M. Gao, X. Yan, H. R. Weng | 2013 | Inhibition of glycogen synthase kinase 3beta activity with lithium prevents and attenuates paclitaxel-induced neuropathic pain | Yes | Yes | No |
| W. Gao, Y. Zan, Z. J. Wang, X. Y. Hu, F. Huang | 2016 | Quercetin ameliorates paclitaxel-induced neuropathic pain by stabilizing mast cells, and subsequently blocking PKCepsilon-dependent activation of TRPV1 | No | Yes | No |
| W. Q. Gao, N. Dybdal, N. Shinsky, et al | 1995 | Neurotrophin-3 reverses experimental cisplatin-induced peripheral sensory neuropathy | Yes | No | No |
| J. M. Garcia, J. P. Cata, P. M. Dougherty, R. G. Smith | 2008 | Ghrelin prevents cisplatin-induced mechanical hyperalgesia and cachexia | Yes | Yes | No |
| P. Gauchan, T. Andoh, K. Ikeda, et al | 2009 | Mechanical allodynia induced by paclitaxel, oxaliplatin and vincristine: different effectiveness of gabapentin and different expression of voltage-dependent calcium channel alpha(2)delta-1 subunit | No | No | No |
| P. Gauchan, T. Andoh, A. Kato, Y. Kuraishi | 2009 | Involvement of increased expression of transient receptor potential melastatin 8 in oxaliplatin-induced cold allodynia in mice | No | No | No |
| P. Gauchan, T. Andoh, A. Kato, A. Sasaki, Y. Kuraishi | 2009 | Effects of the prostaglandin E1 analog limaprost on mechanical allodynia caused by chemotherapeutic agents in mice | No | No | No |
| C. Geis, B. K. Beyreuther, T. Stohr, C. Sommer | 2011 | Lacosamide has protective disease modifying properties in experimental vincristine neuropathy | Yes | Yes | Yes |
| S. Geisler, R. A. Doan, A. Strickland, X. Huang, J. Milbrandt, A. DiAntonio | 2016 | Prevention of vincristine-induced peripheral neuropathy by genetic deletion of SARM1 in mice | Yes | No | No |
| R. Gerritsen Van Der Hoop, P. De Koning, E. Boven, J. P. Neijt, F. G. I. Jennekens, W. H. Gispen | 1988 | Efficacy of the neuropeptide ORG.2766 in the prevention and treatment of cisplatin-induced neurotoxicity in rats | Yes | Yes | No |
| R. Gerritsen van der Hoop, F. P. Hamers, J. P. Neijt, H. Veldman, W. H. Gispen, F. G. Jennekens | 1994 | Protection against cisplatin induced neurotoxicity by ORG 2766: histological and electrophysiological evidence | No | No | No |
| C. Ghelardini, J. F. Desaphy, M. Muraglia, et al | 2010 | Effects of a new potent analog of tocainide on hNav1.7 sodium channels and in vivo neuropathic pain models | No | No | No |
| C. Ghelardini, C. Menicacci, D. Cerretani, E. Bianchi | 2014 | Spinal administration of mGluR5 antagonist prevents the onset of bortezomib induced neuropathic pain in rat | Yes | Yes | Yes |
| O. Ghirardi, P. Lo Giudice, C. Pisano, et al | 2005 | Acetyl-L-Carnitine prevents and reverts experimental chronic neurotoxicity induced by oxaliplatin, without altering its antitumor properties | No | No | No |
| A. Gilardini, R. L. Avila, N. Oggioni, et al | 2012 | Myelin structure is unaltered in chemotherapy-induced peripheral neuropathy | No | No | No |
| W. H. Gispen, F. P. Hamers, C. J. Vecht, F. G. Jennekens, J. P. Neyt | 1992 | ACTH/MSH like peptides in the treatment of cisplatin neuropathy | Yes | Yes | No |
| L. Golchin, M. Shabani, S. Harandi, M. Razavinasab | 2015 | Pistachio supplementation attenuates motor and cognition impairments induced by cisplatin or vincristine in rats | No | Yes | No |
| B. D. Goldstein, H. E. Lowndes, E. S. Cho | 1981 | Neurotoxicology of vincristine in the cat. Electrophysiological studies | No | No | No |
| S. S. Gong, Y. X. Li, M. T. Zhang, et al | 2016 | Neuroprotective Effect of Matrine in Mouse Model of Vincristine-Induced Neuropathic Pain | No | No | No |
| C. Goudet, E. Chapuy, A. Alloui, F. Acher, J. P. Pin, A. Eschalier | 2008 | Group III metabotropic glutamate receptors inhibit hyperalgesia in animal models of inflammation and neuropathic pain | Yes | Yes | No |
| S. Goyal, G. Menaria, D. Kumar, et al | 2016 | Evaluation of the protective effects of Sapindus trifoliatus aqueous extract on vincristine induced neuropathic pain in rats | No | No | No |
| L. S. Green, J. A. Donoso, I. E. Heller-Bettinger, F. E. Samson | 1977 | Axonal transport disturbances in the vincristine-induced peripheral neuropathy | No | No | No |
| N. Greeshma, K. G. Prasanth, B. Balaji | 2015 | Tetrahydrocurcumin exerts protective effect on vincristine induced neuropathy: Behavioral, biochemical, neurophysiological and histological evidence | No | No | No |
| L. A. Griffiths, S. J. L. Flatters | 2015 | Pharmacological Modulation of the Mitochondrial Electron Transport Chain in Paclitaxel-Induced Painful Peripheral Neuropathy | Yes | Yes | No |
| G. Gris, E. Portillo-Salido, B. Aubel, et al | 2016 | The selective sigma-1 receptor antagonist E-52862 attenuates neuropathic pain of different aetiology in rats | No | No | No |
| Q. Gui, C. Xu, D. Li, L. Zhuang, S. Xia, S. Yu | 2015 | Urinary N telopeptide levels in predicting the anti-nociceptive responses of zoledronic acid and paclitaxel in a rat model of bone metastases | No | Yes | No |
| J. Guindon, L. Deng, B. Fan, J. Wager-Miller, A. G. Hohmann | 2014 | Optimization of a cisplatin model of chemotherapy-induced peripheral neuropathy in mice: use of vitamin C and sodium bicarbonate pretreatments to reduce nephrotoxicity and improve animal health status | Yes | Yes | No |
| J. Guindon, A. G. Hohmann | 2013 | Use of sodium bicarbonate to promote weight gain, maintain body temperature, normalize renal functions and minimize mortality in rodents receiving the chemotherapeutic agent cisplatin | Yes | No | No |
| J. Guindon, Y. Lai, S. M. Takacs, H. B. Bradshaw, A. G. Hohmann | 2013 | Alterations in endocannabinoid tone following chemotherapy-induced peripheral neuropathy: effects of endocannabinoid deactivation inhibitors targeting fatty-acid amide hydrolase and monoacylglycerol lipase in comparison to reference analgesics following cisplatin treatment | Yes | Yes | No |
| Z. Guo, Y. Man, X. Wang, et al | 2014 | Levo-tetrahydropalmatine attenuates oxaliplatin-induced mechanical hyperalgesia in mice | No | Yes | No |
| G. Hache, B. P. Guiard, T. H. Nguyen, et al | 2015 | Antinociceptive activity of the new triple reuptake inhibitor NS18283 in a mouse model of chemotherapy-induced neuropathic pain | Yes | No | No |
| F. P. Hamers, R. G. van der Hoop, P. A. Steerenburg, J. P. Neijt, W. H. Gispen | 1991 | Putative neurotrophic factors in the protection of cisplatin-induced peripheral neuropathy in rats | Yes | No | No |
| F. P. T. Hamers, J. H. Brakkee, E. Cavalletti, et al | 1993 | Reduced glutathione protects against cisplatin-induced neurotoxicity in rats | No | No | No |
| F. P. T. Hamers, C. Pette, B. Bravenboer, C. J. Vecht, J. P. Neijt, W. H. Gispen | 1993 | Cisplatin-induced neuropathy in mature rats: Effects of the melanocortin-derived peptide ORG 2766 | No | Yes | No |
| M. V. Hamity, S. R. White, R. Y. Walder, M. S. Schmidt, C. Brenner, D. L. Hammond | 2017 | Nicotinamide riboside, a form of vitamin B3 and NAD + precursor, relieves the nociceptive and aversive dimensions of paclitaxel-induced peripheral neuropathy in female rats | No | Yes | No |
| F. Y. Han, B. D. Wyse, M. T. Smith | 2014 | Optimization and pharmacological characterization of a refined cisplatin-induced rat model of peripheral neuropathic pain | Yes | Yes | No |
| S. M. Han, Y. H. Kim, H. U. Jo, J. A. Kwak, H. J. Park | 2017 | Tianeptine Reduces Mechanical Allodynia in Spinal Nerve-ligated and Chemotherapy-induced Neuropathic Mice | No | Yes | No |
| N. Hansen, N. Uceyler, F. Palm, et al | 2011 | Serotonin transporter deficiency protects mice from mechanical allodynia and heat hyperalgesia in vincristine neuropathy | No | No | No |
| T. Hara, T. Chiba, K. Abe, et al | 2013 | Effect of paclitaxel on transient receptor potential vanilloid 1 in rat dorsal root ganglion | Yes | No | No |
| H. M. Harris, K. J. Sufka, W. Gul, M. A. ElSohly | 2016 | Effects of Delta-9-Tetrahydrocannabinol and Cannabidiol on Cisplatin-Induced Neuropathy in Mice | No | Yes | No |
| Z. W. He, W. Wei, S. P. Li, Q. Ling, K. J. Liao, X. Wang | 2014 | Anti-allodynic effects of obtusifolin and gluco-obtusifolin against inflammatory and neuropathic pain possible mechanism for neuroinflammation | Yes | No | No |
| T. Hidaka, T. Shima, K. Nagira, et al | 2009 | Herbal medicine Shakuyaku-kanzo-to reduces paclitaxel-induced painful peripheral neuropathy in mice | No | No | No |
| H. Higuchi, S. Yamamoto, S. Ushio, T. Kawashiri, N. Egashira | 2015 | Goshajinkigan reduces bortezomib-induced mechanical allodynia in rats: Possible involvement of kappa opioid receptor | No | No | No |
| S. W. Hohmann, C. Angioni, S. Tunaru, et al | 2017 | The G2A receptor (GPR132) contributes to oxaliplatin-induced mechanical pain hypersensitivity | No | Yes | No |
| J. Holmes, J. Stanko, M. Varchenko, et al | 1998 | Comparative neurotoxicity of oxaliplatin, cisplatin, and ormaplatin in a Wistar rat model | No | No | No |
| P. Honore, D. Donnelly-Roberts, M. T. Namovic, et al | 2006 | A-740003 [N-(1-{[(cyanoimino)(5-quinolinylamino) methyl]amino}-2,2-dimethylpropyl)-2-(3,4-dimethoxyphenyl)acetamide], a novel and selective P2X7 receptor antagonist, dose-dependently reduces neuropathic pain in the rat | No | No | No |
| K. Hori, N. Ozaki, S. Suzuki, Y. Sugiura | 2010 | Upregulations of P2X(3) and ASIC3 involve in hyperalgesia induced by cisplatin administration in rats | Yes | Yes | No |
| P. Horvath, J. Szilvassy, J. Nemeth, B. Peitl, M. Szilasi, Z. Szilvassy | 2005 | Decreased sensory neuropeptide release in isolated bronchi of rats with cisplatin-induced neuropathy | No | Yes | No |
| P. Horvath, Z. Szilvassy, B. Peitl, et al | 2006 | Changes in tracheo-bronchial sensory neuropeptide receptor gene expression pattern in rats with cisplatin-induced sensory neuropathy | No | Yes | No |
| Y. L. Hsieh, H. Y. Chen, C. H. Yang, C. C. Yang | 2017 | Analgesic Effects of Transcutaneous Ultrasound Nerve Stimulation in a Rat Model of Oxaliplatin-Induced Mechanical Hyperalgesia and Cold Allodynia | Yes | Yes | No |
| Y. L. Hsieh, Y. C. Fan, C. C. Yang | 2016 | Low-level laser therapy alleviates mechanical and cold allodynia induced by oxaliplatin administration in rats | Yes | Yes | No |
| L. Y. Hu, Y. Zhou, W. Q. Cui, et al | 2017 | Triggering receptor expressed on myeloid cells 2 (TREM2) dependent microglial activation promotes cisplatin-induced peripheral neuropathy in mice | No | No | No |
| K. Huang, D. Bian, B. Jiang, Q. Zhai, N. Gao, R. Wang | 2017 | TRPA1 contributed to the neuropathic pain induced by docetaxel treatment | Yes | No | No |
| Z. Z. Huang, D. Li, C. C. Liu, et al | 2014 | CX3CL1-mediated macrophage activation contributed to paclitaxel-induced DRG neuronal apoptosis and painful peripheral neuropathy | Yes | No | No |
| Z. Z. Huang, D. Li, H. D. Ou-Yang, et al | 2016 | Cerebrospinal Fluid Oxaliplatin Contributes to the Acute Pain Induced by Systemic Administration of Oxaliplatin | Yes | Yes | No |
| Z. Z. Huang, J. Y. Wei, H. D. Ou-Yang, et al | 2016 | mir-500-Mediated GAD67 Downregulation Contributes to Neuropathic Pain | Yes | No | Yes |
| P. Huehnchen, W. Boehmerle, M. Endres | 2013 | Assessment of paclitaxel induced sensory polyneuropathy with "Catwalk" automated gait analysis in mice | Yes | Yes | Yes |
| B. Y. Hwang, E. S. Kim, C. H. Kim, J. Y. Kwon, H. K. Kim | 2012 | Gender differences in paclitaxel-induced neuropathic pain behavior and analgesic response in rats | Yes | Yes | No |
| N. Ishii, H. Tsubouchi, A. Miura, et al | 2017 | Ghrelin alleviates paclitaxel-induced peripheral neuropathy by reducing oxidative stress and enhancing mitochondrial anti-oxidant functions in mice | No | No | No |
| N. Ito, A. Sakai, N. Miyake, et al | 2017 | miR-15b mediates oxaliplatin-induced chronic neuropathic pain through BACE1 down-regulation | Yes | Yes | No |
| S. Ito, K. Tajima, M. Nogawa, et al | 2012 | Etodolac, a cyclooxygenase-2 inhibitor, attenuates paclitaxel-induced peripheral neuropathy in a mouse model of mechanical allodynia | No | No | No |
| Y. Ito, S. Kobuchi, R. Shimizu, Y. Katsuyama | 2017 | Pharmacokinetic and toxicodynamic evaluation of oxaliplatin-induced neuropathy and hematological toxicity in rats | No | No | No |
| F. M. Ja’afer, F. B. Hamdan, F. H. Mohammed | 2006 | Vincristine-induced neuropathy in rat: electrophysiological and histological study | No | No | No |
| A. S. Jaggi, G. Kaur, A. Bali, N. Singh | 2017 | Pharmacological investigations on mast cell stabilizer and histamine receptor antagonists in vincristine-induced neuropathic pain | Yes | No | No |
| A. S. Jaggi, N. Singh | 2010 | Differential effect of spironolactone in chronic constriction injury and vincristine-induced neuropathic pain in rats | Yes | No | No |
| A. S. Jaggi, N. Singh | 2012 | Analgesic potential of intrathecal farnesyl thiosalicylic acid and GW 5074 in vincristine-induced neuropathic pain in rats | Yes | No | No |
| V. Jain, A. Jaggi, N. Singh | 2011 | Non-beneficial effects of rosiglitazone in oxaliplatin-induced cold hyperalgesia in rats | No | No | No |
| S. E. James, M. Dunham, M. Carrion-Jones, A. Murashov, Q. Lu | 2010 | Rho kinase inhibitor Y-27632 facilitates recovery from experimental peripheral neuropathy induced by anti-cancer drug cisplatin | Yes | Yes | No |
| S. M. Jamieson, J. Liu, T. Hsu, B. C. Baguley, M. J. McKeage | 2003 | Paclitaxel induces nucleolar enlargement in dorsal root ganglion neurons in vivo reducing oxaliplatin toxicity | No | No | No |
| S. M. F. Jamieson, J. Liu, B. Connor, M. J. McKeage | 2005 | Oxaliplatin causes selective atrophy of a subpopulation of dorsal root ganglion neurons without inducing cell loss | No | No | No |
| K. Janes, T. Doyle, L. Bryant, et al | 2013 | Bioenergetic deficits in peripheral nerve sensory axons during chemotherapy-induced neuropathic pain resulting from peroxynitrite-mediated post-translational nitration of mitochondrial superoxide dismutase | Yes | No | No |
| K. Janes, E. Esposito, T. Doyle, et al | 2014 | A3 adenosine receptor agonist prevents the development of paclitaxel-induced neuropathic pain by modulating spinal glial-restricted redox-dependent signaling pathways | Yes | No | No |
| K. Janes, J. W. Little, C. Li, et al | 2014 | The development and maintenance of paclitaxel-induced neuropathic pain require activation of the sphingosine 1-phosphate receptor subtype 1 | Yes | Yes | No |
| K. Janes, C. Wahlman, J. W. Little, et al | 2015 | Spinal neuroimmune activation is independent of T-cell infiltration and attenuated by A3 adenosine receptor agonists in a model of oxaliplatin-induced peripheral neuropathy | Yes | No | No |
| X. T. Ji, N. S. Qian, T. Zhang, et al | 2013 | Spinal astrocytic activation contributes to mechanical allodynia in a rat chemotherapy-induced neuropathic pain model | Yes | No | No |
| M. Jia, C. Wu, F. Gao, et al | 2017 | Activation of NLRP3 inflammasome in peripheral nerve contributes to paclitaxel-induced neuropathic pain | Yes | Yes | No |
| S. P. Jiang, Z. D. Zhang, L. M. Kang, Q. H. Wang, L. Zhang, H. P. Chen | 2016 | Celecoxib reverts oxaliplatin-induced neuropathic pain through inhibiting PI3K/Akt2 pathway in the mouse dorsal root ganglion | Yes | Yes | No |
| H. W. Jin, S. J. Flatters, W. H. Xiao, H. L. Mulhern, G. J. Bennett | 2008 | Prevention of paclitaxel-evoked painful peripheral neuropathy by acetyl-L-carnitine: effects on axonal mitochondria, sensory nerve fiber terminal arbors, and cutaneous Langerhans cells | Yes | No | No |
| H. Y. Jin, N. Y. Lee, H. A. Ko, K. A. Lee, T. S. Park | 2016 | Comparison of sensory tests and neuronal quantity of peripheral nerves between streptozotocin (STZ)-induced diabetic rats and paclitaxel (PAC)-treated rats | Yes | Yes | No |
| I. N. Johnston, M. Tan, J. Cao, et al | 2017 | Ibudilast reduces oxaliplatin-induced tactile allodynia and cognitive impairments in rats | Yes | No | No |
| B. S. Jortner, E. S. Cho | 1981 | Neurotoxicity of quelamycin in the rat | No | Yes | No |
| E. K. Joseph, X. Chen, O. Bogen, J. D. Levine | 2008 | Oxaliplatin acts on IB4-positive nociceptors to induce an oxidative stress-dependent acute painful peripheral neuropathy | No | No | No |
| E. K. Joseph, J. D. Levine | 2003 | Sexual dimorphism for protein kinase c epsilon signaling in a rat model of vincristine-induced painful peripheral neuropathy | No | No | No |
| E. K. Joseph, J. D. Levine | 2004 | Caspase signalling in neuropathic and inflammatory pain in the rat | No | No | No |
| E. K. Joseph, J. D. Levine | 2009 | Comparison of oxaliplatin- and cisplatin-induced painful peripheral neuropathy in the rat | No | No | No |
| Y. Jung, J. H. Lee, W. Kim, S. H. Yoon, S. K. Kim | 2017 | Anti-allodynic effect of Buja in a rat model of oxaliplatin-induced peripheral neuropathy via spinal astrocytes and pro-inflammatory cytokines suppression | No | Yes | No |
| U. Jungwirth, D. N. Xanthos, J. Gojo, et al | 2012 | Anticancer activity of methyl-substituted oxaliplatin analogs | Yes | Yes | No |
| J. Kahng, T. K. Kim, E. Y. Chung, Y. S. Kim, J. Y. Moon | 2015 | The effect of thioctic acid on allodynia in a rat vincristine-induced neuropathy model | No | No | No |
| J. Kamei, S. Hayashi, A. Sakai, et al | 2017 | Rikkunshito prevents paclitaxel-induced peripheral neuropathy through the suppression of the nuclear factor kappa B (NFkappaB) phosphorylation in spinal cord of mice | No | No | No |
| J. Kamei, C. Nozaki, A. Saitoh | 2006 | Effect of mexiletine on vincristine-induced painful neuropathy in mice | No | No | No |
| J. Kamei, N. Tamura, A. Saitoh | 2005 | Possible involvement of the spinal nitric oxide/cGMP pathway in vincristine-induced painful neuropathy in mice | Yes | No | No |
| S. Kamisli, O. Ciftci, A. Cetin, K. Kaya, O. Kamisli, H. Celik | 2014 | Fish oil protects the peripheral and central nervous systems against cisplatin-induced neurotoxicity | No | Yes | No |
| S. Kamisli, O. Ciftci, K. Kaya, A. Cetin, O. Kamisli, C. Ozcan | 2015 | Hesperidin protects brain and sciatic nerve tissues against cisplatin-induced oxidative, histological and electromyographical side effects in rats | No | Yes | No |
| O. Kanat, D. Bagdas, H. Y. Ozboluk, M. S. Gurun | 2013 | Preclinical evidence for the antihyperalgesic activity of CDP-choline in oxaliplatin-induced neuropathic pain | No | No | No |
| T. Kanbara, A. Nakamura, M. Shibasaki, et al | 2014 | Morphine and oxycodone, but not fentanyl, exhibit antinociceptive effects mediated by G-protein inwardly rectifying potassium (GIRK) channels in an oxaliplatin-induced neuropathy rat model | No | No | No |
| T. Kanbara, A. Nakamura, K. Takasu, et al | 2014 | The contribution of Gi/o protein to opioid antinociception in an oxaliplatin-induced neuropathy rat model | No | No | No |
| L. A. Kassem, M. M. Gamal El-Din, N. A. Yassin | 2011 | Mechanisms of vincristine-induced neurotoxicity: Possible reversal by erythropoietin | No | No | No |
| Y. Kato, Y. Tateai, M. Ohkubo, et al | 2014 | Gosha-jinki-gan reduced oxaliplatin-induced hypersensitivity to cold sensation and its effect would be related to suppression of the expression of TRPM8 and TRPA1 in rats | No | No | No |
| S. Katsuyama, H. Aso, A. Otowa, et al | 2014 | Antinociceptive Effects of the Serotonin and Noradrenaline Reuptake Inhibitors Milnacipran and Duloxetine on Vincristine-Induced Neuropathic Pain Model in Mice | No | No | No |
| S. Katsuyama, H. Kuwahata, T. Yagi, et al | 2012 | Intraplantar injection of linalool reduces paclitaxel-induced acute pain in mice | No | No | No |
| S. Katsuyama, K. Sato, T. Yagi, Y. Kishikawa, H. Nakamura | 2013 | Effects of repeated milnacipran and fluvoxamine treatment on mechanical allodynia in a mouse paclitaxel-induced neuropathic pain model | No | No | No |
| G. Kaur, A. S. Jaggi, N. Singh | 2010 | Exploring the potential effect of Ocimum sanctum in vincristine-induced neuropathic pain in rats | No | No | No |
| K. Kawakami, T. Chiba, N. Katagiri, et al | 2012 | Paclitaxel increases high voltage-dependent calcium channel current in dorsal root ganglion neurons of the rat | No | No | No |
| T. Kawashiri, N. Egashira, Y. Itoh, et al | 2009 | Neurotropin reverses paclitaxel-induced neuropathy without affecting anti-tumour efficacy | Yes | No | No |
| T. Kawashiri, N. Egashira, K. Kurobe, et al | 2012 | L type Ca2+ channel blockers prevent oxaliplatin-induced cold hyperalgesia and TRPM8 overexpression in rats | No | No | No |
| T. Kawashiri, N. Egashira, H. Watanabe, et al | 2011 | Prevention of oxaliplatin-induced mechanical allodynia and neurodegeneration by neurotropin in the rat model | Yes | No | No |
| D. Kawata, Z. Wu | 2017 | Regulatable Transgene Expression for Prevention of Chemotherapy-Induced Peripheral Neuropathy | No | No | No |
| I. A. Khasabova, S. Khasabov, J. Paz, C. Harding-Rose, D. A. Simone, V. S. Seybold | 2012 | Cannabinoid type-1 receptor reduces pain and neurotoxicity produced by chemotherapy | Yes | No | No |
| I. A. Khasabova, X. Yao, J. Paz, et al | 2014 | JZL184 is anti-hyperalgesic in a murine model of cisplatin-induced peripheral neuropathy | Yes | No | No |
| N. Kiguchi, T. Maeda, Y. Kobayashi, S. Kishioka | 2008 | Up-regulation of tumor necrosis factor-alpha in spinal cord contributes to vincristine-induced mechanical allodynia in mice | No | No | No |
| N. Kiguchi, T. Maeda, Y. Kobayashi, T. Kondo, M. Ozaki, S. Kishioka | 2008 | The critical role of invading peripheral macrophage-derived interleukin-6 in vincristine-induced mechanical allodynia in mice | No | No | No |
| T. J. Kilpatrick, S. Phan, K. Reardon, E. C. Lopes, S. S. Cheema | 2001 | Leukaemia inhibitory factor abrogates Paclitaxel-induced axonal atrophy in the Wistar rat | No | No | No |
| H. K. Kim, S. H. Hwang, S. Abdi | 2017 | Tempol ameliorates and prevents mechanical hyperalgesia in a rat model of chemotherapy-induced neuropathic pain | Yes | Yes | No |
| H. K. Kim, S. H. Hwang, S. O. Lee, S. H. Kim, S. Abdi | 2016 | Pentoxifylline Ameliorates Mechanical Hyperalgesia in a Rat Model of Chemotherapy-Induced Neuropathic Pain | Yes | Yes | No |
| H. K. Kim, S. H. Hwang, E. Oh, S. Abdi | 2017 | Rolipram, a selective phosphodiesterase 4 inhibitor, ameliorates mechanical hyperalgesia in a rat model of chemotherapy-induced neuropathic pain through inhibition of inflammatory cytokines in the dorsal root ganglion | Yes | No | No |
| H. K. Kim, J. Y. Kwon, C. Yoo, S. Abdi | 2015 | The Analgesic Effect of Rolipram, a Phosphodiesterase 4 Inhibitor, on Chemotherapy-Induced Neuropathic Pain in Rats | Yes | No | No |
| H. K. Kim, Y. P. Zhang, Y. S. Gwak, S. Abdi | 2010 | Phenyl N-tert-butylnitrone, a free radical scavenger, reduces mechanical allodynia in chemotherapy-induced neuropathic pain in rats | Yes | Yes | No |
| S. T. Kim, Y. H. Chung, H. S. Lee, et al | 2015 | Protective effects of phosphatidylcholine on oxaliplatin-induced neuropathy in rats | No | Yes | No |
| S. T. Kim, E. J. Kyung, J. S. Suh, et al | 2017 | Phosphatidylcholine attenuated docetaxel-induced peripheral neurotoxicity in rats | No | Yes | No |
| W. Kim, Y. Chung, S. Choi, B. I. Min, S. K. Kim | 2017 | Duloxetine Protects against Oxaliplatin-Induced Neuropathic Pain and Spinal Neuron Hyperexcitability in Rodents | Yes | No | No |
| W. Kim, M. J. Kim, D. Go, B. I. Min, H. S. Na, S. K. Kim | 2016 | Combined Effects of Bee Venom Acupuncture and Morphine on Oxaliplatin-Induced Neuropathic Pain in Mice | Yes | Yes | No |
| K. M. King, A. M. Myers, A. J. Soroka-Monzo, et al | 2017 | Single and combined effects of Delta(9) -tetrahydrocannabinol and cannabidiol in a mouse model of chemotherapy-induced neuropathic pain | Yes | Yes | No |
| R. Kirchmair, A. B. Tietz, E. Panagiotou, et al | 2007 | Therapeutic angiogenesis inhibits or rescues chemotherapy-induced peripheral neuropathy: Taxol- and thalidomide-induced injury of vasa nervorum is ameliorated by VEGF | Yes | Yes | No |
| R. Kirchmair, D. H. Walter, M. Ii, et al | 2005 | Antiangiogenesis mediates cisplatin-induced peripheral neuropathy attenuation or reversal by local vascular endothelial growth factor gene therapy without augmenting tumor growth | Yes | Yes | No |
| R. Kitamura, T. Andoh, H. Fushimi, K. Komatsu, N. Shibahara, Y. Kuraishi | 2013 | Involvement of descending monoaminergic systems in antiallodynic effect of goshajinkigan in oxaliplatintreated mice | No | No | No |
| R. Kitamura, T. Andoh, S. Mizoguchi, Y. Saito, H. Takahata, Y. Kuraishi | 2014 | Gabapentin inhibits bortezomib-induced mechanical allodynia through supraspinal action in mice | No | No | No |
| T. Kiya, T. Kawamata, A. Namiki, M. Yamakage | 2011 | Role of satellite cell-derived L-serine in the dorsal root ganglion in paclitaxel-induced painful peripheral neuropathy | No | No | No |
| M. H. Ko, M. E. Hu, Y. L. Hsieh, C. T. Lan, T. J. Tseng | 2014 | Peptidergic intraepidermal nerve fibers in the skin contribute to the neuropathic pain in paclitaxel-induced peripheral neuropathy | Yes | No | No |
| T. Kono, Y. Suzuki, K. Mizuno, et al | 2015 | Preventive effect of oral goshajinkigan on chronic oxaliplatin-induced hypoesthesia in rats | No | No | No |
| S. L. Kozachik, M. R. Opp, G. G. Page | 2015 | Recovery sleep does not mitigate the effects of prior sleep loss on paclitaxel-induced mechanical hypersensitivity in Sprague-Dawley rats | No | No | No |
| S. L. Kozachik, G. G. Page | 2016 | A Hyperresponsive HPA Axis May Confer Resilience Against Persistent Paclitaxel-Induced Mechanical Hypersensitivity | No | No | No |
| A. Krishnaveni, P. Gokila, K. Murugeswari, K. Periyanayagam | 2017 | Effect of hydroalcoholic extract of samanea saman barks in vincristine-induced peripheral neuropathy in rats | No | No | No |
| K. Krukowski, N. Eijkelkamp, G. Laumet, et al | 2016 | CD8+ T Cells and Endogenous IL-10 Are Required for Resolution of Chemotherapy-Induced Neuropathic Pain | Yes | No | No |
| K. Krukowski, J. Ma, O. Golonzhka, et al | 2017 | HDAC6 inhibition effectively reverses chemotherapy-induced peripheral neuropathy | Yes | Yes | No |
| K. Krukowski, C. H. Nijboer, X. Huo, A. Kavelaars, C. J. Heijnen | 2015 | Prevention of chemotherapy-induced peripheral neuropathy by the small-molecule inhibitor pifithrin-mu | Yes | No | No |
| U. Kuyrukluyildiz, I. Kupeli, Z. Bedir, et al | 2016 | The Effect of Anakinra on Paclitaxel-Induced Peripheral Neuropathic Pain in Rats | No | No | Yes |
| S. L. Kyte, W. Toma, D. Bagdas, et al | 2017 | Nicotine prevents and reverses paclitaxel-induced mechanical allodynia in a mouse model of CIPN | Yes | Yes | Yes |
| M. Leandri, M. Ghignotti, L. Emionite, S. Leandri, M. Cilli | 2012 | Electrophysiological features of the mouse tail nerves and their changes in chemotherapy induced peripheral neuropathy (CIPN) | No | No | No |
| A. Ledeboer, B. M. Jekich, E. M. Sloane, et al | 2007 | Intrathecal interleukin-10 gene therapy attenuates paclitaxel-induced mechanical allodynia and proinflammatory cytokine expression in dorsal root ganglia in rats | Yes | No | No |
| J. H. Lee, D. Go, W. Kim, et al | 2016 | Involvement of spinal muscarinic and serotonergic receptors in the anti-allodynic effect of electroacupuncture in rats with oxaliplatin-induced neuropathic pain | No | Yes | No |
| J. H. Lee, D. X. Li, H. Yoon, et al | 2014 | Serotonergic mechanism of the relieving effect of bee venom acupuncture on oxaliplatin-induced neuropathic cold allodynia in rats | No | Yes | No |
| J. S. Lee, Y. T. Kim, E. K. Jeon, H. S. Won, Y. S. Cho, Y. H. Ko | 2012 | Effect of green tea extracts on oxaliplatin-induced peripheral neuropathy in rats | No | Yes | No |
| M. Lee, S. Cho, K. Roh, et al | 2017 | Glutathione alleviated peripheral neuropathy in oxaliplatin-treated mice by removing aluminum from dorsal root ganglia | No | Yes | No |
| C. A. G. Lee-Kubli, N. A. Calcutt | 2014 | Altered rate-dependent depression of the spinal H-reflex as an indicator of spinal disinhibition in models of neuropathic pain | No | No | No |
| M. Leo, L. I. Schmitt, M. Erkel, M. Melnikova, J. Thomale, T. Hagenacker | 2017 | Cisplatin-induced neuropathic pain is mediated by upregulation of N-type voltage-gated calcium channels in dorsal root ganglion neurons | No | No | No |
| M. Leo, L. I. Schmitt, H. Jastrow, J. Thomale, C. Kleinschnitz, T. Hagenacker | 2017 | Cisplatin alters the function and expression of N-type voltage-gated calcium channels in the absence of morphological damage of sensory neurons | No | No | No |
| C. Leonetti, A. Biroccio, C. Gabellini, et al | 2003 | Alpha-tocopherol protects against cisplatin-induced toxicity without interfering with antitumor efficacy | No | No | No |
| D. Li, H. Chen, X. H. Luo, Y. Sun, W. Xia, Y. C. Xiong | 2016 | CX3CR1-Mediated Akt1 Activation Contributes to the Paclitaxel-Induced Painful Peripheral Neuropathy in Rats | Yes | No | Yes |
| D. Li, Z. Z. Huang, Y. Z. Ling, et al | 2015 | Up-regulation of CX3CL1 via Nuclear Factor-kappaB-dependent Histone Acetylation Is Involved in Paclitaxel-induced Peripheral Neuropathy | Yes | Yes | Yes |
| D. Li, W. Kim, D. Shin, Y. Jung, H. Bae, S. K. Kim | 2016 | Preventive Effects of Bee Venom Derived Phospholipase A(2) on Oxaliplatin-Induced Neuropathic Pain in Mice | Yes | No | No |
| D. Li, Y. Lee, W. Kim, K. Lee, H. Bae, S. K. Kim | 2015 | Analgesic Effects of Bee Venom Derived Phospholipase A2 in a Mouse Model of Oxaliplatin-Induced Neuropathic Pain | Yes | No | No |
| Y. Li, A. K. Kosturakis, R. M. Cassidy, et al | 2015 | MAPK signaling downstream to TLR4 contributes to paclitaxel-induced peripheral neuropathy | Yes | Yes | No |
| Y. Li, C. E. Tatsui, L. D. Rhines, et al | 2017 | Dorsal root ganglion neurons become hyperexcitable and increase expression of voltage-gated T-type calcium channels (Cav3.2) in paclitaxel-induced peripheral neuropathy | Yes | No | Yes |
| Z. Y. Li, Y. P. Zhang, J. Zhang, et al | 2016 | The possible involvement of JNK activation in the spinal dorsal horn in bortezomib-induced allodynia: the role of TNF-alpha and IL-1beta | Yes | No | Yes |
| B. S. Lim, H. J. Moon, D. X. Li, et al | 2013 | Effect of bee venom acupuncture on oxaliplatin-induced cold allodynia in rats | No | No | No |
| H. Lin, B. H. Heo, M. H. Yoon | 2015 | A New Rat Model of Cisplatin-induced Neuropathic Pain | No | No | Yes |
| H. M. Lin, L. F. Lin, Z. Z. Xia, et al | 2017 | Neuroprotective effects and UPLC-Q-TOF/MS-based active components identification of external applied a novel Wen-Luo-Tong microemulsion | No | Yes | No |
| X. Lin, A. S. Dhopeshwarkar, M. Huibregtse, K. Mackie, A. G. Hohmann | 2018 | Slowly Signaling G Protein-Biased CB2 Cannabinoid Receptor Agonist LY2828360 Suppresses Neuropathic Pain with Sustained Efficacy and Attenuates Morphine Tolerance and Dependence | Yes | Yes | Yes |
| B. Ling, N. Authier, D. Balayssac, A. Eschalier, F. Coudore | 2007 | Behavioral and pharmacological description of oxaliplatin-induced painful neuropathy in rat | Yes | Yes | No |
| B. Ling, F. Coudore, L. Decalonne, A. Eschalier, N. Authier | 2008 | Comparative antiallodynic activity of morphine, pregabalin and lidocaine in a rat model of neuropathic pain produced by one oxaliplatin injection | Yes | Yes | No |
| B. Ling, M. A. Coudore-Civiale, D. Balayssac, A. Eschalier, F. Coudore, N. Authier | 2007 | Behavioral and immunohistological assessment of painful neuropathy induced by a single oxaliplatin injection in the rat | Yes | Yes | No |
| Y. Z. Ling, Z. Y. Li, H. D. Ou-Yang, et al | 2017 | The inhibition of spinal synaptic plasticity mediated by activation of AMP-activated protein kinase signaling alleviates the acute pain induced by oxaliplatin | Yes | Yes | No |
| D. Linglu, L. Yuxiang, X. Yaqiong, et al | 2014 | Antinociceptive effect of matrine on vincristine-induced neuropathic pain model in mice | No | No | No |
| T. S. Lisse, L. J. Middleton, A. D. Pellegrini, et al | 2016 | Paclitaxel-induced epithelial damage and ectopic MMP-13 expression promotes neurotoxicity in zebrafish | No | No | No |
| C. Liu, S. Luan, H. OuYang, et al | 2016 | Upregulation of CCL2 via ATF3/c-Jun interaction mediated the Bortezomib-induced peripheral neuropathy | Yes | No | No |
| C. C. Liu, N. Lu, Y. Cui, et al | 2010 | Prevention of paclitaxel-induced allodynia by minocycline: Effect on loss of peripheral nerve fibers and infiltration of macrophages in rats | Yes | No | No |
| H. Liu, C. H. Wang, H. Yang, F. Wang | 2015 | Effects of fisetin on oxaliplatin-induced neuropathic pain in mice | No | Yes | No |
| H. P. Liu, T. W. Ren, W. J. Yan, J. Liu, R. B. Liu | 2016 | Ellagic acid alleviates inflammatory pain and paclitaxel-induced neuropathic pain in murine models | No | No | No |
| X. Liu, G. Zhang, L. Dong, et al | 2013 | Repeated administration of mirtazapine attenuates oxaliplatin-induced mechanical allodynia and spinal NR2B up-regulation in rats | Yes | Yes | No |
| P. M. LoCoco, A. L. Risinger, H. R. Smith, T. S. Chavera, K. A. Berg, W. P. Clarke | 2017 | Pharmacological augmentation of nicotinamide phosphoribosyltransferase (NAMPT) protects against paclitaxel-induced peripheral neuropathy | Yes | Yes | No |
| S. Lolignier, C. Bonnet, C. Gaudioso, et al | 2015 | The Nav1.9 Channel Is a Key Determinant of Cold Pain Sensation and Cold Allodynia | Yes | No | No |
| D. Lucas, C. Scheiermann, A. Chow, et al | 2013 | Chemotherapy-induced bone marrow nerve injury impairs hematopoietic regeneration | No | No | No |
| J. Luo, A. Bavencoffe, P. Yang, et al | 2017 | Zinc inhibits TRPV1 to alleviate chemotherapy-induced neuropathic pain | Yes | No | No |
| X. Luo, B. Fitzsimmons, A. Mohan, et al | 2017 | Intrathecal administration of antisense oligonucleotide against p38alpha but not p38beta MAP kinase isoform reduces neuropathic and postoperative pain and TLR4-induced pain in male mice | Yes | No | No |
| J. J. Lynch, 3rd, C. L. Wade, J. P. Mikusa, M. W. Decker, P. Honore | 2005 | ABT-594 (a nicotinic acetylcholine agonist): anti-allodynia in a rat chemotherapy-induced pain model | No | No | No |
| J. J. Lynch, 3rd, C. L. Wade, C. M. Zhong, J. P. Mikusa, P. Honore | 2004 | Attenuation of mechanical allodynia by clinically utilized drugs in a rat chemotherapy-induced neuropathic pain model | No | No | No |
| M. A. Maj, J. Ma, K. N. Krukowski, A. Kavelaars, C. J. Heijnen | 2017 | Inhibition of mitochondrial p53 accumulation by PFT-micro prevents cisplatin-induced peripheral neuropathy | Yes | No | No |
| P. G. Makker, S. S. Duffy, J. G. Lees, et al | 2017 | Characterisation of Immune and Neuroinflammatory Changes Associated with Chemotherapy-Induced Peripheral Neuropathy | Yes | No | No |
| A. Mangaiarkkarasi, S. Rameshkannan, R. M. Ali | 2015 | Effect of Gabapentin and Pregabalin in Rat Model of Taxol Induced Neuropathic Pain | Yes | Yes | No |
| F. Marchand, A. Alloui, T. Pelissier, et al | 2003 | Evidence for an antihyperalgesic effect of venlafaxine in vincristine-induced neuropathy in rat | No | No | No |
| W. Masocha | 2014 | Paclitaxel-induced hyposensitivity to nociceptive chemical stimulation in mice can be prevented by treatment with minocycline | No | No | No |
| W. Masocha | 2015 | Comprehensive analysis of the GABAergic system gene expression profile in the anterior cingulate cortex of mice with Paclitaxel-induced neuropathic pain | No | No | No |
| W. Masocha | 2016 | Gene expression profile of sodium channel subunits in the anterior cingulate cortex during experimental paclitaxel-induced neuropathic pain in mice | No | No | No |
| W. Masocha, S. S. Parvathy | 2016 | Preventative and therapeutic effects of a GABA transporter 1 inhibitor administered systemically in a mouse model of paclitaxel-induced neuropathic pain | Yes | No | No |
| F. Massicot, G. Hache, L. David, et al | 2013 | P2X7 Cell Death Receptor Activation and Mitochondrial Impairment in Oxaliplatin-Induced Apoptosis and Neuronal Injury: Cellular Mechanisms and In Vivo Approach | Yes | No | No |
| K. Masuguchi, H. Watanabe, T. Kawashiri, et al | 2014 | Neurotropin(R) relieves oxaliplatin-induced neuropathy via Gi protein-coupled receptors in the monoaminergic descending pain inhibitory system | Yes | No | No |
| M. Matsumoto, M. Inoue, A. Hald, W. Xie, H. Ueda | 2006 | Inhibition of paclitaxel-induced A-fiber hypersensitization by gabapentin | Yes | No | No |
| Y. Matsumura, Y. Yokoyama, H. Hirakawa, T. Shigeto, M. Futagami, H. Mizunuma | 2014 | The prophylactic effects of a traditional Japanese medicine, goshajinkigan, on paclitaxel-induced peripheral neuropathy and its mechanism of action | Yes | No | No |
| B. McCormick, D. A. Lowes, L. Colvin, C. Torsney, H. F. Galley | 2016 | MitoVitE, a mitochondria-targeted antioxidant, limits paclitaxel-induced oxidative stress and mitochondrial damage in vitro, and paclitaxel-induced mechanical hypersensitivity in a rat pain model | Yes | No | No |
| M. J. McKeage, F. E. Boxall, M. Jones, K. R. Harrap | 1994 | Lack of neurotoxicity of oral bisacetatoamminedichlorocyclohexylamine-platinum(IV) in comparison to cisplatin and tetraplatin in the rat | No | Yes | No |
| G. Melli, C. Jack, G. L. Lambrinos, M. Ringkamp, A. Hoke | 2006 | Erythropoietin protects sensory axons against paclitaxel-induced distal degeneration | No | No | No |
| X. Meng, Y. Zhang, A. Li, et al | 2011 | The effects of opioid receptor antagonists on electroacupuncture-produced anti-allodynia/hyperalgesia in rats with paclitaxel-evoked peripheral neuropathy | Yes | Yes | No |
| C. Meregalli, A. Canta, V. A. Carozzi, et al | 2010 | Bortezomib-induced painful neuropathy in rats: a behavioral, neurophysiological and pathological study in rats | Yes | Yes | No |
| C. Meregalli, C. Ceresa, A. Canta, et al | 2012 | CR4056, a new analgesic I2 ligand, is highly effective against bortezomib-induced painful neuropathy in rats | No | Yes | No |
| C. Meregalli, A. Chiorazzi, V. A. Carozzi, et al | 2014 | Evaluation of tubulin polymerization and chronic inhibition of proteasome as citotoxicity mechanisms in bortezomib-induced peripheral neuropathy | No | No | No |
| L. Meyer, C. Patte-Mensah, O. Taleb, A. G. Mensah-Nyagan | 2010 | Cellular and functional evidence for a protective action of neurosteroids against vincristine chemotherapy-induced painful neuropathy | Yes | No | No |
| L. Meyer, C. Patte-Mensah, O. Taleb, A. G. Mensah-Nyagan | 2011 | Allopregnanolone prevents and suppresses oxaliplatin-evoked painful neuropathy: multi-parametric assessment and direct evidence | Yes | No | No |
| L. Meyer, C. Patte-Mensah, O. Taleb, A. G. Mensah-Nyagan | 2013 | Neurosteroid 3alpha-androstanediol efficiently counteracts paclitaxel-induced peripheral neuropathy and painful symptoms | Yes | No | No |
| L. Micheli, L. Di Cesare Mannelli, A. Rizzi, et al | 2015 | Intrathecal administration of nociceptin/orphanin FQ receptor agonists in rats: A strategy to relieve chemotherapy-induced neuropathic hypersensitivity | Yes | No | No |
| B. Michot, V. Kayser, G. Bastian, S. Bourgoin, M. Hamon | 2014 | Differential pharmacological alleviation of oxaliplatin-induced hyperalgesia/allodynia at cephalic versus extra-cephalic level in rodents | No | No | No |
| Y. Mihara, N. Egashira, H. Sada, et al | 2011 | Involvement of spinal NR2B-containing NMDA receptors in oxaliplatin-induced mechanical allodynia in rats | Yes | No | No |
| S. Mizoguchi, T. Andoh, T. Yakura, Y. Kuraishi | 2016 | Involvement of c-Myc-mediated transient receptor potential melastatin 8 expression in oxaliplatin-induced cold allodynia in mice | No | No | No |
| K. Mizuno, T. Kono, Y. Suzuki, et al | 2014 | Goshajinkigan, a traditional Japanese medicine, prevents oxaliplatin-induced acute peripheral neuropathy by suppressing functional alteration of TRP channels in rat | No | No | No |
| K. Mizuno, K. Shibata, R. Komatsu, Y. Omiya, Y. Kase, S. Koizumi | 2016 | An effective therapeutic approach for oxaliplatin-induced peripheral neuropathy using a combination therapy with goshajinkigan and bushi | No | No | No |
| M. Mo, I. Erdelyi, K. Szigeti-Buck, J. H. Benbow, B. E. Ehrlich | 2012 | Prevention of paclitaxel-induced peripheral neuropathy by lithium pretreatment | No | Yes | No |
| H. J. Moon, B. S. Lim, D. I. Lee, et al | 2014 | Effects of electroacupuncture on oxaliplatin-induced neuropathic cold hypersensitivity in rats | No | No | No |
| D. R. M. Moreira, D. S. Santos, R. F. D. Espirito Santo, et al | 2017 | Structural improvement of new thiazolidinones compounds with antinociceptive activity in experimental chemotherapy-induced painful neuropathy | No | No | No |
| T. Mori, T. Kanbara, M. Harumiya, et al | 2014 | Establishment of opioid-induced rewarding effects under oxaliplatin- and Paclitaxel-induced neuropathy in rats | No | No | No |
| L. J. Muller, R. Gerritsen Van Der Hoop, C. M. Moorer-Van Delft, W. H. Gispen, E. W. Roubos | 1990 | Morphological and electrophysiological study of the effects of cisplatin and ORG.2766 on rat spinal ganglion neurons | No | No | No |
| G. Mustafa, E. M. Anderson, Y. Bokrand-Donatelli, J. K. Neubert, R. M. Caudle | 2013 | Anti-nociceptive effect of a conjugate of substance P and light chain of botulinum neurotoxin type A | No | No | No |
| A. Muthuraman, N. Singh | 2011 | Attenuating effect of hydroalcoholic extract of Acorus calamus in vincristine-induced painful neuropathy in rats | No | No | No |
| A. Muthuraman, N. Singh, A. S. Jaggi | 2011 | Protective effect of Acorus calamus L. in rat model of vincristine induced painful neuropathy: an evidence of anti-inflammatory and anti-oxidative activity | No | No | No |
| M. Naguib, J. J. Xu, P. Diaz, et al | 2012 | Prevention of paclitaxel-induced neuropathy through activation of the central cannabinoid type 2 receptor system | Yes | No | No |
| Y. Nakahashi, Y. Kamiya, K. Funakoshi, et al | 2014 | Role of nerve growth factor-tyrosine kinase receptor A signaling in paclitaxel-induced peripheral neuropathy in rats | No | No | No |
| A. Namvaran-Abbas-Abad, F. Tavakkoli | 2012 | Antinociceptive effect of Salvia extract on cisplatin-induced hyperalgesia in mice | No | Yes | No |
| H. Nashawi, W. Masocha, I. O. Edafiogho, S. B. Kombian | 2016 | Paclitaxel Causes Electrophysiological Changes in the Anterior Cingulate Cortex via Modulation of the gamma-Aminobutyric Acid-ergic System | No | No | No |
| R. Nassini, M. Gees, S. Harrison, et al | 2011 | Oxaliplatin elicits mechanical and cold allodynia in rodents via TRPA1 receptor stimulation | Yes | Yes | No |
| C. Nativi, R. Gualdani, E. Dragoni, et al | 2013 | A TRPA1 antagonist reverts oxaliplatin-induced neuropathic pain | No | No | No |
| A. M. Nayebi, H. Sharifi, M. Ramadzani, H. Rezazadeh | 2012 | Effect of acute and chronic administration of carbamazepine on Cisplatin-induced hyperalgesia in rats | No | Yes | No |
| H. Neelakantan, S. J. Ward, E. A. Walker | 2016 | Effects of paclitaxel on mechanical sensitivity and morphine reward in male and female C57Bl6 mice | No | Yes | No |
| I. S. A. Neuman, P. S. Heijmen, R. C. Peters, G. S. F. Ruigt | 1993 | Fish electroreception as a model for vincristine-induced neuropathies and a possible preventive role for ORG 2766 treatment | No | No | No |
| B. Nie, C. Liu, X. Bai, et al | 2017 | AKAP150 involved in paclitaxel-induced neuropathic pain via inhibiting CN/NFAT2 pathway and downregulating IL-4 | Yes | Yes | No |
| B. Nie, S. Zhang, Z. Huang, et al | 2017 | Synergistic Interaction Between Dexmedetomidine and Ulinastatin Against Vincristine-Induced Neuropathic Pain in Rats | Yes | Yes | No |
| J. Nie, X. Liu | 2017 | Leonurine attenuates hyperalgesia in mice with induced adenomyosis | No | Yes | No |
| J. Nie, X. Liu | 2017 | Quercetin alleviates generalized hyperalgesia in mice with induced adenomyosis | No | Yes | No |
| F. R. Nieto, C. M. Cendan, F. J. Canizares, et al | 2014 | Genetic inactivation and pharmacological blockade of sigma-1 receptors prevent paclitaxel-induced sensory-nerve mitochondrial abnormalities and neuropathic pain in mice | Yes | No | No |
| F. R. Nieto, J. M. Entrena, C. M. Cendan, E. D. Pozo, J. M. Vela, J. M. Baeyens | 2008 | Tetrodotoxin inhibits the development and expression of neuropathic pain induced by paclitaxel in mice | Yes | No | No |
| K. Nishida, S. Kuchiiwa, S. Oiso, et al | 2008 | Up-regulation of matrix metalloproteinase-3 in the dorsal root ganglion of rats with paclitaxel-induced neuropathy | Yes | No | No |
| T. Nishida, M. Tsubota, Y. Kawaishi, et al | 2016 | Involvement of high mobility group box 1 in the development and maintenance of chemotherapy-induced peripheral neuropathy in rats | No | Yes | No |
| K. Noda, H. Akita, M. Ogata, M. Saji | 2014 | Paclitaxel-induced hyperalgesia modulates negative affective component of pain and NR1 receptor expression in the frontal cortex in rats | No | No | No |
| H. Nodera, A. Spieker, M. Sung, S. Rutkove | 2011 | Neuroprotective effects of Kv7 channel agonist, retigabine, for cisplatin-induced peripheral neuropathy | No | Yes | No |
| M. Norcini, E. Vivoli, N. Galeotti, E. Bianchi, A. Bartolini, C. Ghelardini | 2009 | Supraspinal role of protein kinase C in oxaliplatin-induced neuropathy in rat | Yes | Yes | No |
| N. Nozaki-Taguchi, S. R. Chaplan, E. S. Higuera, R. C. Ajakwe, T. L. Yaksh | 2001 | Vincristine-induced allodynia in the rat | No | Yes | No |
| R. Ochi-ishi, K. Nagata, T. Inoue, H. Tozaki-Saitoh, M. Tsuda, K. Inoue | 2014 | Involvement of the chemokine CCL3 and the purinoceptor P2X7 in the spinal cord in paclitaxel-induced mechanical allodynia | No | No | No |
| T. Ogawa, Y. Mimura, K. Isowa, et al | 2001 | An antimicrotubule agent, TZT-1027, does not induce neuropathologic alterations which are detected after administration of vincristine or paclitaxel in animal models | No | No | No |
| M. Ohsawa, S. Otake, T. Murakami, S. Yamamoto, T. Makino, H. Ono | 2014 | Gabapentin prevents oxaliplatin-induced mechanical hyperalgesia in mice | No | No | No |
| K. Okubo, T. Takahashi, F. Sekiguchi, et al | 2011 | Inhibition of T-type calcium channels and hydrogen sulfide-forming enzyme reverses paclitaxel-evoked neuropathic hyperalgesia in rats | No | No | No |
| E. A. Old, S. Nadkarni, J. Grist, et al | 2014 | Monocytes expressing CX3CR1 orchestrate the development of vincristine-induced pain | Yes | Yes | No |
| B. Orhan, S. Yalcin, G. Nurlu, D. Zeybek, S. Muftuoglu | 2004 | Erythropoietin against cisplatin-induced peripheral neurotoxicity in rats | No | Yes | No |
| G. Ozturk, O. Anlar, E. Erdogan, M. Kosem, H. Ozbek, A. Turker | 2004 | The effect of Ginkgo extract EGb761 in cisplatin-induced peripheral neuropathy in mice | No | Yes | No |
| G. Ozturk, E. Erdogan, O. Anlar, M. Kosem, M. Taspinar | 2005 | Effect of leukemia inhibitory factor in experimental cisplatin neuropathy in mice | No | Yes | No |
| A. Pacini, L. Micheli, M. Maresca, et al | 2016 | The alpha9alpha10 nicotinic receptor antagonist alpha-conotoxin RgIA prevents neuropathic pain induced by oxaliplatin treatment | Yes | No | No |
| B. Y. Park, S. H. Park, W. M. Kim, M. H. Yoon, H. G. Lee | 2010 | Antinociceptive Effect of Memantine and Morphine on Vincristine-induced Peripheral Neuropathy in Rats | Yes | No | No |
| H. J. Park, Y. H. Kim, H. J. Koh, et al | 2012 | Analgesic effects of dexmedetomidine in vincristine-evoked painful neuropathic rats | Yes | Yes | No |
| H. J. Park, H. G. Lee, Y. S. Kim, et al | 2012 | Ginkgo biloba extract attenuates hyperalgesia in a rat model of vincristine-induced peripheral neuropathy | Yes | Yes | No |
| H. J. Park, M. J. Marino, E. S. Rondon, Q. Xu, T. L. Yaksh | 2015 | The effects of intraplantar and intrathecal botulinum toxin type B on tactile allodynia in mono and polyneuropathy in the mouse | Yes | No | No |
| H. J. Park, J. A. Stokes, M. Corr, T. L. Yaksh | 2014 | Toll-like receptor signaling regulates cisplatin-induced mechanical allodynia in mice | Yes | No | No |
| H. J. Park, J. A. Stokes, E. Pirie, J. Skahen, Y. Shtaerman, T. L. Yaksh | 2013 | Persistent hyperalgesia in the cisplatin-treated mouse as defined by threshold measures, the conditioned place preference paradigm, and changes in dorsal root ganglia activated transcription factor 3: the effects of gabapentin, ketorolac, and etanercept | No | Yes | No |
| J. H. Park, J. Chae, K. Roh, et al | 2015 | Oxaliplatin-Induced Peripheral Neuropathy via TRPA1 Stimulation in Mice Dorsal Root Ganglion Is Correlated with Aluminum Accumulation | Yes | Yes | No |
| J. S. Park, S. Kim, A. Hoke | 2015 | An exercise regimen prevents development paclitaxel induced peripheral neuropathy in a mouse model | Yes | Yes | No |
| S. S. Parvathy, W. Masocha | 2013 | Matrix metalloproteinase inhibitor COL-3 prevents the development of paclitaxel-induced hyperalgesia in mice | Yes | No | No |
| S. S. Parvathy, W. Masocha | 2015 | Coadministration of indomethacin and minocycline attenuates established paclitaxel-induced neuropathic thermal hyperalgesia: Involvement of cannabinoid CB1 receptors | No | No | No |
| D. Pascual, C. Goicoechea, E. Burgos, M. I. Martin | 2010 | Antinociceptive effect of three common analgesic drugs on peripheral neuropathy induced by paclitaxel in rats | Yes | Yes | No |
| D. Pascual, C. Goicoechea, M. Suardiaz, M. I. Martin | 2005 | A cannabinoid agonist, WIN 55,212-2, reduces neuropathic nociception induced by paclitaxel in rats | Yes | Yes | No |
| K. F. Paton, N. Kumar, R. S. Crowley, J. L. Harper, T. E. Prisinzano, B. M. Kivell | 2017 | The analgesic and anti-inflammatory effects of Salvinorin A analogue beta-tetrahydropyran Salvinorin B in mice | Yes | No | No |
| P. Peng, Q. Xi, S. Xia, et al | 2012 | Pregabalin attenuates docetaxel-induced neuropathy in rats | No | Yes | No |
| E. Persohn, A. Canta, S. Schoepfer, et al | 2005 | Morphological and morphometric analysis of paclitaxel and docetaxel-induced peripheral neuropathy in rats | No | No | No |
| C. M. Peters, J. M. Jimenez-Andrade, B. M. Jonas, et al | 2007 | Intravenous paclitaxel administration in the rat induces a peripheral sensory neuropathy characterized by macrophage infiltration and injury to sensory neurons and their supporting cells | Yes | No | No |
| M. Petrini, F. Vaglini, G. Cervetti, et al | 1999 | Is lithium able to reverse neurological damage induced by vinca alkaloids? | No | No | No |
| M. Pevida, A. Lastra, A. Hidalgo, A. Baamonde, L. Menendez | 2013 | Spinal CCL2 and microglial activation are involved in paclitaxel-evoked cold hyperalgesia | No | No | No |
| C. Pisano, G. Pratesi, D. Laccabue, et al | 2003 | Paclitaxel and Cisplatin-induced neurotoxicity: a protective role of acetyl-L-carnitine | Yes | Yes | No |
| J. L. Podratz, H. Lee, P. Knorr, et al | 2017 | Cisplatin induces mitochondrial deficits in Drosophila larval segmental nerve | No | No | No |
| J. L. Podratz, N. P. Staff, J. B. Boesche, et al | 2013 | An automated climbing apparatus to measure chemotherapy-induced neurotoxicity in Drosophila melanogaster | No | No | No |
| J. L. Podratz, N. P. Staff, D. Froemel, et al | 2011 | Drosophila melanogaster: a new model to study cisplatin-induced neurotoxicity | No | No | No |
| R. C. Polomano, A. J. Mannes, U. S. Clark, G. J. Bennett | 2001 | A painful peripheral neuropathy in the rat produced by the chemotherapeutic drug, paclitaxel | Yes | No | No |
| B. Ponsati, C. Carreno, V. Curto-Reyes, et al | 2012 | An inhibitor of neuronal exocytosis (DD04107) displays long-lasting in vivo activity against chronic inflammatory and neuropathic pain | Yes | Yes | No |
| N. Pourmohammadi, H. Alimoradi, S. E. Mehr, et al | 2012 | Lithium Attenuates Peripheral Neuropathy Induced by Paclitaxel in Rats | No | Yes | No |
| P. F. Pradat, F. Finiels, P. Kennel, et al | 2001 | Partial prevention of cisplatin-induced neuropathy by electroporation-mediated nonviral gene transfer | No | Yes | No |
| E. J. Rahn, L. Deng, G. A. Thakur, et al | 2014 | Prophylactic cannabinoid administration blocks the development of paclitaxel-induced neuropathic nociception during analgesic treatment and following cessation of drug delivery | Yes | Yes | No |
| E. J. Rahn, A. Makriyannis, A. G. Hohmann | 2007 | Activation of cannabinoid CB1 and CB2 receptors suppresses neuropathic nociception evoked by the chemotherapeutic agent vincristine in rats | Yes | No | No |
| E. J. Rahn, A. M. Zvonok, G. A. Thakur, A. D. Khanolkar, A. Makriyannis, A. G. Hohmann | 2008 | Selective activation of cannabinoid CB2 receptors suppresses neuropathic nociception induced by treatment with the chemotherapeutic agent paclitaxel in rats | Yes | No | No |
| C. L. Renn, V. A. Carozzi, P. Rhee, D. Gallop, S. G. Dorsey, G. Cavaletti | 2011 | Multimodal assessment of painful peripheral neuropathy induced by chronic oxaliplatin-based chemotherapy in mice | Yes | Yes | No |
| F. K. Rigo, G. D. Dalmolin, G. Trevisan, et al | 2013 | Effect of omega-conotoxin MVIIA and Phalpha1beta on paclitaxel-induced acute and chronic pain | Yes | Yes | No |
| C. R. Robinson, P. M. Dougherty | 2015 | Spinal astrocyte gap junction and glutamate transporter expression contributes to a rat model of bortezomib-induced peripheral neuropathy | No | No | No |
| C. R. Robinson, H. Zhang, P. M. Dougherty | 2014 | Altered discharges of spinal neurons parallel the behavioral phenotype shown by rats with bortezomib related chemotherapy induced peripheral neuropathy | No | No | No |
| C. R. Robinson, H. Zhang, P. M. Dougherty | 2014 | Astrocytes, but not microglia, are activated in oxaliplatin and bortezomib-induced peripheral neuropathy in the rat | No | No | No |
| V. Rodriguez-Menendez, A. Gilardini, M. Bossi, et al | 2008 | Valproate protective effects on cisplatin-induced peripheral neuropathy: an in vitro and in vivo study | No | No | No |
| I. Roglio, R. Bianchi, F. Camozzi, et al | 2009 | Docetaxel-induced peripheral neuropathy: protective effects of dihydroprogesterone and progesterone in an experimental model | No | Yes | No |
| H. K. Romero, S. B. Christensen, L. Di Cesare Mannelli, et al | 2017 | Inhibition of alpha9alpha10 nicotinic acetylcholine receptors prevents chemotherapy-induced neuropathic pain | Yes | No | No |
| J. Ruiz-Medina, A. Baulies, S. A. Bura, O. Valverde | 2013 | Paclitaxel-induced neuropathic pain is age dependent and devolves on glial response | No | No | No |
| J. W. Russell, J. S. Gill, E. J. Sorenson, D. A. Schultz, A. J. Windebank | 2001 | Suramin-induced neuropathy in an animal model | No | No | No |
| T. Ruyang, Z. Yang, F. Wei | 2015 | Gabapentin prevents oxaliplatin-induced central sensitization in the dorsal horn neurons in rats | No | No | No |
| H. Sada, N. Egashira, S. Ushio, T. Kawashiri, M. Shirahama, R. Oishi | 2012 | Repeated administration of amitriptyline reduces oxaliplatin-induced mechanical allodynia in rats | Yes | No | No |
| L. Saha, D. Hota, A. Chakrabarti | 2012 | Evaluation of lercanidipine in Paclitaxel-induced neuropathic pain model in rat: a preliminary study | No | No | No |
| F. Saika, N. Kiguchi, Y. Kobayashi, et al | 2009 | Suppressive effect of imipramine on vincristine-induced mechanical allodynia in mice | No | No | No |
| A. Sakamoto, T. Andoh, Y. Kuraishi | 2016 | Involvement of mast cells and proteinase-activated receptor 2 in oxaliplatin-induced mechanical allodynia in mice | Yes | No | No |
| M. Sakurai, N. Egashira, T. Kawashiri, T. Yano, H. Ikesue, R. Oishi | 2009 | Oxaliplatin-induced neuropathy in the rat: involvement of oxalate in cold hyperalgesia but not mechanical allodynia | Yes | No | No |
| K. Salat, A. Cios, E. Wyska, et al | 2014 | Antiallodynic and antihyperalgesic activity of 3-[4-(3-trifluoromethyl-phenyl)-piperazin-1-yl]-dihydrofuran-2-one compared to pregabalin in chemotherapy-induced neuropathic pain in mice | Yes | Yes | No |
| K. Salat, A. Furgala, R. Salat | 2017 | Evaluation of cebranopadol, a dually acting nociceptin/orphanin FQ and opioid receptor agonist in mouse models of acute, tonic, and chemotherapy-induced neuropathic pain | Yes | Yes | No |
| K. Salat, M. Kolaczkowski, A. Furgala, et al | 2017 | Antinociceptive, antiallodynic and antihyperalgesic effects of the 5-HT1A receptor selective agonist, NLX-112 in mouse models of pain | No | Yes | No |
| V. K. Samineni, L. S. Premkumar, C. L. Faingold | 2017 | Neuropathic pain-induced enhancement of spontaneous and pain-evoked neuronal activity in the periaqueductal gray that is attenuated by gabapentin | Yes | Yes | No |
| M. D. Sanna, C. Ghelardini, N. Galeotti | 2017 | St. John's Wort Potentiates anti-Nociceptive Effects of Morphine in Mice Models of Neuropathic Pain | Yes | Yes | Yes |
| K. A. Schappacher, L. Styczynski, M. L. Baccei | 2017 | Early life vincristine exposure evokes mechanical pain hypersensitivity in the developing rat | Yes | Yes | No |
| D. Schellingerhout, L. G. LeRoux, B. P. Hobbs, S. Bredow | 2012 | Impairment of retrograde neuronal transport in oxaliplatin-induced neuropathy demonstrated by molecular imaging | No | No | No |
| T. E. Schwingel, C. P. Klein, N. F. Nicoletti, et al | 2014 | Effects of the compounds resveratrol, rutin, quercetin, and quercetin nanoemulsion on oxaliplatin-induced hepatotoxicity and neurotoxicity in mice | No | Yes | No |
| D. Screnci, M. J. McKeage, P. Galettis, T. W. Hambley, B. D. Palmer, B. C. Baguley | 2000 | Relationships between hydrophobicity, reactivity, accumulation and peripheral nerve toxicity of a series of platinum drugs | No | No | No |
| G. C. Segat, M. N. Manjavachi, D. O. Matias, et al | 2017 | Antiallodynic effect of beta-caryophyllene on paclitaxel-induced peripheral neuropathy in mice | Yes | Yes | No |
| Y. Seto, F. Okazaki, K. Horikawa, J. Zhang, H. Sasaki, H. To | 2016 | Influence of dosing times on cisplatin-induced peripheral neuropathy in rats | No | No | No |
| Y. Seto, M. Takase, Y. Tsuji, H. To | 2017 | Pregabalin reduces cisplatin-induced mechanical allodynia in rats | No | No | No |
| M. Shabani, M. Nazeri, S. Parsania, et al | 2012 | Walnut consumption protects rats against cisplatin-induced neurotoxicity | No | Yes | No |
| M. Shahid, F. Subhan, N. Ahmad, R. D. E. Sewell | 2017 | The flavonoid 6-methoxyflavone allays cisplatin-induced neuropathic allodynia and hypoalgesia | No | Yes | No |
| N. Sharawy, L. Rashed, M. F. Youakim | 2015 | Evaluation of multi-neuroprotective effects of erythropoietin using cisplatin induced peripheral neurotoxicity model | No | Yes | No |
| Y. Shen, Z. J. Zhang, M. D. Zhu, B. C. Jiang, T. Yang, Y. J. Gao | 2015 | Exogenous induction of HO-1 alleviates vincristine-induced neuropathic pain by reducing spinal glial activation in mice | No | No | No |
| Y. Shidahara, S. Ogawa, M. Nakamura, et al | 2016 | Pharmacological comparison of a nonhuman primate and a rat model of oxaliplatin-induced neuropathic cold hypersensitivity | Yes | Yes | No |
| H. Shimizu, M. Ohgoh, Y. Momose, Y. Nishizawa, H. Ogura | 2002 | Massive cell death of cerebellar granule neurons accompanied with caspase-3-like protease activation and subsequent motor discoordination after intracerebroventricular injection of vincristine in mice | Yes | No | No |
| Y. K. Shin, S. Y. Jang, H. K. Lee, et al | 2010 | Pathological adaptive responses of schwann cells to endoplasmic reticulum stress in bortezomib-induced peripheral neuropathy | No | No | No |
| M. Shirahama, S. Ushio, N. Egashira, et al | 2012 | Inhibition of Ca2+/Calmodulin-dependent protein kinase II reverses oxaliplatin-induced mechanical allodynia in Rats | Yes | No | No |
| C. Siau, G. J. Bennett | 2006 | Dysregulation of cellular calcium homeostasis in chemotherapy-evoked painful peripheral neuropathy | No | No | No |
| C. Siau, W. Xiao, G. J. Bennett | 2006 | Paclitaxel- and vincristine-evoked painful peripheral neuropathies: loss of epidermal innervation and activation of Langerhans cells | Yes | No | No |
| M. Sisignano, C. Angioni, C. K. Park, et al | 2016 | Targeting CYP2J to reduce paclitaxel-induced peripheral neuropathic pain | No | No | No |
| R. A. Slivicki, Y. O. Ali, H. C. Lu, A. G. Hohmann | 2016 | Impact of Genetic Reduction of NMNAT2 on Chemotherapy-Induced Losses in Cell Viability In Vitro and Peripheral Neuropathy In Vivo | Yes | Yes | No |
| S. B. Smith, S. E. Crager, J. S. Mogil | 2004 | Paclitaxel-induced neuropathic hypersensitivity in mice: responses in 10 inbred mouse strains | No | No | No |
| J. A. Sprowl, G. Ciarimboli, C. S. Lancaster, et al | 2013 | Oxaliplatin-induced neurotoxicity is dependent on the organic cation transporter OCT2 | Yes | No | No |
| M. Sui, S. Lessans, T. Yan, D. Cao, L. Lao, S. G. Dorsey | 2016 | Mechanism of electroacupuncture on Zusanli (ST 36) for chemotherapy-induced peripheral neuropathy | No | Yes | No |
| T. Suzuki, K. Miyamoto, N. Yokoyama, et al | 2016 | Processed aconite root and its active ingredient neoline may alleviate oxaliplatin-induced peripheral neuropathic pain | Yes | No | No |
| T. Suzuki, A. Yamamoto, M. Ohsawa, Y. Motoo, H. Mizukami, T. Makino | 2017 | Effect of ninjin'yoeito and ginseng extracts on oxaliplatin-induced neuropathies in mice | Yes | No | No |
| S. M. Sweitzer, J. L. Pahl, J. A. DeLeo | 2006 | Propentofylline attenuates vincristine-induced peripheral neuropathy in the rat | Yes | No | No |
| J. Szilvassy, I. Sziklai, T. Racz, P. Horvath, G. Rabloczky, Z. Szilvassy | 2000 | Impaired bronchomotor responses to field stimulation in guinea-pigs with cisplatin-induced neuropathy | No | Yes | No |
| L. E. Ta, A. J. Bieber, S. M. Carlton, C. L. Loprinzi, P. A. Low, A. J. Windebank | 2010 | Transient Receptor Potential Vanilloid 1 is essential for cisplatin-induced heat hyperalgesia in mice | Yes | Yes | No |
| L. E. Ta, P. A. Low, A. J. Windebank | 2009 | Mice with cisplatin and oxaliplatin-induced painful neuropathy develop distinct early responses to thermal stimuli | Yes | Yes | No |
| L. E. Ta, J. D. Schmelzer, A. J. Bieber, et al | 2013 | A novel and selective poly (ADP-ribose) polymerase inhibitor ameliorates chemotherapy-induced painful neuropathy | Yes | Yes | No |
| O. Taleb, F. Bouzobra, H. Tekin-Pala, L. Meyer, A. G. Mensah-Nyagan, C. Patte-Mensah | 2016 | Behavioral and electromyographic assessment of oxaliplatin-induced motor dysfunctions: Evidence for a therapeutic effect of allopregnanolone | Yes | No | No |
| K. D. Tanner, J. D. Levine, K. S. Topp | 1998 | Microtubule disorientation and axonal swelling in unmyelinated sensory axons during vincristine-induced painful neuropathy in rat | Yes | Yes | No |
| K. D. Tanner, D. B. Reichling, R. W. Gear, S. M. Paul, J. D. Levine | 2003 | Altered temporal pattern of evoked afferent activity in a rat model of vincristine-induced painful peripheral neuropathy | No | No | No |
| K. D. Tanner, D. B. Reichling, J. D. Levine | 1998 | Nociceptor hyper-responsiveness during vincristine-induced painful peripheral neuropathy in the rat | No | No | No |
| A. Tasnim, Z. Rammelkamp, A. B. Slusher, K. Wozniak, B. S. Slusher, M. H. Farah | 2016 | Paclitaxel causes degeneration of both central and peripheral axon branches of dorsal root ganglia in mice | No | No | No |
| P. Tassler, A. L. Dellon, G. J. Lesser, S. Grossman | 2000 | Utility of decompressive surgery in the prophylaxis and treatment of cisplatin neuropathy in adult rats | No | No | No |
| Y. Tatsushima, N. Egashira, T. Kawashiri, et al | 2011 | Involvement of substance P in peripheral neuropathy induced by paclitaxel but not oxaliplatin | Yes | No | No |
| B. Tenci, L. Di Cesare Mannelli, M. Maresca, et al | 2017 | Effects of a water extract of Lepidium meyenii root in different models of persistent pain in rats | No | No | No |
| S. Teo | 2000 | Lack of peripheral neuropathy in Beagle dogs after 53 weeks oral administration of thalidomide capsules | No | No | No |
| M. P. Ter Laak, F. P. T. Hamers, C. J. Kirk, W. H. Gispen | 2000 | rhGGF2 protects against cisplatin-induced neuropathy in the rat | Yes | Yes | No |
| A. Thacheril Mohanan, S. Venkatesan, N. Sermugapandian, M. Al-Safhi, G. Khan | 2013 | Attenuating effect of Cilostazol against vincristine - Induced neuropathic pain in mice | No | Yes | No |
| D. Thangamani, I. O. Edafiogho, W. Masocha | 2013 | The anticonvulsant enaminone E139 attenuates paclitaxel-induced neuropathic pain in rodents | No | No | No |
| V. R. Thiagarajan, P. Shanmugam, U. M. Krishnan, A. Muthuraman | 2014 | Ameliorative effect of Vernonia cinerea in vincristine-induced painful neuropathy in rats | No | No | No |
| V. R. Thiagarajan, P. Shanmugam, U. M. Krishnan, A. Muthuraman, N. Singh | 2013 | Antinociceptive effect of Butea monosperma on vincristine-induced neuropathic pain model in rats | No | No | No |
| K. Thibault, B. Calvino, I. Rivals, et al | 2014 | Molecular mechanisms underlying the enhanced analgesic effect of oxycodone compared to morphine in chemotherapy-induced neuropathic pain | Yes | Yes | No |
| K. Thibault, B. Elisabeth, D. Sophie, F. Z. Claude, R. Bernard, C. Bernard | 2008 | Antinociceptive and anti-allodynic effects of oral PL37, a complete inhibitor of enkephalin-catabolizing enzymes, in a rat model of peripheral neuropathic pain induced by vincristine | Yes | Yes | No |
| K. Thibault, I. Rivals, S. Dahoma, S. Dubacq, S. Pezet, B. Calvino | 2013 | Structural and molecular alterations of primary afferent fibres in the spinal dorsal horn in vincristine-induced neuropathy in rat | Yes | Yes | No |
| L. Tian, T. Fan, N. Zhou, H. Guo, W. Zhang | 2015 | Role of PAR2 in regulating oxaliplatin-induced neuropathic pain via TRPA1 | No | No | No |
| G. C. Todd, W. J. Griffing, W. R. Gibson, D. M. Morton | 1979 | Animal models for the comparative assessment of neurotoxicity following repeated administration of vinca alkaloids | No | No | No |
| W. Toma, S. L. Kyte, D. Bagdas, et al | 2017 | Effects of paclitaxel on the development of neuropathy and affective behaviors in the mouse | Yes | Yes | Yes |
| K. Tomiwa, C. Nolan, J. B. Cavanagh | 1986 | The effects of cisplatin on rat spinal ganglia: a study by light and electron microscopy and by morphometry | No | No | No |
| M. Tomohisa, O. Junpei, M. Aki, et al | 2015 | Possible involvement of the Sigma-1 receptor chaperone in chemotherapeutic-induced neuropathic pain | Yes | No | No |
| R. Tonello, C. Fusi, S. Materazzi, et al | 2017 | The peptide Phalpha1beta, from spider venom, acts as a TRPA1 channel antagonist with antinociceptive effects in mice | Yes | Yes | Yes |
| K. S. Topp, K. D. Tanner, J. D. Levine | 2000 | Damage to the cytoskeleton of large diameter sensory neurons and myelinated axons in vincristine-induced painful peripheral neuropathy in the rat | Yes | No | No |
| S. Toyama, N. Shimoyama, Y. Ishida, T. Koyasu, H. H. Szeto, M. Shimoyama | 2014 | Characterization of acute and chronic neuropathies induced by oxaliplatin in mice and differential effects of a novel mitochondria-targeted antioxidant on the neuropathies | Yes | No | No |
| S. Toyama, N. Shimoyama, M. Shimoyama | 2017 | The analgesic effect of orexin-A in a murine model of chemotherapy-induced neuropathic pain | Yes | No | No |
| G. Tredici, M. Braga, G. Nicolini, et al | 1999 | Effect of recombinant human nerve growth factor on cisplatin neurotoxicity in rats | No | Yes | No |
| G. Tredici, G. Cavaletti, M. G. Petruccioli, D. Fabbrica, M. Tedeschi, P. Venturino | 1994 | Low-dose glutathione administration in the prevention of cisplatin- induced peripheral neuropathy in rats | No | No | No |
| M. Tsubaki, T. Takeda, T. Tani, et al | 2015 | PKC/MEK inhibitors suppress oxaliplatin-induced neuropathy and potentiate the antitumor effects | No | No | No |
| S. Tuncer, N. Dalkilic, M. Akif Dunbar, B. Keles | 2010 | Comparative effects of alpha lipoic acid and melatonin on cisplatin-induced neurotoxicity | No | No | No |
| E. Turkiew, D. Falconer, N. Reed, A. Hoke | 2017 | Deletion of Sarm1 gene is neuroprotective in two models of peripheral neuropathy | No | No | No |
| N. Uceyler, I. Kobsar, L. Biko, et al | 2006 | Heterozygous P0 deficiency protects mice from vincristine-induced polyneuropathy | No | No | No |
| H. Uchida, J. Nagai, H. Ueda | 2014 | Lysophosphatidic acid and its receptors LPA1 and LPA3 mediate paclitaxel-induced neuropathic pain in mice | No | No | No |
| H. Uchino, Y. Matsumura, T. Negishi, et al | 2005 | Cisplatin-incorporating polymeric micelles (NC-6004) can reduce nephrotoxicity and neurotoxicity of cisplatin in rats | No | Yes | No |
| M. L. Uhelski, I. A. Khasabova, D. A. Simone | 2015 | Inhibition of anandamide hydrolysis attenuates nociceptor sensitization in a murine model of chemotherapy-induced peripheral neuropathy | Yes | Yes | No |
| Q. L. Uy, T. H. Moen, R. J. Johns, A. H. Owens, Jr. | 1967 | Vincristine neurotoxicity in rodents | No | No | No |
| L. Van Helleputte, M. Kater, D. P. Cook, et al | 2017 | Inhibition of histone deacetylase 6 (HDAC6) protects against vincristine-induced peripheral neuropathies and inhibits tumor growth | No | No | No |
| B. Vashistha, A. Sharma, V. Jain | 2014 | Ameliorative potential of ferulic acid in vincristine-induced painful neuropathy in rats: An evidence of behavioral and biochemical examination | No | No | No |
| S. Vencappa, L. F. Donaldson, R. P. Hulse | 2015 | Cisplatin induced sensory neuropathy is prevented by vascular endothelial growth factor-A | No | No | No |
| G. Venkata, Ramalingayya, S. P. Cheruku, et al | 2017 | Rutin protects against neuronal damage in vitro and ameliorates doxorubicin-induced memory deficits in vivo in Wistar rats | Yes | No | No |
| G. Vera, P. A. Cabezos, M. I. Martin, R. Abalo | 2013 | Characterization of cannabinoid-induced relief of neuropathic pain in a rat model of cisplatin-induced neuropathy | No | Yes | No |
| G. Vera, A. Chiarlone, P. A. Cabezos, D. Pascual, M. I. Martin, R. Abalo | 2007 | WIN 55,212-2 prevents mechanical allodynia but not alterations in feeding behaviour induced by chronic cisplatin in the rat | No | No | No |
| E. Verdu, J. J. Vilches, F. J. Rodriguez, D. Ceballos, A. Valero, X. Navarro | 1999 | Physiological and immunohistochemical characterization of cisplatin- induced neuropathy in mice | Yes | No | No |
| A. Verheyen, E. Peeraer, D. Lambrechts, et al | 2013 | Therapeutic potential of VEGF and VEGF-derived peptide in peripheral neuropathies | No | No | No |
| A. Verheyen, E. Peeraer, R. Nuydens, et al | 2012 | Systemic anti-vascular endothelial growth factor therapies induce a painful sensory neuropathy | No | No | No |
| K. V. Viana-Cardoso, M. T. da Silva, R. C. Junior, et al | 2011 | Repeated cisplatin treatments inhibit gastrointestinal motility and induces baroreflex changes and mechanical hyperalgesia in rats | No | No | Yes |
| J. A. Vincent, K. B. Wieczerzak, H. M. Gabriel, P. Nardelli, M. M. Rich, T. C. Cope | 2016 | A novel path to chronic proprioceptive disability with oxaliplatin: Distortion of sensory encoding | Yes | Yes | No |
| C. F. Wan, L. L. Zheng, Y. Liu, X. Yu | 2016 | Houttuynia cordata Thunb reverses oxaliplatin-induced neuropathic pain in rat by regulating Th17/Treg balance | No | Yes | No |
| M. L. Wang, G. Yu, S. P. Yi, et al | 2015 | Antinociceptive effects of incarvillateine, a monoterpene alkaloid from Incarvillea sinensis, and possible involvement of the adenosine system | No | No | No |
| M. S. Wang, A. A. Davis, D. G. Culver, J. D. Glass | 2002 | Wld<sup>s</sup> mice are resistant to paclitaxel (Taxol) neuropathy | No | No | No |
| M. S. Wang, A. A. Davis, D. G. Culver, Q. Wang, J. C. Powers, J. D. Glass | 2004 | Calpain inhibition protects against Taxol-induced sensory neuropathy | No | No | No |
| Y. Wang, S. E. Cao, J. Tian, G. Liu, X. Zhang, P. Li | 2013 | Auraptenol attenuates vincristine-induced mechanical hyperalgesia through serotonin 5-HT1A receptors | No | No | No |
| Y. S. Wang, Y. Y. Li, W. Cui, et al | 2017 | Melatonin Attenuates Pain Hypersensitivity and Decreases Astrocyte-Mediated Spinal Neuroinflammation in a Rat Model of Oxaliplatin-Induced Pain | Yes | No | No |
| S. J. Ward, S. D. McAllister, R. Kawamura, R. Murase, H. Neelakantan, E. A. Walker | 2014 | Cannabidiol inhibits paclitaxel-induced neuropathic pain through 5-HT(1A) receptors without diminishing nervous system function or chemotherapy efficacy | No | No | No |
| S. J. Ward, M. D. Ramirez, H. Neelakantan, E. A. Walker | 2011 | Cannabidiol prevents the development of cold and mechanical allodynia in paclitaxel-treated female C57Bl6 mice | No | No | No |
| M. Waseem, H. Tabassum, S. Parvez | 2016 | Neuroprotective effects of melatonin as evidenced by abrogation of oxaliplatin induced behavioral alterations, mitochondrial dysfunction and neurotoxicity in rat brain | No | No | No |
| H. Watanabe, T. Kawashiri, S. Ushio, et al | 2014 | Neurotropin relieves oxaliplatin-induced neuropathy via G<inf>i</inf> protein-coupled receptors in the monoaminergic descending pain inhibitory system | Yes | No | No |
| J. Y. Wei, C. C. Liu, H. D. Ouyang, et al | 2017 | Activation of RAGE/STAT3 pathway by methylglyoxal contributes to spinal central sensitization and persistent pain induced by bortezomib | Yes | Yes | No |
| I. Weissman-Fogel, A. Dashkovsky, Z. Rogowski, D. Yarnitsky | 2008 | Vagal damage enhances polyneuropathy pain: additive effect of two algogenic mechanisms | Yes | No | No |
| H. R. Weng, N. Aravindan, J. P. Cata, J. H. Chen, A. D. S. Shaw, P. M. Dougherty | 2005 | Spinal glial glutamate transporters downregulate in rats with taxol-induced hyperalgesia | No | No | No |
| H. R. Weng, J. V. Cordella, P. M. Dougherty | 2003 | Changes in sensory processing in the spinal dorsal horn accompany vincristine-induced hyperalgesia and allodynia | No | No | No |
| P. M. Whitaker-Azmitia, M. Raio, D. Raio, A. Borella | 1995 | A 5-HT 3 receptor antagonist fails to prevent cisplatin-induced toxicity in immature rat spinal cord | Yes | No | No |
| J. L. Wilkerson, S. Ghosh, D. Bagdas, et al | 2016 | Diacylglycerol lipase beta inhibition reverses nociceptive behaviour in mouse models of inflammatory and neuropathic pain | Yes | Yes | No |
| S. A. Woller, M. Corr, T. L. Yaksh | 2015 | Differences in cisplatin-induced mechanical allodynia in male and female mice | No | No | No |
| T. Wongtawatchai, S. Agthong, A. Kaewsema, V. Chentanez | 2009 | Sex-related differences in cisplatin-induced neuropathy in rats | No | No | No |
| T. Wongtawatchai, S. Agthong, A. Kaewsema, V. Chentanez | 2012 | Altered phosphorylation of mitogen-activated protein kinases in dorsal root ganglia and sciatic nerve of rats with cisplatin-induced neuropathy | No | No | No |
| P. K. Working, M. S. Newman, T. Sullivan, et al | 1998 | Comparative intravenous toxicity of cisplatin solution and cisplatin encapsulated in long-circulating, pegylated liposomes in cynomolgus monkeys | No | No | No |
| K. M. Wozniak, K. Nomoto, R. G. Lapidus, et al | 2011 | Comparison of neuropathy-inducing effects of eribulin mesylate, paclitaxel, and ixabepilone in mice | No | Yes | No |
| K. M. Wozniak, J. J. Vornov, Y. Wu, et al | 2017 | Peripheral neuropathy induced by microtubule-targeted chemotherapies: insights into acute injury and long-term recovery | Yes | Yes | No |
| K. M. Wozniak, J. J. Vornov, Y. Wu, et al | 2016 | Sustained Accumulation of Microtubule-Binding Chemotherapy Drugs in the Peripheral Nervous System: Correlations with Time Course and Neurotoxic Severity | No | No | No |
| K. M. Wozniak, Y. Wu, M. H. Farah, B. A. Littlefield, K. Nomoto, B. S. Slusher | 2013 | Neuropathy-inducing effects of eribulin mesylate versus paclitaxel in mice with preexisting neuropathy | No | No | No |
| K. M. Wozniak, Y. Wu, J. J. Vornov, et al | 2012 | The orally active glutamate carboxypeptidase II inhibitor E2072 exhibits sustained nerve exposure and attenuates peripheral neuropathy | Yes | Yes | No |
| Y. Wu, J. Li, J. Zhou, Y. Feng | 2014 | Dynamic long-term microstructural and ultrastructural alterations in sensory nerves of rats of paclitaxel-induced neuropathic pain | Yes | Yes | No |
| Y. Q. Wu, R. L. Dang, M. M. Tang, et al | 2016 | Long Chain Omega-3 Polyunsaturated Fatty Acid Supplementation Alleviates Doxorubicin-Induced Depressive-Like Behaviors and Neurotoxicity in Rats: Involvement of Oxidative Stress and Neuroinflammation | Yes | Yes | No |
| Z. Wu, S. Wang, I. Wu, M. Mata, D. J. Fink | 2015 | Activation of TLR-4 to produce tumour necrosis factor-alpha in neuropathic pain caused by paclitaxel | No | No | No |
| W. Xiao, A. Boroujerdi, G. J. Bennett, Z. D. Luo | 2007 | Chemotherapy-evoked painful peripheral neuropathy: analgesic effects of gabapentin and effects on expression of the alpha-2-delta type-1 calcium channel subunit | No | No | No |
| W. H. Xiao, G. J. Bennett | 2008 | Chemotherapy-evoked neuropathic pain: Abnormal spontaneous discharge in A-fiber and C-fiber primary afferent neurons and its suppression by acetyl-L-carnitine | Yes | Yes | No |
| W. H. Xiao, F. Y. Zheng, G. J. Bennett, T. Bordet, R. M. Pruss | 2009 | Olesoxime (cholest-4-en-3-one, oxime): analgesic and neuroprotective effects in a rat model of painful peripheral neuropathy produced by the chemotherapeutic agent, paclitaxel | Yes | Yes | No |
| W. H. Xiao, H. Zheng, G. J. Bennett | 2012 | Characterization of oxaliplatin-induced chronic painful peripheral neuropathy in the rat and comparison with the neuropathy induced by paclitaxel | Yes | No | No |
| W. H. Xiao, H. Zheng, F. Y. Zheng, R. Nuydens, T. F. Meert, G. J. Bennett | 2011 | Mitochondrial abnormality in sensory, but not motor, axons in paclitaxel-evoked painful peripheral neuropathy in the rat | No | No | No |
| J. D. Xie, S. R. Chen, H. Chen, H. L. Pan | 2017 | Bortezomib induces neuropathic pain through protein kinase C-mediated activation of presynaptic NMDA receptors in the spinal cord | No | No | No |
| F. Xu, S. Xu, L. Wang, et al | 2011 | Antinociceptive efficacy of verticinone in murine models of inflammatory pain and paclitaxel induced neuropathic pain | No | No | No |
| J. Xu, W. Wang, X. X. Zhong, Y. Feng, X. Wei, X. G. Liu | 2016 | Methylcobalamin ameliorates neuropathic pain induced by vincristine in rats: Effect on loss of peripheral nerve fibers and imbalance of cytokines in the spinal dorsal horn | Yes | No | No |
| J. J. Xu, P. Diaz, B. Bie, et al | 2014 | Spinal gene expression profiling and pathways analysis of a CB<inf>2</inf> agonist (MDA7)-targeted prevention of paclitaxel-induced neuropathy | Yes | No | No |
| T. Xu, D. Li, X. Zhou, et al | 2017 | Oral Application of Magnesium-L-Threonate Attenuates Vincristine-induced Allodynia and Hyperalgesia by Normalization of Tumor Necrosis Factor-alpha/Nuclear Factor-kappaB Signaling | Yes | Yes | No |
| T. Xu, X. L. Zhang, H. D. Ou-Yang, et al | 2017 | Epigenetic upregulation of CXCL12 expression mediates antitubulin chemotherapeutics-induced neuropathic pain | No | No | No |
| Y. Xu, G. Cheng, Y. Zhu, et al | 2016 | Anti-nociceptive roles of the glia-specific metabolic inhibitor fluorocitrate in paclitaxel-evoked neuropathic pain | No | No | No |
| S. Yalcin, S. Kilickap, C. M. Temucin, M. Erman | 2006 | Recombinant human erythropoietin in comparison to amifostine against cisplatin-induced peripheral sensorial neurotoxicity in rats | No | Yes | No |
| S. Yalcin, G. Nurlu, B. Orhan, et al | 2003 | Protective effect of amifostine against cisplatin-induced motor neuropathy in rat | No | Yes | No |
| K. Yamamoto, N. Chiba, T. Chiba, et al | 2015 | Transient receptor potential ankyrin 1 that is induced in dorsal root ganglion neurons contributes to acute cold hypersensitivity after oxaliplatin administration | Yes | No | No |
| K. Yamamoto, M. Tsuboi, T. Kambe, et al | 2016 | Oxaliplatin administration increases expression of the voltage-dependent calcium channel alpha2delta-1 subunit in the rat spinal cord | Yes | No | No |
| S. Yamamoto, T. Kawashiri, H. Higuchi, et al | 2015 | Behavioral and pharmacological characteristics of bortezomib-induced peripheral neuropathy in rats | No | No | No |
| S. Yamamoto, H. Ono, K. Kume, M. Ohsawa | 2016 | Oxaliplatin treatment changes the function of sensory nerves in rats | No | No | No |
| Y. Yamashita, N. Egashira, K. Masuguchi, S. Ushio, T. Kawashiri, R. Oishi | 2011 | Comparison of peripheral neuropathy induced by standard and nanoparticle albumin-bound paclitaxel in rats | No | No | No |
| Y. Yamashita, K. Irie, A. Kochi, et al | 2017 | Involvement of Charcot-Marie-Tooth disease gene mitofusin 2 expression in paclitaxel-induced mechanical allodynia in rats | No | No | No |
| F. Yan, J. J. Liu, V. Ip, S. M. Jamieson, M. J. McKeage | 2015 | Role of platinum DNA damage-induced transcriptional inhibition in chemotherapy-induced neuronal atrophy and peripheral neurotoxicity | No | No | No |
| X. Yan, D. W. Maixner, R. Yadav, et al | 2015 | Paclitaxel Induces Acute Pain via Directly Activating Toll like Receptor 4 | Yes | Yes | No |
| Y. Yang, Y. G. Zhang, G. A. Lin, et al | 2014 | Spinal changes of a newly isolated neuropeptide endomorphin-2 concomitant with vincristine-induced allodynia | Yes | No | No |
| J. H. Yeo, S. Y. Yoon, S. J. Kim, et al | 2016 | Clonidine, an alpha-2 adrenoceptor agonist relieves mechanical allodynia in oxaliplatin-induced neuropathic mice; potentiation by spinal p38 MAPK inhibition without motor dysfunction and hypotension | Yes | Yes | No |
| J. H. Yeo, S. Y. Yoon, S. K. Kwon, et al | 2016 | Repetitive acupuncture point treatment with diluted bee venom relieves mechanical allodynia and restores intraepidermal nerve fiber loss in oxaliplatin-induced neuropathic mice | Yes | Yes | Yes |
| E. Yilmaz, M. S. Gold | 2015 | Sensory neuron subpopulation-specific dysregulation of intracellular calcium in a rat model of chemotherapy-induced peripheral neuropathy | Yes | No | No |
| E. Yilmaz, M. S. Gold | 2016 | Paclitaxel-induced increase in NCX activity in subpopulations of nociceptive afferents: A protective mechanism against chemotherapy-induced peripheral neuropathy? | No | No | No |
| H. Yoon, M. J. Kim, I. Yoon, D. X. Li, H. Bae, S. K. Kim | 2015 | Nicotinic Acetylcholine Receptors Mediate the Suppressive Effect of an Injection of Diluted Bee Venom into the GV3 Acupoint on Oxaliplatin-Induced Neuropathic Cold Allodynia in Rats | No | Yes | No |
| M. S. Yoon, Z. Katsarava, M. Obermann, et al | 2009 | Erythropoietin overrides the triggering effect of DNA platination products in a mouse model of cisplatin-induced neuropathy | Yes | Yes | No |
| S. Y. Yoon, C. R. Robinson, H. Zhang, P. M. Dougherty | 2013 | Spinal astrocyte gap junctions contribute to oxaliplatin-induced mechanical hypersensitivity | Yes | No | No |
| S. Y. Yoon, J. H. Yeo, S. D. Han, D. J. Bong, B. Oh, D. H. Roh | 2013 | Diluted bee venom injection reduces ipsilateral mechanical allodynia in oxaliplatin-induced neuropathic mice | Yes | Yes | No |
| M. Zanardelli, L. Micheli, L. Cinci, P. Failli, C. Ghelardini, L. Di Cesare Mannelli | 2014 | Oxaliplatin neurotoxicity involves peroxisome alterations. PPARgamma agonism as preventive pharmacological approach | Yes | Yes | No |
| C. E. Zbarcea, I. C. Ciotu, V. Bild, et al | 2017 | Therapeutic potential of certain drug combinations on paclitaxel-induced peripheral neuropathy in rats | No | No | No |
| C. E. Zbarcea, S. Negres, C. Chirita | 2011 | Gabapentin, alone and associated with tramadol reduces peripheral paclitaxel-induced neuropathy in rats | No | No | No |
| C. E. Zbarcea, S. Negres, A. Nicoleta Cristea, C. Chirita | 2011 | The effect of dextromethorphan, gabapentin, amitriptyline and tramadol on a mouse model of vincristine - induced peripheral neuropathy | No | No | No |
| H. Zhang, J. A. Boyette-Davis, A. K. Kosturakis, et al | 2013 | Induction of monocyte chemoattractant protein-1 (mcp-1) and its receptor ccr2 in primary sensory neurons contributes to paclitaxel-induced peripheral neuropathy | Yes | No | No |
| H. Zhang, P. M. Dougherty | 2014 | Enhanced excitability of primary sensory neurons and altered gene expression of neuronal ion channels in dorsal root ganglion in paclitaxel-induced peripheral neuropathy | Yes | No | No |
| H. Zhang, Y. Li, M. de Carvalho-Barbosa, et al | 2016 | Dorsal Root Ganglion Infiltration by Macrophages Contributes to Paclitaxel Chemotherapy-Induced Peripheral Neuropathy | Yes | No | No |
| J. Zhang, Y. M. Su, D. Li, et al | 2014 | TNF-alpha-mediated JNK activation in the dorsal root ganglion neurons contributes to Bortezomib-induced peripheral neuropathy | Yes | No | No |
| J. Zhang, R. P. Tuckett | 2008 | Comparison of paclitaxel and cisplatin effects on the slowly adapting type I mechanoreceptor | No | Yes | No |
| Y. Zhang, A. Li, J. Xin, et al | 2017 | Electroacupuncture alleviates chemotherapy-induced pain through inhibiting phosphorylation of spinal CaMKII in rats | Yes | Yes | Yes |
| M. Zhao, K. Isami, S. Nakamura, H. Shirakawa, T. Nakagawa, S. Kaneko | 2012 | Acute cold hypersensitivity characteristically induced by oxaliplatin is caused by the enhanced responsiveness of TRPA1 in mice | No | No | No |
| M. Zhao, S. Nakamura, T. Miyake, et al | 2014 | Pharmacological characterization of standard analgesics on oxaliplatin-induced acute cold hypersensitivity in mice | No | No | No |
| F. Y. Zheng, W. H. Xiao, G. J. Bennett | 2011 | The response of spinal microglia to chemotherapy-evoked painful peripheral neuropathies is distinct from that evoked by traumatic nerve injuries | No | No | No |
| H. Zheng, W. H. Xiao, G. J. Bennett | 2011 | Functional deficits in peripheral nerve mitochondria in rats with paclitaxel- and oxaliplatin-evoked painful peripheral neuropathy | Yes | No | No |
| F. H. Zhou, Y. Yu, X. F. Zhou, C. J. Xian | 2015 | Methotrexate chemotherapy triggers touch-evoked pain and increased CGRP-positive sensory fibres in the tibial periosteum of young rats | Yes | No | No |
| H. H. Zhou, L. Zhang, Q. G. Zhou, Y. Fang, W. H. Ge | 2016 | (+)-Borneol attenuates oxaliplatin-induced neuropathic hyperalgesia in mice | No | Yes | No |
| W. Zhou, A. Kavelaars, C. J. Heijnen | 2016 | Metformin prevents cisplatin-induced cognitive impairment and brain damage in mice | Yes | No | No |
| H. Q. Zhu, J. Xu, K. F. Shen, R. P. Pang, X. H. Wei, X. G. Liu | 2015 | Bulleyaconitine A depresses neuropathic pain and potentiation at C-fiber synapses in spinal dorsal horn induced by paclitaxel in rats | No | No | No |
| J. Zhu, V. A. Carozzi, N. Reed, et al | 2016 | Ethoxyquin provides neuroprotection against cisplatin-induced neurotoxicity | No | Yes | No |
| J. Zhu, W. Chen, R. Mi, C. Zhou, N. Reed, A. Hoke | 2013 | Ethoxyquin prevents chemotherapy-induced neurotoxicity via Hsp90 modulation | No | Yes | No |
